# Unusually Chemoselective Photocyclization of 2-(Hydroxyimino)aldehydes to Cyclobutanol Oximes: Synthetic, Stereochemical, and Mechanistic Aspects

**DOI:** 10.1021/acs.joc.2c01503

**Published:** 2022-10-05

**Authors:** Antonio Di Sabato, Francesca D’Acunzo, Dario Filippini, Fabrizio Vetica, Antonio Brasiello, Davide Corinti, Enrico Bodo, Cinzia Michenzi, Edoardo Panzetta, Patrizia Gentili

**Affiliations:** † Department of Chemistry, Sapienza University of Rome, Piazzale Aldo Moro 5, 00185 Rome, Italy; ‡ Institute of Biological Systems (ISB), Sezione Meccanismi di Reazione, Italian National Research Council (CNR), c/o Department of Chemistry, Sapienza University of Rome, Piazzale Aldo Moro 5, 00185 Rome, Italy; § Department of Chemical Engineering Materials Environment, Sapienza University of Rome, via Eudossiana 18, 00184 Rome, Italy; ∥ Department of Chemistry and Technology of Drugs, Sapienza University of Rome, Piazzale Aldo Moro 5, 00185 Rome, Italy

## Abstract

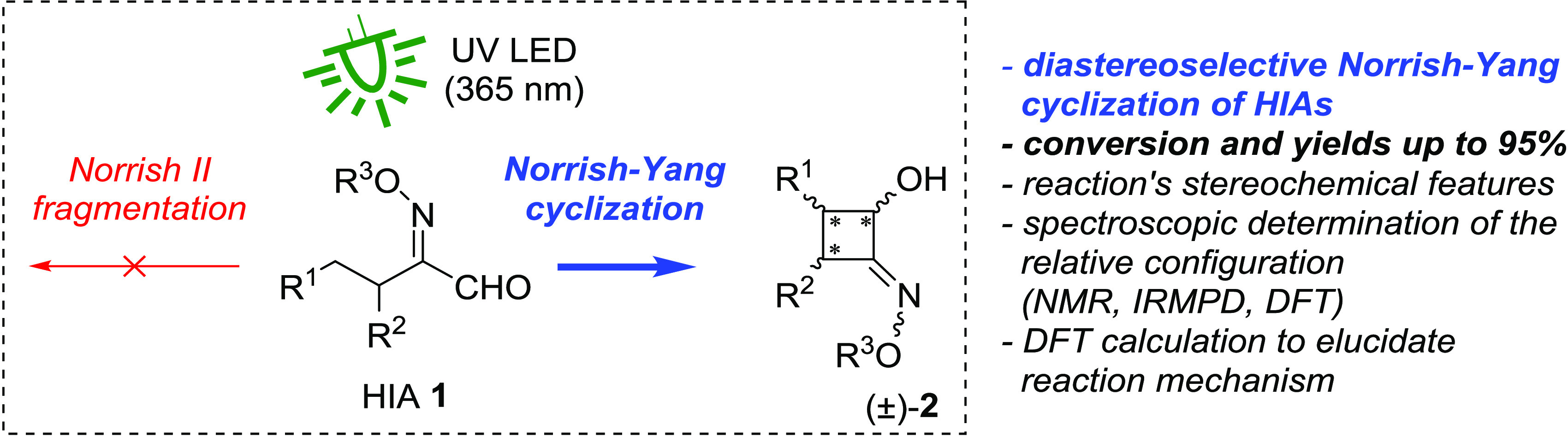

Photocyclization of carbonyl compounds (known as the Norrish–Yang reaction) to yield cyclobutanols is, in general, accompanied by fragmentation reactions. The latter are predominant in the case of aldehydes so that secondary cyclobutanols are not considered accessible via the straightforward Norrish–Yang reaction. A noteworthy exception has been reported in our laboratory, where cyclobutanols bearing a secondary alcohol function were observed upon UV light irradiation of 2-(hydroxyimino)aldehydes (HIAs). This reaction is here investigated in detail by combining synthesis, spectroscopic data, molecular dynamics, and DFT calculations. The synthetic methodology is generally applicable to a series of HIAs, affording the corresponding cyclobutanol oximes (CBOs) chemoselectively (i.e., without sizable fragmentation side-reactions), diastereoselectively (up to >99:1), and in good to excellent yields (up to 95%). CBO oxime ether derivatives can be purified and diastereomers isolated by standard column chromatography. The mechanistic and stereochemical picture of this photocyclization reaction, as well as of the postcyclization *E*/Z isomerization of the oxime double bond is completed.

## Introduction

**Scheme 1:**
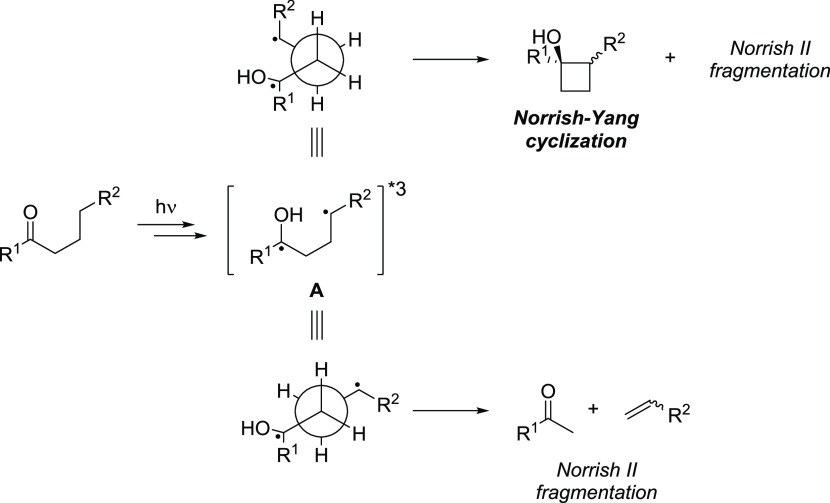
Norrish–Yang and Norrish II Products from Photoactivation of Carbonyl Compounds Cyclization (top) requires rotation of σ-bonds to ensure the orbital overlap and allow the ring closure with different degrees of diastereoselectivity, depending on the substrate structure. Fragmentation (bottom) can also occur from a transoid configuration of the radicals involved.

A renewed interest has been experienced recently in synthetic photochemical processes, thanks to the availability of convenient light sources.^1−4^ Light can be used to access reaction pathways that are forbidden in the ground state. In many cases, this is achieved in the absence of costly and polluting artificial catalysts, thus abiding by the principles of green chemistry in full. The Norrish–Yang (N–Y) reaction is a photoactivated transformation of carbonyl compounds that does not require the presence of a catalyst and leads to the formation of four-membered rings, the only requirement being the presence of a γ-H in the substrate.^5^ By general consensus, it occurs when the n−π* transition of a carbonyl group leads, *via* intramolecular abstraction of γ-H, to the generation of an excited 1,4-biradical triplet state, which then undergoes cyclization to afford four-membered rings (Scheme 1).

The excited biradical can also undergo a C_α_–C_β_ bond cleavage (Norrish II fragmentation), and the prevalence of cyclization over fragmentation is determined by geometric requirements.^6,7^ It should be noted that the mechanism involving spin-forbidden transitions may be complemented by “singlet state only” Norrish pathways.^8^ Norrish-Type I fragmentation, consisting of the cleavage of the carbonyl–C_α_ bond may also occur (not shown).^9^

Since its discovery in 1958, the N–Y reaction has proven of wide scope and synthetic usefulness. It is mostly known to afford cyclobutanols bearing a tertiary alcohol function from variously substituted, mostly aromatic ketones,^10−24^ 1,2-diketones,^25^ α-oxooximes,^26,27^ and β-lactams from α-oxoamides^28,29^ or β-ketoamides,^30,31^ respectively. The scope of the N–Y reaction has been extended also to larger-membered cyclic products *via* remote intramolecular H-abstraction by substrate design.^3,23^

Two main aspects are especially relevant for the applicability of the N–Y reaction in organic synthesis, i.e., the chemoselectivity of cyclization *vs*. Norrish I and II fragmentation reactions and the stereoselectivity of the ring closure (Scheme 1). The latter generates at least two stereogenic centers from an achiral precursor (*vide infra*, Scheme 3). Therefore, characterizing, understanding, and harnessing the stereochemical aspects of this reaction is of great synthetic value. Both the incidence of the Norrish II fragmentation and the stereoselectivity of cyclization are related to conformational changes in the triplet state (Scheme 1).^13^

**Scheme 2:**
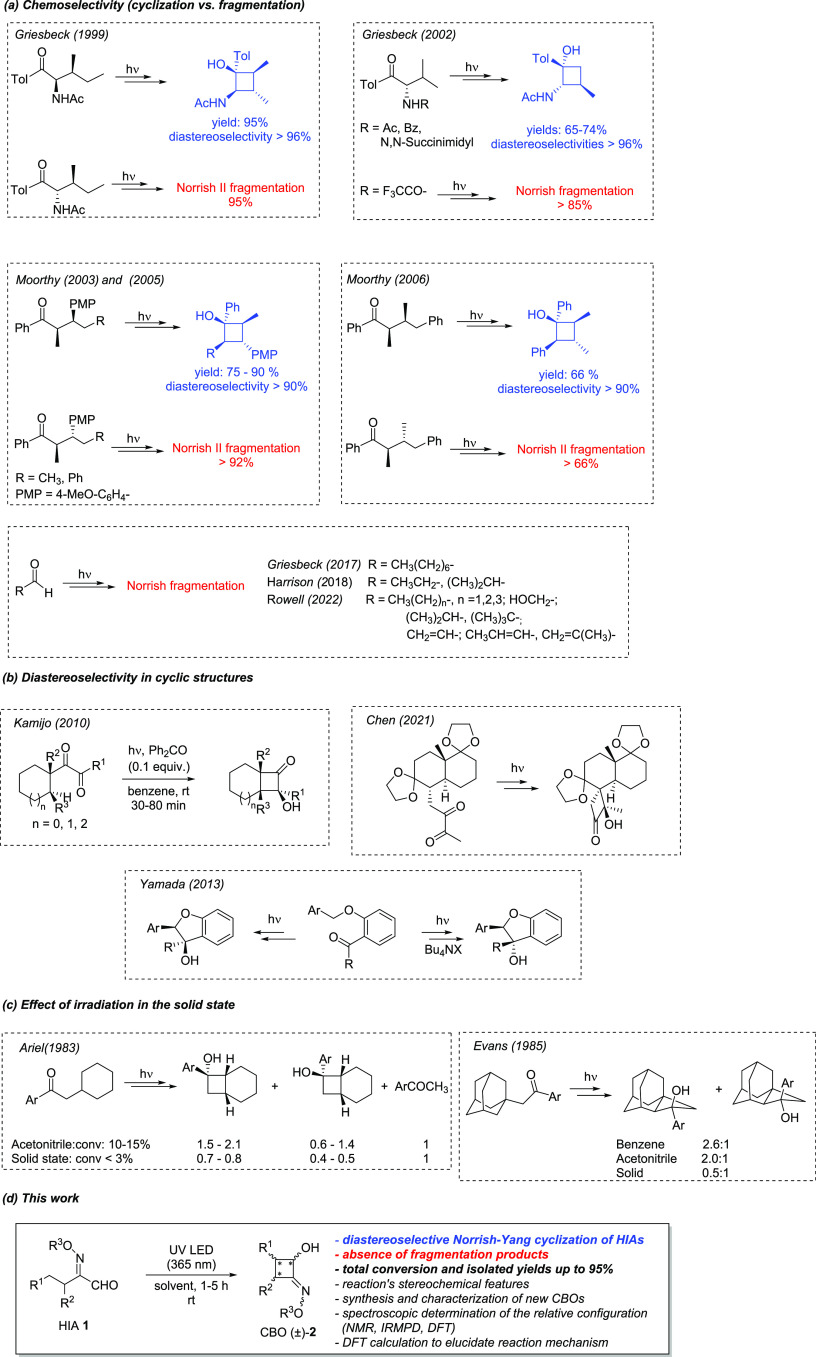
Examples of Chemo- and Diastereoselectivity of Photoreactions of Carbonyl Compounds Resulting from Structural Features and Reaction Conditions (a) Chemoselectivity induced by the stereochemistry^14,20,32^ or by the hydrogen bonding properties^15^ of substituents. The ring closure in these cases is generally diastereoselective in a solvent-dependent manner.^16^ (b) Diastereoselective photocyclization of cyclic structures induced by hydrogen bonding^22,24^ or cation−π interactions^23^ in the transition state. (c) Effect of the crystal structure in solid-state irradiation: stereoselectivity reversal^11^ and chemoselectivity.^10^

Therefore, researchers have been directing the fate of the photochemical reactions of carbonyls by affecting the conformational freedom through different strategies (Scheme 2). These include varying the stereochemistry or hydrogen bonding ability of substituents,^13−15,20,24,28,32^ reacting cyclic or bicyclic structures,^11,22,24^ changing solvents and introducing hydrogen bonding or tetralkylammonium-π interactions,^16,21,23^ running the reaction in the solid state,^10,17^ and linking chiral auxiliaries.^28^

Quantum chemical methods based on the calculation of accurate and reliable electronic energies are routinely used to predict and explain the outcome of chemical reactions.^33^ In particular, the N–Y reaction has been studied previously using DFT methods by Tang and Paton.^34^ Their conclusion is that the reaction stereochemistry is determined by a very rapid diradical recombination instead of emerging from following the minimum energy path.

The computational problem when facing a reaction such as N–Y lies in the correct treatment of a diradical singlet state. This in principle, can be achieved using a multireference method, but when the system size increases, correlating such a solution becomes problematic. Another option, the one we have used here, consists of using DFT with a functional that accounts for a suitable treatment of nonlocality effects.^35^

Within the framework of our continuing investigation of the 2-(hydroxyimino)aldehyde (HIA) functionality, as a versatile structure in multistimuli-sensitive polymers, we have been studying the physico- and photochemical properties of several HIAs.^36−38^

Our initial studies on different aliphatic HIAs were carried out by irradiation with high-pressure mercury lamps (λ = 365 nm with a large bandwidth of about 100 nm) that cover the forbidden n−π* carbonyl transition, and, to some extent, allowed the oxime π–π* transition. Thus, both Norrish-type reactions (Scheme 1 and Norrish I) and *E*/Z-oxime configurational isomerization were potentially triggered. In fact, we observed that along with the relatively fast (2 h) substituent-dependent *E*/*Z* isomerism, N–Y photoisomerization to cyclobutanol oximes (CBOs) occurred over longer irradiation times (>20 h) leaving, however, large and variable fractions of unreacted HIA. We found no evidence of concurring Norrish I and II fragmentations. In subsequent studies, we began using LED sources (λ = 365 ± 10 nm) to stimulate the aldehyde group selectively on HIAs linked to polymethacrylates and to 1,2,3-triazoles. The overall outcome was the same in all cases, i.e., HIA conversion was quantitative within 3–4 h and the only detected photoproducts were CBOs. Only in those cases in which a different substituent competes for the same wavelength does the HIA fail to yield the corresponding CBO.^39,40^ So, we proved that HIAs and LED sources are a promising combination in terms of excellent conversions, convenient reaction time, and the absence of undesired products, for the synthesis of secondary cyclobutanols from aldehydes. It is worth noting that, in general, the clean N–Y reaction is not easily obtained, and, more specifically, to our knowledge the synthetically relevant N–Y reaction of aldehydes, which afford four-membered rings bearing the secondary alcohol, has only been reported by our laboratory.^37,38^ In fact, the photochemistry of aldehydes generally results in fragmentation reactions that are of importance in atmospheric chemistry,^41,42^ for light-induced fragrance release,^43,44^ and for photocleavable polymeric materials.^45^ In the case of HIAs, instead, we observed that the exclusive N–Y reaction occurs in solution without the need for conformationally demanding substituents or templates. However, in our previous work, our results were limited to small-scale mixtures of diastereomers of unidentified stereochemistry, so it was clear that more work needed to be done to elucidate the stereochemistry of products and to impart any synthetic value to the N–Y cyclization of HIAs. In the synthetic aspect of the present work, we somewhat expand the substrate scope and we address scale-up and isolation of stereoisomers. From a more mechanistic point of view, we determine experimentally the stereoselectivity of the ring closure by detailed NMR analysis, and we perform DFT calculations to help elucidate the energetic factors involved. Molecular dynamics is used to explore CBO-solvent interactions and their role in postcyclization oxime *E*/*Z* isomerism.^38^ NMR-based absolute configuration assignment is also explored.

## Results and Discussion

### Photoinduced Norrish–Yang Cyclization of 2-(Hydroxyimino)aldehydes

First, variously substituted 2-(hydroxyimino)aldehydes **1a–j** were efficiently synthesized, starting from the corresponding aldehydes *via* α-oximation reaction (see the SI).^12^ The presence of either a secondary or tertiary C3 position (R^2^ = H and R^2^ ≠ H, respectively) was envisioned since the latter case would lead to the generation of a third stereocenter during the photoisomerization (**2g–j**).

**Scheme 3:**
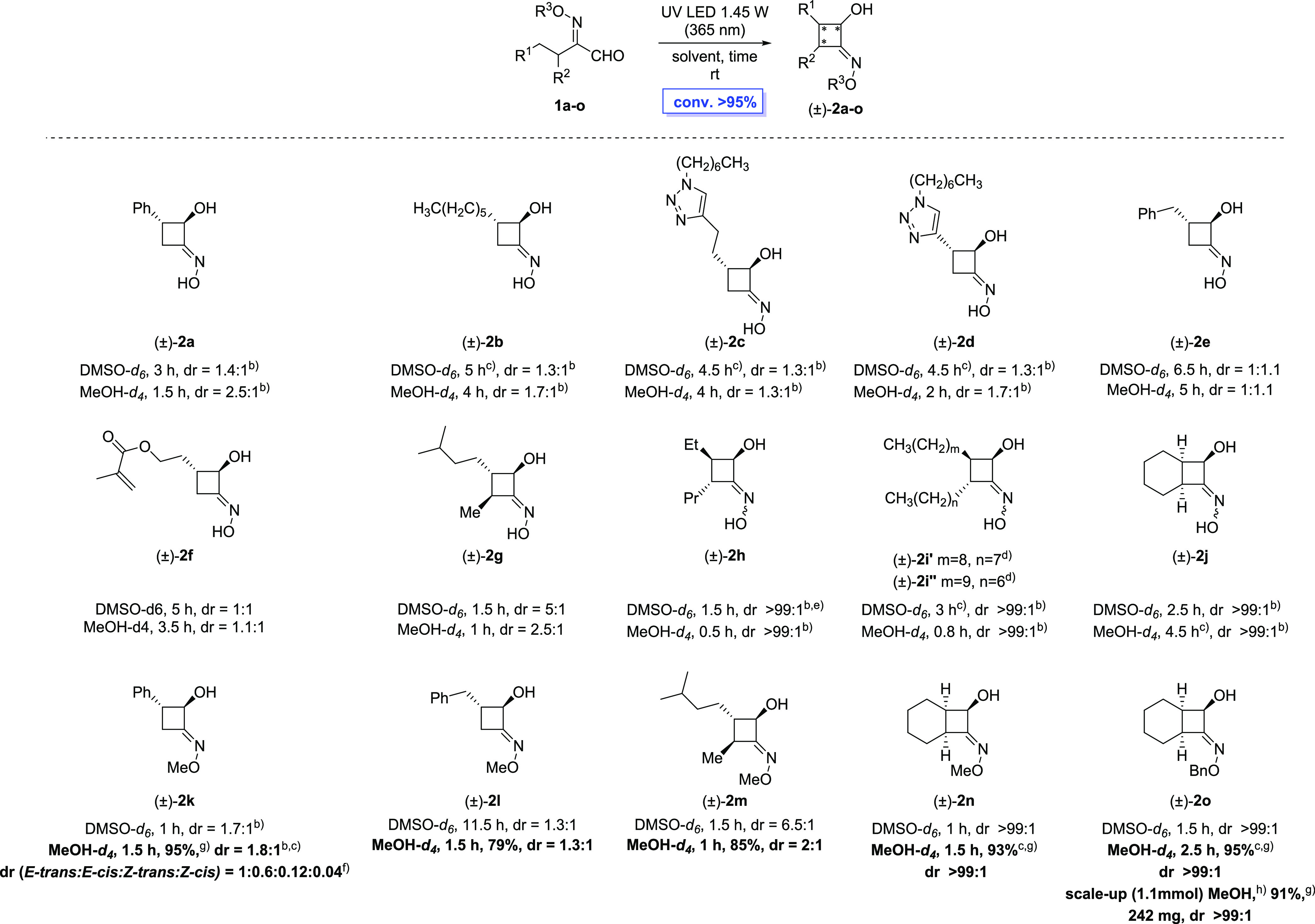
Diastereoselective Photoinduced Norrish–Yang Cyclization of 2-(Hydroxyimino)aldehydes (HIAs) [a] The substrate (0.060–0.075 mmol) was dissolved in the appropriate deuterated solvent (600–750 μL, *C* = 0.1 M) in an NMR quartz tube. The sample was placed in a photoreactor using 1450 mW LED radiant flux; [b] dr values corresponding to the *trans*:*cis* ring-closure products determined via ^1^H NMR, and major diastereoisomers are depicted for each compound; [c] Same reaction conditions, except for 1030 mW LED radiant flux; [d] since the H-abstraction could occur on the two different alkyl chains of substrate **1i**, the exact structure of the product could not be determined; [e] a third diastereoisomer was observed (ca. 10%), ascribable to the relative *cis* configuration of Et and Pr substituents; [f] all diastereoisomers were isolated via flash column chromatography and characterized; [g] isolated yield after flash column chromatography; and [h] scale-up experiment starting from 1.1 mmol of **1j** dissolved in 4.3 mL of MeOH (*C* = 0.25 M). Reaction completion was achieved in 13 h.

Additionally, we synthesized the methylated derivatives of different HIAs (**1a**, **1e**, **1g**, **1j**), and the benzylated **1j** to test for any differences in terms of reactivity and diastereoselectivity in the photoreactions (*vide infra*, Scheme 3).

We selected 2-(hydroxyimino)-4-phenylbutanal **1a** as the model substrate to evaluate the effect of the solvent and light intensity on the reaction outcome (Table 1). The photoreactions were carried out directly in deuterated solvents in NMR tubes with LED sources (λ = 365 nm) of two different powers of irradiation (radiant flux: 1030 and 1450 mW) and were monitored *via*^1^H NMR.^36−38^ Care was taken to prevent sample heating for a fair comparison of results (see the SI for details on the reaction apparatus).

**Table 1:**
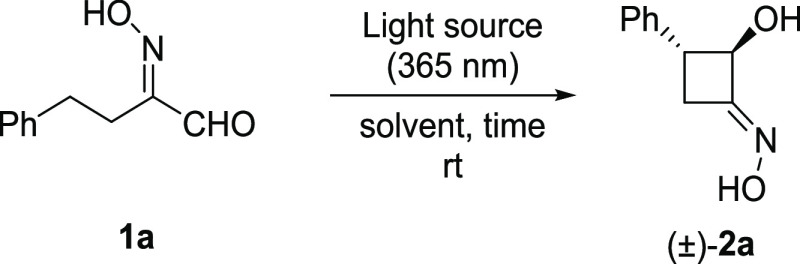
Screening of the Reaction Conditions for the Photoisomerization of **1a**

entry^a^	solvent	time (h)	dr^c^
1^b^	DMSO-*d*_6_	4.0	1.4:1
2	DMSO-*d*_6_	3.0	1.4:1
3	MeOH-*d*_4_	1.5	2.5:1
4	ACN-*d*_3_	5.0	2.5:1
5	DCM-*d*_2_		decomp^d^
6	benzene-*d*_6_		decomp^d^

a The substrate (0.060–0.075 mmol) was dissolved in the appropriate deuterated solvent (600–750 μL, *C* = 0.1 M) in an NMR quartz tube. The sample was placed in a photoreactor using 1450 mW LED radiant flux.

b Same reaction conditions, except for 1030 mW LED radiant flux.

c dr values corresponding to the *trans:cis* diastereomers determined *via*^1^H NMR.

d decomposition yielded a complex mixture of products.

Then we used the most intense LED source for the continuation of this study (Table 1, entries 3–6). The relative configuration of the two stereocenters was assigned on the basis of the *J* coupling constants, in accordance with literature data (*vide infra*),^46^ the major diastereoisomer being *trans*-**2a**. HRMS analysis confirmed that the observed photoreaction is an isomerization, the mass of the product being the same as that of the substrate. The collision-induced dissociation (CID) analyses of HIA **1a** and CBO **2a**, carried out in negative ion mode, showed different fragmentation patterns. While **1a** displayed only a peak corresponding to the [M–H]^−^ ion, **2a** also exhibits the peaks corresponding to the loss of H_2_O, HCN, and CO molecules (see the SI). Both *E*/Z-oxime configurations are observed in **2a** (*E*/*Z* = 15:1 in DMSO-*d*_6_), even though the HIA precursor **1a** is only present as an *E*-oxime isomer. Oxime isomerization during and after the photoreaction will be discussed in the pertinent section of the paper.

Whereas in Moorthy′s work, the competition between Norrish II and Norrish–Yang reactions is independent of the polarity of the solvent,^15,16,20,21,32^ in the case of HIAs, fragmentation and cyclization occur alternatively. In fact, when apolar solvents were employed, such as DCM or benzene, decomposition of the starting material was observed yielding a complex mixture of products (Table 1, entries 5–6). Conversely, by carrying out the reaction in polar solvents like MeOH-*d*_4_ and ACN-*d*_3_, the corresponding CBO **2a** was isolated as a single product with complete conversion and improved diastereoselectivity of 2.5:1 in favor of the *trans* isomer (Table 1, entries 3–4). While the diastereomeric *ratio* (dr) values were comparable, the reaction carried out in MeOH-*d*_4_ was faster, with complete conversion in 1.5 h compared to the 5 h in ACN-*d*_3_ (for details see the SI).

Consequently, we selected DMSO-*d*_6_ and MeOH-*d*_4_ as the best solvents to proceed toward the evaluation of the general applicability of the reaction on variously substituted HIAs **1b–o** (Scheme 3). On the one hand, monitoring of the reaction course is easier in DMSO, owing to better resolution of diastereoisomeric peaks and to the presence of reliable −OH signals. On the other hand, the use of MeOH could lead to higher diastereoselectivity and allows easier isolation of the synthesized CBOs by simple solvent evaporation. Indeed, the products were fully characterized to confirm an excellent degree of purity without any further purification. A further synthetic advantage is provided by functionalization of the oxime–OH group (**1k–o**), which resulted in stability of the obtained CBOs on silica, thus enabling also the isolation of all diastereoisomers *via* flash column chromatography, achieving good to excellent product recovery (up to 95% yield). It is worth noting that in CD_3_OD, variable amounts of the hemiacetal form of **1k–o** were observed, thanks to the distinct position of the >C=NO*CH*_3_ and >C=NO*CH*_2_-Ph signals (3.8 and 5.05 ppm, respectively). However, the carbonyl/hemiacetal equilibrium did not affect either the product yield or reaction times.

In all of the tested cases, excellent conversion was observed in the formation of the desired CBOs (Scheme 3). Both aliphatic and aromatic R^1^ substituents were well tolerated in terms of conversion to CBO. It is worth mentioning that the functionalization of the oxime moiety allowed for the purification of the products *via* column chromatography with good to excellent yields and, in the case of products **2k**, **2l**, and **2m**, all of the generated diastereoisomers were successfully isolated and characterized separately (see the SI). Most importantly, the presence of different functional groups such as esters and double bonds was well tolerated in the radical cyclization. In fact, compound **2f** was isolated with complete conversion and no detectable side products.

Finally, to demonstrate the applicability of the procedure, a scale-up experiment (1.1 mmol) was carried out with substrate **1o**, isolating the corresponding CBO **2o** with comparable yield (91%) and the same excellent diastereoselectivity (>99:1 dr), further validating the synthetic value of this methodology.

### Spectroscopic and Stereochemical Analyses of Cyclobutanol Oximes

**Scheme 4:**
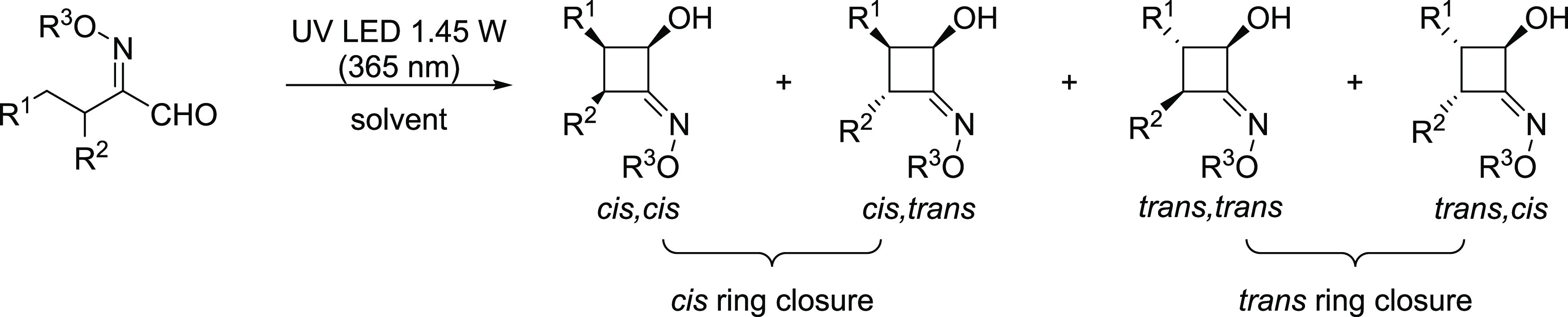
Possible Stereoisomers Derived from a *trans* or *cis* Ring Closure Only one enantiomer is reported for each diastereoisomer.

As shown in Scheme 3, all C3-substituted HIAs gave the corresponding cyclobutanol oximes with excellent stereocontrol and, being the CBO moiety a confined small ring with many functional groups in the proximal vicinity, the spectroscopic determination of the relative configuration of the obtained compounds was a key challenge. In fact, in the case of products **2a–f** and **2k–l** the possible diastereomers are derived only from a *cis* or *trans* ring closure (between OH and R^1^), while the introduction of an additional substituent in compounds **2g–j** and **2m–o** implicates the generation of a third stereocenter during the photoisomerization so that up to eight stereoisomers can be formed. Several factors (solvent polarity, hydrogen bond, stereoelectronic requirements, substituent steric hindrance) can potentially play a role in determining the *cis* or *trans* ring closure and, consequentially, the stereoselectivity of the reaction (Scheme 4).

**Figure 1:**
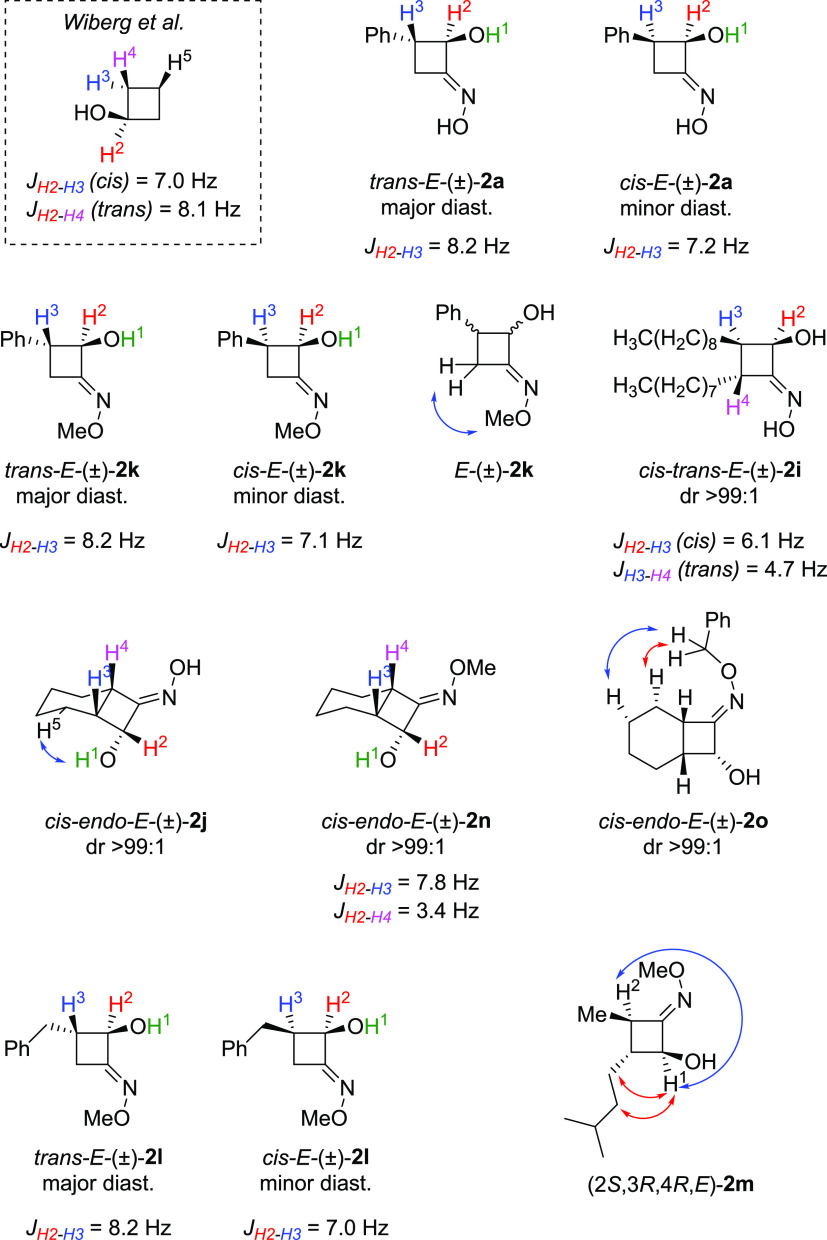
Determination of the relative configuration of the obtained CBO *via* decoupling ^1^H NMR and NOESY NMR experiments.

The assignment of *cis/trans* ring signals was determined by a combination of spin-decoupled ^1^H, ^13^C, HSQC, and NOESY NMR spectroscopy (see the Supporting Information). In the case of products **2a**–**j** and **2k–l**, the values of the *J* couplings between protons H^2^ and H^3^ in both major and minor diastereoisomers were in good agreement with literature data on substituted cyclobutanols,^46^ hence identifying the major diastereoisomer as the *trans*-CBO (Figure 1). Similarly, the relative configuration of products **2j** and **2-n–o** was assigned mainly on the basis of *J* coupling values, confirming the formation of the *endo* product for the CBOs **2e,i,j**. Furthermore, the NOESY NMR (see the SI, Figure S3) showed a weak contact between the alcoholic H^1^ and the alkylic H^5^, which is also compatible with the most probable distances in the *endo* structure (see SI, Figures S10 and S11).

Additionally, NOESY NMR on the methylated CBO **2k** exhibited a clear correlation between the methyl group and CH_2_, allowing us to assign the configuration of the oxime double bond as *E* in both isomers. In compound **2o**, the benzylic CH_2_ group showed NOE contact with two cyclohexane protons, confirming the *E* configuration of the oxime double bond.

Finally, all analyzed CBOs gave the same CID fragmentation pattern as that of **2a**, both in negative and positive ion modes.

Once the NMR diagnostic signals were assigned, we were able to evaluate the diastereoselectivity of the ring closure as a function of substitution and solvent. We first focused our attention on substrates with R^2^ = H, which would afford the corresponding CBOs bearing only two stereogenic centers (Scheme 3, **2a–f**). The diastereoselectivity was limited in these cases, with dr in the 1.1:1–1.7:1 range (*trans*:*cis*), both in DMSO and MeOH. Subsequently, HIAs having R^2^ ≠ H were tested, to evaluate the influence of the introduction of a third incipient chiral center on the reaction outcome (Scheme 3, **2g–j**). Interestingly, the reactions carried out in DMSO-*d*_6_ showed better diastereoselectivity in favor of a *cis* closure of the cyclobutane ring.^32^ Indeed, in DMSO, we observed the formation of a single diastereoisomer of cyclobutanol oximes **2h**, **2i**, and **2j** (Scheme 3). It should be noted that, in the case of HIA **1i**, H-abstraction can occur at two equivalent Cγ-positions, R^1^ and R^2^ being just slightly different in length (product **2i** in Scheme 3). However, the two resulting regioisomers are not distinguishable by either NMR or MS analysis. It appears that the presence of an additional substituent (R^2^ ≠ H) causes the inversion of the ring closure stereoselectivity from *trans* to *cis* (products **2h–j**). Interestingly, in the case of product **2g** (R^2^ = Me), the ring closure still favors the *trans* diastereoisomer. Presumably, the smaller steric hindrance of the Me group (**2g**) compared to Pr (**2h**), is not sufficient to invert the diastereoselection.

*O*-functionalized HIAs showed a similar behavior; in the case of methylated compounds **1k–l** (R^2^ = H), the diastereoselectivity (dr up to 1.8:1) decreased in comparison with the corresponding nonmethylated compound **1a** (dr = 2.5:1), whereas excellent stereoselectivity was observed for both methylated **1n** (R^2^ ≠ H) and benzylated **1o** (R^2^ ≠ H), affording the same level of diastereocontrol in the *cis* ring closure (>99:1 dr) (Scheme 3).

Considering that HIA **1g** was prepared starting from (±)-citronellol, we envisioned that the preparation of both optically pure HIAs derived by (*R*)- or (*S*)-citronellol, both commercially available, could give us crucial information on the stereochemical features of the process. Indeed, we prepared HIAa (*S*)-**1g** and (*R*)-**1g** and the corresponding methylated one (*R*)-**1m**. The obtained substrates were probed under photoirradiation conditions and the results were analyzed by both NMR and HPLC on a chiral stationary phase, in comparison with the corresponding racemic analogues.

**Scheme 5:**
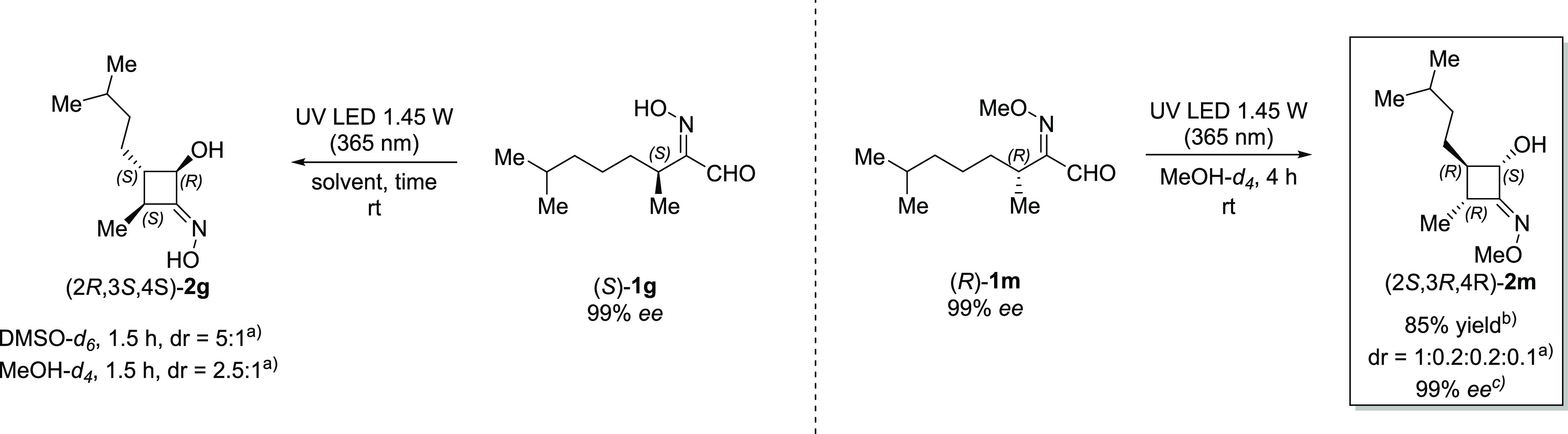
Photoinduced Norrish−Yang Cyclization Optically Pure HIAs (*R*)-**1g**, (*S*)-**1g**, and the Corresponding Methylated One (*R*)-**1m** [a] dr values corresponding to the *trans*:*cis* ring-closure products determined via ^1^H NMR. The second diastereoisomer possesses an absolute configuration of 2S,3S,4S (cis ring closure); [b] isolated yield after flash column chromatography; [c] determined via HPLC analysis on a chiral stationary phase.

The product (4*S*)-**2g** was obtained with excellent conversion and a diastereomeric ratio of 5:1 (Scheme 5). The same level of diastereocontrol was observed on the racemic product (±)-**2g** (Scheme 3), highlighting that the presence of a defined chiral center does not influence the diastereoselectivity of the transformation. Similar results were observed with the methylated product (4*R*)-**2m**. Additionally, for this compound, all of the diastereoisomers were purified by column chromatography and characterized separately. The purification was performed as well on the racemic product (±)-**2m** and the major diastereoisomers were analyzed by HPLC on a chiral stationary phase, showing complete retention of the optical purity with 99% *ee* values.

These outcomes showed that no racemization process occurs during the photochemical isomerization reaction, which generates the two new stereocenters in a complete enantioselective process. Moreover, it allowed us to unambiguously assign the absolute configuration of the major diastereoisomer of the obtained optically pure CBOs as (2*S*,3*R*,4*R*,*E*)-**2m**, on the basis of the known configuration of C4 combined with the relative configuration determined *via* NMR analyses.

### IR Multiple Photodissociation Spectroscopy

**Figure 2:**
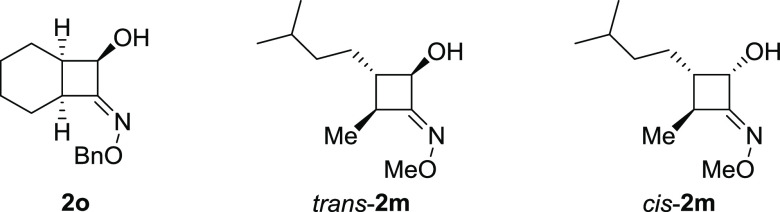
Rlative configuration of CBO analyzed by IRMPD.

The stereochemical assignments were further validated by relying on IR multiple photon dissociation (IRMPD) spectroscopy. Using this technique, it is possible to obtain the IR spectra of ions, which are mass-selected and trapped in the cell of a mass spectrometer, by plotting the abundancies of the fragments that are produced by the interaction of the ions with resonant photons as a function of the photon energy.^47,48^ IRMPD spectroscopy has proven to be a valuable tool for the characterization of reaction intermediates,^49−52^ and it has also shown potential for the discrimination of stereoisomers.^53−56^ It is thus a promising approach for the unambiguous identification of the herein-presented compounds. Compounds **2o**, *cis-***2m**, and *trans-***2m** (Figure 2) were characterized as bare protonated species. Photofragmentation mass spectra are reported in Figures S19–21 in the SI. Main fragmentation channels are also described and agree with the structural features of the ions. Interestingly, [*cis-***2m**+H]^+^ and [*trans-***2m**+H]^+^ show some differences in the fragmentation channels, in particular, the loss of a water molecule is only present in the dissociation pathway of [*cis-***2m** +H]^+^.

**Figure 3:**
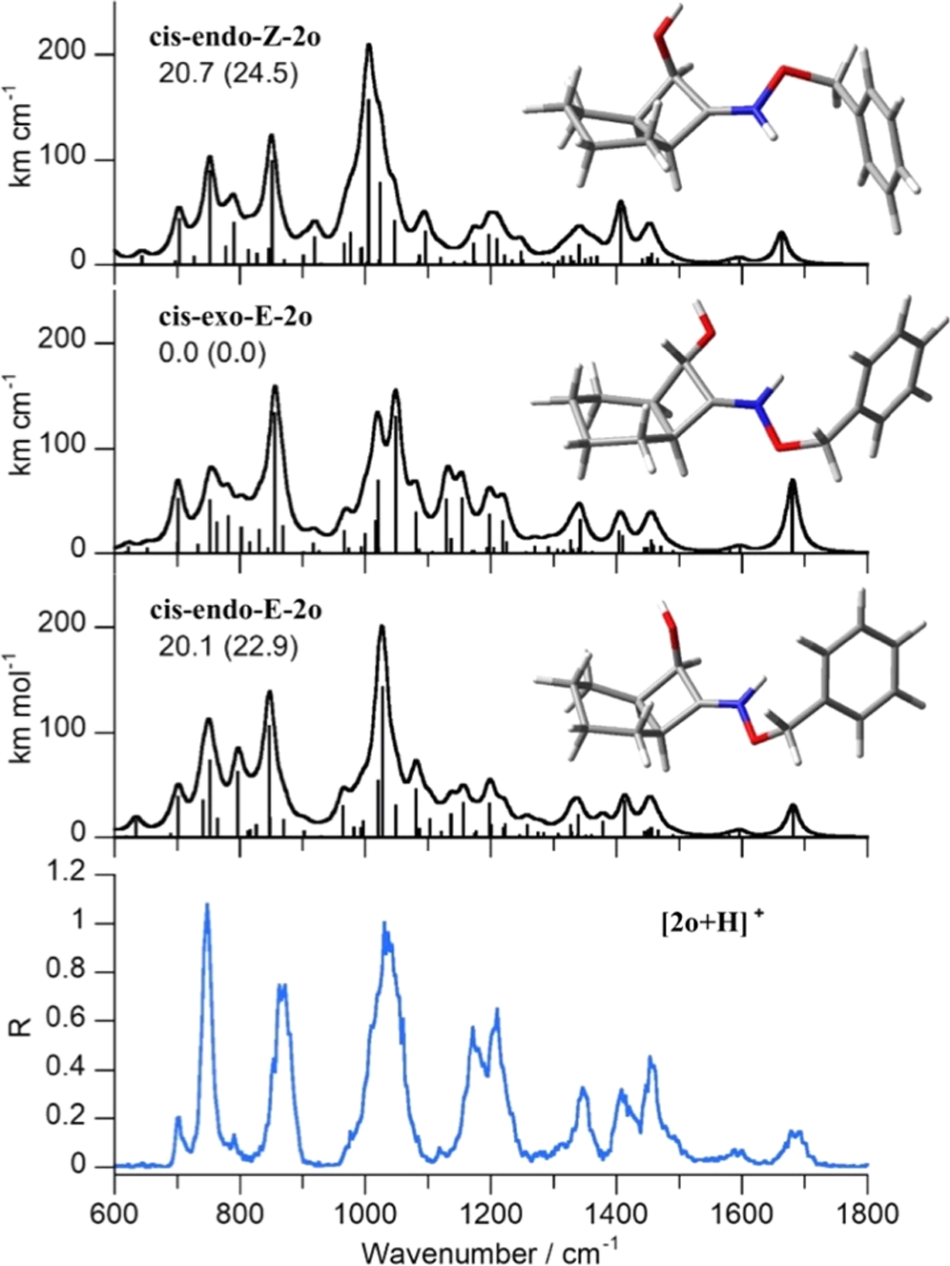
IRMPD spectrum of [**2o** +H]^+^, compared with calculated IR spectra for *cis-endo-E-***2o**, *cis-exo-E-***2o**, and *cis-endo-Z-***2o**. Optimized geometries are reported together with relative free energies (enthalpies) at 298 K in kJ mol^–1^.

Figure 3 reports the IRMPD spectrum of [**2o**+H]^+^ together with the calculated IR spectrum of the optimized structure designed to have the hypothesized stereoisomerism, *cis-endo-E-***2o**. For comparison purposes, the geometries of the *exo* stereoisomer, *cis-exo-E-***2o**, and the Z isomer *cis-exo-E-***2o** were also optimized and the corresponding spectra are reported. Protonation of the alcohol group for *cis-endo-E-***2o** was also tested, but the optimization process led to the N-protonated form. The experimental spectrum shows the best agreement with the calculated one of *cis-endo-E-***2o**, confirming the assigned stereoselectivity of the synthetic procedure. It has to be noted that the calculated isomers are not interconvertible, thus relative energies cannot be used to predict the actual isomer population. Vibrational modes assignment is reported in Table S1 in the SI. Indeed, the calculated vibrations of the stereoisomers *cis-exo-E-***2o** and *cis-endo-Z-***2o** do not significantly differ from the ones of *cis-endo-E-***2o**. However, it is possible to highlight a few characteristic features. Regarding *cis-exo-E-***2o**, the most evident difference is the presence of a partially split band comprising at 1049 cm^–1^ the NO stretching coupled with the OH bending and at 1021 cm^–1^ the NO bend and CC stretch. This is in contrast with the single broad IRMPD band at 1033 cm^–1^ that is better interpreted by the same couple of vibrational modes calculated for *cis-endo-E-***2o** at 1029 and 1022 cm^–1^, respectively. Also, the two experimental bands at 1213 and 1173 cm^–1^ are better reproduced by the cluster of calculated vibrational modes of *cis-endo-Z-***2o** in that spectral range. The spectrum of the protonated Z isomer of compound **2o**, *cis-endo-Z-***2o**, albeit showing a somehow good agreement with the experiment, presents a slight shift in the frequency of the CN stretching mode with respect to the corresponding IRMPD band: values are 1664 and 1683 cm^–1^, respectively. Indeed, the experimental frequency is better reproduced by *cis-endo-E-***2o** with its calculated value of 1682 cm^–1^. The pronounced signal at 1033 cm^–1^ is also not well simulated by the NO stretching calculated for *cis-endo-Z-***2o** at 1006 cm^–1^, while the cluster of vibrational modes around 1200 cm^–1^ again does not accurately reproduce the experiment. In summary, the comparison allows the assessment of *cis-endo-E-***2o** as the sampled isomeric form of [**2o**+H]^+^.

To confirm the opposite stereoselectivity of the *trans* ring closure in the presence of the Me group as R^2^ in product **2m** (*vide supra*), the isolated pure diastereoisomers *cis*-**2m** and *trans*-**2m** were also characterized using IRMPD spectroscopy, and the experiments interpreted by DFT calculations. The results are reported in Figure S18. Both IRMPD spectra of [*cis*-**2m**+H]^+^ and [*trans*-**2m**+H]^+^ are in good agreement with the calculated spectra of the corresponding E isomers protonated on the nitrogen atom: *cis*-*E*-**2m** and *trans-E*-**2m**, respectively. The vibrational mode assignment for both species is reported in Tables S2 and S3. Compounds *cis*-**2m** and *trans*-**2m** differ in the configuration of one of their stereocenters, in particular the C2 atom. Indeed, both their IRMPD spectra present comparable vibrational features as highlighted in Tables S2 and S3; however, the spectral range below 800 cm^–1^ shows some differences which can be correlated to the different stereochemistry of compounds *cis*-**2m** and *trans*-**2m**. Calculated vibrations of *cis-E*-**2m** in that range show the presence of several modes of similar intensity related to the NH bend coupled with other vibrational modes, which is in agreement with the broadening of the lower portion of the IRMPD signal at 757 cm^–1^ and the emerging of a smaller feature at 690 cm^–1^. On the contrary, the IRMPD spectrum of [*trans*-**2m**+H]^+^ presents a single signal at 750 cm^–1^ consistent with the presence in the calculated *trans-E*-**2m** spectrum of a dominant signal at 755 cm^–1^, i.e., the out-of-plane NH bending. Protonation on the alcoholic functionality was also taken into account for both [*cis*-**2m**+H]^+^ and [*trans*-**2m**+H]^+^ leading to the structures *cis-E*-**2m**(-O^+^H_2_) and *trans-E*-**2m**(-O^+^H_2_), respectively. Their presence in the sampled population can indeed be discarded on the basis of the evident disagreement of computed spectra with experimental ones. Finally, the calculated spectra of the Z isomers of *cis*-**2m** and *trans*-**2m**, *cis-Z*-**2m** and *trans-Z*-**2m**, respectively, are reported and compared to the IRMPD spectra to assess their absence in the sampled gas-phase population and confirm the samples to be single diastereoisomers. Though simulating most of the vibrational bands, the calculated spectra of *cis-Z*-**2m** and *trans-Z*-**2m** show poor agreement with the experiment. It should be noted that the theoretical CN stretching modes (1648 cm^–1^ for both *cis-Z*-**2m** and *trans-Z*-**2m**) are blue-shifted with respect to the corresponding IRMPD bands at 1673 cm^–1^ in a similar fashion as already observed for [**2o**+H]^+^. These results confirm the spectroscopic assignment of the relative configuration of the major diastereoisomer as *trans*-**2g/m**, opposite to products **2h-j**.

### Mechanistic Insight through DFT Calculations

Once having unambiguously assigned the relative configuration of the final products and having studied the dependence of the reaction outcomes on the HIA structure, we focused on the postulation of a reaction mechanism on the basis of DFT calculations, which could also rationalize the diastereoselectivity observed.

**Figure 4:**
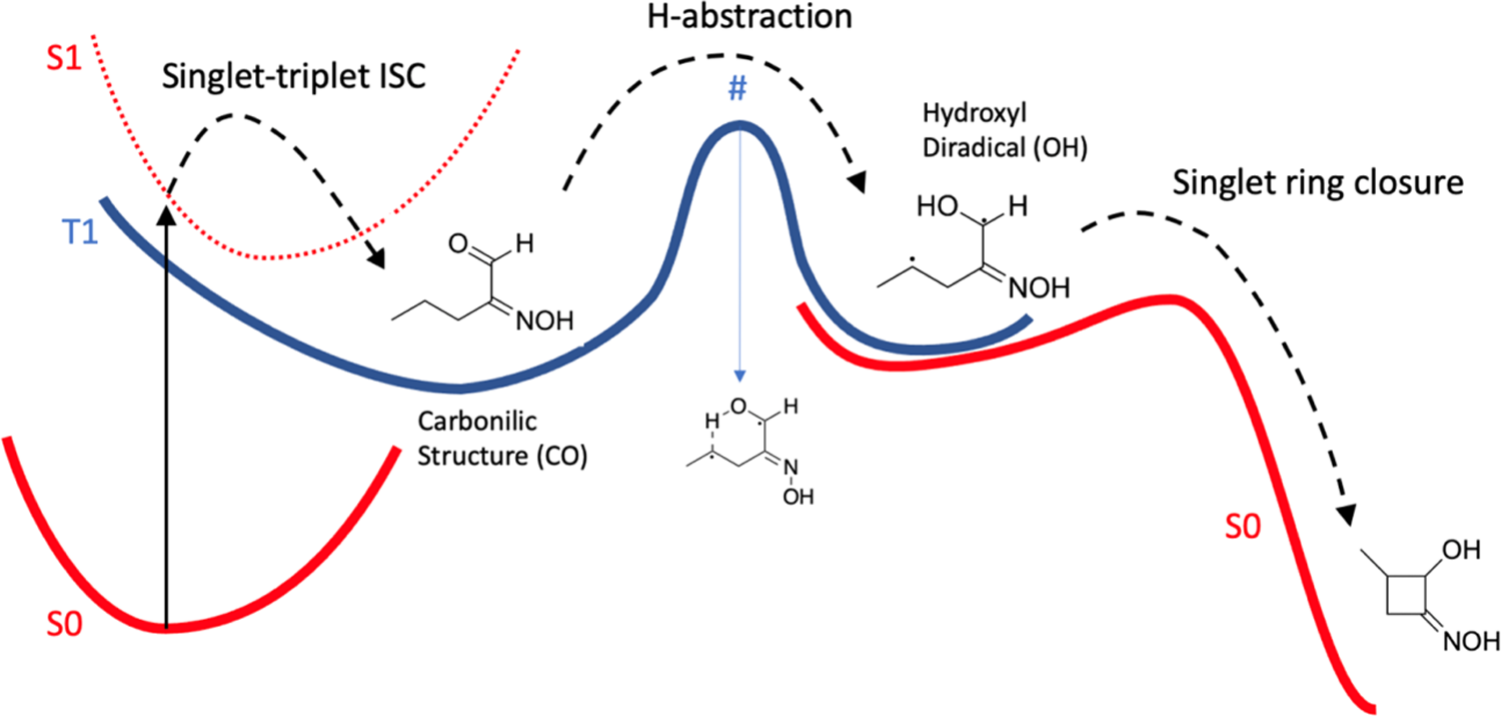
Simplified scheme illustrating the proposed reaction mechanism for the HIA model compound **1p**. The potential energy curves in blue and red are drawn only for illustrative purposes. See text for details.

The proposed reaction mechanism follows the one presented in ref (34) and is illustrated qualitatively in Figure 4. The blue line corresponds to the T_1_ triplet PES. The red ones are for singlet states. The initial photoexcitation brings the molecule to the S_1_ state.^57^ This state in compounds like **1p** and **1j** absorbs at 310 nm (∼4 eV above S_0_) and is dominated by a ^1^(n, π*) excitation. The second excited state S_2_, dominated by a ^1^(π, π*) excitation, is located at 5.4–5.5 eV above S0 (∼230 nm). Hence, the only state that can be pumped by the LED irradiation is S_1_. While we cannot completely exclude that the ^1^(π, π*) state could play a role, we verified that, at least in the very first stage of the mechanism, its energy remains around 2 eV above that of S_1_, therefore, in the following, we assume that it does not partake in the reaction. An ISC to the T_1_ triplet state ^3^(n, π*) follows. We note that this nonradiative S_1_ ⇝ T_1_ transition is a nonefficient process activated by vibronic couplings.^58^ The triplet PES has a first minimum where the molecule is in the carbonylic form. The latter undergoes unimolecular H-abstraction to form a diradical hydroxyl. This step is hindered by an activation barrier of around 20 kcal/mol and the transition state is characterized by a cyclic six-membered ring.

As also noted in ref (34), in the diradical hydroxyl intermediate, the distance between the two unpaired electron sites is large enough for the two spin arrangements, triplet (↑↑) and singlet (↑↓) to have essentially the same energy.

The final step in the reaction path consists of a ring closure in the singlet state due to the pairing of the two radical sites. The ring closure brings the two diradicals into the final cyclic products. The ring closure does not seem to be characterized by a downhill energetic path toward the final products. We have found that this step is hindered by a barrier. The different stereochemistry is essentially due to the relative position of the OH group before closure.

**Figure 5:**
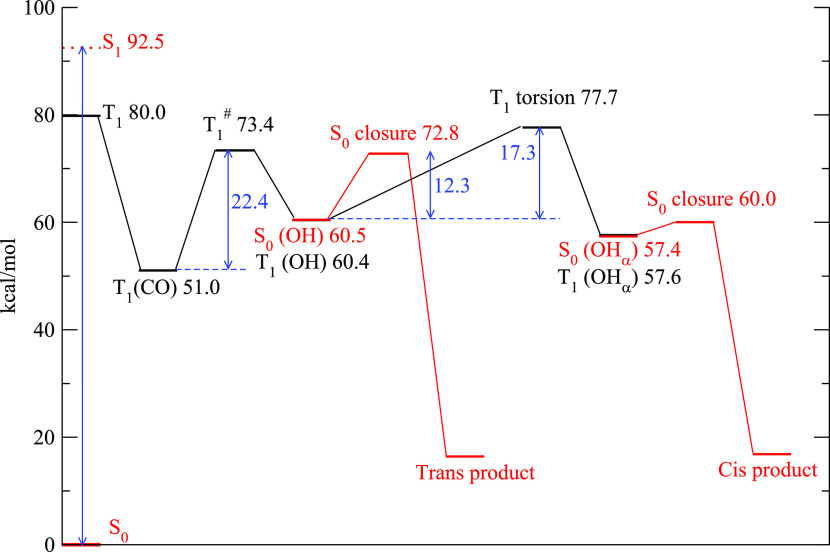
Energetic profile of the reaction of compound HIA **1p**. The triplet stationary points are drawn in black and the singlet ones in red. The numbers represent the energy differences with the ground state S_0_ in its minimum geometry and are expressed in kcal/mol.

The quantitative version of the above scheme for the model compound HIA **1p** is shown in Figure 5. Model compound **1p** was chosen as representative for HIAs with R^2^ = H to simplify DFT calculations. The reactive process starts on the left where the photoabsorption brings the reactant to the S_1_ state (located approximately 90 kcal/mol above the ground state). The vertical energy of the corresponding triplet is 80 kcal/mol. These numbers are compatible with those reported in ref (34). The minimal structure of the triplet state in its carbonylic form, **T**_**1**_**(CO)**, is located 51.0 kcal/mol above S_0_ and is reported in Figure S12a. Its structure is characterized by a noncoplanar geometry of the oxime group with respect to the carbonyl with the unpaired spins residing mainly on the C=N bond and only slightly on the two oxygen atoms (purple isosurface in Figure S12).

The triplet can undergo unimolecular H-abstraction to form the hydroxyl structure. The barrier (**T**_**1**_^**#**^) to the reactive process is 22.4 kcal/mol and leads to the hydroxyl diradical intermediate **T**_**1**_**(OH)**. The latter structure is shown in Figure S12b. The oxime group, in this structure, is coplanar with the hydroxyl and the unpaired spins are mainly delocalized over the carbon and nitrogen. This minimum is less stable than **T**_**1**_**(CO)** by about 9 kcal/mol.

In the diradical, owing to the localization and the distance of the unpaired spin, the energy of the triplet state is almost degenerate with the singlet (Δ*E* = 0.01 kcal/mol). The local minimum geometry of the singlet **S**_**0**_**(OH)** is also very similar to that of **T**_**1**_**(OH)** and is reported in Figure S12c.

At this point, the **S**_**0**_**(OH)** molecule can undergo a ring closure and transform into the final product CBO **2p** with a *trans* ring closure. By tracing a minimum energy path of the closure, we estimated a barrier of around 12–13 kcal/mol. Some of the sampled geometries along the path are shown in Figure S13a.

If the conversion from the triplet **T**_**1**_**(OH)** to the singlet **S**_**0**_**(OH)** is not fast, the former can undergo a rotation around the C–CHOH bond to produce the conformer **T**_**1**_**(OH**_**α**_**)** and its corresponding singlet state **S**_**0**_**(OH**_**α**_**)** whose structure is reported in Figure S13d. The transformation of **T**_**1**_**(OH)** into **T**_**1**_**(OH**_**α**_**)** is hindered by a considerable rotational barrier of 17.3 kcal/mol. The ring closure from **S**_**0**_**(OH**_**α**_**)** requires only 2–3 kcal/mol and leads to the *cis* product through the path illustrated in Figure S13b.

**Figure 6:**
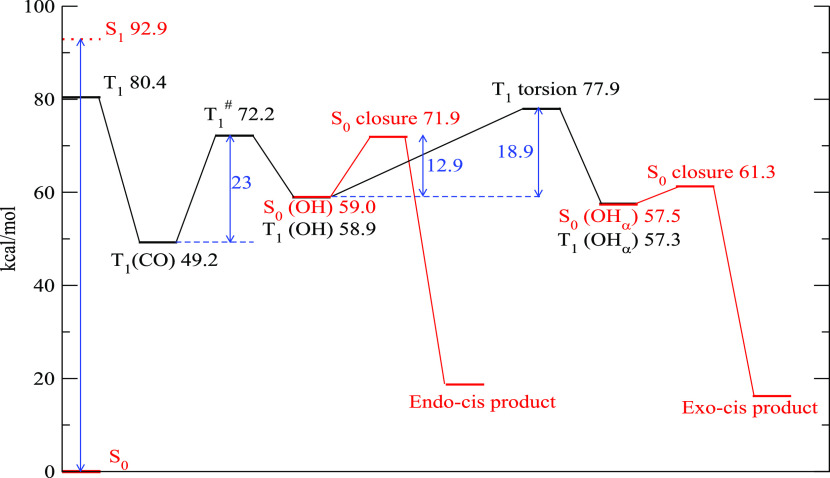
Energetic profile of the reaction of compound **1j**. The triplet stationary points are drawn in black and the singlet ones in red. The numbers represent the energy differences with the ground state S_0_ in its minimum geometry and are expressed in kcal/mol.

The data pertaining to compound HIA **1j** are presented in an analogous fashion in Figure 6, where we see that the general reactive scheme closely follows the one illustrated for HIA **1p**.

The H-abstraction in **T**_**1**_**(CO)** can take place either toward the equatorial or axial position of the target CH_2_ and we have localized either transition states. The equatorial abstraction (Figure S14a) produces a conformation that invariably leads to a ring closure toward a *trans* configuration. The axial abstraction (Figure S14b) leads to the observed *cis* final product. The barrier of axial H-abstraction is 23 kcal/mol, similar to the one found for HIA **1j**, while that of the equatorial is higher (28.3 kcal/mol). Thus, in Figure 6, for clarity, we have reported only the lowest energy transition state that is also the one that leads to the observed final products.

Once **T**_**1**_**(OH)** is formed, the molecules can undergo the same processes we have illustrated for HIA **1p**. The ring closure either produces the *endo-cis* or *exo-cis* stereochemistry depending on the position of the OH group in the initial singlet state before the formation of the C–C bond. The closure initiated in the **S**_**0**_**(OH**) geometry leads to the observed *endo-cis* product, while a closure initiated in the **S**_**0**_**(OH**_**α**_**)** leads to the *exo-cis* product (see Figure S15). The **S**_**0**_**(OH)** and **S**_**0**_**(OH**_**α**_**)** interconvert through a torsion around the C—CHOH bond that is hindered by an energetic barrier of almost 19 kcal/mol.

It is evident from the data presented in Figures 5 and 6 that the possibility of the **T**_**1**_ state to evolve particular stereochemistry is due to the competition between the rotational barrier and the closure one. If the rotational barrier is much lower than the latter, the reaction will not be fully stereoselective and both closure processes are possible. The outcome, in this case, is more likely to be determined by the relative height of the two barriers along the closure barriers in the diradical singlet states **S**_**0**_**(OH)** and **S**_**0**_**(OH**_**α**_**)**. Otherwise, if the rotational barrier is higher than that of the closure ones, the final product can only be the one arising from the **T**_**1**_**(OH)** geometry because the two conformers **T**_**1**_**(OH)** and **T**_**1**_**(OH**_**α**_**)** cannot interconvert rapidly enough.

In both molecules analyzed here, the energy barrier to the torsional motion in **T**_**1**_**(OH)** turns out to be higher than the one for the singlet ring closure, thereby preventing the T_1_ state to access the **T**_**1**_**(OH**_**α**_**)** conformer (Figure 5). In both **1j** and **1p**, we should therefore expect an almost complete stereoselectivity.^34^ This is consistent with our experimental results for compound **1j**, which forms exclusively the *endo-cis* product. In contrast, when testing HIAs with R^2^ = H, to which **1p** is the model compound, no ring closure stereoselectivity is observed. This effect may be ascribed to differences in the solvent coordination of **1j** and **1p** biradicals, which are not taken into account by the *in vacuo* calculations and are impossible to include even with continuum (implicit) solvent models. Coordination of the less sterically demanding **1p** biradical may lower its torsional barrier, resulting in decreased stereoselectivity.

As mentioned in the introduction, in addition to the diastereoselectivity of the Norrish–Yang ring closure, it is important to consider the possibility for the hydroxyl diradical (OH) to undergo Norrish II fragmentation (Scheme 1). In a Norrish-type II reaction pattern, not only must the two radical carbon centers be in a *transoid* conformation,^21,32^ but also the C2-C3 σ bond must be parallel to the *p* radical orbitals, to ensure both bond cleavage and the formation of the double bond in the resulting fragment (Scheme 1). In our system, stereoelectronic and steric features, due to the presence of the oxime group, might prevent the correct parallel alignment in the *transoid* conformation. In fact, in our DFT calculations we have computed the energies of the singlet states of the hydroxyl diradical (OH) and determined the optimal geometries at the nearest local minima, which turn out to be in a *cisoid* conformation (Figure S12c), thus favoring the Norrish–Yang cyclization pathway over fragmentation.

### Solvent-Dependent *E*/*Z* Isomerization of the Oxime Double Bond

**Figure 7:**
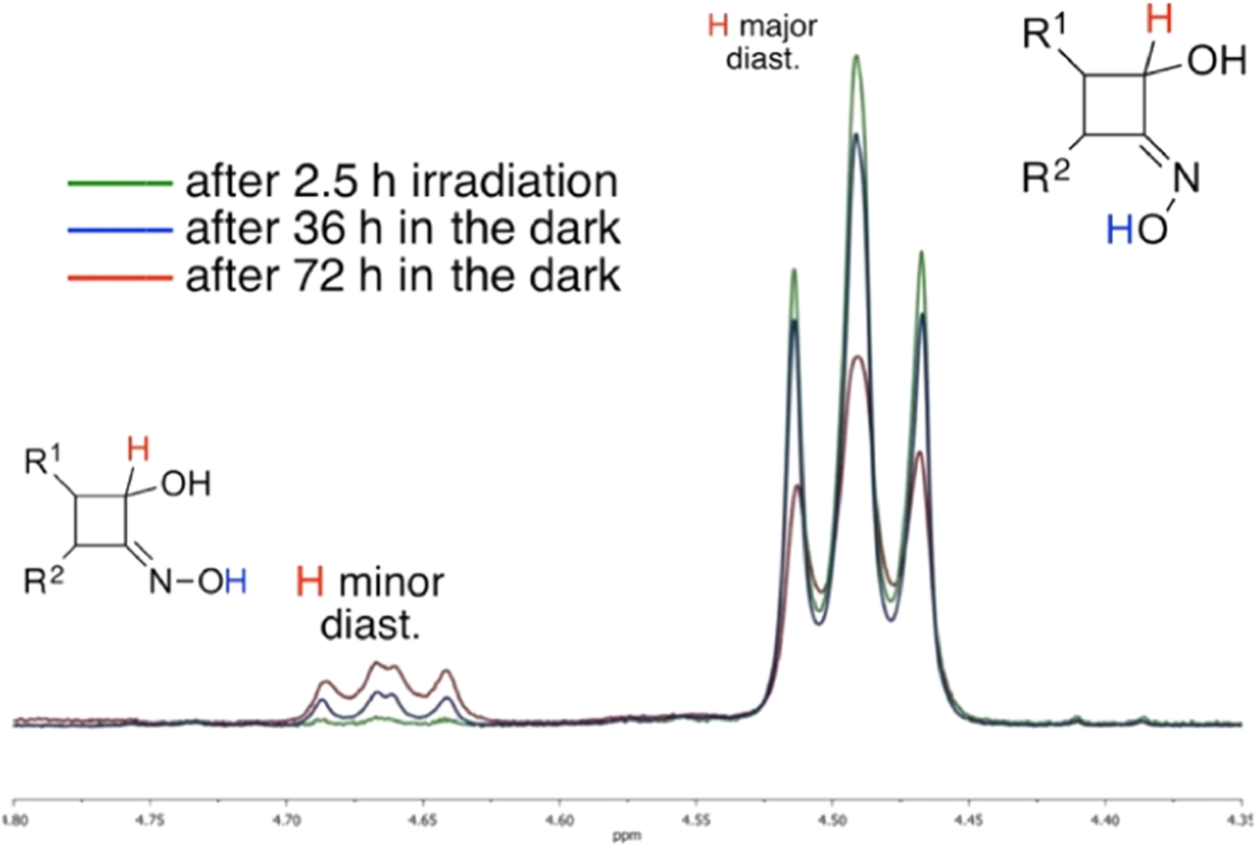
Expansion of ^1^H NMR spectra of **2j** (see the Supporting Information) at different times of storing in the dark at room temperature after the photoisomerization depicting diagnostic peaks of H^2^ as an example. Diagnostic CBO protons are shown in different colors.

Finally, our attention was drawn to the *E/Z* isomerism of the oxime group. All substrates exhibit >95% *E* configuration prior to photoisomerization, regardless of the solvent, whereas different *E*/*Z* ratios (ranging from 99:1 to 1.4:1) in the CBO products are initially obtained in a solvent-dependent manner. This matter is thereby described specifically with substrate **1j**. The corresponding CBO **2j** is initially obtained in DMSO-*d*_6_ as a single diastereomer. However, to our surprise, a second set of **2j** CBO signals appeared over time upon standing in DMSO-*d*_6_ at the expense of those of the original diastereomer, reaching a final ratio of 5:1 within 72 h after irradiation (see the SI for the final ^1^H NMR spectrum). For clarity, Figure 7 shows the evolution over time of the cyclobutane H^2^ signal. It should be noted that both isomers exhibit the *cis* ring closure. In CD_3_OD, instead, the same two sets of signals appear already during the photoreaction in a 2.5:1 ratio. This ratio remained unchanged for several days upon storage in CD_3_OD. The outcome in CD_3_CN and acetone-*d*_6_ was the same as in methanol, i.e., the initial isomer ratio did not change over time.

However, when CD_3_OD was removed and the samples were redissolved in DMSO-*d*_6_, the NMR signals changed over time to finally reach the same 5:1 ratio as in the sample obtained directly in DMSO-*d*_6_. We ascribe the new set of CBO signals to DMSO-assisted *E*/Z isomerization of the oxime C=N bond. In fact, DMSO-assisted double bond configurational isomerizations have been reported for α-oxo-oximes,^59^ hydrazones,^60^ and azolylmethylidene 2-indanone derivatives.^61^ In all cases, the isomerizations were ascribed to the H-bonding between DMSO and the C=N−OH of the isomerizing double bond, with no need for photoactivation or acid catalysis. Specifically, noncatalyzed oxime isomerization can occur by rotation of the substituents around the C=N bond through a polar transition state, sensitive to solvent polarity.^62^ Strong H-bonding between the labile oxime O–H hydroxyl group and DMSO, combined with the bulk of the two alkyl substituents on the already tense four-membered ring structure in products **2j**, could affect the C=N character, thus assisting the *E*/Z isomerization. (see Scheme S1 in the Supporting Information). The same solvent-promoted *E*/*Z* CBO isomerization is observed in **2g–i** (Scheme 3), whereas *O*-functionalized HIAs do not exhibit this behavior, thus proving the crucial role of the oxime-OH group in this isomerization. Hence, to explore the role of the interaction between the oxime OH and DMSO or methanol, we relied on molecular dynamics calculations.

**Figure 8:**
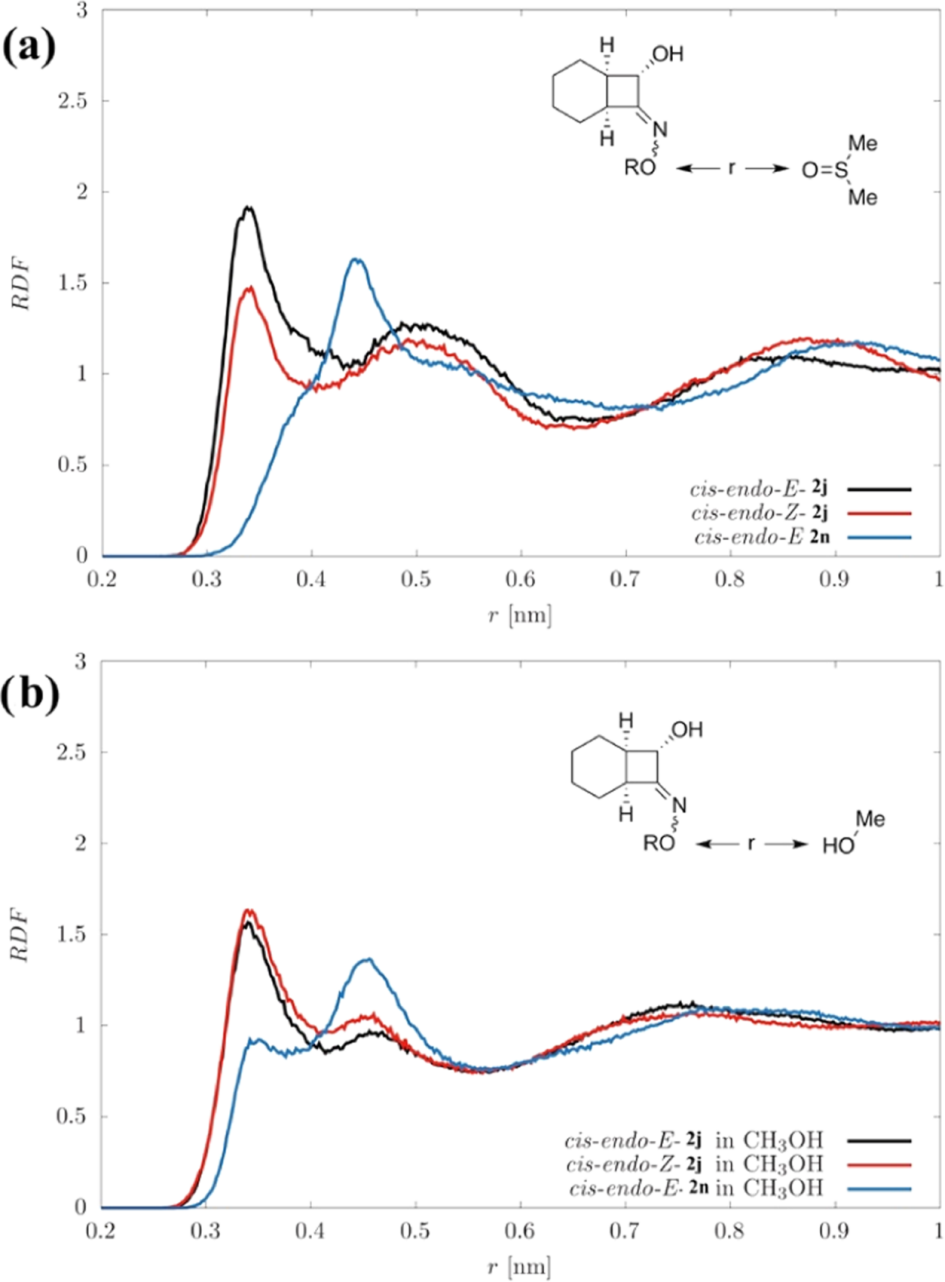
Radial distribution functions related to the interaction between CBO oxime oxygen and DMSO (a) and oxygen and MeOH (b), R = H (**2j**) e R = CH_3_ (**2n**).

Molecular dynamics simulations were performed via the GROMACS (v. 2021.2) software package^63^ on the major diastereomer (*cis-endo*) of free oxime **2j** and **2n**, the corresponding oxime methyl ether. Details about simulations can be found in the SI. In Figure 8, radial distribution functions (RDFs) of DMSO and MeOH oxygen with respect to the oxime oxygen are shown to highlight the solvent-oxime interaction. An analogous curve is reported for reference in Figure S7 for **2j** and DCM, representative of a nonhydrogen bonding solvent. Interestingly, the curves of weakly interacting systems (i.e., **2n**-DMSO and **2j**-DCM) exhibit similar features. On the other hand, the **2j**-DMSO and **2j**-MeOH (Figure 8a,b, respectively) curves exhibit a peak at *r* = 0.34 nm, which is absent in Figure S7, thus pointing at a strong oxime-solvent interaction in both configurational isomers of **2j**. However, the peak of (*E*)-**2j-**DMSO is higher than that of (*Z*)-**2j-**DMSO, whereas no such difference is present with MeOH. Furthermore, the interactions of the CBO alcoholic oxygen with DMSO of both isomers are very similar to that of the oxime OH in (*Z*)-**2j** (Figure S8). In other words, whereas the *Z*-oxime interacts similarly with both solvents, DMSO forms a stronger hydrogen bonding with the (*E*)-**2j** oxime, thus weakening the CNO–H bond and promoting *E*/*Z* C = NOH isomerization.

## Conclusions

In conclusion, we provide a unique and straightforward strategy for the synthesis of cyclobutanol oximes *via* Norrish–Yang photocyclization of 2-(hydroxyimino)aldehydes and their ethers. The reaction is virtually free from competing fragmentation processes, which are usually predominant in the case of aldehydes, and is diastereoselective in many cases. Interestingly, we demonstrate that alkylating the oxime group in the starting HIAs makes it possible to isolate the cyclobutanol oxime diastereoisomers in good to excellent yields (up to 95%), which adds synthetic significance to this work. Reaction conditions are convenient, in that cyclization is efficiently obtained under LED irradiation, with excellent conversion in short reaction times and in the absence of a photosensitizer. The stereochemical features of this isomerization are elucidated, studying the solvent and substituents’ effects on the diastereoselectivity, including the solvent-dependent *E*/*Z* isomerization of the oxime double bond. The latter is investigated through a series of control experiments combined with molecular dynamics calculations confirming that DMSO promotes postcyclization isomerization through a configuration-dependent strong interaction of the oxime-OH group with the solvent. Consistently, postcyclization *E*/*Z* isomerization is prevented through alkylation of the oxime group. DFT calculations *in vacuo* helped us elucidate some mechanistic features of this Norrish–Yang photocyclization and provide a rationale for the observed high stereoselectivity of a sterically demanding substrate, whereas solvent effects should be taken into account with less hindered 2-(hydroxyimino)aldehydes.

## Experimental Section

### General Information

All analytical and technical grade solvents were used as received. All commercially available reagents were used as received. Column chromatography was carried out using *Merck* silica gel (230–400 mesh, 40–63 μm particle size, 60 Å pore size) or on activated neutral alumina (Brockmann I, 40–160 μm particle size, 58 Å pore size). Thin-layer chromatography (TLC) was performed using glass plates precoated with a 0.25 mm thickness of silica gel and fluorescent indicator and visualized with UV light (254 nm), KMnO_4_ solution, or 2,4-dinitrophenylhydrazine solution. ^1^H NMR and ^13^C-NMR spectra were recorded in DMSO-*d*_6_ (vials, *VWR*), CDCl_3_ (bottle, *Aldrich*), or CD_3_OD (vials, *Eurisotop*) on a *Bruker* Avance300 spectrometer (300 MHz). Chemical shifts are reported in ppm relative to the resonance of DMSO-*d*_6_ (δ = 2.50), CDCl_3_ (δ = 7.26), or CD_3_OD (δ = 3.31) for ^1^H NMR and to the central peak of DMSO-*d*_6_ (δ = 39.5), CDCl_3_ (δ = 77.1), or CD_3_OD (δ = 49.0) for ^13^C-NMR. The multiplicity is abbreviated as follows: s (singlet), d (doublet), t (triplet), q (quartet), m (multiplet), and bs (broad singlet). The coupling constant *J* is given in Hz (Hertz). Photostimulations were performed in a 5 mm diameter clear fused quartz *Wilmad* Precision NMR sample tube. GC–MS analyses have been run on an HP 5892 series II GC, equipped with a 5% phenyl silicone 30 m × 0.25 mm × 25 μm capillary column and coupled to an HP 5972 MSD instrument operating at 70 eV. High-resolution mass spectra (HRMS) were obtained with a Bruker BioApex Fourier transform ion cyclotron resonance (FT-ICR) mass spectrometer. Collision-induced dissociation (CID) was carried out using ESI LTQ-XL (Thermo Scientific) at different collision energies. The photochemical setup is described in detail in the Supporting Information.

### General Procedure for the Photoinduced Norrish–Yang Cyclization

The 2-(hydroxyimino)aldehyde or alkylated 2-(hydroxyimino)aldehyde (0.060–0.075 mmol, 1.0 equiv) is dissolved in the appropriate deuterated solvent (600–750 μL, C = 0.1 M) and placed in an NMR quartz tube. The sample is placed in a photoreactor (see section **1.1** of the Supporting Information) and irradiated with a 365 nm LED light source while thermostated at 25 °C with a cooling fan. Reaction completion is monitored with ^1^H NMR until the disappearance of the aldehydic proton of the 2-(hydroxyimino)aldehyde.

### IR Multiple Photodissociation Spectroscopy

#### IR Ion Spectroscopy Experiments

Infrared multiple photon dissociation (IRMPD) spectra were obtained at the Free Electron Laser for Infrared eXperiments (FELIX) facility (Nijmegen, The Netherlands) using a commercial 3D quadrupole ion trap mass spectrometer (Bruker amaZon speed ETD), modified to allow the trapped ions to interact with the FEL light.^64^ Samples were prepared with solubilizing compounds **2o**, *cis-***2m**, and *trans-***2m** in methanol and diluting them to a final concentration of 10^–5^ M. Subsequently, solutions were directly infused at a 120 μL h^–1^ rate and ionized using an ESI source operated in positive ion mode. Protonated ions were mass-selected and irradiated with a single IR pulse from the IR free electron laser, which was operated at 10 Hz with an energy of 70–100 mJ per pulse. The assayed frequency range was 600–1800 cm^–1^ with steps of 3 cm^–1^ in which four replicate mass spectra were averaged. Attenuators were employed when needed to reduce the laser power and avoid signal saturation. IRMPD spectra were collected by plotting the photofragmentation yield R (*R* = −ln[*I*_P_/(*I*_P_ + ∑*I*_F_)], where *I*_P_ and *I*_F_ are the abundances of the parent ion and of a fragment ion, respectively) as a function of the photon frequency.^65^ Yield was finally linearly corrected for the frequency-dependent variation in laser pulse energy.^66^

#### Computational Methods

Conformers of the different stereoisomers of [**2o**+H]^+^, [*cis-***2m** +H]^+^, and [*trans-***2m**+H]^+^ were sampled using the Conformer Distribution tool, as implemented in the software suite Spartan′16,^67^ and the semiempirical method PM6. Selected geometries were optimized at the B3LYP/6-311+G(2df,pd) level using Gaussian 09 rev. D.01^68^ Harmonic vibrational frequencies were computed at the same level of theory, thus obtaining IR spectra and thermodynamic corrections to the electronic energies. Harmonic frequencies were scaled by 0.974 on the basis of a good agreement with the IRMPD spectra.^69,70^ Calculated linear IR spectra have been convoluted with a Lorentzian profile of 12 cm^–1^ (fwhm).

### Molecular Dynamics Methods

The simulation box consists of a single oxime molecule surrounded by 163 solvent molecules. The different oxime isomers were considered here: *cis-endo-E*-**2j**, *cis-endo-Z*-**2j**, *cis-exo-E*-**2j**, *cis-exo-E*-**2j** as well as *cis-endo-E*-**2n** CBOs. Simulations are carried out using the leap-frog algorithm with a time step of 0.5 fs. A UFF force field is adopted for the purpose.^71^ Long-range electrostatic interactions are considered using the PME method while a cut-off scheme with a cut-off distance of 1 nm is applied for van der Waals interactions. Initial configuration minimization is carried out using the conjugate gradient method; further MD simulations lasting 90 ns were performed for each system to guarantee equilibrium. Canonical ensembles (NVT) are obtained through a V-rescale thermostat with the coupling constant τ_T_ = 0.1 ps and reference temperature 298.15 K. Periodic boundary conditions were applied in the three spatial directions. Data were collected during 30 000 000 step-lasting simulations (20 000 000 in the case of DCM).

### DFT Calculations

All calculations have been carried out using the Orca code (version 5).^72^ The first set of calculations has been done using density functional theory (DFT) and specifically ωB97X-D3BJ^73,74^ with the Def2-TZVP^75^ basis set using the RIJCOSX approximation.^76^ The ωB97X family of separated range functionals has shown decent performances in describing diradicals of organic molecules.^77^ To control the reliability of the results, all calculations have also been repeated using the M06-2X functional^78^ with the cc-pVTZ basis set^79^ using the RI approximation.^80^ In the main text, we shall report only the ωB97X-D3BJ calculations and the M06-2X ones can be found in the SI. The energy of the diradical singlet has been obtained using DFT and the broken symmetry (BS) formalism,^81^ i.e., by calculating the triplet UKS solution and flipping the spin on one of the two atoms where the unpaired spin resides. We are aware of the intrinsic limits of this approach and of the problems due to the strong spin contamination in the broken symmetry singlet solutions, but the size of the systems and the number of calculations did not allow for a better treatment via multiconfigurational methods. The calculations have been carried out using the following procedure. Initially, the singlet ground state optimal geometries and energies have been calculated. The vertical triplet state energies have then been evaluated, and the corresponding triplet geometries relaxed to the nearest minima on the triplet potential energy surface. At this point, the triplet molecule can undergo a hydrogen abstraction from the CH_2_ on the side chain to yield the diradicals reported in Scheme 1 on the right. The transition state for the abstraction process has been calculated in the triplet multiplicity and fully characterized by an Intrinsic Reaction Coordinate (IRC) calculation to ensure its uniqueness. Since the stereochemistry of the final product is determined by the relative position of the OH group, the energetic barrier along a torsion of the RNC–^•^CHOH bond has also been computed in the triplet state. Within the BS approach, we have computed the energies of the singlet states of the two diradical molecules, and we have determined the optimal geometries at the nearest local minima that turn out to be essentially the same as the triplet ones. The final step consists in calculating the minimum energy path to the ring closure due to the pairing of the two radical sites in the singlet state. The ring closure brings the two diradicals into the final cyclic products. The closure paths have been traced using a relaxed optimization scan over the C^•^–C^•^ distance built using 16 points between 3.5 and 1.5 Å. All reported data are electronic energies. While the stationary points of the triplet PES have been characterized (when possible) using a frequency calculation, thermodynamic functions have not been reported for various reasons. First, consistency: some of the computed points have been found using constrained optimizations for which it would not be possible to evaluate frequencies. Second, thermodynamics would rely on the harmonic approximation, which is known to have limits. In addition, the thermal energy components would have been calculated using ideal gas approximate formulae, thus introducing an additional level of approximation. Finally, since the entire reaction involves only one molecule, therefore it is safe to assume that entropic effects are only limited.

## Supporting Information Available

Materials and methods; synthetic procedures; ^1^H, ^13^C{^1^H}, and 2D NMR spectra of all compounds; ESI-HRMS; molecular dynamics and DFT calculations; CID and IRMPD spectra; and HPLC chromatograms (PDF)
