# Supplementary material for: Unusually Chemoselective Photocyclization of 2-(Hydroxyimino)aldehydes to Cyclobutanol Oximes: Synthetic, Stereochemical, and Mechanistic Aspects
Source: J Org Chem. 2022 Oct 5;87(21):13803–18. doi: 10.1021/acs.joc.2c01503 (PMC9639046; doi:10.1021/acs.joc.2c01503)
Supplement: Supplementary file 1 — jo2c01503_si_001.pdf [file jo2c01503_si_001.pdf]

Supporting information

The unusually chemoselective photocyclization of 2-(hydroxyimino)aldehydes to cyclobutanol oximes: Synthetic, stereochemical and mechanistic aspects

Antonio Di Sabato,[1,2] Francesca D'Acunzo,[2] Dario Filippini,[1] Fabrizio Vetica[1,*] Antonio Brasiello,[3] Davide Corinti,[4] Enrico Bodo,[1] Cinzia Michenzi,[1] Edoardo Panzetta,[1] Patrizia Gentili[1,2,*]

[1]Department of Chemistry, Sapienza University of Rome, Piazzale Aldo Moro 5, 00185 Rome, Italy
[2]Institute of Biological Systems (ISB), Italian National Research Council (CNR), Sezione Meccanismi di Reazione, c/o Department of Chemistry, Sapienza University of Rome, Piazzale Aldo Moro 5, 00185 Rome, Italy
[3]Department of Chemical Engineering Materials Environment, Sapienza University of Rome, via Eudossiana 18, 00184 Rome, Italy
[4]Dipartimento di Chimica e Tecnologie del Farmaco, Sapienza University of Rome, Piazzale Aldo Moro 5, 00185 Rome, Italy

email: fabrizio.vetica@uniroma1.it, patrizia.gentili@uniroma1.it

Contents

1. Materials and Methods 3
 1.1. Photochemical setup 3
2. Synthetic procedures 4
 2.1. General procedure for aldehydes synthesis via Albright-Onodera oxidation[1] 4
 2.2. General procedure for aldehydes synthesis via Parikh-Doering oxidation[4] 4
 2.3. Synthesis of 3-propylhexanal 5
 2.4. Synthesis of 3-octyltridecanal 6
 2.5. General procedure for α-oximation of aldehydes[12] 7
 2.6. General procedure for Cu catalyzed azide-alkyne Huisgen cycloaddition 9
 2.7. General procedure for methylation of 2-(hydroxyimino)aldehydes 9
 2.8. Synthesis of 2-((benzyloxy)imino)-2-cyclohexylethanal 10
 2.9. General procedure for the photocyclization of 2-(hydroxyimino)aldehydes 10
3. Copy of NMR spectra of new compounds 15
 3.1 NOESY contacts relevant to the determination of the relative configuration of CBOs 2j, 2n and 2o. . 57
4. CBO C=N double bond *E/Z* isomerization 60
5. Molecular dynamics 61
 5.1 Methods 61
 5.2 Discussion 61
6. CID spectra 65
7. DFT Calculations 67
8. IRMPD 69
9. HPLC trace of compound (4R)-2m 73
10. References 73

1. Materials and Methods

All analytical and technical grade solvents were used as received. All commercially available reagents were used as received. Column chromatography was carried out using *Merck* silica gel (230-400 mesh, 40-63 µm particle size, 60 Å pore size) or on activated neutral alumina (Brockmann I, 40-160 µm particle size, 58 Å pore size). Thin layer chromatography (TLC) was performed using glass plates precoated with a 0.25 mm thickness of silica gel and fluorescent indicator and visualized with UV light (254 nm), $KMnO_4$ solution or 2,4-Dinitrophenylhydrazine solution. ^{1}H-NMR and ^{13}C-NMR spectra were recorded in DMSO-d_6 (vials, *VWR*), $CDCl_3$ (bottle, *Aldrich*) or CD_3OD (vials, *Eurisotop*) on a *Bruker* Avance300 spectrometer (300 MHz). Chemical shifts are reported in ppm relative to the resonance of DMSO-d_6 (δ = 2.50), $CDCl_3$ (δ = 7.26) or CD_3OD (δ = 3.31) for ^{1}H-NMR and to the central peak of DMSO-d_6 (δ = 39.5) , $CDCl_3$ (δ = 77.1) or CD_3OD (δ = 49.0) for ^{13}C-NMR. The multiplicity is abbreviated as follows: s (singlet), d (doublet), t (triplet), q (quartet), m (multiplet), bs (broad singlet). The coupling constant *J* is given in Hz (Hertz). Photostimulations were performed in 5 mm diameter clear fused quartz *Wilmad* Precision NMR sample tube. GC-MS analyses have been run on a HP 5892 series II GC, equipped with a 5% phenyl silicone 30m × 0.25mm × 25µm capillary column and coupled to a HP 5972 MSD instrument operating at 70 eV. High-resolution mass spectra (HRMS) were obtained with Bruker BioApex Fourier transform ion cyclotron resonance (FT-ICR) mass spectrometer. Collision induced dissociation (CID) were carried out by using ESI LTQ-XL (Thermo Scientific) at different collision energies

1.1. Photochemical setup

The photoreactor for NMR-scale experiments consists of a lab-made LED chip assembly equipped with a sample holder. (Figure S1). The LED assembly is obtained by gluing a single LED chip (Nichia NVSU233A-D1 UV SMD-LED with PCB (10x10mm), λ = 365nm, 1030mW radiant flux or Nichia NVSU233B SMD-LED UV with PCB (10x10mm), λ = 365nm, 1450mW radiant flux, 3.75 V forward voltage from LUMITRONIX® LED-Technik GmbH) on an aluminum heat sink, using Fischer Elektronik heat-conducting adhesive WLK DK 4. The LED is powered with a constant current power supply (Meanwell LCM-40 Series, LUMITRONIX® LED-Technik GmbH). The sample holder consists of an aluminum block with a 7 mm housing to accommodate a 5mm NMR tube. The sample holder is screwed on top of the LED holder for easy replacement of the LED chip. The NMR tubing is placed in direct contact with the silicon lens that covers the light source and ensures a 60° viewing angle. Care should be taken not to damage the silicon lens. The system is kept at room temperature with the aid of a cooling fan.

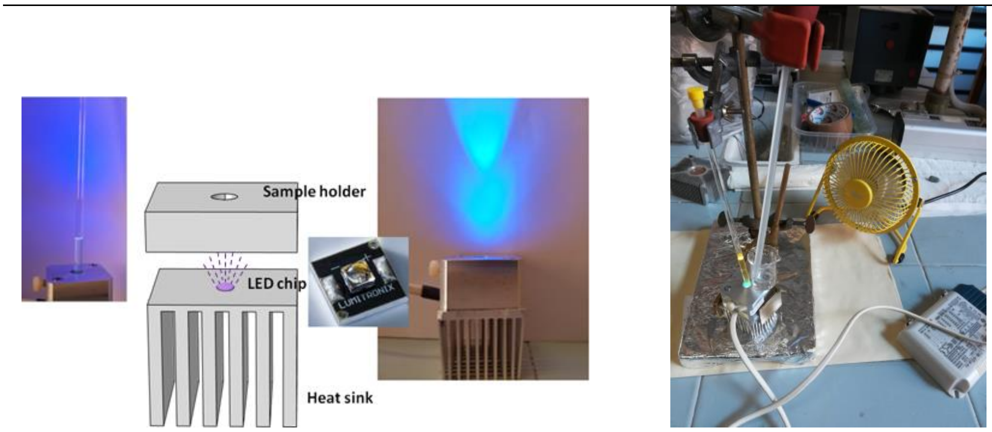

Figure S1. LED assembly for the photostimulation of samples for NMR studies. Schematic is not drawn to scale.

2. Synthetic procedures

2.1. General procedure for aldehydes synthesis via Albright-Onodera oxidation[1]

To a solution of the alcohol (4.0 mmol, 1.0 equiv) in CH_2Cl_2 (10 mL) and DMSO (8.0 mmol, 2 equiv), P_2O_5 (7.2 mmol, 1.8 equiv) is added slowly to avoid overheating. The resulting slurry is stirred at room temperature for 1 h then cooled to 0 °C with an ice bath, then Et_3N (14.0 mmol, 3.5 equiv) is added dropwise. The resulting clear solution is stirred at room temperature for about 30 min (reaction completion is monitored via GC-MS analysis), then quenched with cold (4 °C) 1 M HCl (15 mL). The organic layer is separated, then washed with H_2O (1 x 15 mL) and brine (3 x 15 mL) and dried over Na_2SO_4. The solvent is removed by evaporation at reduced pressure and the residue is purified through column chromatography to afford the aldehydes as colorless liquids.

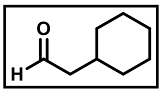

2-cyclohexylethanal. Reaction performed on 500 mg (3.91 mmol) of 2-cyclohexylethan-1-ol. Flash chromatography was performed with a mixture of CH_2Cl_2/Et_2O as eluant in ratio 50:1. Colorless liquid, 380 mg (3.02 mmol), 77 % yield.
^{1}H-NMR (300 MHz, $CDCl_3$) δ 9.75 (t, *J* = 2.4 Hz, 1H), 2.29 (dd, *J* = 6.8, 2.4 Hz, 2H), 1.98 – 1.80 (m, 1H), 1.76 – 1.62 (m, 5H), 1.37 – 0.91 (m, 5H). Data are in agreement to those reported in the literature[1].

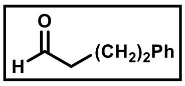

4-phenylbutanal. Reaction performed on 500 mg (3.33 mmol) of 4-phenylbutan-1-ol. Flash chromatography was performed with a mixture of petroleum ether/Et_2O in gradient from 10:1 to 8:1. Colorless liquid, 383 mg (2.59 mmol), 78 % yield.
^{1}H-NMR (300 MHz, $CDCl_3$) δ 9.76 (t, *J* = 1.6 Hz, 1H), 7.39 – 7.10 (m, 5H), 2.67 (t, *J* = 7.5 Hz, 2H), 2.46 (td, *J* = 7.3, 1.6 Hz, 2H), 2.02 – 1.92 (m, 2H). Data are in agreement to those reported in the literature[2].

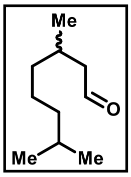

3,7-dimethyloctanal. Reaction performed on 500 mg (3.16 mmol) of 3,7-dimethyloctan-1-ol. The aqueous phase was extracted with 20 mL of Et_2O given the low solubility of the product in CH_2Cl_2. Flash chromatography was performed with a mixture of petroleum ether/Et_2O as eluant in ratio 20:1. Light yellow oil, 460 mg (2.94 mmol), 93 % yield.
^{1}H NMR (300 MHz, $CDCl_3$) δ 9.76 (t, *J* = 2.4 Hz, 1H), 2.40 (ddd, *J* = 16.0, 5.8, 2.1 Hz, 1H), 2.22 (ddd, *J* = 15.9, 7.8, 2.6 Hz, 1H), 2.12 – 1.98 (m, 1H), 1.56 – 1.48 (m, 1H), 1.34 – 1.11 (m, 6H), 0.96 (d, *J* = 6.7 Hz, 3H), 0.86 (d, *J* = 6.7, 1.5 Hz, 6H). Data are in agreement to those reported in the literature[4].

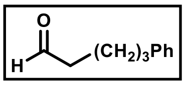

5-phenylpentanal. Reaction performed on 1000 mg (6.10 mmol) of 5-phenylpentan-1-ol. Flash chromatography was performed with a mixture of pentane/Et_2O in gradient from 30:1 to 15:1. Colorless oil, 834 mg (5.15 mmol), 83 % yield
^{1}H NMR (300 MHz, $CDCl_3$) δ 9.76 (t, *J* = 1.7 Hz, 1H), 7.38 – 7.10 (m, 5H), 2.66-2.64 (m, 2H), 2.54 – 2.40 (m, 2H), 1.68 (m, 4H). Data are in agreement to those reported in the literature[4].

2.2. General procedure for aldehydes synthesis via Parikh-Doering oxidation[4]

1.0 mmol of alcohol (1.0 equiv), 7 mL CH_2Cl_2 and 0.54 mL (4.0 mmol, 4.0 equiv) Et_3N are introduced in a flame-dried two-necked round bottomed flask equipped with a nitrogen inlet and magnetic stirring. The flask is chilled in an ice bath, then a solution of 0.48 g (3.0 mmol, 3.0 equiv) SO_3·Pyridine complex in 3 mL DMSO is added. Stirring is continued at 0°C for 1 h, then for 3 h at room temperature. The following workup procedure is aimed at minimizing product loss and maximizing removal of DMSO from the reaction crude. The reaction mixture is diluted with CH_2Cl_2 or Et_2O and washed with a 1:1 (v/v) mixture of NH_4Cl (sat) and brine. Most of the CH_2Cl_2 (if used) is removed by rotary evaporation and the residue is taken up with Et_2O. The aqueous phase is extracted with fresh Et_2O to minimize product loss. The combined organic phases are washed again

with the NH_4Cl/ NaCl (sat), then with brine alone to remove as much DMSO as possible. After drying over anhydrous Na_2SO_4, the solvent is removed and the residue is purified by silica gel chromatography.

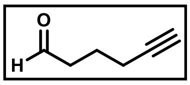

5-hexynal. Reaction performed on 1.80 g (18.4 mmol) of 5-hexyn-1-ol. Flash chromatography was performed with a mixture of petroleum ether/Et_2O in gradient from 20:1 to 8:1. Whereas Et_2O was removed by distillation, the product could not be isolated from solvent hydrocarbons. After a first distillation through a Vigreux column, we held on to the product-rich residue and we submitted the richest distilled fraction to a second distillation in an attempt to further concentrate the product. We thus obtained two product-rich lightly yellow oily residues and the yield of 5-hexynal was estimated by ^{1}H-NMR analysis. The residual solvent was assumed to be equivalent to hexane (C_6H_{14}), so that the integral of all signals below 1.8 ppm are worth 14H. Based on the amounts of the two fractions, we estimate 940 mg (9.79 mmol; 54% yield) as 73% (w/w) in hexanes.
^{1}H-NMR (300 MHz, $CDCl_3$) δ 9.77 (t, *J* = 1.3 Hz, 1H); 2.58 (td, *J* = 7.2, 1.3 Hz, 2H); 2.24 (td, *J* = 6.8, 2.5 Hz, 2H); 1.96 (t, *J* = 2.5 Hz, 1H); 1.82 (m, 2H). Data are in agreement to those reported in the literature[6].

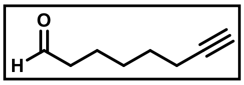

7-octynal. Reaction performed on 430 mg (3.41 mmol) of 7-octyn-1-ol. Flash chromatography was performed with a mixture of hexane/EtOAc in gradient from 15:1 to 12:1. Light yellow oil, 354 mg (2.85 mmol), 84 % yield.
^{1}H-NMR (300 MHz, $CDCl_3$) δ 9.77 (t, *J* = 7.3 Hz, 1H); 2.45 (td, *J* = 7.3, 1.7 Hz, 2H); 2.21 (td, *J* = 7.0, 2.7 Hz, 2H); 1.94 (t, *J* = 2.7 Hz, 1H); 1.66-1.48 (m, 6H). Data are in agreement to those reported in the literature[6].

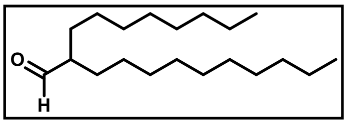

2-octyldodecanal. Reaction performed on 925 mg (3.10 mmol) of 2-octyldodecan-1-ol. Colorless liquid, 830 mg (2.80 mmol), 90 % yield.
^{1}H-NMR (300 MHz, $CDCl_3$) δ 9.54 (d, *J* = 3.18 Hz, 1H); 2.18-2.24 (m, 1H); 1.67 -1.57 (m, 2H), 1.25-1.45 (m, 30H); 0.87 (t, *J* = 6.42 Hz, 6H). Data are in agreement to those reported in the literature[7].

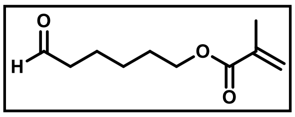

6-oxohexyl 2-methylprop-2-enoate. Under N_2 atmosphere, to an ice-cold solution of 1,6- hexanediol (2.00 g, 16.9 mmol) in anhydrous THF (20 mL) were added successively Et_3N (1.17 mL, 8.46 mmol), DMAP (0.206 mg, 1.69 mmol) , and methacrylic anhydride (1.26 mL, 8.46 mmol). The mixture was stirred at 0° C for 1 h. MeOH (2.7 mL) was added, and stirring was maintained for 10 minutes. After solvents evaporation under reduced pressure, the crude mixture was diluted with ethyl acetate (25 mL) and washed with HCl 1M, saturated $NaHCO_3$ and brine. The organic phase was dried over Na_2SO_4, filtered, and the solvents evaporated under reduced pressure. 1.052 g of a mixture of 6-hydroxyhexyl 2-methylprop-2-enoate and the dimethacrylate by-product was obtained and used in the next step without further purification. Under N_2 atmosphere, to the crude mixture obtained in dichloromethane (32 mL), was added Et_3N (2.7 mL, 19.6 mmol), followed, after cooling in an ice bath, by a solution of SO_3-Pyr complex (2.110 g, 13.7 mmol) in DMSO (13.3 mL). The mixture was stirred at room temperature for 24 h. The crude mixture was washed with NH_4Cl (sat) , $NaHCO_3$ (sat) and H_2O. The solvent was removed by rotary evaporation, and the mixture was re-dissolved with Et_2O (30 mL), then washed with brine and H_2O. The organic phase was dried over Na_2SO_4 , filtered, and the solvents evaporated. The residue was purified by silica gel chromatography with a hexane/ethyl acetate gradient to afford 835 mg of 6-oxohexyl 2-methylprop-2-enoate as a colorless liquid (4.54 mmol), 54 % overall yield.
^{1}H-NMR (300 MHz, $CDCl_3$) δ 9.7 (t, *J* = 1.6 Hz, 1H); 6.07 (m, 1H); 5.5 (m, 1H); 4.1 (t, *J* = 6.5 Hz, 2H); 2.4 (m, 2H); 1.9 (t, *J* = 1.2 Hz, 3H); 1.6 - 1.73 (m, 4H); 1.35 - 1.46 (m, 2H). Data are in agreement to those reported in the literature[10].

2.3. Synthesis of 3-propylhexanal

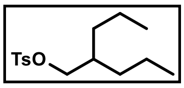

2-propylpentyl 4-methylbenzenesulfonate. 130 mg (1.0 mmol, 1.0 equiv) of 2-propyl-1-pentanol and 286 mg (1.5 mmol, 1.5 equiv) of *p*-toluensulfonyl chloride are dissolved in 2

mL of CH_2Cl_2. The resulting solution is cooled to 0 °C, then Et_3N (417 µL, 303 mg, 3.0 mmol, 3 equiv) is added dropwise and the reaction mixture is allowed to react at 25 °C. After 20 h the reaction is diluted with 13 mL of CH_2Cl_2 then the organic layer is washed with HCl 1 M (1 x 15 mL), water (1 x 10 mL), saturated aqueous $NaHCO_3$ (1 x 15 mL) and brine (1 x 15 mL), dried over Na_2SO_4 and the solvent removed by evaporation at reduced pressure. The title compound is obtained as a colorless oil (278 mg, 0.98 mmol, 98 % yield) and used without further purification.
^{1}H-NMR (300 MHz, $CDCl_3$) δ 7.78 (d, *J* = 8.3 Hz, 2H), 7.34 (d, *J* = 8.0 Hz, 2H), 3.90 (d, *J* = 5.4 Hz, 2H), 2.45 (s, 3H), 1.76 – 1.50 (m, 1H), 1.23 – 1.18 (m, 8H), 0.92 – 0.69 (m, 6H). Data are in agreement to those reported in the literature[8].

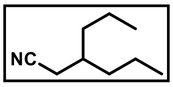

3-propylhexanenitrile. 2-propylpentyl 4-methylbenzenesulfonate (222 mg, 0.78 mmol, 1.0 equiv) is dissolved in 3 mL DMSO, then KCN (76 mg, 1.17 mmol, 1.5 equiv) is added and the reaction is warmed to 90 °C. After 3 h the reaction is cooled down to room temperature, diluted with 15 mL of saturated Na_2CO_3 and extracted 4 times with Et_2O. The combined organic layers are washed with brine, dried over Na_2SO_4 and evaporated at reduced pressure to afford 92 mg (0.66 mmol, 85 % yield) of 3-propylhexanenitrile as a colorless oil.
^{1}H NMR ($CDCl_3$, 300 MHz) δ 2.32 (d, *J* = 5.9 Hz, 2H), 1.74–1.68 (m,1H), 1.44–1.23 (m, 8H), 0.94–0.87 (m, 6H). Data are in agreement to those reported in the literature[9].

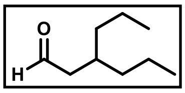

3-propylhexanal. In a flame-dried two-necked round bottom flask 92 mg (0.66 mmol, 1.0 equiv) of 3-propylhexanenitrile are dissolved in 11 mL of anhydrous hexane then a 1 M solution of diisobutylaluminum hydride in heptane (DIBAL-H, 1.66 mL, 2.5 equiv) is added under an Ar atmosphere. After 3 h, 9 mL of 96 % EtOH and 5.5 mL of H_2O are added dropwise to hydrolyze the excess of DIBAL-H and stirred for 1 h. The reaction mixture is then extracted with Et_2O (3 x 10 mL), the organic layers washed with 3 M HCl (1 x 20 mL), H_2O (1 x 20 mL), saturated $NaHCO_3$ (1 x 20 mL), brine (1 x 20 mL) and dried over Na_2SO_4. The product is purified via flash chromatography using as eluant a mixture of pentane/Et_2O in ratio 30:1. The product-containing fractions were distilled through a Vigreux column at ambient pressure to remove the solvents present, since evaporation with Rotavapor led to complete product loss. Although it was possible to remove all the Et_2O this way, still accompanied with partial evaporation of the product, hydrocarbons were still present in the distillation residue. Given the ^{1}H-NMR ratio of the multiplets at δ = 1.32 – 1.20 (comprising both 3-propylhexanal and hydrocarbon methylene hydrogens) and at δ = 0.92 – 0.86 (representative of the terminal methyl protons), the solvent still present in the sample was assumed to be heptane. Therefore the clear colorless liquid obtained is estimated to be a 37 % (w/w) solution of 3-propylhexanal in heptane (118 mg of solution, estimated 44 mg of 3-propylhexanal, 0.31 mmol, 47 % yield).
^{1}H NMR ($CDCl_3$, 300 MHz) δ 9.76 (t, *J* = 2.4 Hz, 1H), 2.33 (dd, *J* = 6.6, 2.4 Hz, 2H), 2.08 – 1.85 (m, 1H), 1.38 – 1.15 (m, 8H + 24H from heptane), 0.94 – 0.80 (m, 6H + 14H from heptane). Data are in agreement to those reported in the literature[10].

2.4. Synthesis of 3-octyltridecanal

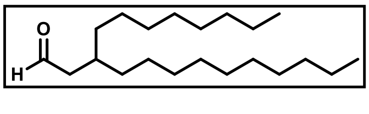

3-octyltridecanal. Under Ar atmosphere and at 0 °C, *t*-BuOK (0.94 g, 8.4 mmol) was added to a solution of $CH_3OCH_2PPh_3Cl$ (2.9 g, 8.3 mmol) in anhydrous THF (30 mL). The mixture was kept under magnetic stirring at room temperature for 4 h. 2-octyldodecanal (0.83 g, 2.8 mmol) dissolved in anhydrous THF (5 mL) was added at 0 °C and the mixture was kept under magnetic stirring at room temperature for 17 h. The mixture was filtered and the solvent evaporated under reduced pressure. The crude was dissolved in Et_2O (20 mL), washed with saturated NH_4Cl solution, dried over Na_2SO_4 and the solvent evaporated at reduced pressure. The residue was dissolved in THF (9 mL), then an aqueous HCl solution (1 M, 2.1 mL) was added and the mixture was stirred for 2 h at 75 °C. Et_2O (10 mL) was added and the mixture was washed with H_2O (5 mL). The aqueous phase was extracted with Et_2O (15 mL); the organic phases was washed successively with saturated $NaHCO_3$ and brine and dried over anhydrous Na_2SO_4. White solid, 0.35 g (1.1 mmol), 40 % yield.
^{1}H NMR ($CDCl_3$, 300 MHz) δ 9.76 (t, J = 2.4 Hz, 1H); 2.32 (dd, J = 6.6, 2.5 Hz, 2H); 1.94 (m, 1H); 1.37 (m, 32H); 0.88 (t, J = 6.5 Hz, 6H). Data are in agreement to those reported in the literature[10].

2.5. General procedure for α-oximation of aldehydes[12]

In a two-necked round bottom flask equipped with magnetic stirring are introduced in this order, under a N_2 atmosphere, 10 mL DMF, pyrrolidine (0.6 mmol, 0.2 equiv), *p*-toluenesulfonic acid (0.6 mmol, 0.2 equiv) and the aldehyde (3.0 mmol, 1.0 equiv). Then $NaNO_2$ (3.0 mmol, 1.0 equiv) is added, followed by $FeCl_3 \cdot 6H_2O$ (3.0 mmol, 1.0 equiv) in small portions to avoid excessive heating. The reaction is stirred for 4-6 h at room temperature (completion is monitored by TLC using hexane/ethyl acetate 5:1 as eluant). The mixture is diluted with 30 mL EtOAc and 20 mL of a 1:1 (v/v) mixture of NH_4Cl (sat) and brine and stirred for 20 minutes at room temperature. The organic layer is set aside and the aqueous phase is extracted with 20 mL fresh EtOAc to minimize product loss. The pooled organic solutions are washed once more with 20 mL of NH_4Cl (sat)/brine mixture, then three times with brine. After drying over anhydrous Na_2SO_4, EtOAc is removed by rotary evaporation. The product is purified by silica gel chromatography.

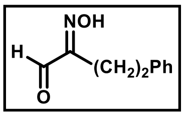

2-(hydroxyimino)-4-phenylbutanal (1a). Reaction performed on 350 mg (2.36 mmol) of 4-phenylbutanal. Flash chromatography was performed with a mixture of petroleum ether/Et_2O in gradient from 5:1 to 4:1. White to light yellow solid, 290 mg (1.64 mmol), 69 % yield, m.p. = 61-63 °C.

^{1}H-NMR (300 MHz, DMSO-*d*$_6$) δ 12.97 (s, 1H), 9.36 (s, 1H), 7.31 – 7.21 (m, 2H), 7.18 – 7.14 (m, 3H), 2.75 – 2.54 (m, 4H).

^{13}C{^{1}H}**-NMR** (75 MHz, DMSO-*d*$_6$) δ 191.6, 158.9, 140.8, 128.3, 128.1, 126.1, 30.6, 23.3.

ESI-HRMS (negative): calculated for $C_{10}H_{10}NO_2$ $[M-H]^-$, 176.0717; found 176.0720 ± 4.0 x10^{-4} u averaged from five measurements.

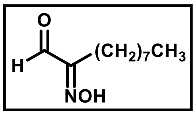

2-(hydroxyimino)decanal (1b). Reaction performed on 750 mg (4.81 mmol) of decanal. Flash chromatography was performed with a mixture of hexane/EtOAc in gradient from 30:1 to 8:1. White solid, 581 mg (3.14 mmol), 65 % yield, m.p. = 60-62 °C.

^{1}H-NMR (300 MHz, $CDCl_3$) δ 9.44 (s, 1H), 8.64 (bs, 1H) 2.59 – 2.34 (m, 2H), 1.53 – 1.39 (m, 2H), 1.26 (m, 10H), 0.87 (t, *J* = 6.7 Hz, 3H).

^{13}C{^{1}H}**-NMR** (75 MHz, $CDCl_3$) δ 191.3, 162.2, 32.2, 30.0, 29.5, 29.4, 25.8, 23.0, 22.2, 14.4. Data are in agreement to those reported in the literature[12].

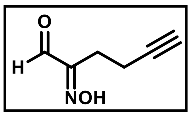

2-(hydroxyimino)-5-hexynal. Reaction performed on 940 mg (9.79 mmol) of 5-hexynal. Flash chromatography was performed with a mixture of hexane/Et_2O in gradient from 10:1 to 4:1. Yellow oil, 340 mg (2.72 mmol), 28 % yield.

^{1}H-NMR (300 MHz, DMSO-*d*$_6$) δ 13.06 (s, 1H); 9.40 (s, 1H); 2.76 (t, *J* = 7.6 Hz, 1H); 2.53 (t, *J* = 6.6 Hz 2H); 2.32 (td, *J* = 7.4, 2.6 Hz, 2H).

^{13}C{^{1}H}**-NMR** (75 MHz, DMSO-*d*$_6$) δ 190.9; 159.4; 82.6; 70.5; 69.4; 21.1; 14.7. Data are in agreement to those reported in the literature[13].

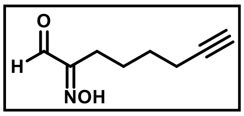

2-(hydroxyimino)-7-octynal. Reaction performed on 240 mg (1.94 mmol) of 7-octynal. Flash chromatography was performed with a mixture of hexane/EtOAc in gradient from 8:1 to 5:1. Yellow oil, 219 mg (1.43 mmol), 74 % yield.

^{1}H-NMR (300 MHz, $CDCl_3$) δ 9.46 (s, 1H); 8.66 (bs, 1H); 2.52 (t, *J* = 7.2 Hz, 2H); 2.20 (td, *J* = 7.0, 2.7 Hz, 2H); 1.94 (t, *J* = 2.7 Hz, 1H); 1.66 – 1.48 (m, 4H).

^{13}C{^{1}H}**-NMR** (75 MHz,$CDCl_3$) δ 191.0; 161.4; 84.1; 68.7; 28.4; 24.7; 21.4; 18.2. Data are in agreement to those reported in the literature[13].

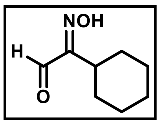

2-cyclohexyl-2-(hydroxyimino)ethanal (1j). Reaction performed on 400 mg (3.17 mmol) of 2-cyclohexylethanal. Flash chromatography was performed with a mixture of petroleum ether/Et_2O in gradient from 8:1 to 5:1. White solid, 374 mg (2.41mmol), 76 % yield, m.p. = 84-86 °C.

^{1}H-NMR (300 MHz, DMSO-*d$_6$*) δ 12.86 (s, 1H), 9.292 and 9.287 (s, 1H, *syn* and *anti* conformations), 3.10 – 2.70 (m, 1H), 1.99 – 1.52 (m, 5H), 1.42 – 1.37 (m, 2H), 1.21 – 1.15 (m, 3H).

^{13}C{^{1}H}**-NMR** (75 MHz, DMSO-*d$_6$*) δ 192.2, 161.4, 33.8, 26.9, 25.9, 25.5. Data are in agreement to those reported in the literature[12].

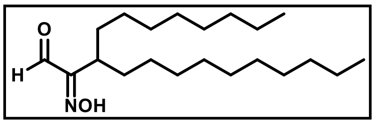

2-(hydroxyimino)-3-octyltridecanal (1i). Reaction performed on 350 mg (1.03 mmol) of 3-octyltridecanal. Flash chromatography was performed with a mixture of hexane/Et_2O in ratio 90:1. Gray solid, 210 mg (0.62 mmol), 60 % yield.

^{1}H-NMR (300 MHz, DMSO-*d$_6$*) δ : 12.8 (s, 1H); 9.34 (s, 1H); 3.06 (m, 1H); 1.68 (m, 2H); 1.46 (m, 2H); 1.19 (m, 28H), 0.839 (t, *J* = 6.5 Hz, 6H).

^{13}C{^{1}H}**-NMR** (75 MHz, DMSO-*d$_6$*) δ 192.2; 160.8; 34.4; 31.3; 31.3; 30.7; 29.0; 28.9; 28.9; 28.7; 28.7; 27.3; 22.12; 22.1; 13.9. Data are in agreement to those reported in the literature[11].

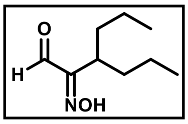

2-(hydroxyimino)-3-propylhexanal (1h). Reaction performed on 95 mg (0.67 mmol) of 3-propylhexanal. Flash chromatography was performed with a mixture of pentane/Et_2O in gradient from 15:1 to 10:1. Colorless oil, 36 mg (0.21 mmol), 32 % yield.

^{1}H-NMR (300 MHz, DMSO-*d$_6$*) δ 12.85 (s, 1H), 9.336 and 9.331 (s, 1H, *syn* and *anti* conformations), 3.20 – 2.99 (m, 1H), 1.74 – 1.62 (m, 2H), 1.50 – 1.31 (m, 2H), 1.20 – 0.96 (m, 4H), 0.79 (t, *J* = 7.3 Hz, 6H).

^{13}C{^{1}H}**-NMR** (75 MHz, DMSO-*d$_6$*) δ 192.4, 160.9, 33.9, 33.0, 20.6, 13.9.

ESI-HRMS (negative): calculated for $C_9H_{16}NO_2$ [M-H]$^-$, 170.1187; found 170.1189 ± 3.5 x 10^{-4} u, averaged from five measurements.

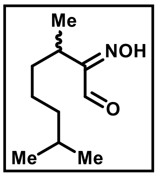

2-(hydroxyimino)-3,7-dimethyloctanal (1g). Reaction performed on 459 mg (2.94 mmol) of 3,7-dimethyloctanal. Flash chromatography was performed with a mixture of pentane/Et_2O in gradient from 15:1 to 5:1. Colorless oil, 434 mg (2.35 mmol), 80 % yield.

^{1}H NMR (300 MHz, DMSO-*d$_6$*) δ 12.83 (s, 1H), 9.32 (s, 1H).
3.20 – 3.01 (m, 1H), 1.80 – 1.58 (m, 1H), 1.42 (m, 2H), 1.13 – 1.03 (m, 7H), 0.83 – 0.74 (m, 6H).

^{13}C{^{1}H} **NMR** (75 MHz, DMSO-*d$_6$*) δ 192.2, 161.7, 38.2, 32.2, 28.6, 27.2, 25.1, 22.4, 16.2.

ESI-HRMS (negative): calculated for $C_{10}H_{18}NO_2$ [M-H]$^-$, 184.1343; found 184.1345 ± 3.5 x 10^{-4} u, averaged from five measurements.

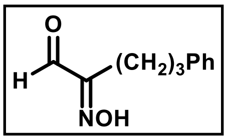

2-(hydroxyimino)-5-phenylpentanal (1e). Reaction performed on 795 mg (4.91 mmol) of 3,7-dimethyloctanal. Flash chromatography was performed with a mixture of pentane/Et_2O in gradient from 12:1 to 5:1. Colorless oil, 643 mg (3.37 mmol), 69 % yield.

^{1}H NMR (300 MHz, DMSO) δ 12.90 (s, 1H), 9.39 (s, 1H), 7.28 – 7.23 (m, 2H), 7.19 – 7.10 (m, 3H), 2.59 – 2.45 (m, 2H), 2.40 – 2.31 (m, 2H), 1.75 – 1.57 (m, 2H).

^{13}C{^{1}H} **NMR** (75 MHz, DMSO) δ 191.9, 159.5, 141.5, 128.3, 128.1, 125.8, 35.2, 26.8, 21.2.

ESI-HRMS (negative): calculated for $C_{11}H_{12}NO_2$ [M-H]$^-$ 190.0874; found 190.0875 (error -0.77 ppm).

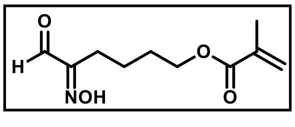

5-(hydroxyimino)-6-oxohexyl 2-methylprop-2-enoate (1f). Reaction performed on 829 mg (4.51 mmol) of 6-oxohexyl 2-methylprop-2-enoate. Flash chromatography was performed with a mixture of hexane/EtOAc in gradient from 30:1 to 8:1. Colorless oil, 575 mg (2.70 mmol), 60 % yield.

^{1}H-NMR (300 MHz, DMSO-*d$_6$*) δ 9.79 (s, 1H); 9.45 (s, 1H); 6.1 (m, 1H); 5.5 (m, 1H); 4.1 (t, 2H, *J* = 6.2 Hz); 2.5 (t, 2H, *J* = 7.5 Hz); 1.9 (m, 3H); 1.5-1.7 (m, 4H). Data are in agreement to those reported in the literature[11].

2.6. General procedure for Cu catalyzed azide-alkyne Huisgen cycloaddition

1.0 mmol of alkyne and 1.0 mmol of heptylazide are dissolved in 4 mL acetonitrile in a schlenk tube equipped with magnetic stirring. Then, 0.23 mL (0.21 g, 2.0 mmol) of 2,6-lutidine and 0.35 mL (0.26 g, 2.0 mmol) of di*iso*propylethylamine are added and the solution is degassed through three freeze-pump-thaw cycles under Argon. 19 mg of CuI (0.10 mmol) is added under Ar. The reaction is stirred for 3 h at room temperature. Most of the CH_3CN is removed by rotary evaporation and the resulting residue is dissolved in 50 mL EtOAc. The organic phase is washed three times with 25 mL NH_4Cl (sat), then with 25 mL HCl 0.1 M, and finally with 25 mL distilled water. After drying over anhydrous sodium sulfate, the solvent is evaporated and the residue is purified by column chromatography on silica gel. Removal of residual copper ions was achieved by washing a dichloromethane solution of the product several times with EDTA 0.1 M, previously adjusted at pH 7.6 (10:1 organic phase/EDTA solution).

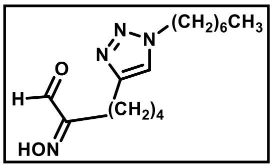

6-(1-heptyl-1H-1,2,3-triazol-4-yl)-2-(hydroxyimino)hexanal (1c). Reaction performed on 188 mg (1.23 mmol) of 2-(hydroxyimino)-7-octynal. White solid, 295 mg (1.00 mmol), 82 % yield, m.p. = 42-44 °C.
^{1}H-NMR (300 MHz, DMSO-*d*$_6$) δ : 12.89 (s, 1H), 9.38 (s, 1H), 7.79 (s, 1H), 4.25 (t, *J* = 7.1 Hz, 2H), 2.56 (t, *J* = 7.4 Hz, 2H), 2.35 (t, *J* = 7.4 Hz, 2H), 1.85 – 1.65 (m, 2H), 1.59 – 1.45 (m, 2H), 1.44 – 1.29 (m, 2H), 1.21 (m, 8H), 0.83 (t, *J* = 6.8 Hz, 3H).
^{13}C{^{1}H}**-NMR** (75 MHz, DMSO-*d*$_6$) δ δ 191.9, 159.7, 146.5, 121.6, 49.1, 31.1, 29.7, 29.1, 28.0, 25.8, 24.7, 24.6, 22.0, 21.0, 13.9. All analytical data are in accordance with literature reports. [13]

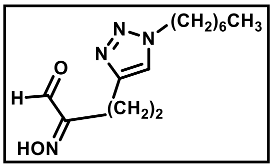

4-(1-heptyl-1H-1,2,3-triazol-4-yl)-2-(hydroxyimino)butanal (1d). Reaction performed on 96 mg (1.0 mmol) of 2-(hydroxyimino)-5-hexynal. Flash chromatography was performed using hexane/EtOAc in ratio 1:1. White solid, 160 mg (0.60 mmol), 60 % yield, m.p. = 64-66 °C.
^{1}H-NMR (300 MHz, DMSO-*d*$_6$) δ 13.00 (s, 1H); 9.39 (s, 1H,); 7.86 (s, 1H); 4.27 (t, *J* = 7.0 Hz, 2H); 2.80 – 2.60 (m, 4H); 1.76 (m, *J* = 7.14 Hz, 2H); 1.24 (m, 8H); 0.85 (t, *J* = 6,48 Hz, 3H).
^{13}C{^{1}H}**-NMR** (75 MHz, DMSO-*d*$_6$) δ 191.5; 158.7; 145.5; 121.8; 49.1; 31.1; 29.7; 28.1; 25.8; 22.0; 21.6; 20.9; 13.9. All analytical data are in accordance with literature reports. [13]

2.7. General procedure for methylation of 2-(hydroxyimino)aldehydes

The 2-(hydroxyimino)aldehyde (1.3 mmol, 1.0 equiv) and K_2CO_3 (5.2 mmol, 4 equiv) are dissolved in 4 mL of acetone. The resulting suspension is stirred for 10 minutes, then DMS (dimethyl sulfate, 2.6 mmol, 2.0 equiv) is added dropwise. After 2.5 h, 25 mL of H_2O are added to the reaction mixture and stirred for 15 minutes, then the aqueous solution is extracted with diethyl ether (3 x 20 mL).The reunited organic layers are washed with saturated $NaHCO_3$ (1 x 40 mL) and brine (1 x 40 mL), dried over Na_2SO_4 and the solvent evaporated at reduced pressure. The crude is purified through a quick filtration over a pad of silica gel.

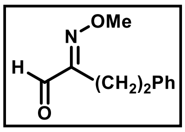

2-(methoxyimino)-4-phenylbutanal (1k). Reaction performed on 164 mg (0.93 mmol) of 2-(hydroxyimino)-4-phenylbutanal (**1a**). Flash chromatography was performed with a mixture of petroleum ether/Et_2O in ratio 30:1. Colorless liquid, 159 mg (0.83 mmol), 90 % yield.
^{1}H-NMR (300 MHz, DMSO-*d*$_6$) δ 9.32 (s, 1H), 7.28 – 7.24 (m, 2H), 7.20 – 7.11 (m, 3H), 4.05 (s, 3H), 2.77 – 2.54 (m, 4H).
^{13}C{^{1}H}**-NMR** (75 MHz, DMSO-*d*$_6$) δ 190.4, 158.6, 140.5, 128.3, 128.1, 126.2, 63.7, 30.7, 23.8.

ESI-HRMS (positive): calculated for $C_{11}H_{14}NO_2$ [M+H]$^+$, 192.1019; found 192.1021 ± 4.0 x 10^{-4} u, averaged from six measurements.

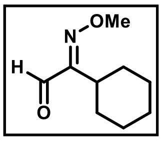

2-cyclohexyl-2-(methoxyimino)ethanal (1n). Reaction performed on 200 mg (1.29 mmol) of 2-cyclohexyl-2-(hydroxyimino)ethanal (**1j**). Flash chromatography was performed with a mixture of petroleum ether/Et_2O in ratio 50:1. White solid, 188 mg (1.11 mmol), 86 % yield, m.p. = 51-52 °C.

^{1}H-NMR (300 MHz, $CDCl_3$) δ 9.318 and 9.314 (s, 1H, *syn* and *anti* conformations), 4.08 (s, 3H), 3.19 – 2.68 (m, 1H), 1.90 – 1.68 (m, 5H), 1.51 – 1.47 (m, 2H), 1.27 – 1.22 (m, 3H).
^{13}C{^{1}H}-NMR (75 MHz, $CDCl_3$) δ 191.4, 162.1, 63.7, 35.1, 27.5, 26.3, 25.8.
ESI-HRMS (positive): calculated for $C_9H_{16}NO_2$ [M+H]$^+$, 170.1176; found 170.1177 ± 3.5 x 10^{-4} u, averaged from five measurements.

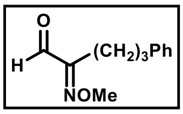

2-(methoxyimino)-5-phenylpentanal (1l). Reaction performed on 151 mg (0.79 mmol) of 2-(hydroxyimino)-5-phenylpentanal (**1e**). Flash chromatography was performed with a mixture of pentane/Et_2O in ratio 30:1. Colorless oil, 65 mg (0.32 mmol), 41 % yield.

^{1}H NMR (300 MHz, DMSO) δ 9.35 (s, 1H), 7.29 – 7.22 (m, 2H), 7.19 – 7.13 (m, 3H), 4.06 (s, 3H), 2.54 (m, 2H), 2.43 – 2.29 (m, 2H), 1.75 – 1.56 (m, 2H).
^{13}C{^{1}H} **NMR** (75 MHz, DMSO) δ 190.6, 159.2, 141.3, 128.3, 125.9, 63.6, 35.1, 26.7, 21.7.
ESI-HRMS (positive): calculated for $C_{12}H_{16}NO_2$ [M+H]$^+$ 206.1176: found 206.1176 (error -0.22 ppm).

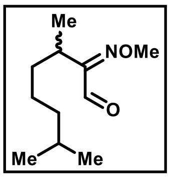

2-(methoxyimino)-3,7-dimethyloctanal (1m). Reaction performed on 120 mg (0.65 mmol) of 2-(hydroxyimino)-3,7-dimethyloctanal (**1g**). Flash chromatography was performed using pure petroleum ether followed by a mixture of petroleum ether/Et_2O in ratio 20:1. Colorless oil, 92 mg (0.46 mmol), 71 % yield.

^{1}H NMR (300 MHz, DMSO) δ 9.29 (d, J = 1.4 Hz, 1H), 4.05 (s, 3H), 3.16 – 2.97 (m, 1H), 1.63 (dt, J = 18.0, 8.9 Hz, 1H), 1.43 (td, J = 13.6, 6.8 Hz, 2H), 1.06 (t, J = 7.0 Hz, 7H), 0.79 (dd, J = 6.6, 3.5 Hz, 6H).
^{13}C{^{1}H} **NMR** (75 MHz, DMSO) δ 191.0, 161.6, 63.7, 38.1, 32.2, 29.3, 27.2, 25.0, 22.6, 22.3, 16.2.
ESI-HRMS (positive): calculated for $C_{11}H_{22}NO_2$ [M+H]$^+$, 200.16451; found 200.16465 ± 4.0 x 10^{-4} u, averaged from five measurements.

2.8. Synthesis of 2-((benzyloxy)imino)-2-cyclohexylethanal

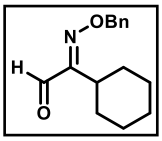

2-((benzyloxy)imino)-2-cyclohexylethanal (1o). In a round-bottom flask 96 mg (0.62 mmol, 1.0 equiv) of 2-cyclohexyl-2-(hydroxyimino)ethanal (**1j**) and 74 µL of benzyl bromide (106 mg, 0.62 mmol, 1.0 equiv) are dissolved in 3 mL of acetone, then 342 mg of K_2CO_3 (2.48 mmol, 4 equiv) are added slowly. After 1.5 h the crude is diluted with 20 mL of H_2O and extracted with diethyl ether (4 x 15 mL). The reunited organic layers are dried with Na_2SO_4 and the solvent is removed via evaporation at reduced pressure. The product is purified via flash chromatography using as eluant a mixture of petroleum ether/Et_2O in ratio 20:1. Light yellow solid, 147 mg (0.60 mmol), 97 % yield, m.p. = 38-39 °C.
^{1}H-NMR (300 MHz, DMSO-d_6) δ 9.278 and 9.274 (s, 1H, *syn* and *anti* conformations), 7.63 – 7.14 (m, 5H), 5.35 (s, 2H), 3.05 – 2.74 (m, 1H), 1.84 – 1.61 (m, 5H), 1.48 – 1.44 (m, 2H), 1.32 – 1.01 (m, 3H).
^{13}C{^{1}H}-NMR (75 MHz, DMSO-d_6) δ 190.9, 161.5, 136.7, 128.5, 128.2, 128.0, 77.3, 34.6, 26.9, 25.7, 25.4.
ESI-HRMS (positive): calculated for $C_{15}H_{20}NO_2$ [M+H]$^+$, 246.1489; found 246.1486 ± 3.5 x 10^{-4} u, averaged from six measurements.

2.9. General procedure for the photocyclization of 2-(hydroxyimino)aldehydes

The 2-(hydroxyimino)aldehyde or alkylated 2-(hydroxyimino)aldehyde (0.060 – 0.075 mmol, 1.0 equiv) is dissolved in the appropriate deuterated solvent (600 – 750 µL, C = 0.1 M) and placed in an NMR quartz tube. The sample is placed in the photoreactor (see section **1.1** of the supporting information) and irradiated with a 365 nm LED light source while thermostated at 25 °C with a cooling fan. Reaction completion is monitored with ^{1}H-NMR until disappearance of the aldehydic proton of the 2-(hydroxyimino)aldehyde.

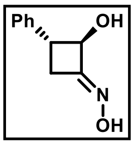

(±)-2-hydroxy-3-phenylcyclobutan-1-one oxime (2a). Reaction performed on 11 mg (0.062 mmol) of 2-(hydroxyimino)-4-phenylbutanal (**1a**). Conversion > 99 %, 99% yield, dr (*trans:cis*) = 1 : 0.8, isolated 11 mg as colorless oil, characterized without purification. Diastereomeric ratio determined via ^{1}H-NMR.

^{1}H-NMR (300 MHz, DMSO-d_6) δ 10.51 (s, 1H, *trans* diastereomer), 10.46 (s, 1H, *cis* diastereomer), 7.34 – 7.15 (m, 5H + 5H, both diastereomers), 6.05 (d, *J* = 8.0 Hz, 1H, *cis* diastereomer), 5.49 (d, *J* = 6.6 Hz, 1H, *trans* diastereomer), 5.07 – 4.96 (m, 1H, *trans* diastereomer), 4.63 (td, *J* = 7.8, 2.9 Hz, 1H, *cis* diastereomer), 3.58 (td, *J* = 8.7, 6.0 Hz, 1H, *trans* diastereomer), 3.23 (td, *J* = 9.4, 7.0 Hz, 1H, *cis* diastereomer), 3.07 (ddd, *J* = 16.1, 9.9, 2.9 Hz, 1H, *cis* diastereomer), 2.91 (ddd, *J* = 16.9, 9.1, 0.8 Hz, 1H, *trans* diastereomer), 2.79 (ddd, *J* = 16.9, 6.0, 2.8 Hz, 1H, *trans* diastereomer), 2.47 – 2.39 (m, 1H, *cis* diastereomer, partially overlapped with residual DMSO signal).

^{13}C{^{1}H}**-NMR** (75 MHz, DMSO-d_6) *trans* diastereomer: δ 158.5, 139.7, 128.6, 127.8, 126.0, 72.9, 41.2, 32.0; *cis* diastereomer: δ 157.2, 142.3, 128.4, 126.8, 126.4, 78.6, 45.3, 30.0.

ESI-HRMS (positive): calculated for $C_{10}H_{11}NO_2Na$ [M+Na]$^+$, 200.0682; found 200.0686 ± 4.5 x 10^{-4} u, averaged from five measurements.

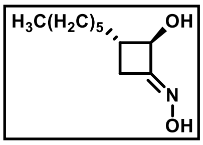

(±)-3-hexyl-2-hydroxycyclobutan-1-one oxime (2b). Reaction performed on 11 mg (0.062 mmol) of 2-(hydroxyimino)decanal (**1b**). Conversion > 99 %, 99% yield, dr (*trans:cis*) = 1 : 0.8, isolated 11 mg as colorless oil, characterized without purification. Diastereomeric ratio determined via ^{1}H-NMR.

^{1}H-NMR (300 MHz, DMSO-d_6) *trans* and *cis* diastereomers: δ 10.27 (s, 1H), 10.26 (s, 1H), 5.59 (d, *J* = 7.8 Hz, 1H), 5.42 (d, *J* = 7.2 Hz, 1H), 4.71 (dt, *J* = 7.2, 2.8 Hz, 1H), 4.20 (td, *J* = 7.5, 3.1 Hz, 1H), 2.71 (ddd, *J* = 15.9, 9.0, 3.1 Hz, 1H), 2.46 – 2.39 (m, 1H), 2.29 – 2.10 (m, 2H), 1.98 (dt, *J* = 12.2, 8.1 Hz, 1H), 1.91 – 1.80 (m, 1H), 1.31 – 1.14 (m, 20H), 0.91 – 0.78 (m, 6H).

^{13}C{^{1}H}**-NMR** (75 MHz, DMSO-d_6) *trans* and *cis* diastereomers: δ 159.5, 157.9, 76.6, 71.5, 40.6, 35.7, 33.7, 31.3, 31.25, 30.8, 29.1, 28.9, 28.6, 28.4, 27.1, 26.9, 22.1, 14.0.

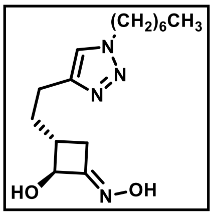

(±)-3-(2-(1-heptyl-1H-1,2,3-triazol-4-yl)ethyl)-2-hydroxycyclobutan-1-one oxime (2c). Reaction performed on 11 mg (0.037 mmol) of 6-(1-heptyl-1H-1,2,3-triazol-4-yl)-2-(hydroxyimino)hexanal (**1c**). Conversion > 99 %, 99% yield, isolated 11 mg as colorless oil, dr (*trans:cis*) = 1 : 0.8, characterized without purification. Diastereomeric ratio determined via ^{1}H-NMR.

^{1}H-NMR (300 MHz, DMSO-d_6) *trans* and *cis* diastereomers: δ 10.33 (s, 1H), 10.30 (s, 1H), 7.84 (s, 2H), 5.67 (d, *J* = 7.7 Hz, 1H), 5.53 (d, *J* = 7.2 Hz, 1H), 4.76 (dt, *J* = 7.2, 2.9 Hz, 1H), 4.35 – 4.31 (m, 1H), 4.27 (t, *J* = 7.1 Hz, 4H), 2.72 (ddd, *J* = 16.0, 9.1, 3.2 Hz, 1H), 2.62 (t, *J* = 7.6 Hz, 4H), 2.39 – 2.19 (m, 2H), 2.11 – 1.81 (m, 4H), 1.81 – 1.70 (m, 4H), 1.61 – 1.46 (m, 2H), 1.35 – 1.10 (m, 20H), 0.84 (t, *J* = 6.8 Hz, 6H).

^{13}C{^{1}H}**-NMR** (75 MHz, DMSO-d_6) *trans* and *cis* diastereomers: δ 159.2, 157.7, 146.8, 146.4, 121.7, 121.6, 76.4, 71.5, 49.1, 40.2, 35.2, 33.4, 31.1, 30.7, 29.7, 28.9, 28.7, 28.0, 25.8, 23.2, 23.1, 22.0, 13.9, 13.9.

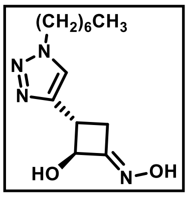

(±)-3-(1-heptyl-1H-1,2,3-triazol-4-yl)-2-hydroxycyclobutan-1-one oxime (2d). Reaction performed on 13 mg (0.045 mmol) of 4-(1-heptyl-1H-1,2,3-triazol-4-yl)-2-(hydroxyimino)butanal (**1d**). Conversion > 99 %, 99% yield, dr (*trans:cis*) = 1 : 0.8, isolated 13 mg as colorless oil, characterized without purification. Diastereomeric ratio determined via ^{1}H-NMR.

^{1}H-NMR (300 MHz, DMSO-d_6) *trans* and *cis* diastereomers: δ 10.52 (s, 1H), 10.50 (s, 1H), 8.02 (s, 1H), 7.85 (s, 1H), 6.04 (d, *J* = 8.1 Hz, 1H), 5.56 (d, *J* = 6.9 Hz, 1H), 4.98 (t, *J* = 7.0 Hz, 1H), 4.70 (td, *J* = 7.4, 2.9 Hz, 1H), 4.30 (t, *J* = 7.1 Hz, 4H), 3.69 (dd, *J* = 15.3, 7.9 Hz, 1H), 3.32 – 3.23 (m, *J* = 9.4, 7.2 Hz, 1H), 3.07 (ddd, *J* = 16.4, 10.1, 3.1 Hz, 1H), 2.94 – 2.86 (m, 4H), 2.53 – 2.43 (m, 1H), 1.85 – 1.70 (m, *J* = 14.0, 7.0 Hz, 4H), 1.35 – 1.11 (m, 14H), 0.85 (t, *J* = 6.8 Hz, 6H).
^{13}C{^{1}H}-NMR (75 MHz, DMSO-d_6) *trans* and *cis* diastereomers: δ 158.2, 157.1, 147.7, 144.8, 122.8, 121.7, 77.9, 72.2, 49.3, 49.2, 36.8, 33.3, 31.5, 31.1, 30.4, 29.8, 29.7, 28.1, 28.1, 25.8, 22.0, 13.9.

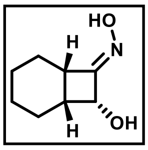

(±)-*Endo*-*cis*-(*E*)-8-hydroxybicyclo[4.2.0]octan-7-one oxime (2j). Reaction performed on 11 mg (0.062 mmol) of 2-cyclohexyl-2-(hydroxyimino)ethanal (**1j**). Conversion > 99 %, 99% yield, isolated 11 mg as thick colorless oil, characterized without purification.
^{1}H-NMR (300 MHz, DMSO-d_6) δ 9.93 (s, 1H), 5.37 (d, *J* = 6.6 Hz, 1H), 4.63 – 4.31 (m, 1H), 2.85 – 2.61 (m, 1H), 2.41 – 1.98 (m, 2H), 1.61 – 1.23 (m, 5H), 1.17 – 0.92 (m, 2H).
^{13}C{^{1}H}-NMR (75 MHz, DMSO-d_6) δ 160.6, 69.8, 36.9, 32.8, 23.2, 22.7, 22.1, 21.0.

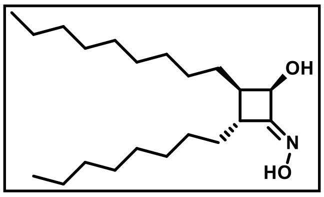

(±)-2-hydroxy-3-nonyl-4-octylcyclobutan-1-one oxime (2i). Please note that since the HIA **1i** possesses two different γ hydrogen, it is possible to also form the 2-decyl-3-heptyl-4-hydroxycyclobutan-1-one oxime regioisomer, the two being indistinguishable from one another through NMR analysis. Reaction performed on 10 mg (0.03 mmol) of 2-(hydroxyimino)-3-octyltridecanal (**1i**).Conversion > 99 %, 99% yield, isolated 11 mg as thick colorless oil, characterized without purification.
^{1}H-NMR (300 MHz, DMSO-d_6) δ 10.16 (s, 1H), 5.44 (d, *J* = 7.1 Hz, 1H), 4.02 (t, *J* = 6.3 Hz, 1H), 2.28 (td, *J* = 8.4, 5.1 Hz, 1H), 1.95 – 1.81 (m, 1H), 1.67 (dt, *J* = 13.7, 7.0 Hz, 1H), 1.55 – 1.39 (m, 3H), 1.31 – 1.19 (m, 26H), 0.85 (t, *J* = 6.4 Hz, 6H).
^{13}C{^{1}H}-NMR (75 MHz, DMSO-d_6) δ 159.9, 73.8, 45.8, 44.4, 33.7, 33.7, 31.3, 31.3, 30.5, 29.1, 29.0, 29.0, 28.9, 28.7, 28.6, 27.0, 26.9, 26.5, 26.5, 22.1, 13.9.

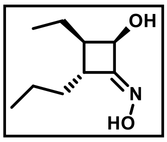

(±)-3-ethyl-2-hydroxy-4-propylcyclobutan-1-one oxime (2h). Reaction performed on 11 mg (0.064 mmol) of 2-(hydroxyimino)-3-propylhexanal (**1h**). Conversion > 99 %, 99% yield, isolated 11 mg as thick colorless oil, characterized without purification.
^{1}H-NMR (300 MHz, DMSO-d_6) tabulation referred to the major diastereomer: δ 10.18 (s, 1H), 5.47 (d, *J* = 7.1 Hz, 1H), 4.05 (t, *J* = 6.0 Hz, 1H), 2.31 (td, *J* = 8.4, 4.8 Hz, 1H), 1.97 – 1.81 (m, 1H), 1.68 – 1.22 (m, 6H), 0.95 – 0.79 (m, 6H).
^{13}C{^{1}H}-NMR (75 MHz, DMSO-d_6)) tabulation referred to the major diastereomer: δ 159.8, 73.5, 47.7, 44.0, 32.9, 26.5, 19.9, 14.1, 11.8.

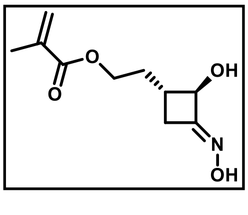

(±)-2-(2-hydroxy-3-(hydroxyimino)cyclobutyl)ethyl 2-methylprop-2-enoate (2f). Reaction performed on 13 mg (0.061 mmol) of 5-(hydroxyimino)-6-oxohexyl 2-methylprop-2-enoate (**1f**). Conversion > 99 %, 99% yield, isolated 13 mg as thick colorless oil, characterized without purification.
^{1}H NMR (300 MHz, DMSO-d_6) *trans* and *cis* diastereomers: δ 10.36 (s, 1H), 10.32 (s, 1H), 6.08 – 5.94 (m, 1H), 5.68 – 5.59 (m, 3H), 4.82 – 4.72 (m, 1H), 4.37 – 4.22 (m, 1H), 4.19 – 4.04 (m, 2H), 4.02 – 3.77 (m, 2H), 2.74 (ddd, *J* = 15.9, 8.7, 3.4 Hz, 1H), 2.44 – 2.22 (m, 2H), 2.16 – 1.46 (m, 11H), 1.18 – 0.48 (m, 4H).
^{13}C{^{1}H} NMR (75 MHz, DMSO-d_6) *trans* and *cis* diastereomers: δ 166.6, 166.6, 158.9, 157.7, 136.0, 136.0, 125.7, 125.6, 76.3, 71.4, 63.2, 63.1, 37.9, 32.5, 32.3, 30.8, 29.0, 27.9, 18.0.

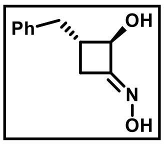

(±)-3-benzyl-2-hydroxycyclobutan-1-one oxime (2e). Reaction performed on 15 mg (0.078 mmol) of 5-(hydroxyimino)-6-oxohexyl 2-methylprop-2-enoate (**1e**). Conversion > 99 %, 99% yield, isolated 15 mg as thick colorless oil, characterized without purification.

^{1}H NMR (300 MHz, DMSO-*d_6*) *cis* and *trans* diastereomers: δ 10.39 (s, 1H), 10.34 (s, 1H), 7.30 – 7.14 (m, 10H), 5.74 – 5.52 (m, 2H), 4.82 (m, 1H), 4.36 (m, 1H), 2.98 – 2.84 (m, 2H), 2.75 – 2.53 (m, 4H), 2.32 – 2.21 (m, 3H), 2.03 (dd, *J* = 16.4, 8.2 Hz, 1H).

^{13}C{^{1}H} **NMR** (75 MHz, DMSO-*d_6*) *cis* and *trans* diastereomers: δ 158.9, 157.6, 140.7, 140.1, 128.8, 128.6, 128.3, 128.3, 125.9, 125.7, 76.1, 71.5, 41.5, 39.1, 37.2, 34.2, 30.6, 29.0.

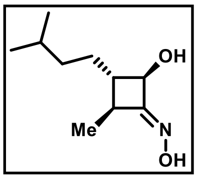

(±)-2-hydroxy-3-isopentyl-4-methylcyclobutan-1-one oxime (2g). Reaction performed on 10 mg (0.054 mmol) of 2-(hydroxyimino)-3,7-dimethyloctanal (**1g**). Conversion > 99 %, 99% yield, isolated 10 mg as thick colorless oil, characterized without purification.

^{1}H NMR (300 MHz, DMSO-*d_6*) tabulation referred to the major (*2R,3S,4S,E*) diastereomer: δ 10.12 (s, 1H), 5.47 (d, J = 7.6 Hz, 1H), 4.01 (t, J = 6.6 Hz, 1H), 2.37 – 2.20 (m, 1H), 1.56 – 1.39 (m, 2H), 1.26 (d, J = 7.1 Hz, 3H), 1.25 – 1.00 (m, 3H), 0.84 (d, J = 6.6 Hz, 6H).

^{13}C{^{1}H} **NMR** (75 MHz, DMSO) δ 160.7, 73.7, 48.6, 36.2, 30.8, 27.5, 22.5, 22.4, 17.0.

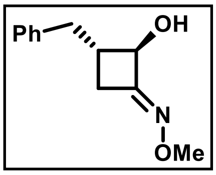

(±)-3-benzyl-2-hydroxycyclobutan-1-one O-methyl oxime (2l). Reaction performed on 34 mg (0.166 mmol) of 2-(methoxyimino)-5-phenylpentanal **(1l)**. Purification via flash chromatography using as eluant a mixture of pentane/CH_2Cl_2 in gradient from 4:1 to 1:1 followed by a mixture of pentane/CH_2Cl_2/Et_2O in gradient from 50:50:1 to 10:10:1. Colorless oil, 29 mg (0.141 mmol), 85 % yield as sum of *cis*/*trans* diastereomers, dr (*cis:trans*) = 1:1.3.

^{1}H NMR (600 MHz, CD_2Cl_2) *cis* diastereomer: δ 7.32 – 7.28 (m, 2H), 7.25 – 7.19 (m, 3H), 5.02 (ddd, *J* = 8.2, 6.8, 3.0 Hz, 1H), 3.86 (s, 3H), 3.08 (dd, *J* = 14.3, 6.0 Hz, 1H), 2.89 – 2.73 (m, 1H), 2.64 – 2.56 (m, 2H), 2.53 (ddd, *J* = 17.3, 4.7, 3.0 Hz, 1H), 2.36 (d, *J* = 6.6 Hz, 1H). *trans* diastereomer: δ 7.34 – 7.30 (m, 2H), 7.25 – 7.20 (m, 3H), 4.58 (td, *J* = 6.4, 3.2 Hz, 1H), 3.82 (s, 3H), 2.99 – 2.89 (m, 2H), 2.84 (dd, *J* = 14.1, 7.8 Hz, 1H), 2.51 – 2.44 (m, 1H), 2.33 (d, *J* = 6.9 Hz, 1H), 2.18 (dd, *J* = 16.7, 8.5 Hz, 1H).

^{13}C{^{1}H} **NMR** (75 MHz, CD_2Cl_2) *cis* diastereomer: δ 157.9 , 139.1, 128.2, 128.0, 125.6, 76.1 61.1, 41.5, 39.1, 29.4. *trans* diastereomer: δ 159.5, 139.7, 128.4, 127.9, 125.8, 71.9, 61.2, 36.8, 33.6, 30.7.

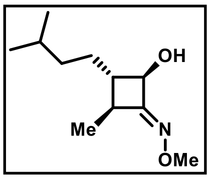

2-hydroxy-3-((3-methyl)-1-butyl)-4-methylcyclobutan-1-one O-methyl oxime (2m). Reaction performed on 27 mg (0.136 mmol) of 2-(hydroxyimino)-3,7-dimethyloctanal (**1m**). Purification via flash chromatography using as eluant a mixture of CH_2Cl_2/Et_2O in gradient from 100:1 to 30:1. Colorless oil, 24 mg (0.121 mmol), 89 % yield as sum of *trans/cis* diastereomers, dr (*trans:cis*) = 2 : 1.

^{1}H NMR (300 MHz, CD_2Cl_2) *trans* diastereomer: δ 4.24 (m, 1H), 3.79 (s, 3H), 2.71 (d, *J* = 6.4 Hz, 1H), 2.46 (p, *J* = 7.2 Hz, 1H), 1.68 – 1.54 (m, *J* = 7.2 Hz, 4H), 1.35 (d, *J* = 6.0 Hz, 3H), 1.23 – 1.28 (m, 2H), 0.90 (d, *J* = 6.6 Hz, 6H). *cis* diastereomer: δ 4.37 (dd, *J* = 6.3, 2.4 Hz, 1H), 3.72 (s, 3H), 2.99 (d, *J* = 2.5 Hz, 1H), 2.27 (dt, *J* = 13.8, 6.9 Hz, 1H), 1.61 – 1.59 (m, 1H), 1.57 – 1.45 (m, 2H), 1.20 – 1.11 (m, 6H), 0.90 (d, *J* = 6.6 Hz, 6H).

^{13}C{^{1}H} **NMR** (75 MHz, CD_2Cl_2) *trans* diastereomer: δ 161.6, 74.0, 61.1, 48.6, 40.2, 36.0, 30.8, 27.6 , 21.8, 21.8, 16.3. *cis* diastereomer: δ 161.7, 75.7 , 61.2, 47.1, 37.8, 36.0, 31.0, 27.5, 21.8 , 15.8.

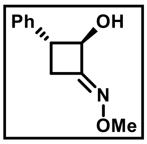

(±)-(*E*)-2-hydroxy-3-phenylcyclobutan-1-one O-methyl oxime (2k). Reaction performed on 19 mg (0.099 mmol) of 2-(methoxyimino)-4-phenylbutanal (**1k**). Purification via flash chromatography using as eluant CH_2Cl_2 followed by CH_2Cl_2/Et_2O in gradient from 50:1 to 10:1. Colorless oil, 18 mg (0.094 mmol), 95 % yield as sum of *cis*/*trans* diastereomers, dr (*E-trans:E-cis:Z-trans:Z-cis*) = 1 : 0.6 : 0.12 : 0.04.

^{1}H-NMR (300 MHz, DMSO-*d_6*) *E-trans* diastereomer: δ 7.32 – 7.24 (m, 2H), 7.22 – 7.15 (m, 3H), 5.62 (d, *J* = 6.5 Hz, 1H), 5.11 – 4.98 (m, 1H), 3.77 (s, 3H), 3.60 (td, *J* = 8.8, 5.8 Hz, 1H), 2.93 (ddd, *J* = 17.2, 9.3, 1.1

Hz, 1H), 2.78 (ddd, J = 17.2, 5.8, 2.9 Hz, 1H); *E-cis* diastereomer: δ 7.35 – 7.27 (m, 4H), 7.27 – 7.17 (m, 1H), 6.16 (d, J = 7.8 Hz, 1H), 4.65 (td, J = 7.6, 3.0 Hz, 1H), 3.76 (s, 3H), 3.24 (td, J = 9.5, 7.2 Hz, 1H), 3.05 (ddd, J = 16.2, 9.9, 2.9 Hz, 1H), 2.58 – 2.41 (m, 1H); *Z-trans* diastereomer: δ 7.36 – 7.11 (m, 5H), 5.52 (d, J = 6.9 Hz, 1H), 5.08 – 4.94 (m, 1H), 3.72 (s, 3H), 3.58 (dd, J = 16.4, 8.4 Hz, 1H), 3.02 – 2.81 (m, 2H); *Z-cis* diastereomer: δ 7.48 – 7.14 (m, 5H), 6.07 (d, J = 7.9 Hz, 1H), 4.65 (td, J = 7.7, 3.2 Hz, 1H), 3.71 (s, 3H), 3.24 (td, J = 10.0, 7.5 Hz, 1H), 2.90 (ddd, J = 16.2, 10.0, 3.0 Hz, 1H), 2.62 – 2.49 (m, 1H).
^{13}C{^{1}H}**-NMR** (75 MHz, DMSO-d_6) *E-trans* diastereomer: δ 160.2, 139.3, 128.6, 127.9, 126.1, 72.7, 61.1, 41.2, 32.3; *E-cis* diastereomer: δ 158.8, 141.9, 128.4, 126.8, 126.6, 78.4, 61.0, 45.1, 30.3; *Z-trans* diastereomer: δ 158.6, 139.1, 128.8, 127.8, 126.1, 72.4, 61.1, 40.3, 33.0.

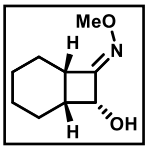

(±)-*Endo-cis*-(*E*)-8-hydroxybicyclo[4.2.0]octan-7-one O-methyl oxime (2n). Reaction performed on 30 mg (0.18 mmol, C = 0.3 M) of 2-cyclohexyl-2-(methoxyimino)ethanal (**1n**). Purification via chromatography over neutral Al_2O_3 using as eluant CH_2Cl_2/CH_3OH in ratio 70:1. Thick colorless oil, 28 mg (0.14 mmol), 93 % yield.
^{1}H-NMR (300 MHz, DMSO-d_6) δ 5.51 (d, J = 6.5 Hz, 1H), 4.53 (dd, J = 7.7, 6.6 Hz, 1H), 3.67 (s, 3H), 2.87 – 2.67 (m, 1H), 2.36 – 2.06 (m, 2H), 1.67 – 1.19 (m, 5H), 1.13 – 0.92 (m, 2H).
^{13}C{^{1}H}**-NMR** (75 MHz, DMSO-d_6) δ 162.3, 69.6, 61.1, 37.3, 32.6, 23.3, 22.6, 22.0, 21.0.

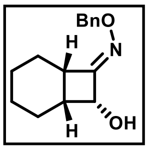

(±)-*Endo-cis*-(*E*)-8-hydroxybicyclo[4.2.0]octan-7-one O-benzyl oxime (2o). Reaction performed on 20 mg (0.082 mmol) of 2-((benzyloxy)imino)-2-cyclohexylethanal (**1o**). Purification via flash chromatography using as eluant CH_2Cl_2/Et_2O in gradient from 10:1 to 8:1. Light yellow oil, 19 mg (0.078 mmol), 95 % yield. Scale-up reaction performed on 265 mg (1.08 mmol) of 2-((benzyloxy)imino)-2-cyclohexylethanal (**1o**) in 4.3 mL of MeOH (C = 0.25 M) and 13 h reaction time. After flash chromatography 242 mg (0.99 mmol) of **2o** were obtained, 91 % yield.
^{1}H-NMR (300 MHz, DMSO-d_6) δ 7.48 – 7.05 (m, 5H), 5.55 (bs, 1H), 4.95 (s, 2H), 4.56 (d, J = 7.0 Hz, 1H), 2.81 (dd, J = 10.7, 4.3 Hz, 1H), 2.35 – 2.06 (m, 2H), 1.67 – 1.21 (m, 5H), 1.19 – 0.69 (m, 2H).
^{13}C{^{1}H}**-NMR** (75 MHz, DMSO-d_6) δ 163.0, 138.4, 128.2, 127.6, 127.5, 74.8, 69.7, 37.2, 32.6, 23.2, 22.6, 21.9, 21.0.
ESI-HRMS (positive): calculated for $C_{15}H_{20}NO_2$ $[M+H]^+$, 246.1489; found 246.1490 ± 4.0 x 10^{-4} u, averaged from five measurements.

3. Copy of NMR spectra of new compounds

^{1}H-NMR (300 MHz, DMSO-d_6)

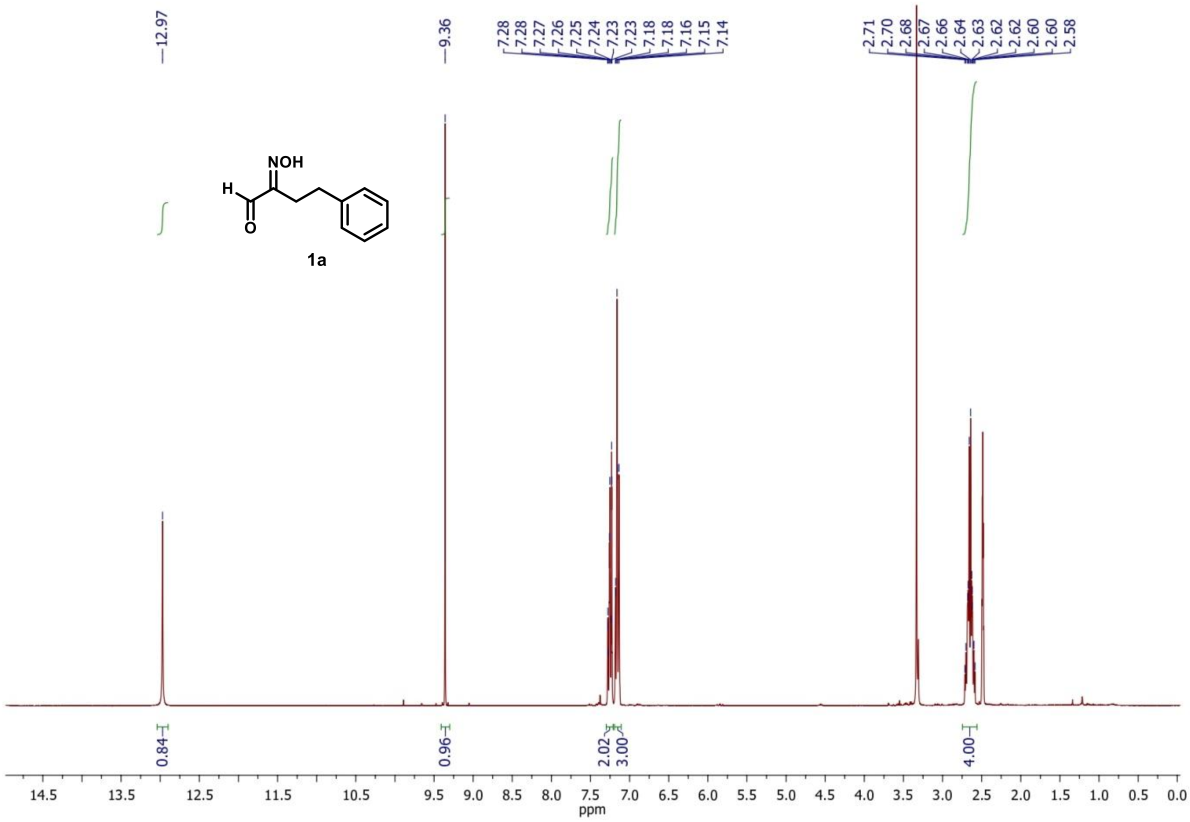

^{13}C{^{1}H}-NMR (75 MHz, DMSO-d_6)

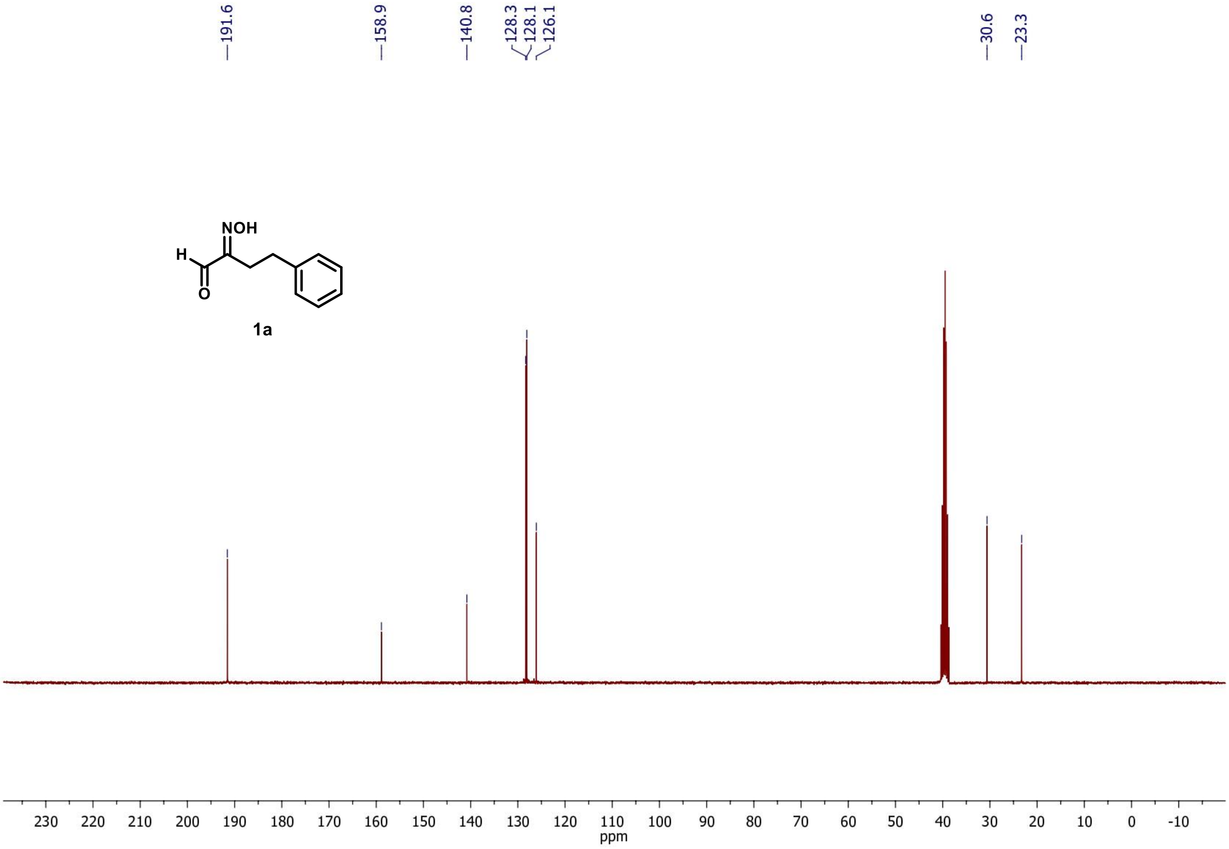

^{1}H-NMR (300 MHz, DMSO-d_6)

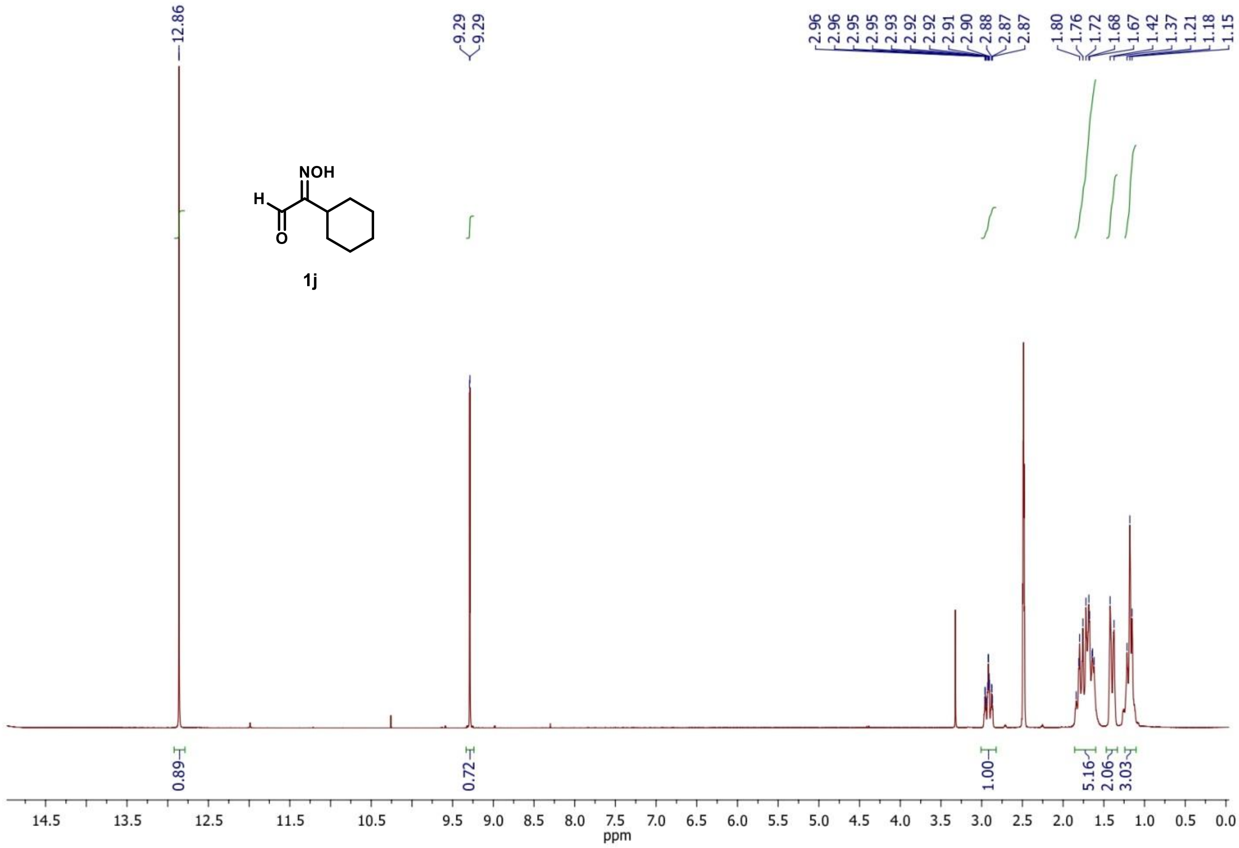

$^{13}C\{^{1}H\}$-NMR (75 MHz, DMSO-d_6)

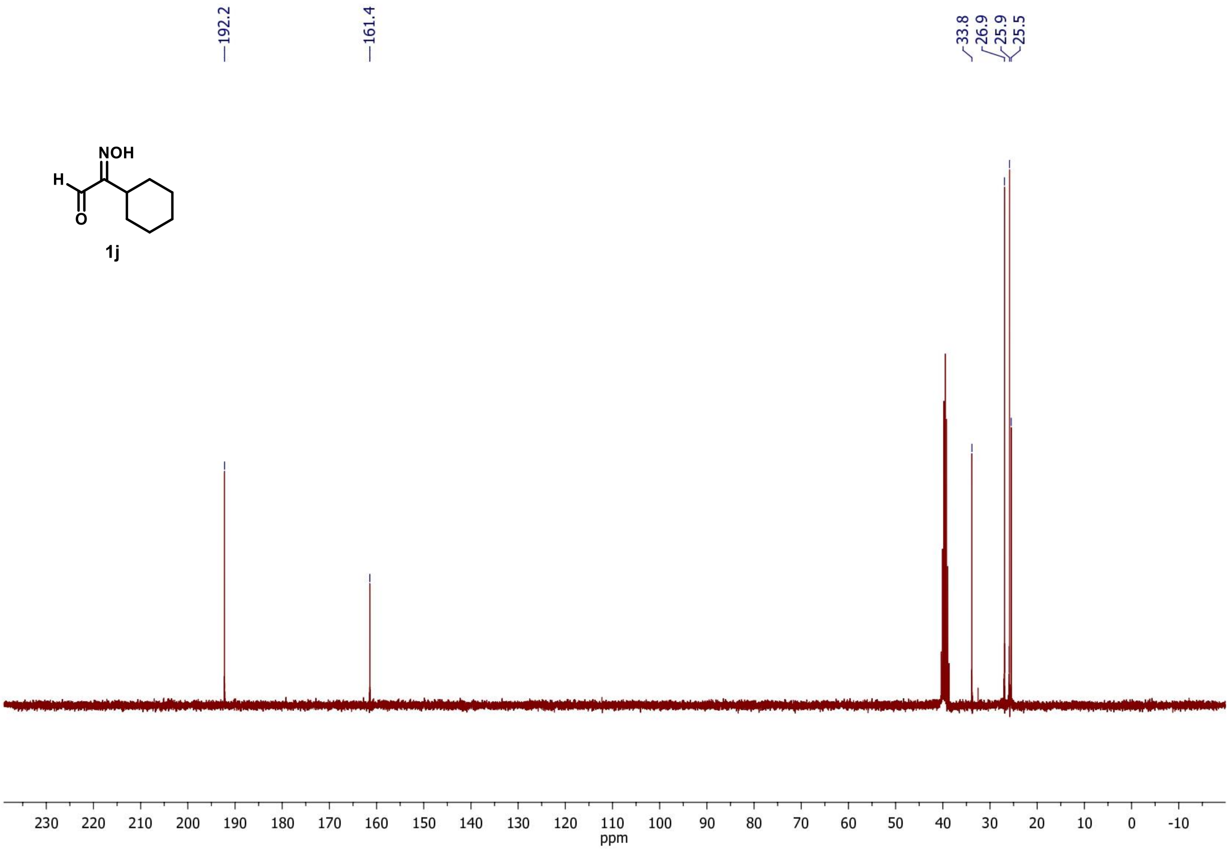

^{1}H-NMR (300 MHz, DMSO-d_6)

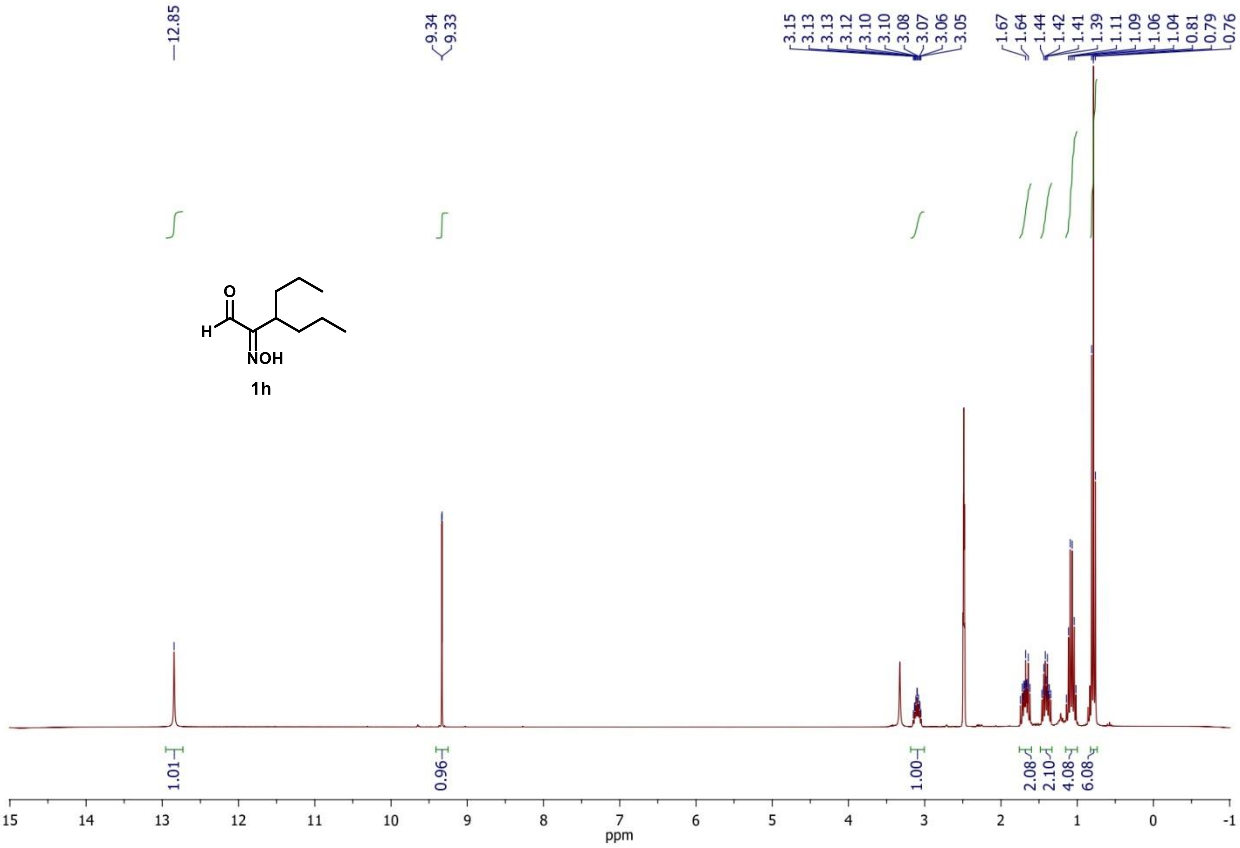

^{13}C{^{1}H}-NMR (75 MHz, DMSO-d_6)

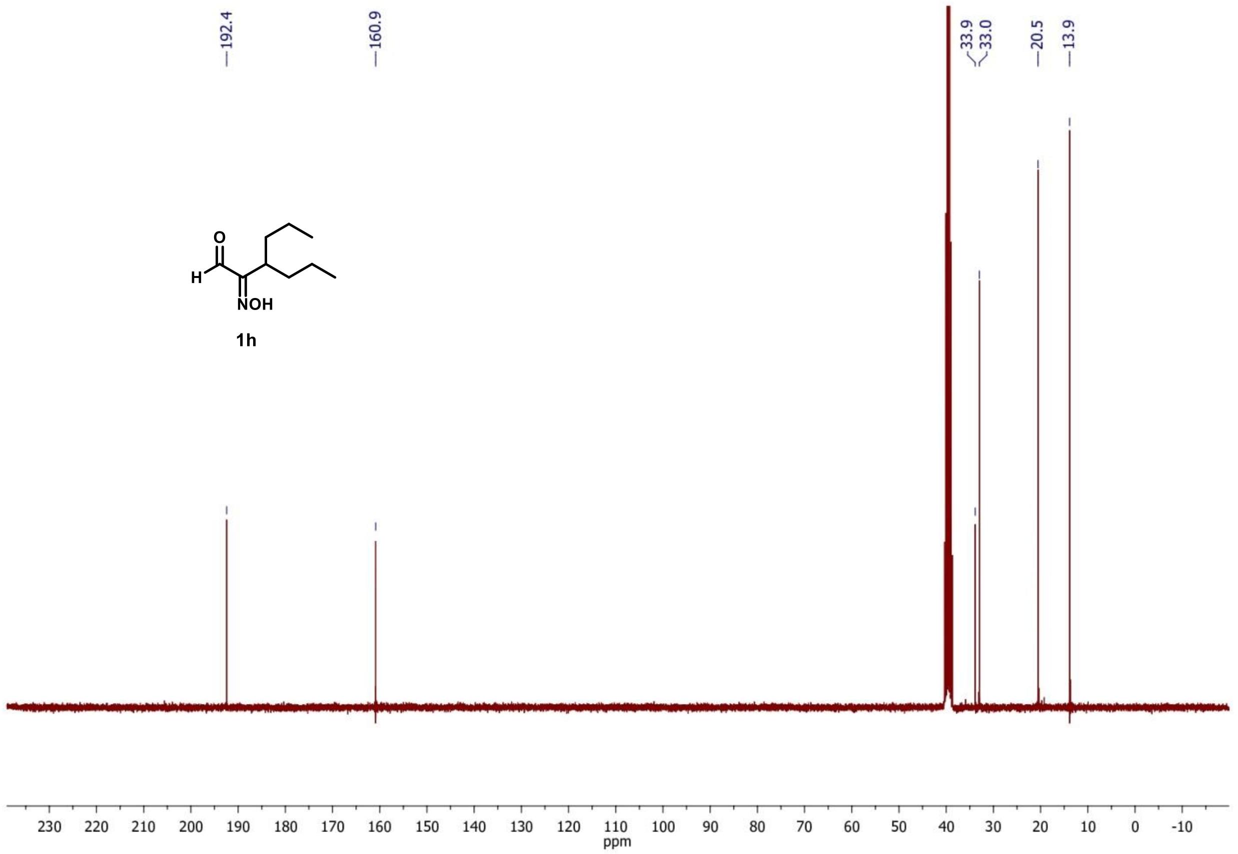

^{1}H-NMR (300 MHz, DMSO-d_6)

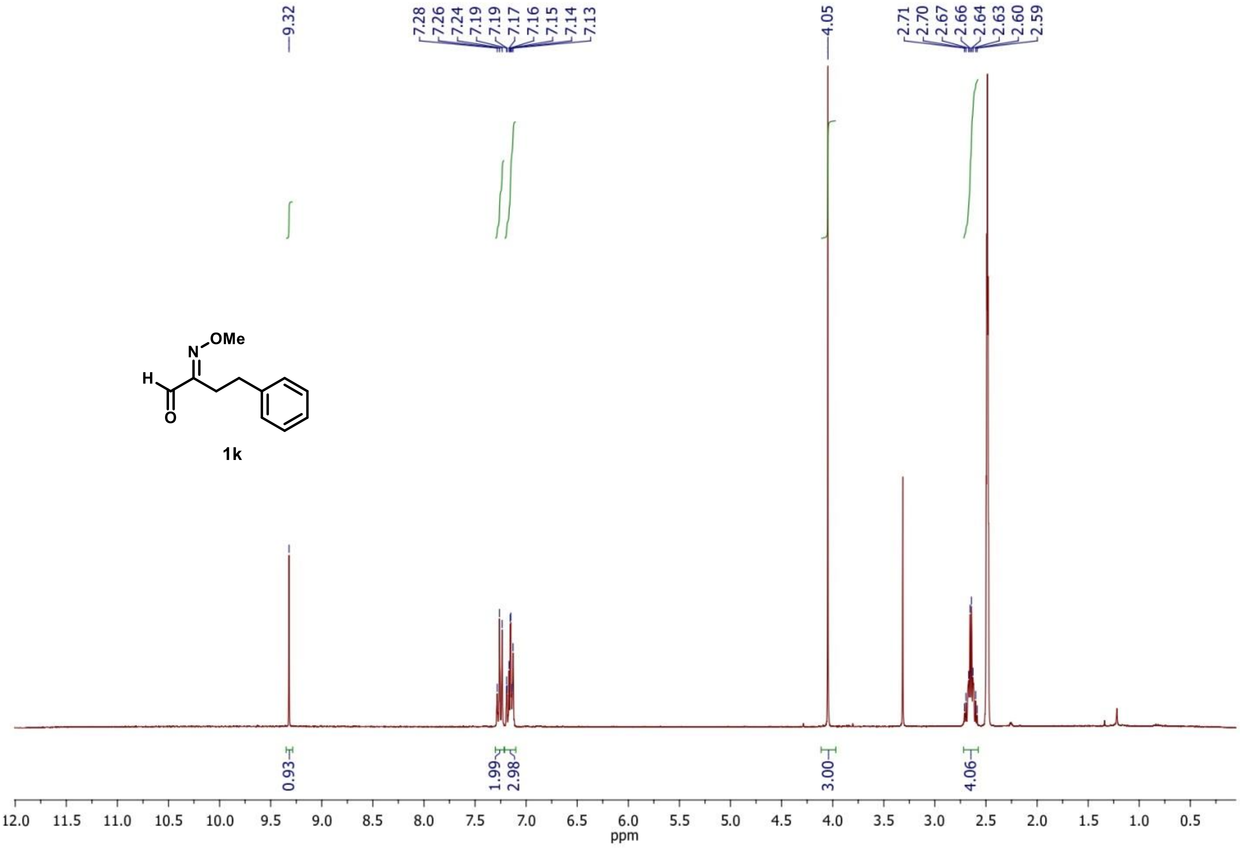

^{13}C{^{1}H}-NMR (75 MHz, DMSO-d_6)

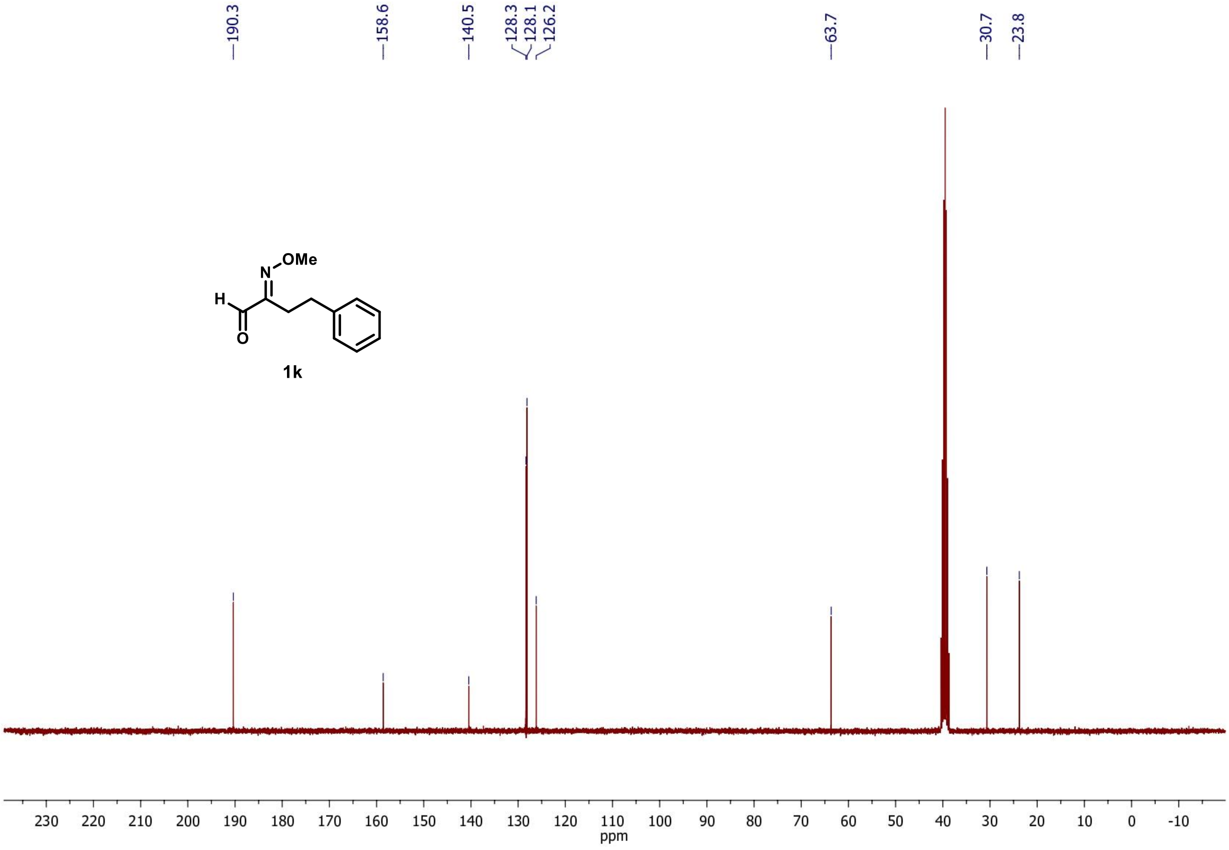

^{1}H-NMR (300 MHz,$CDCl_3$)

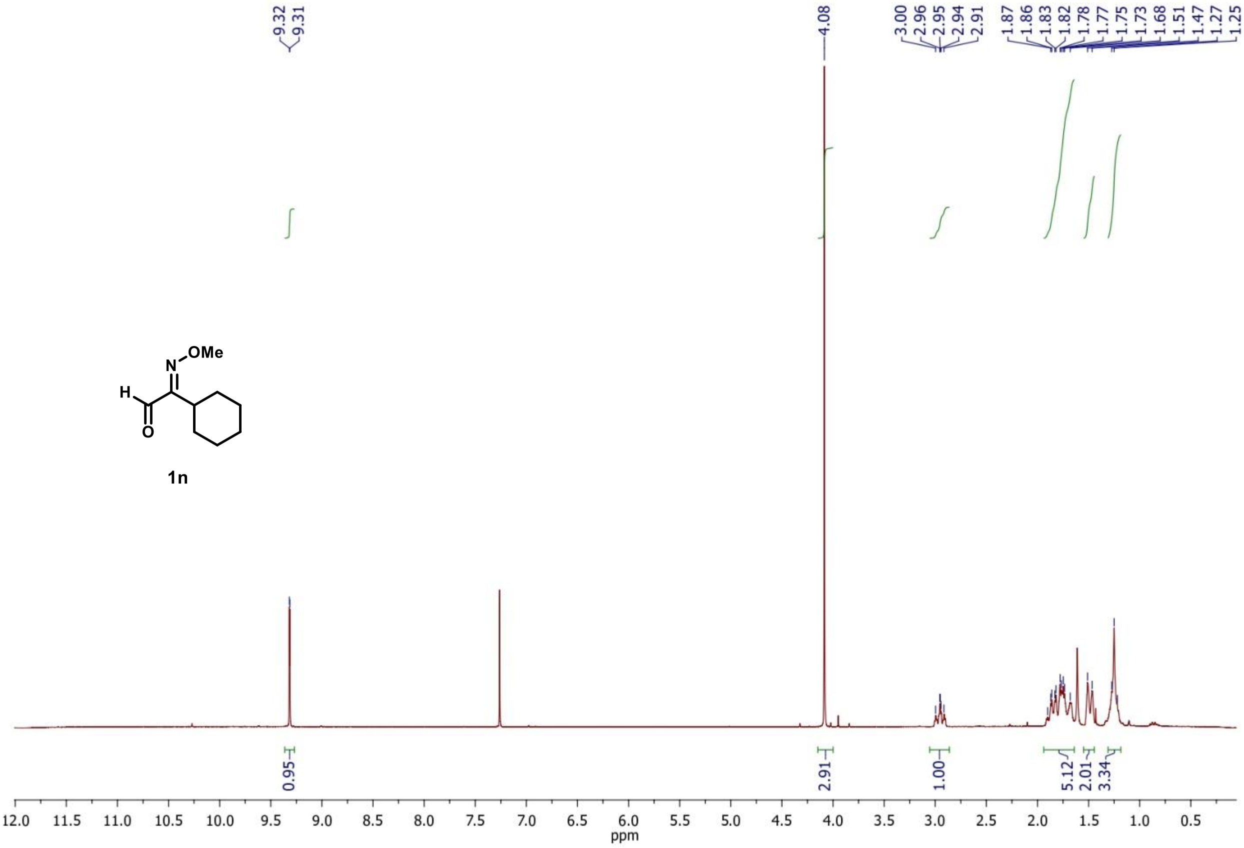

$^{13}C\{^{1}H\}$-NMR (75 MHz, $CDCl_3$)

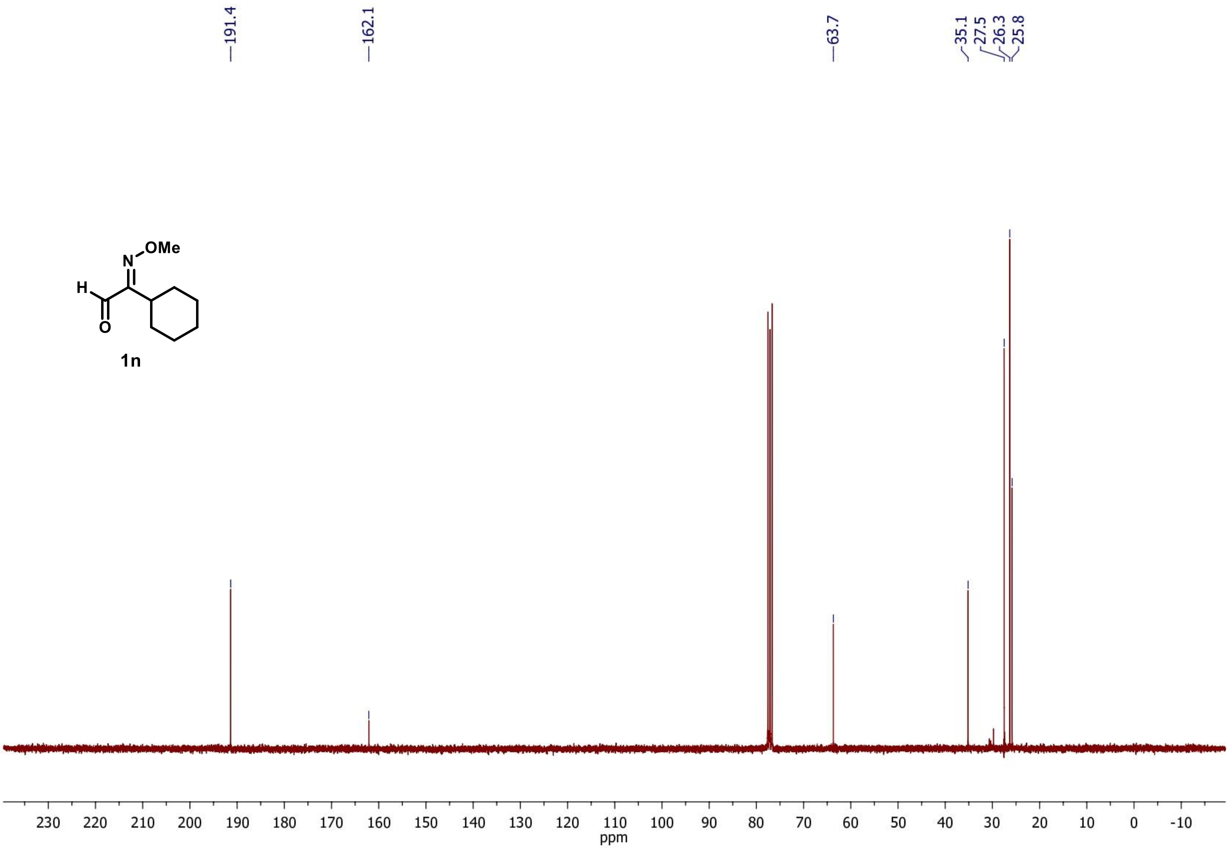

^{1}H-NMR (300 MHz, DMSO-d_6)

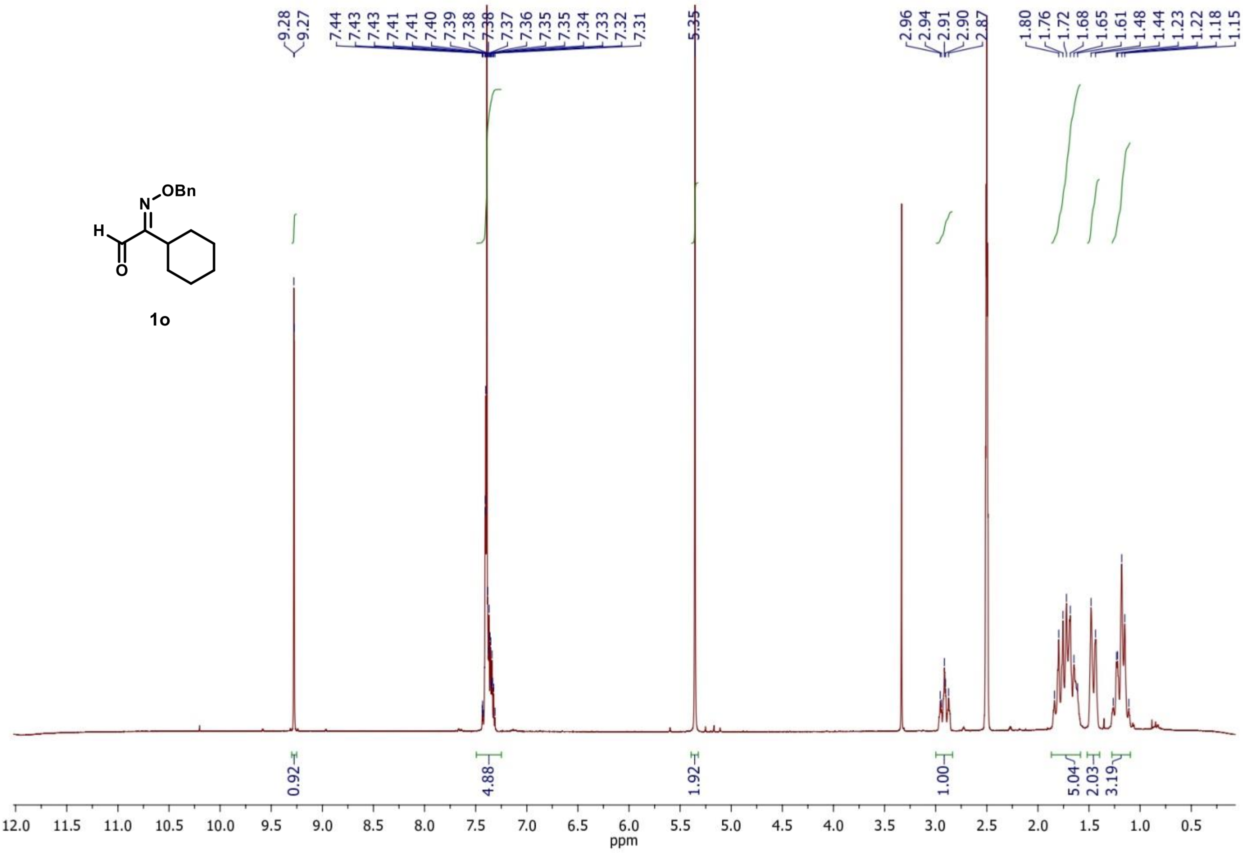

^{13}C{^{1}H}-NMR (75 MHz, DMSO-d_6)

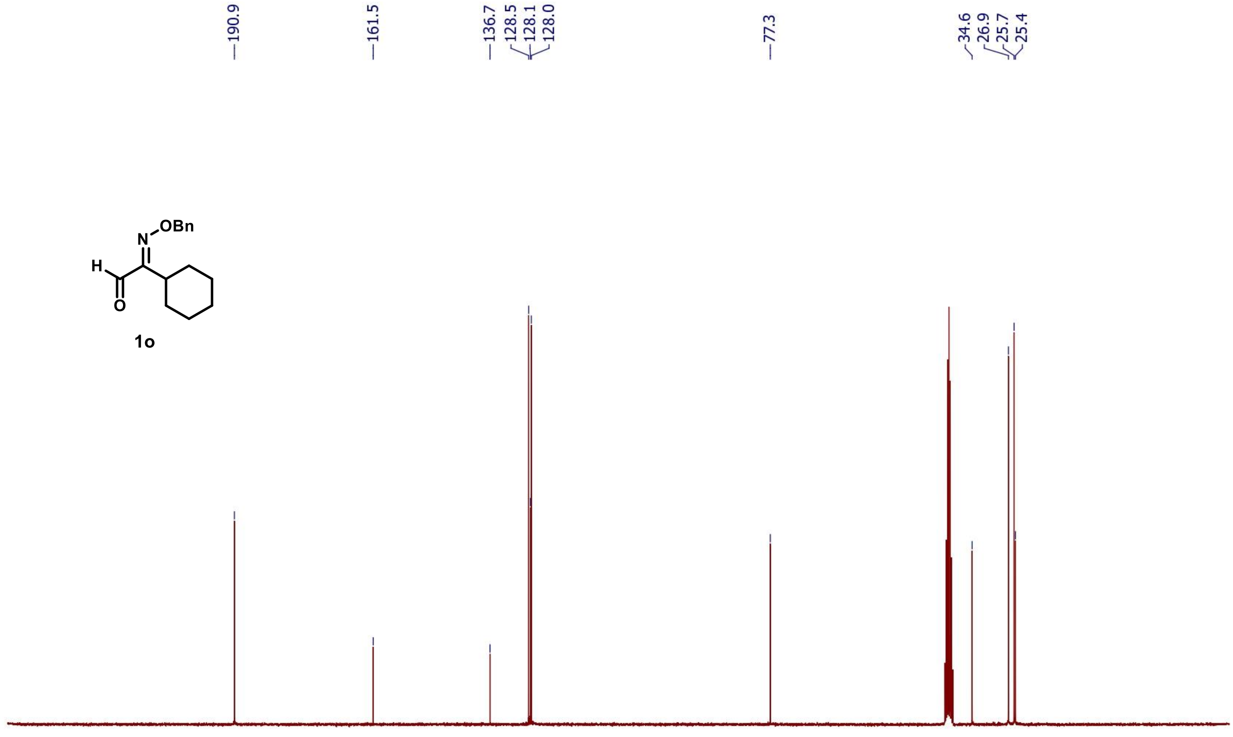

^{1}H-NMR (300 MHz, DMSO-d_6)

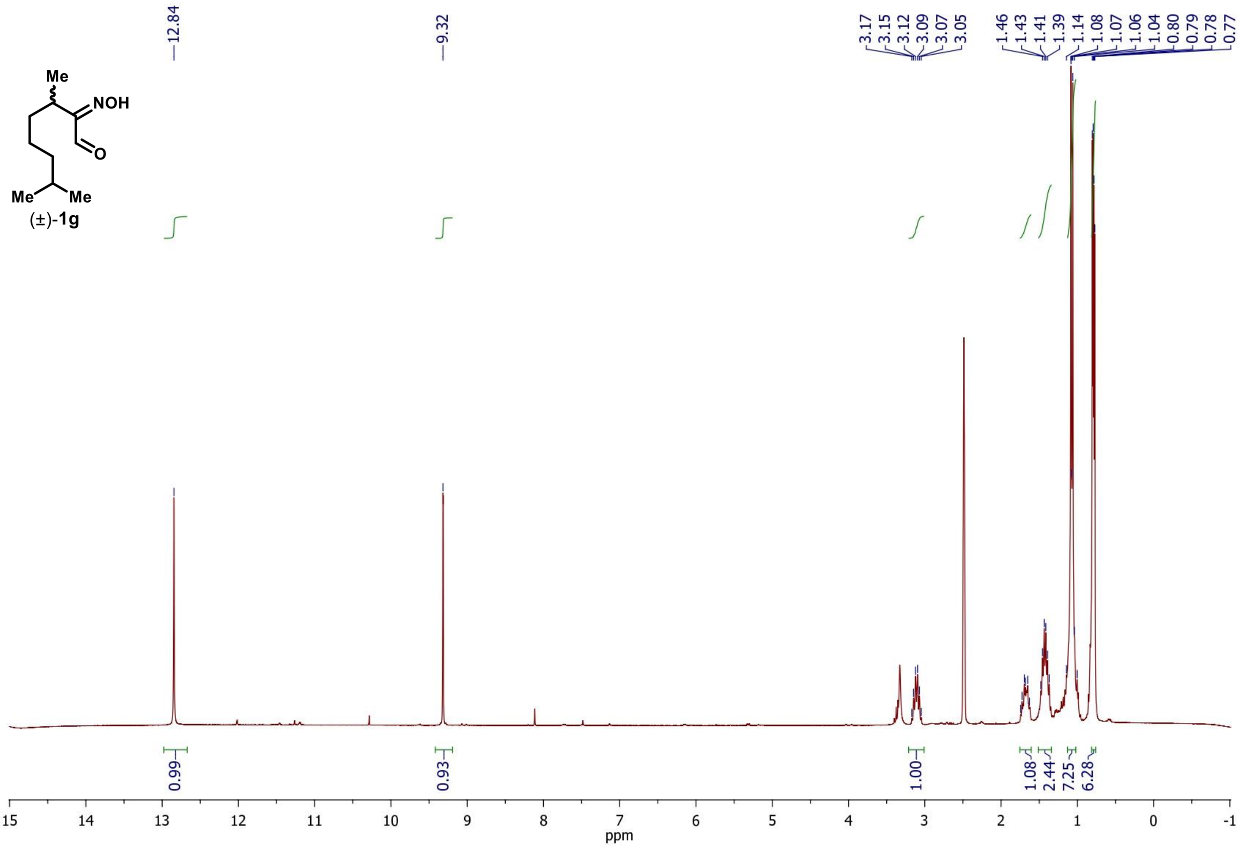

^{13}C{^{1}H}-NMR (75 MHz, DMSO-d_6)

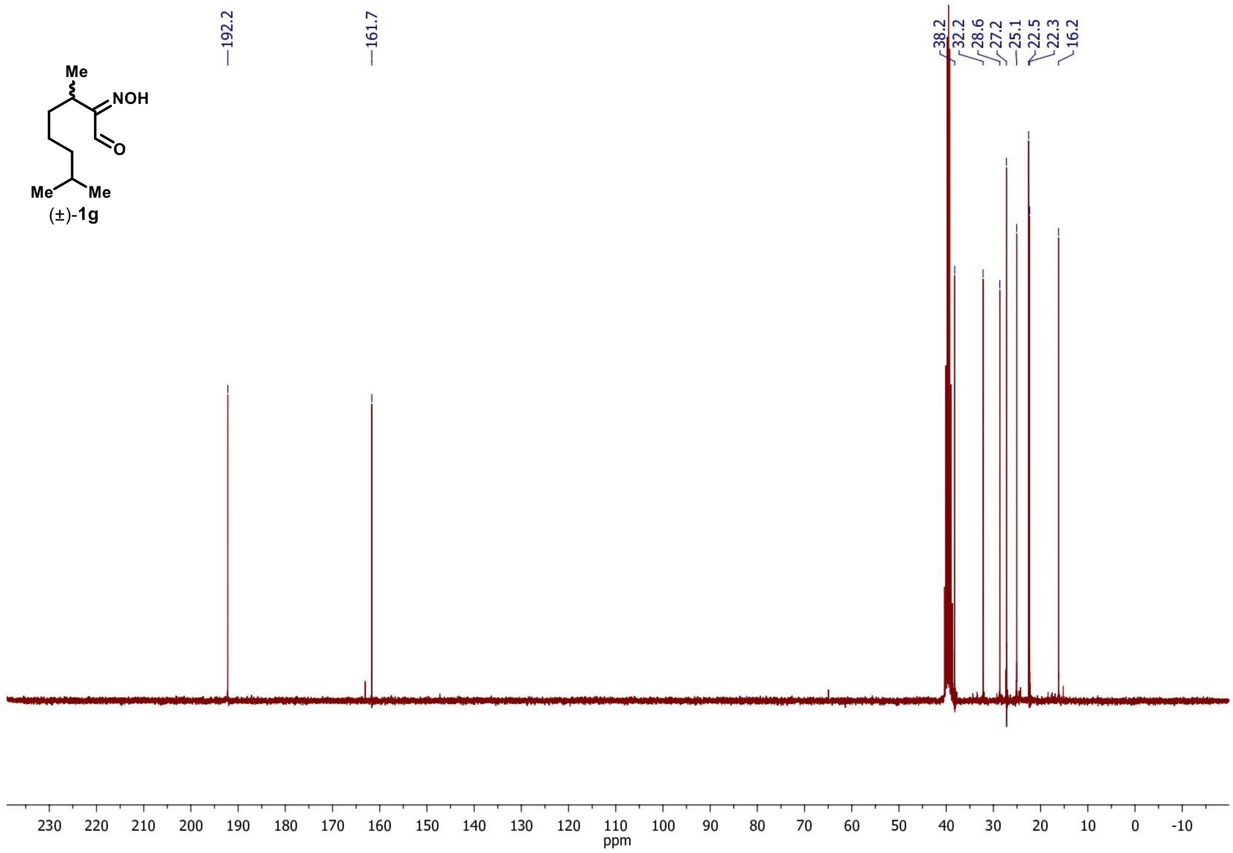

^{1}H-NMR (300 MHz, DMSO-d_6)

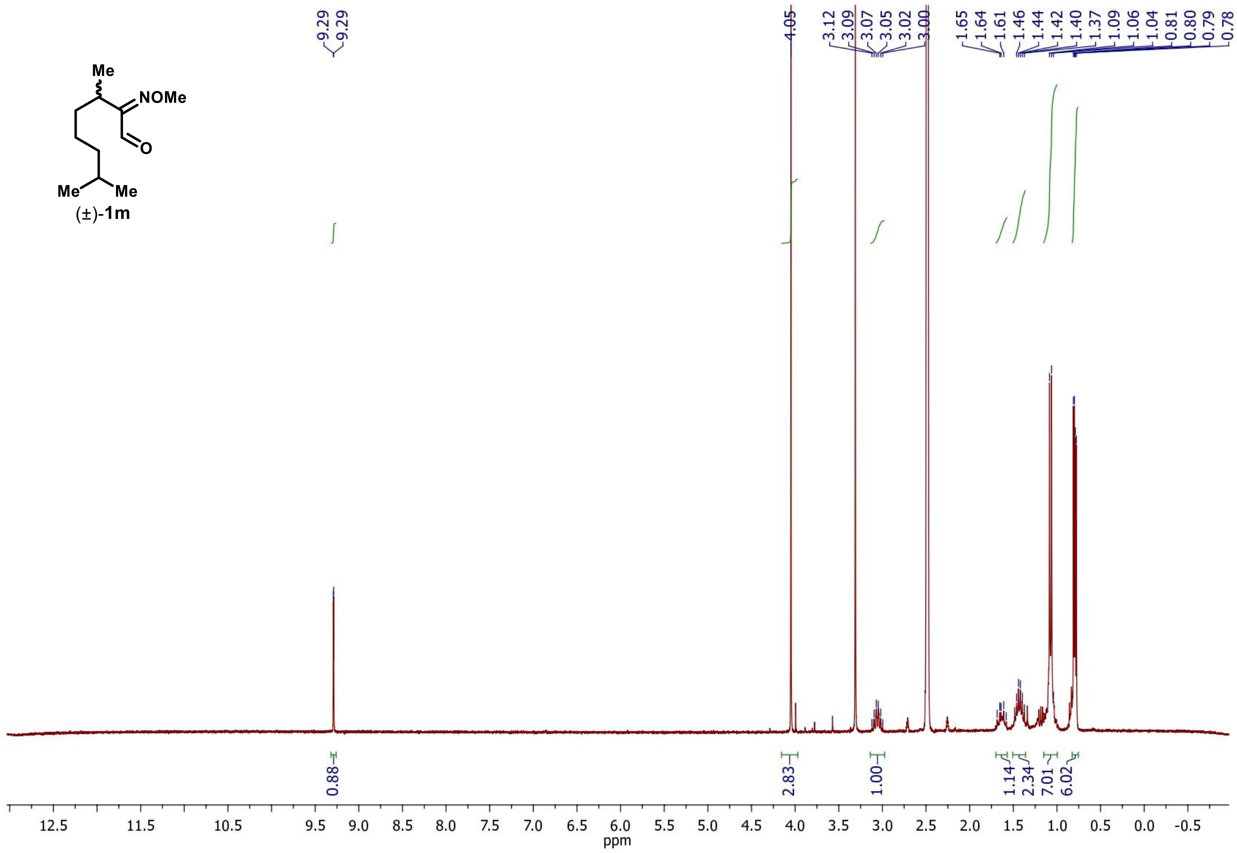

^{13}C{^{1}H}-NMR (75 MHz, DMSO-d_6)

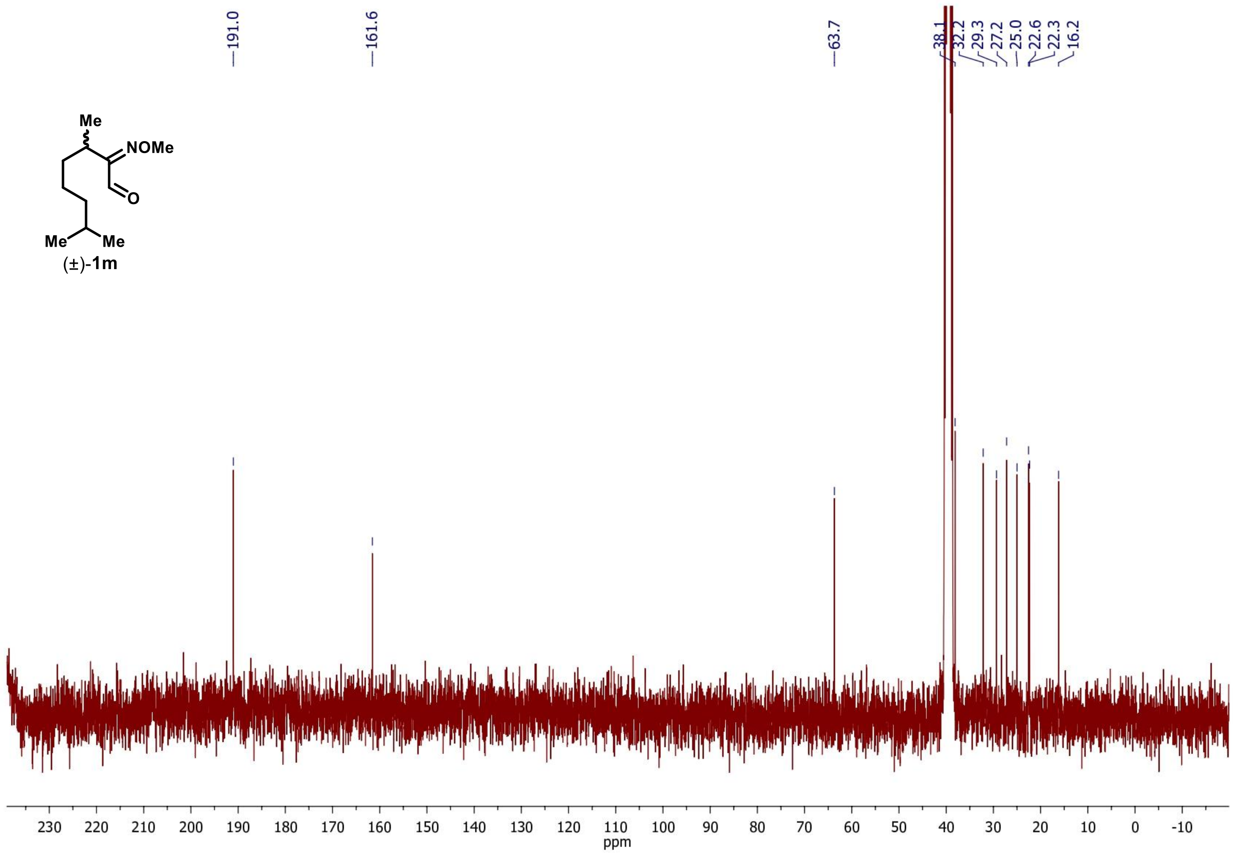

^{1}H-NMR (300 MHz, DMSO-d_6)

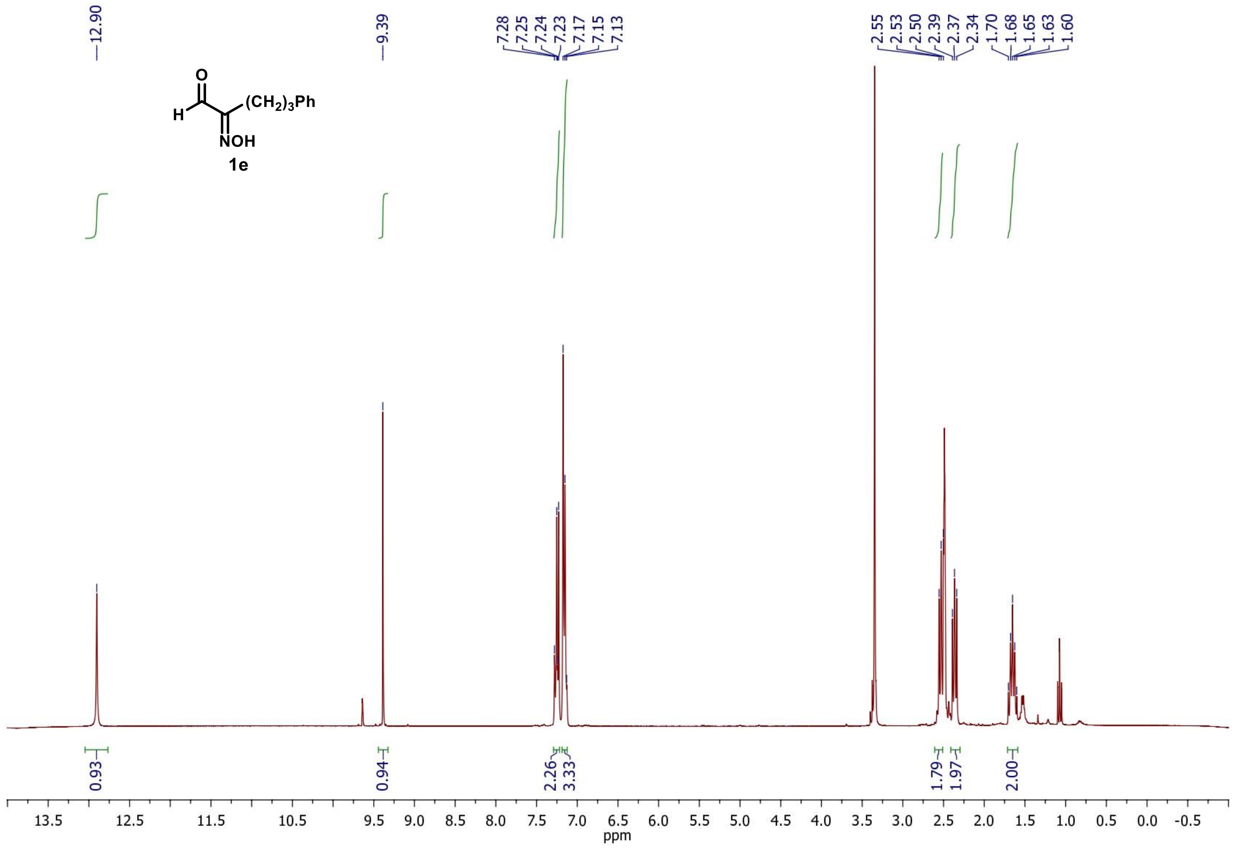

^{13}C{^{1}H}-NMR (75 MHz, DMSO-d_6)

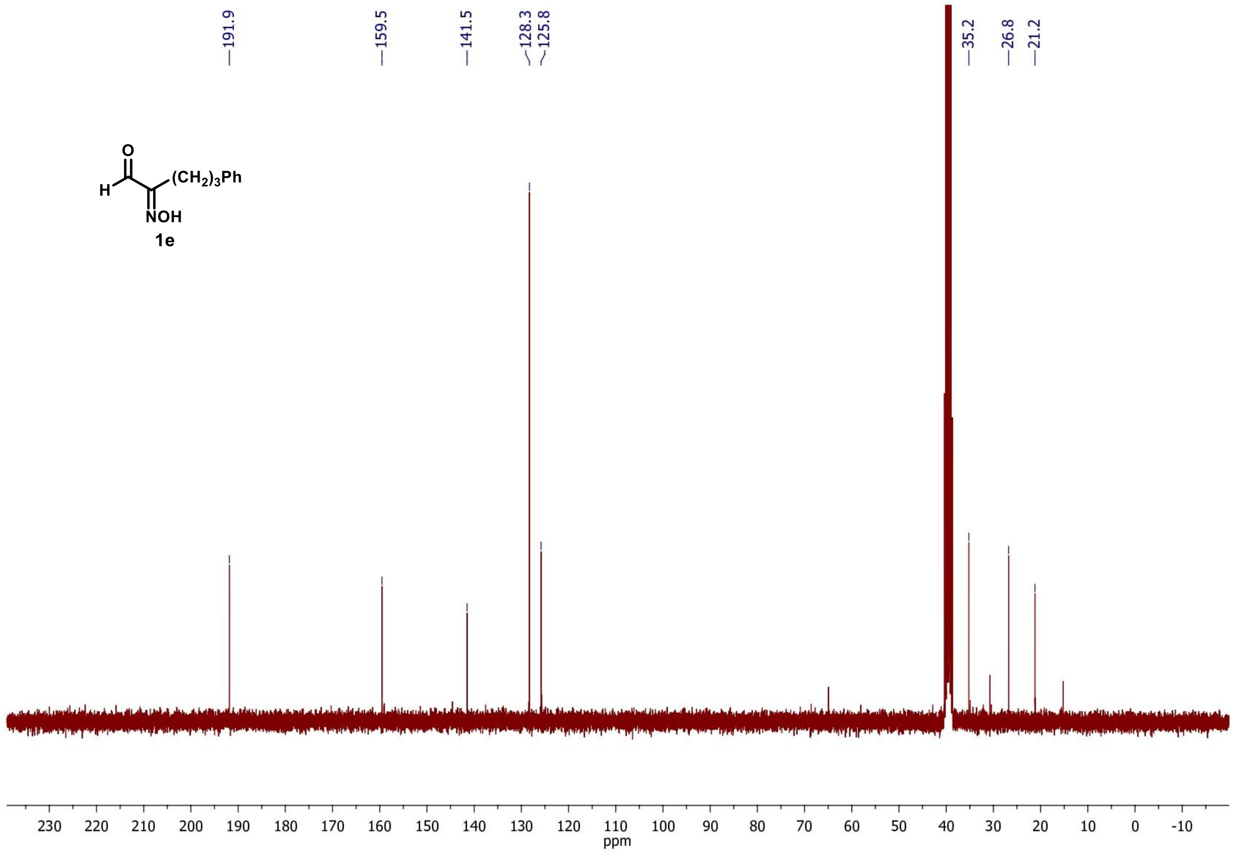

^{1}H-NMR (300 MHz, DMSO-d_6)

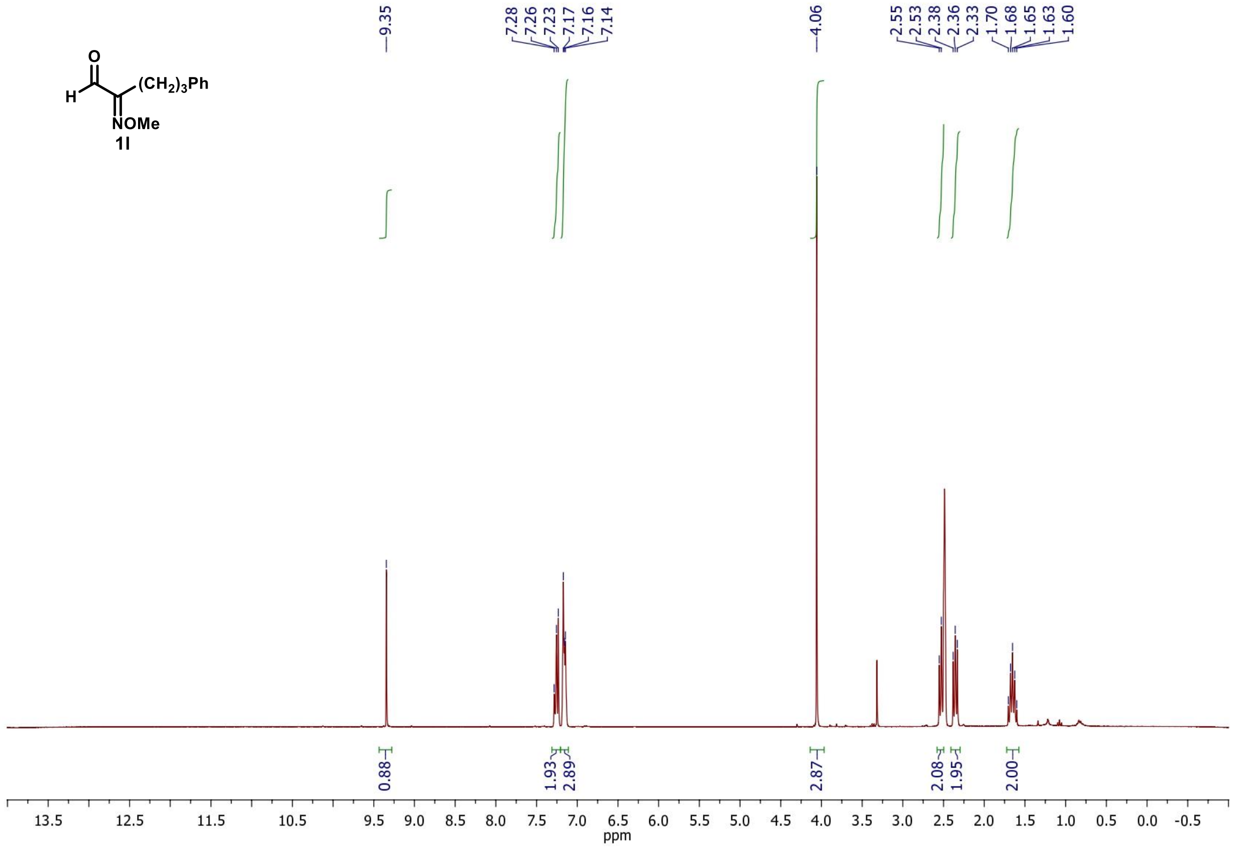

^{13}C{^{1}H}-NMR (75 MHz, DMSO-d_6)

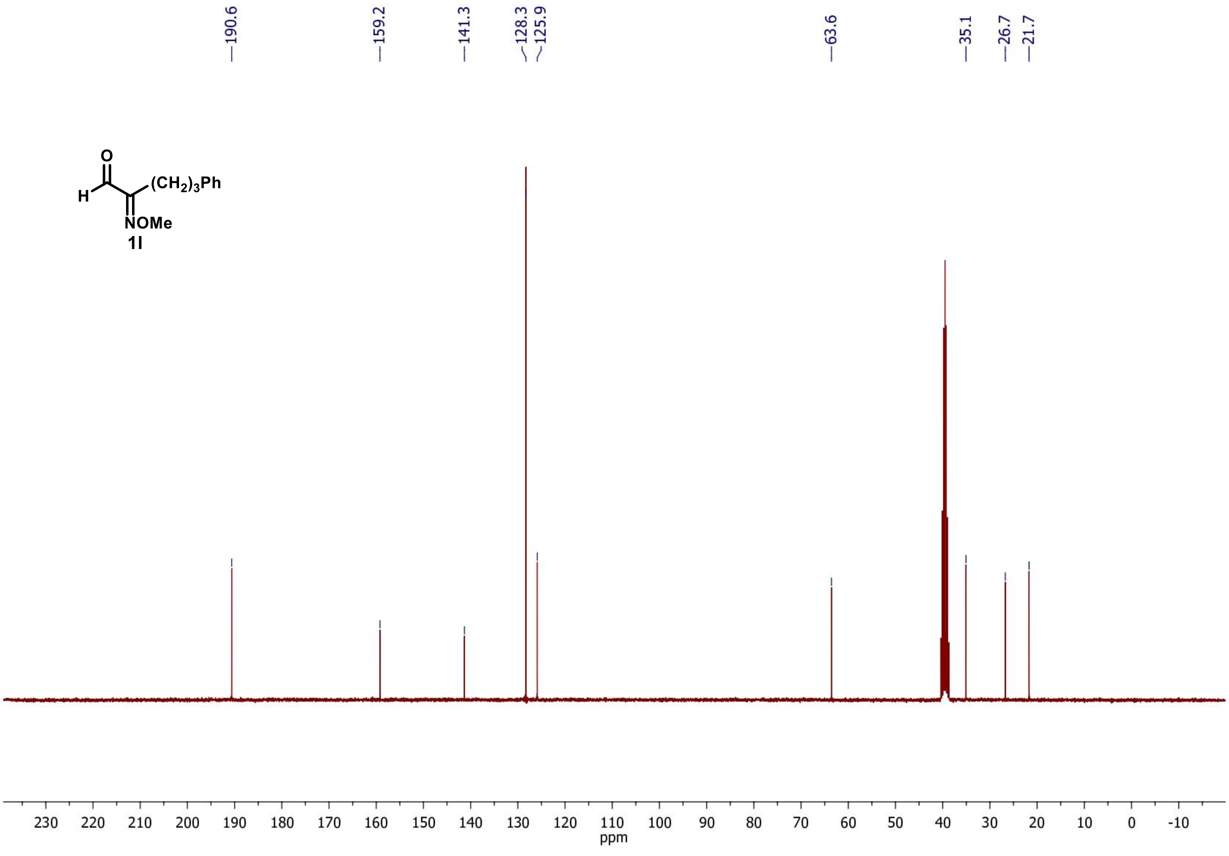

^{1}H-NMR (300 MHz, DMSO-d_6)

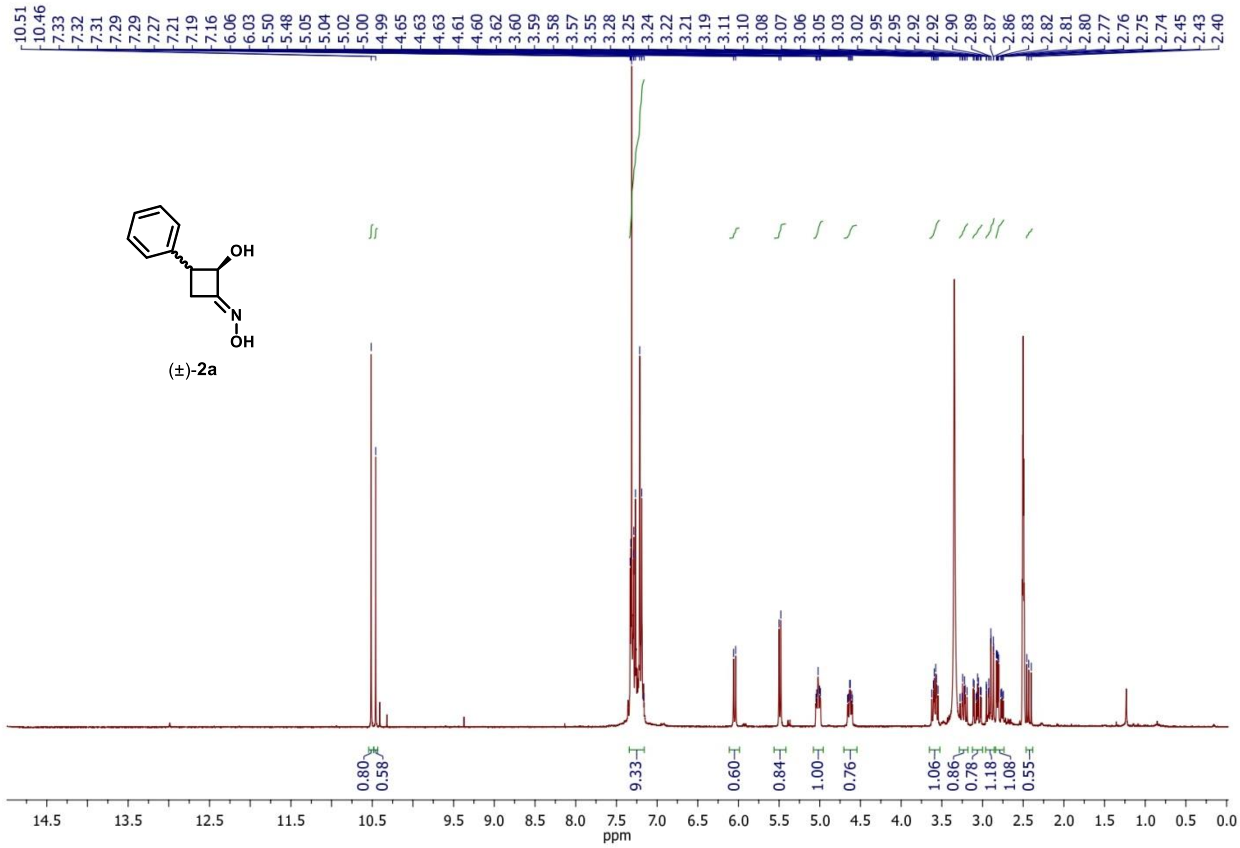

^{13}C{^{1}H}-NMR (75 MHz, DMSO-d_6)

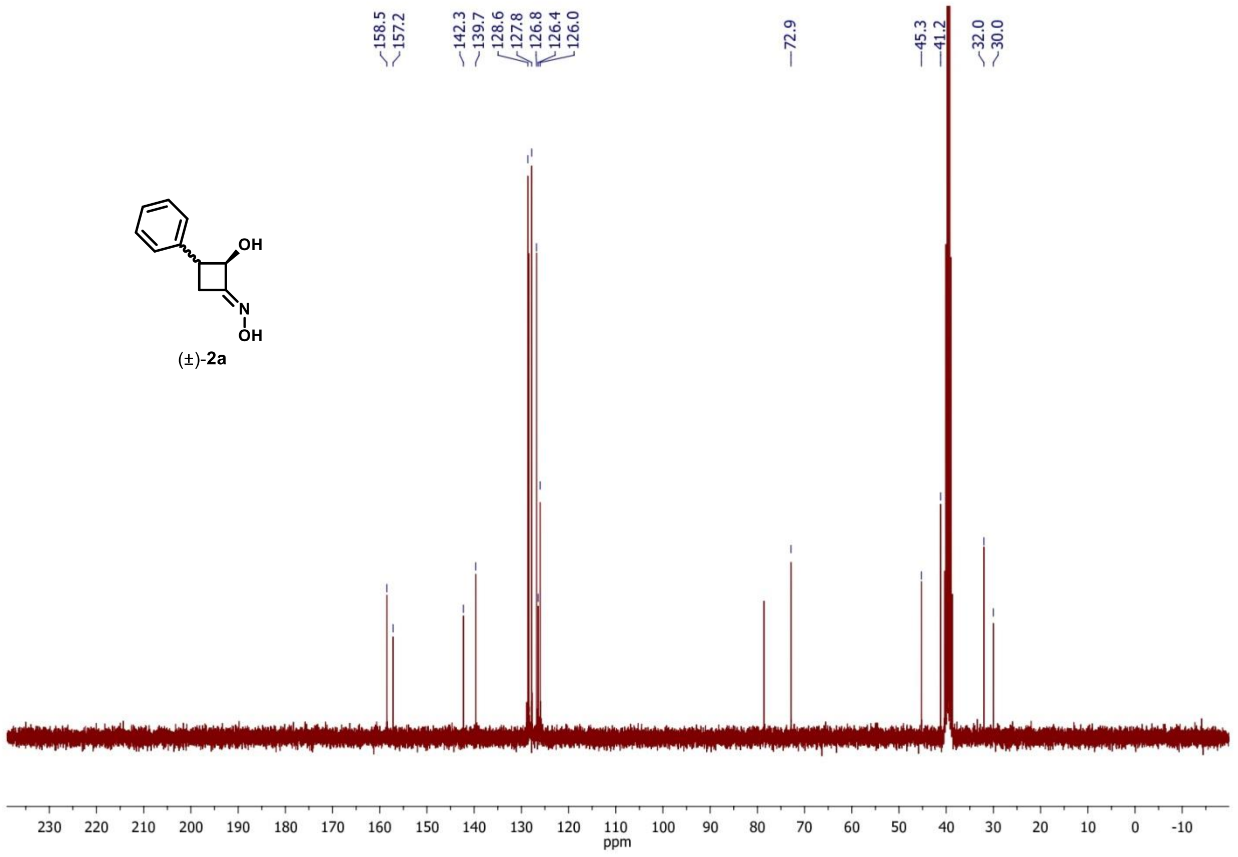

NOESY (300 MHz, DMSO-d_6)

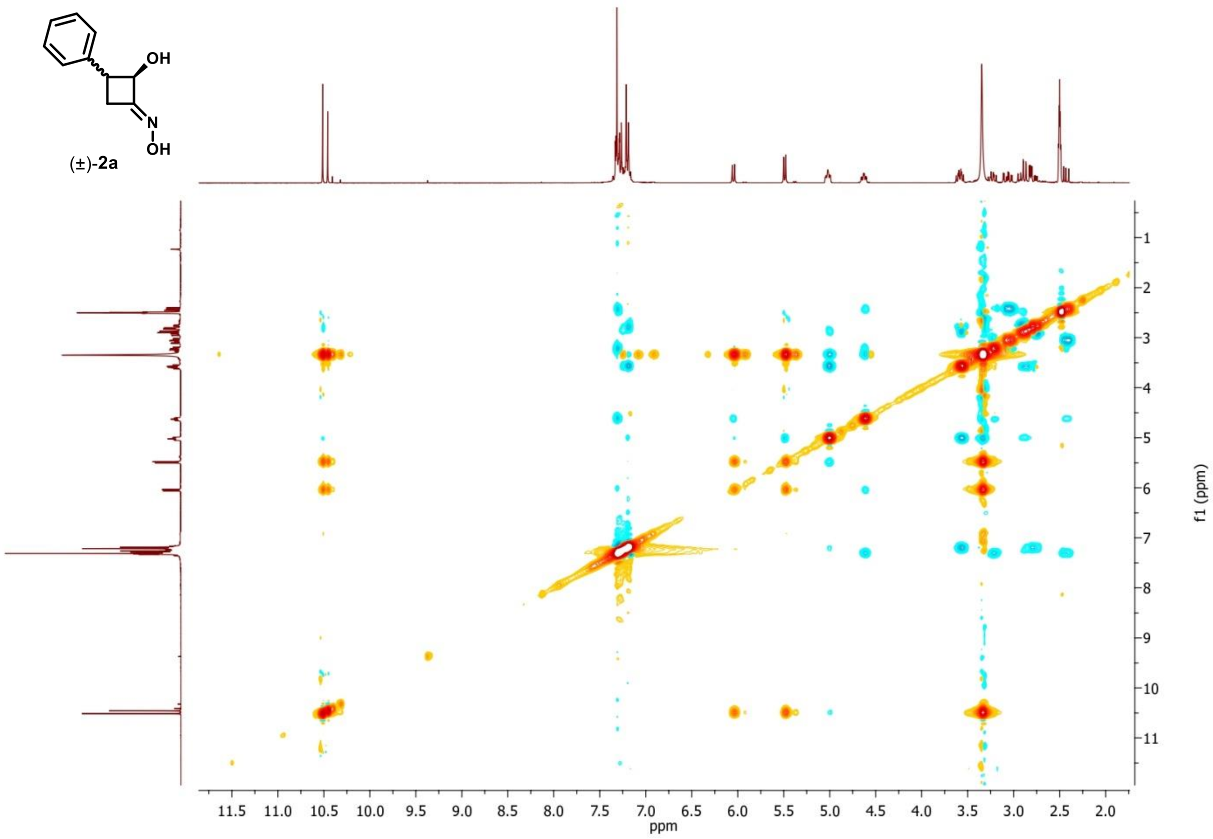

HSQC (300 MHz, DMSO-d_6)

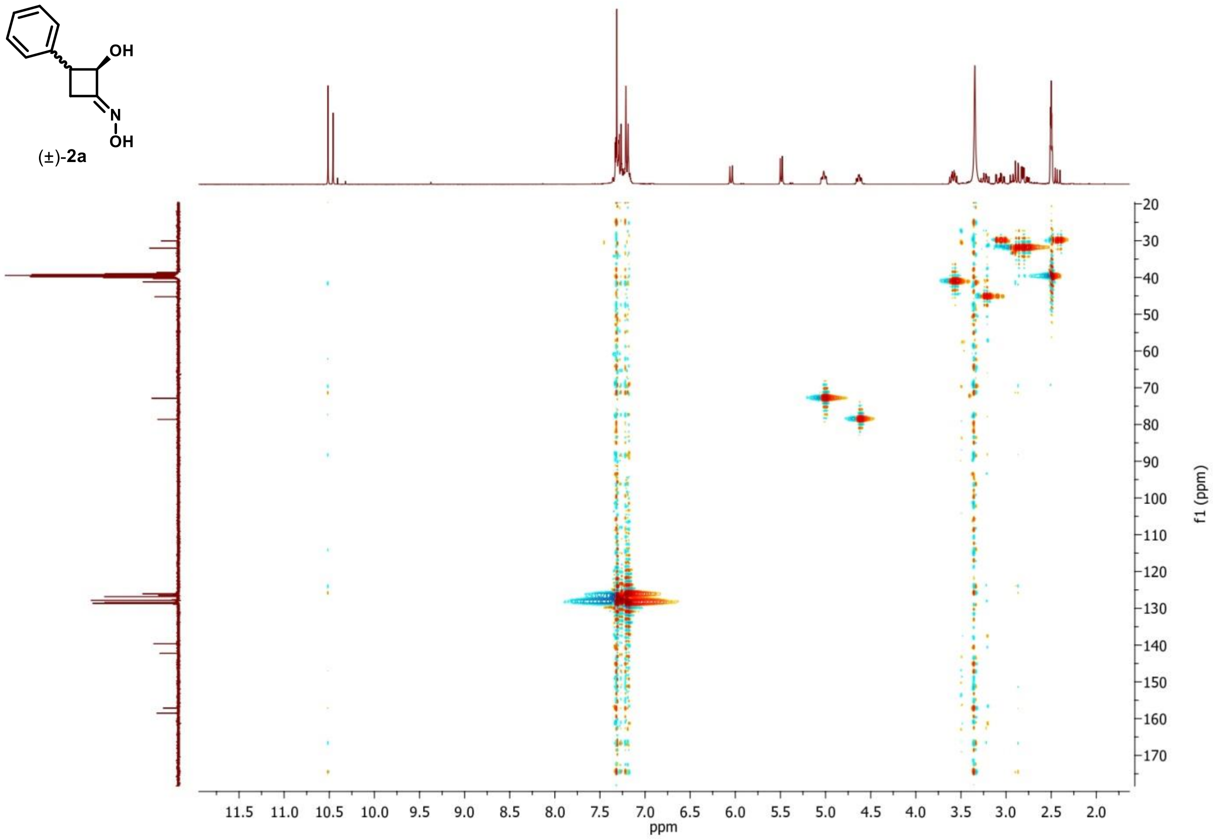

^{1}H-NMR (300 MHz, DMSO-d_6)

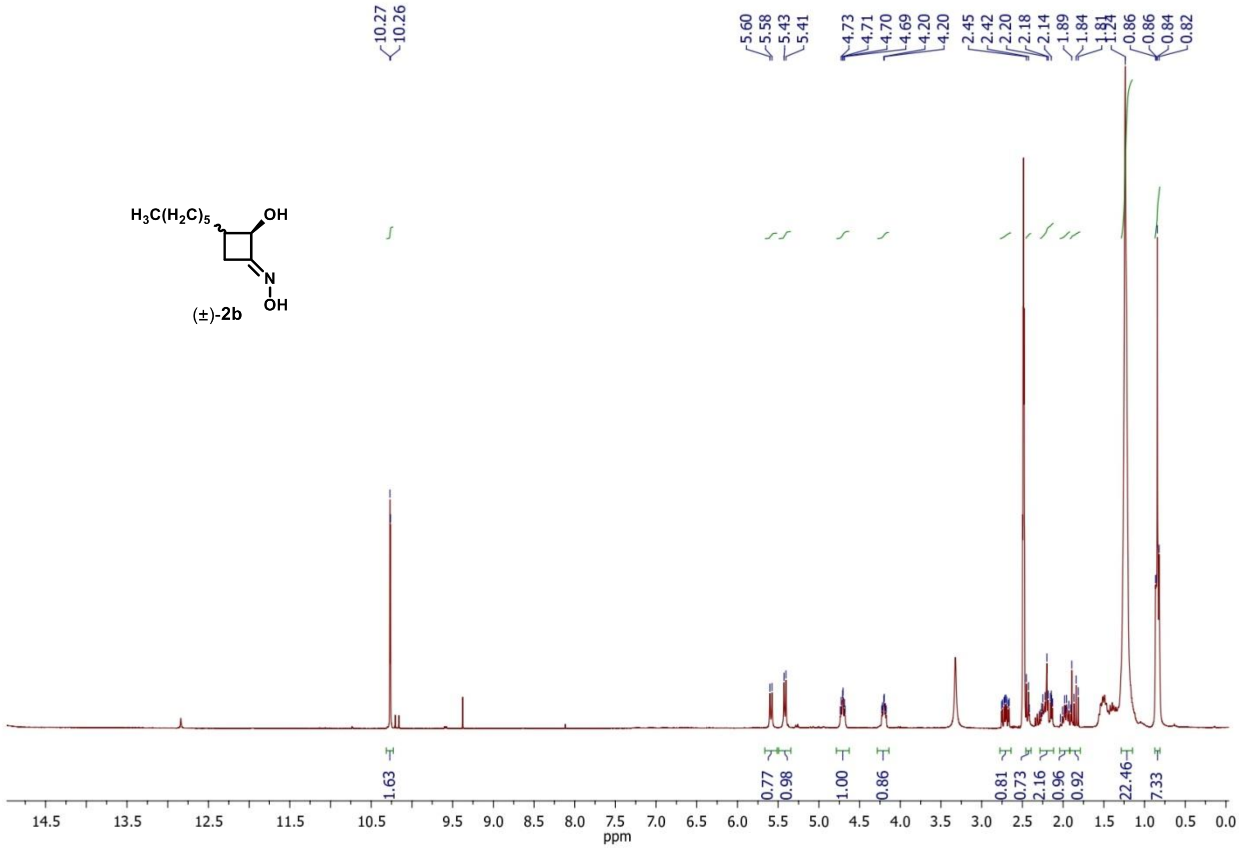

$^{13}C\{^{1}H\}$-NMR (75 MHz, DMSO-d_6)

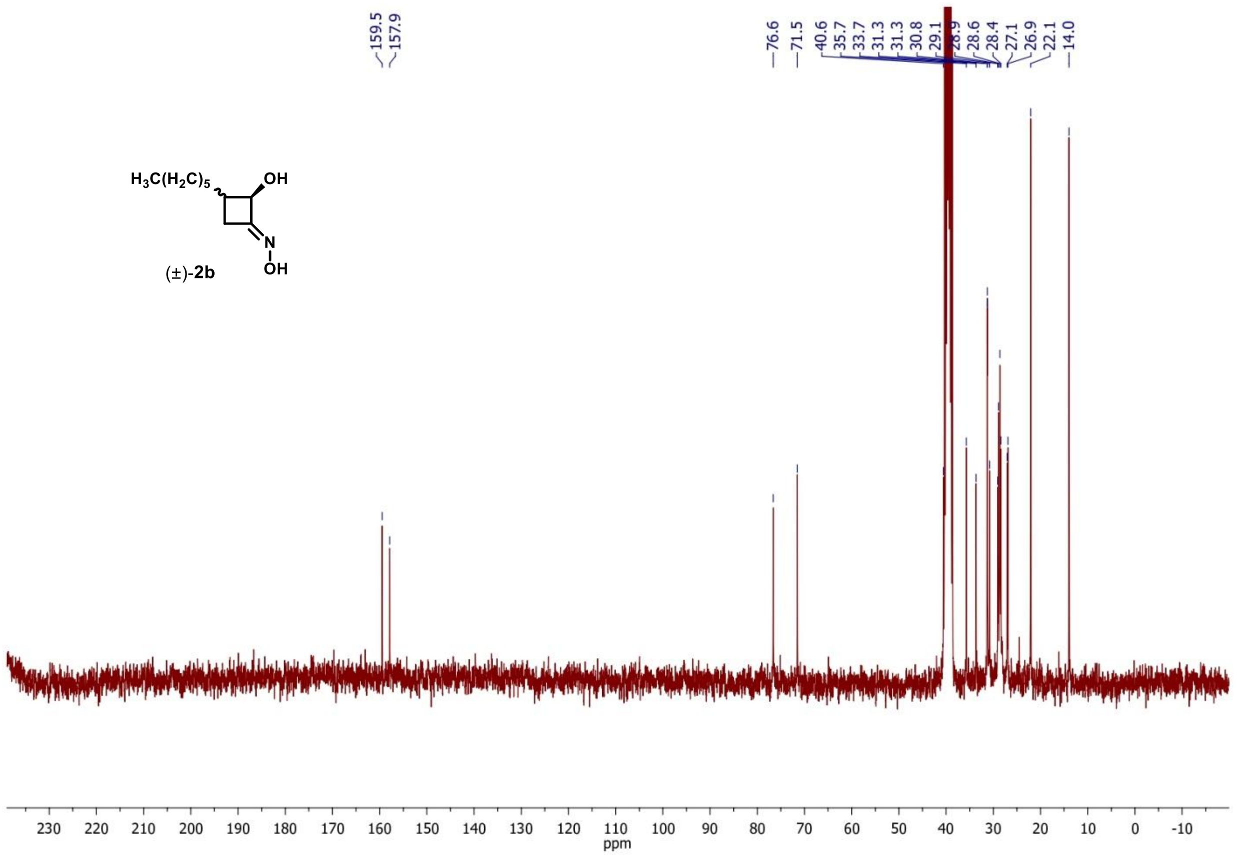

^{1}H-NMR (300 MHz, DMSO-d_6)

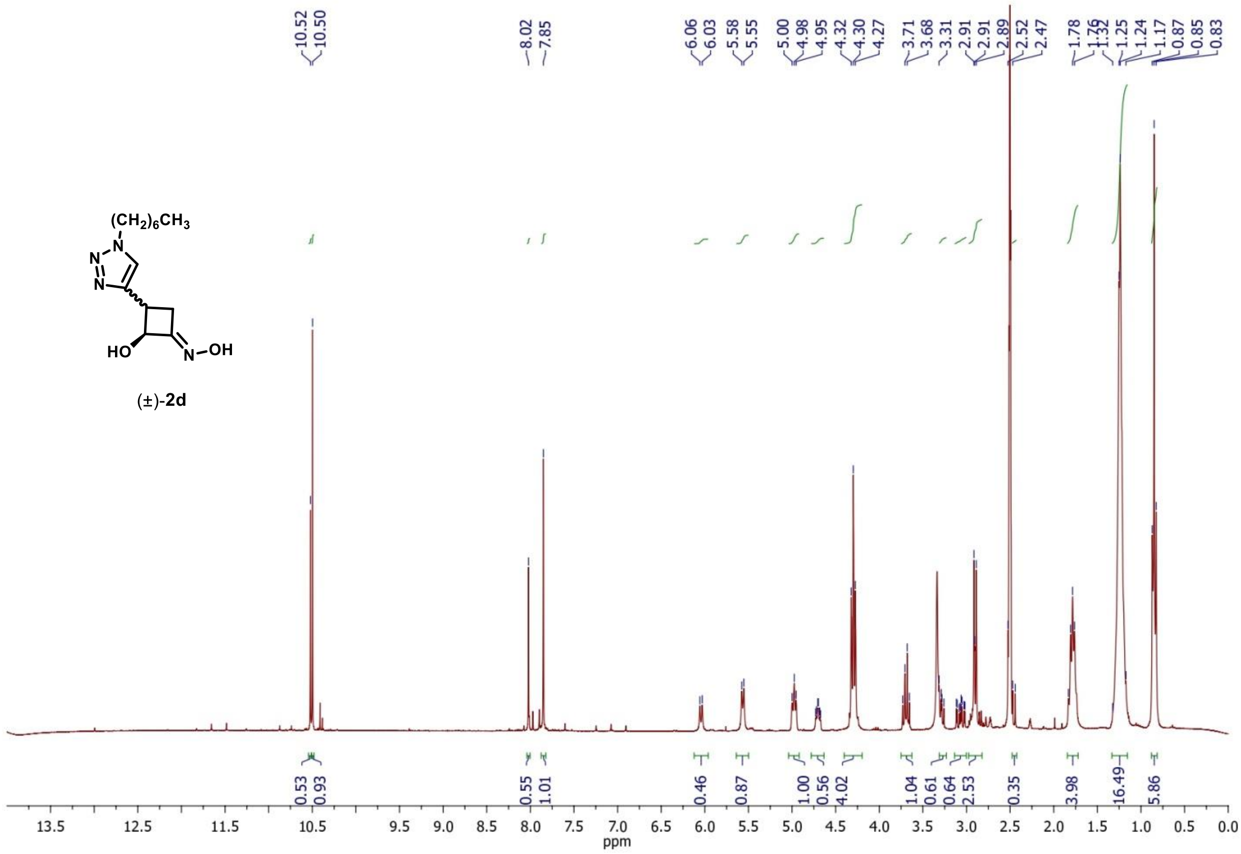

$^{13}C\{^{1}H\}$-NMR (75 MHz, DMSO-d_6)

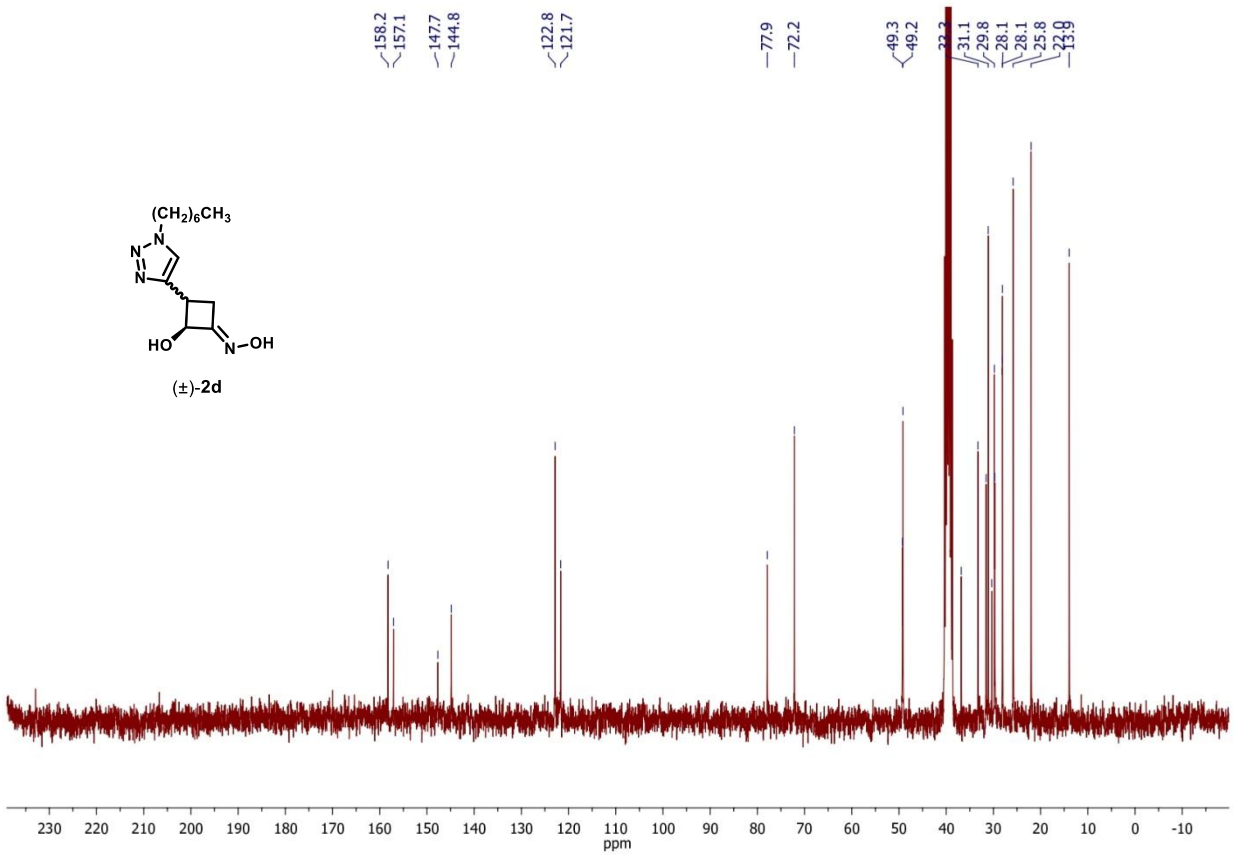

DEPT-135 (75 MHz, DMSO-d_6)

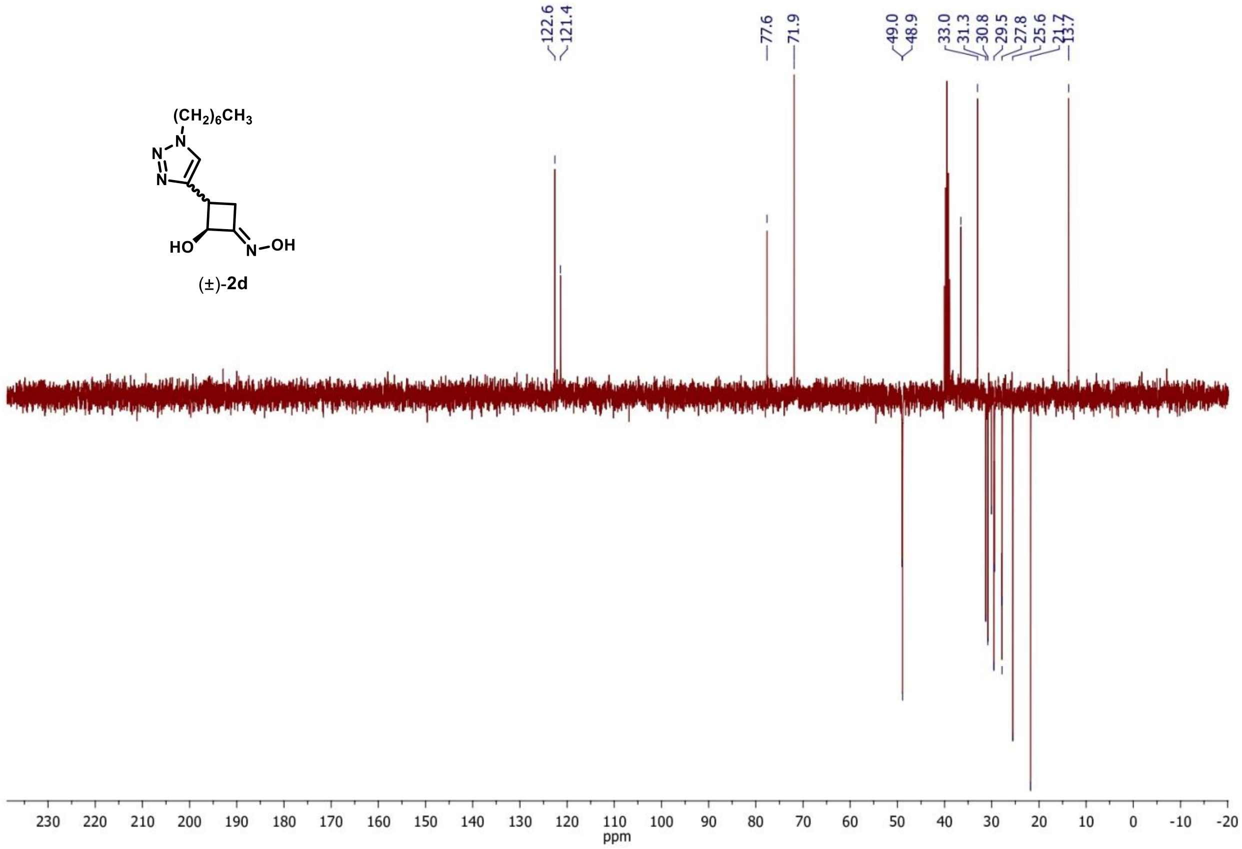

COSY-45 (300 MHz, DMSO-d_6)

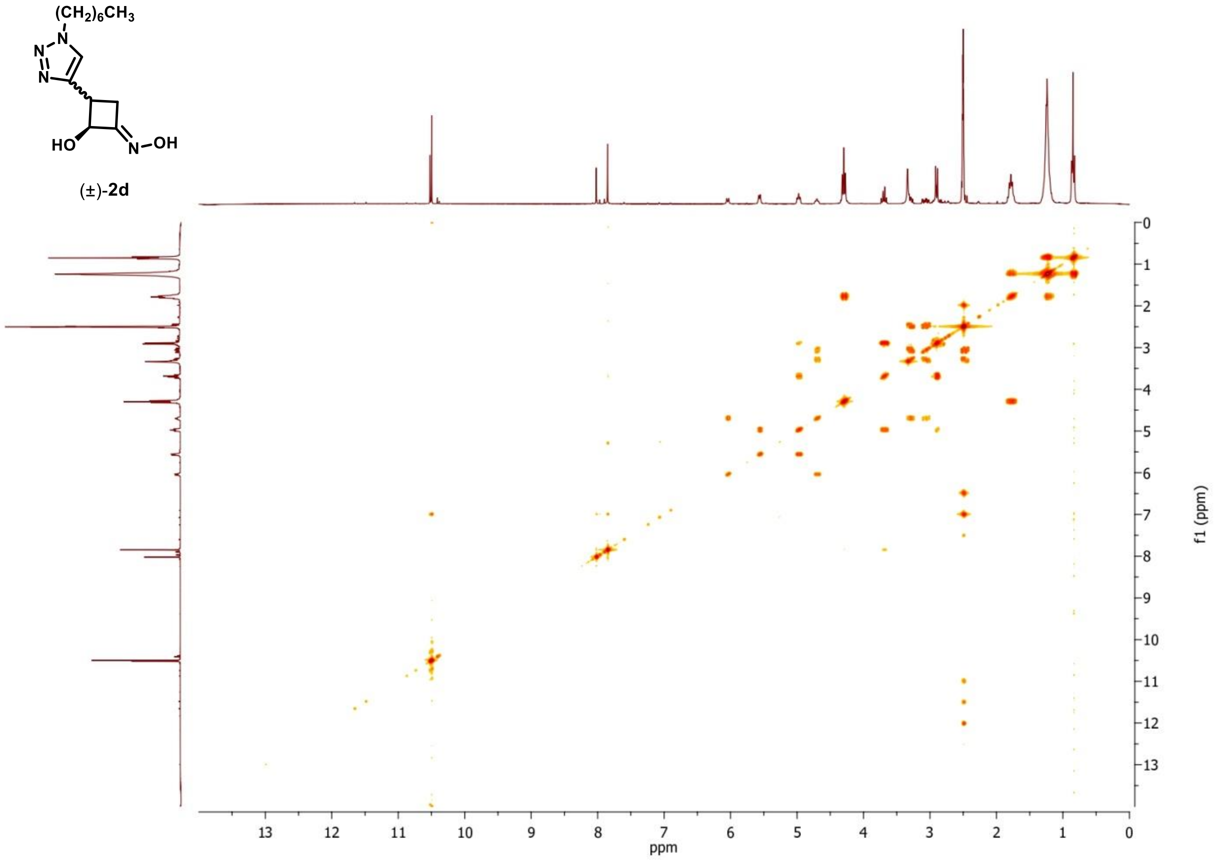

HSQC (300 MHz, DMSO-d_6)

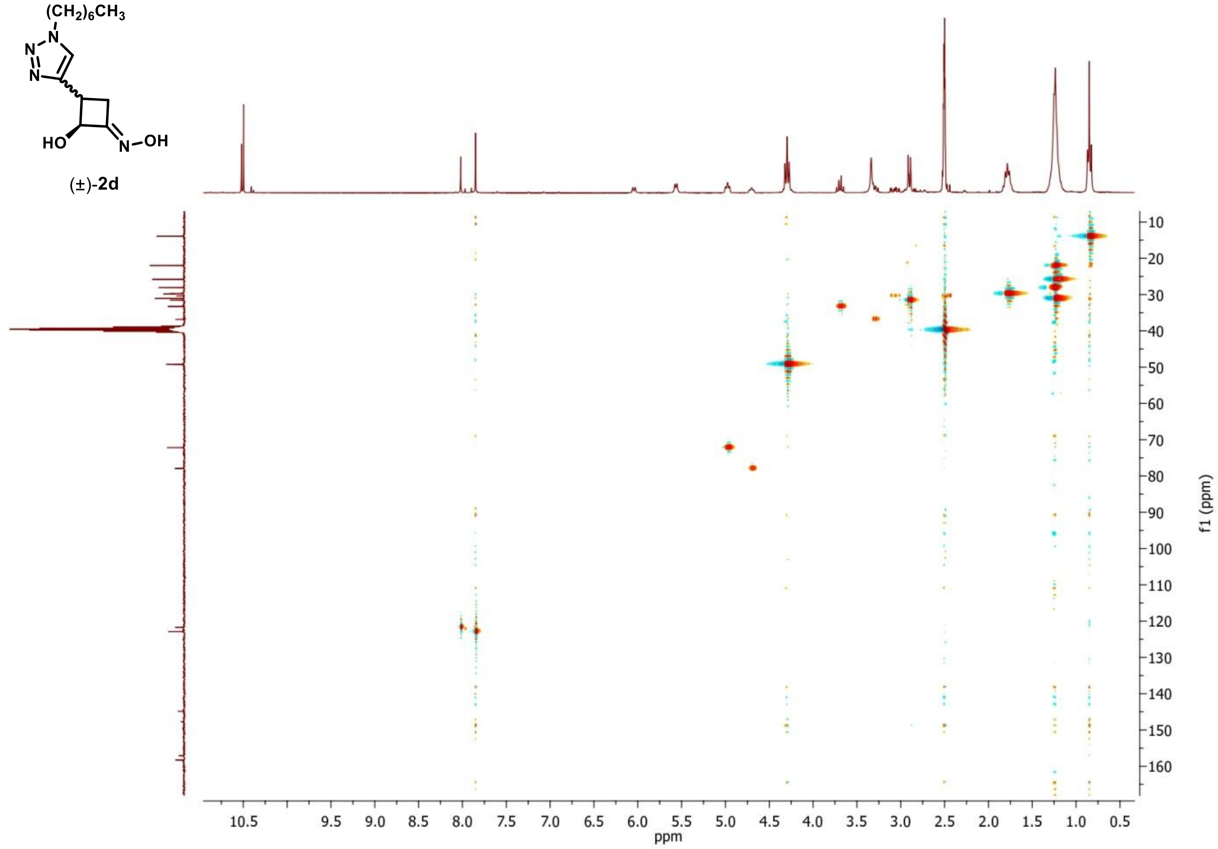

^{1}H-NMR (300 MHz, DMSO-d_6)

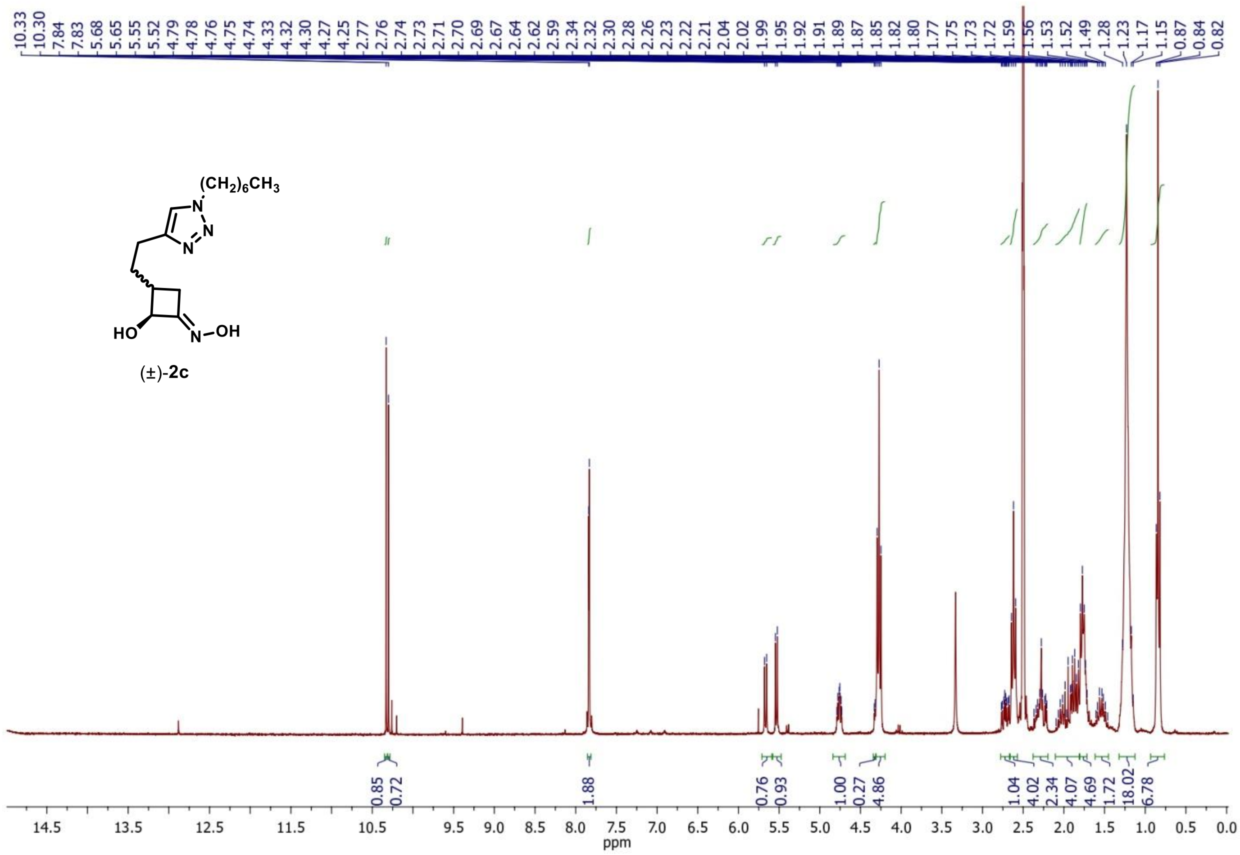

$^{13}C\{^{1}H\}$-NMR (75 MHz, DMSO-d_6)

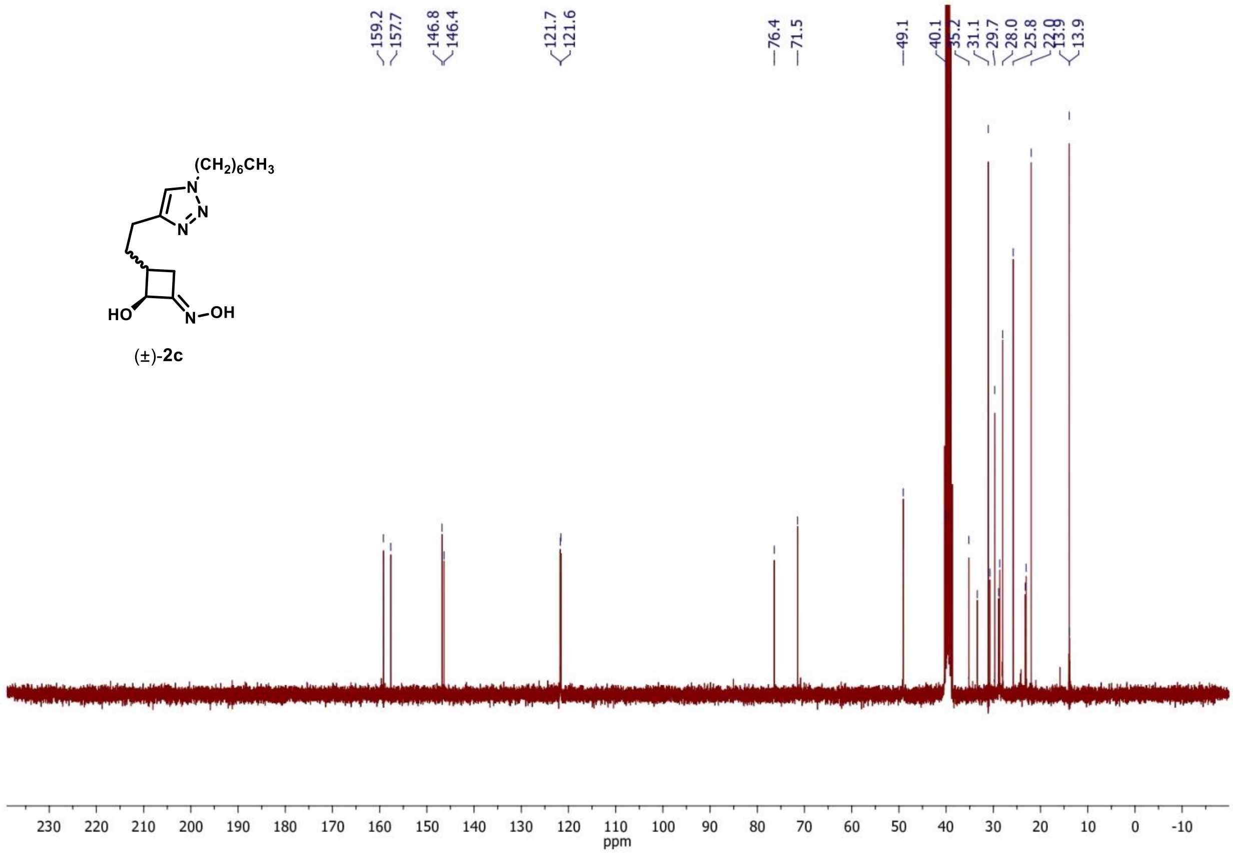

DEPT-135 (75 MHz, DMSO-d_6)

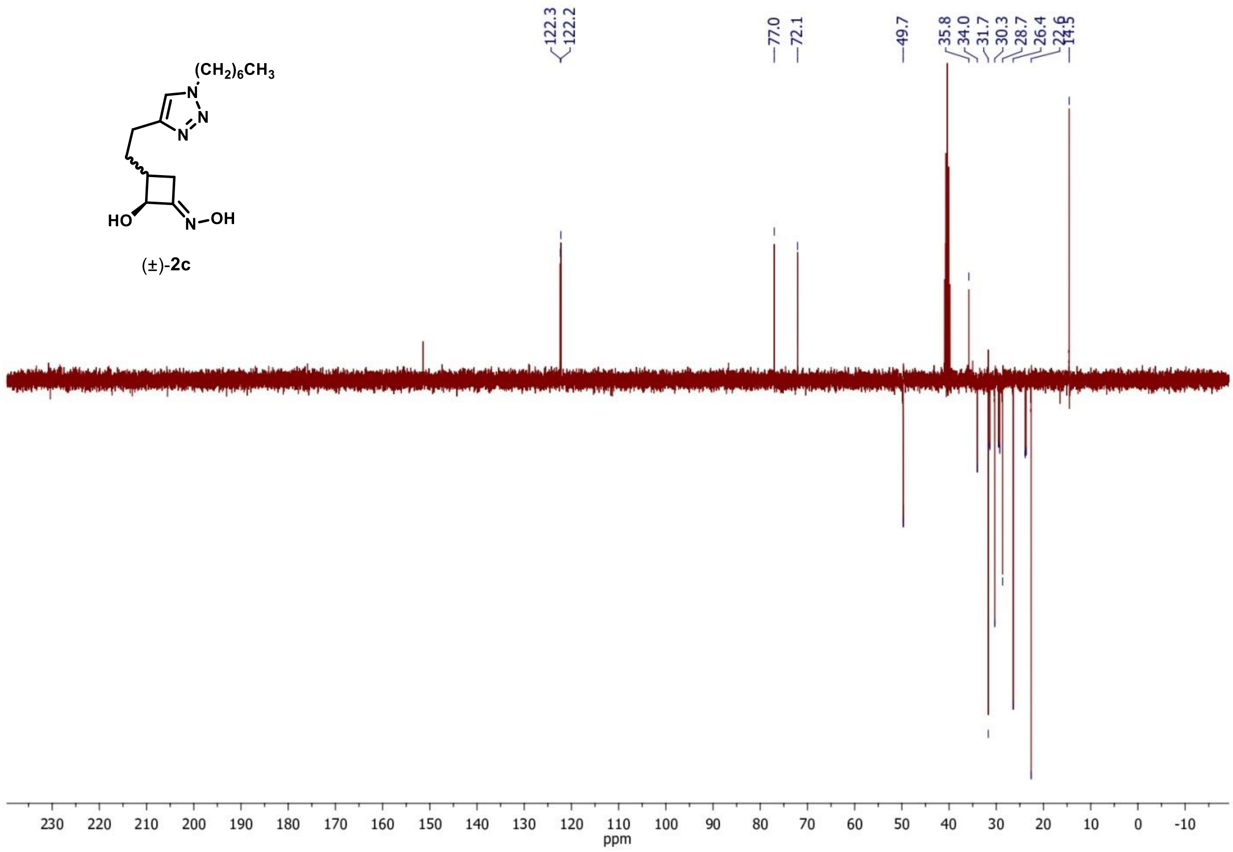

HSQC (300 MHz, DMSO-d_6)

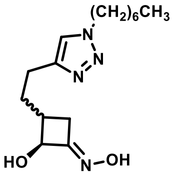

(±)-**2c**

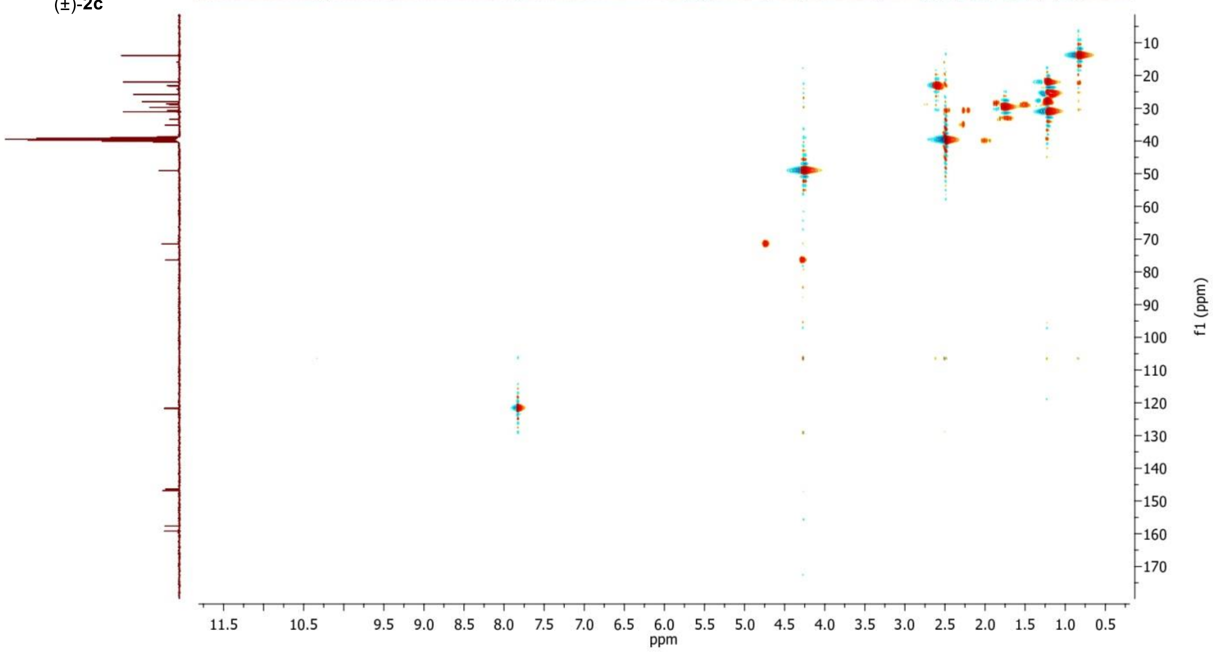

HMBC (300 MHz, DMSO-d_6)

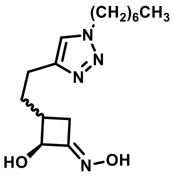

(±)-**2c**

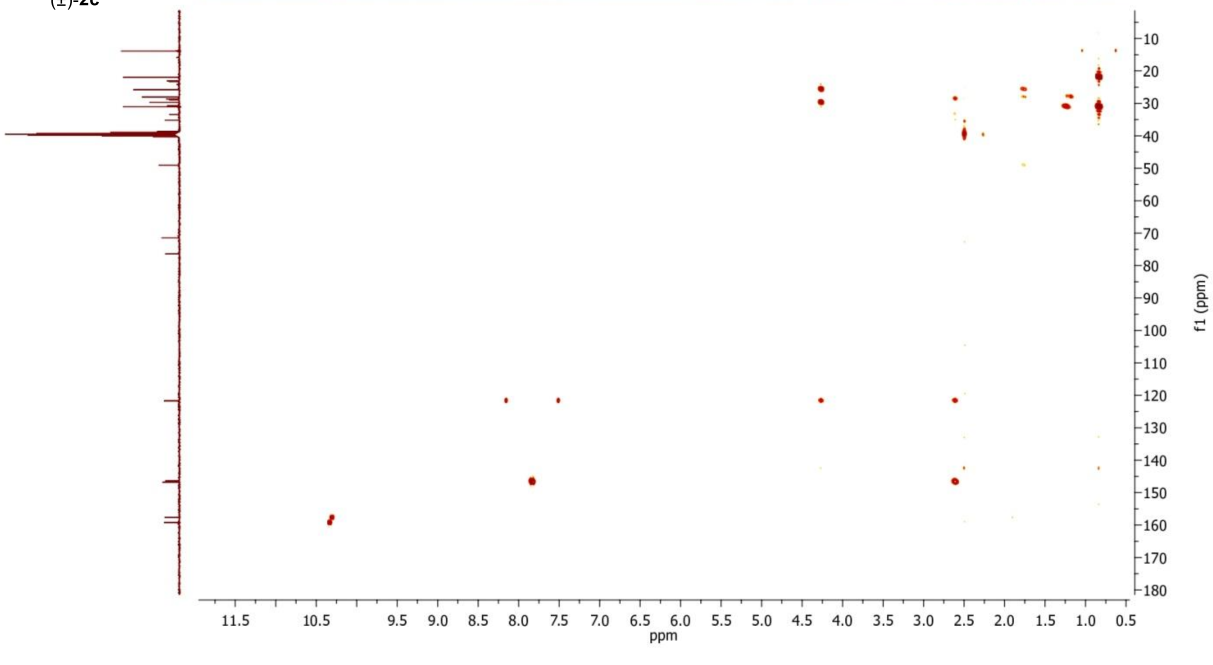

^{1}H-NMR (300 MHz, DMSO-d_6)

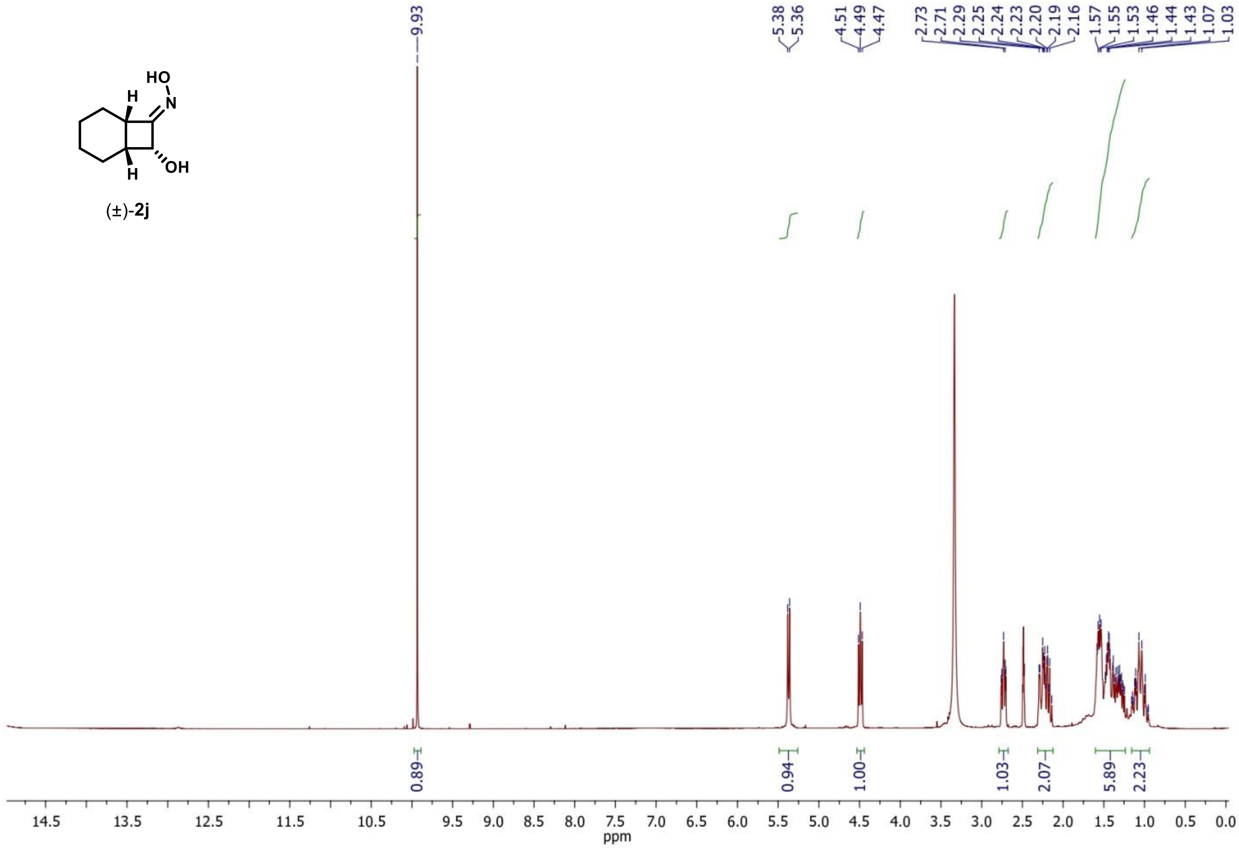

^{1}H-NMR (300 MHz, DMSO-d_6)

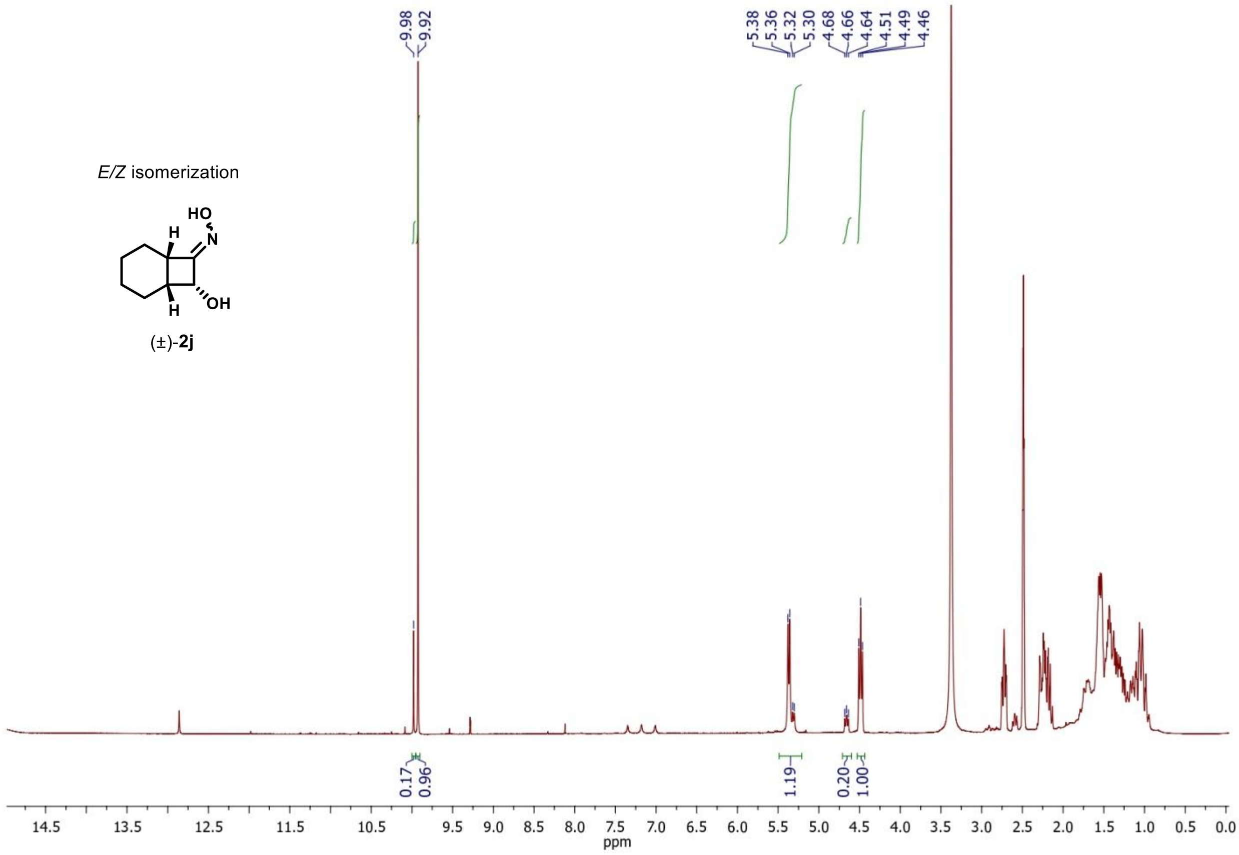

$^{13}C\{^{1}H\}$-NMR (75 MHz, DMSO-d_6)

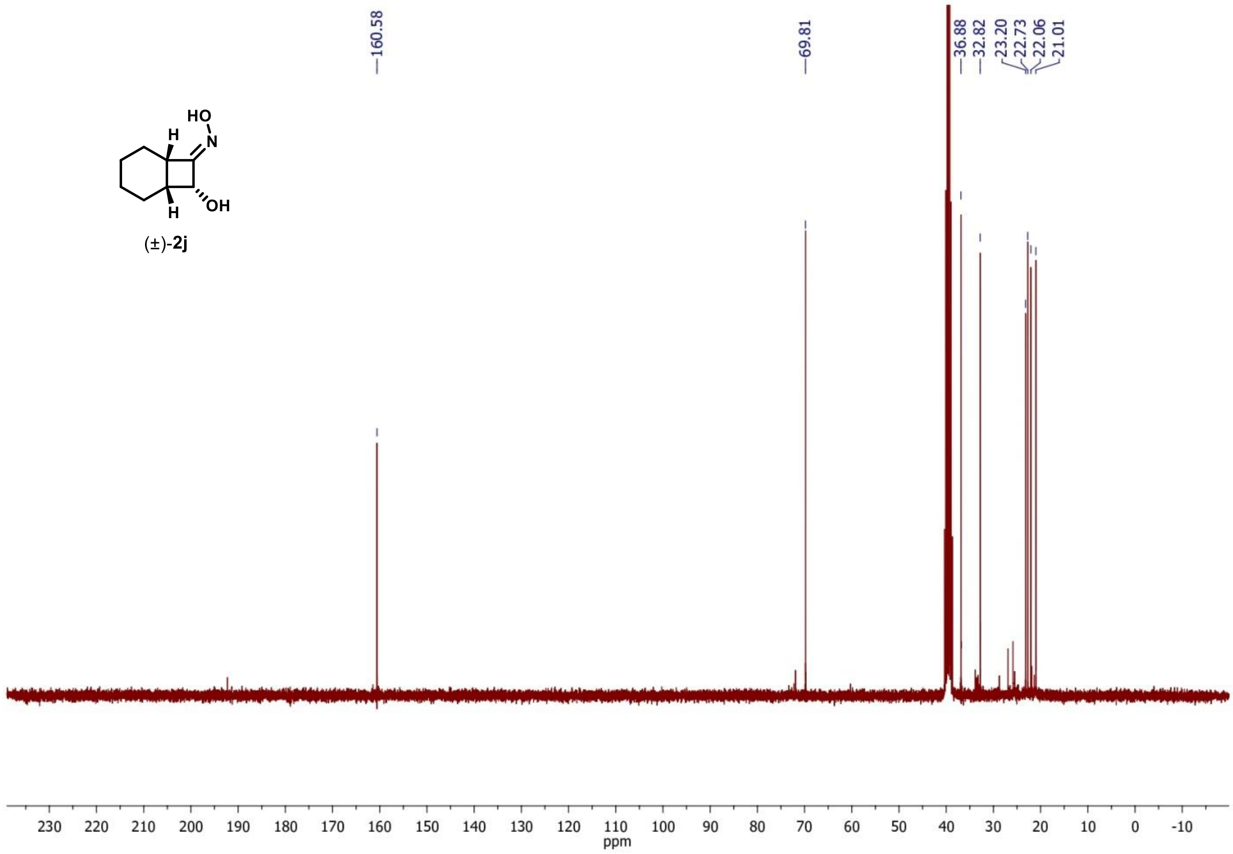

NOESY (300 MHz, DMSO-d_6)

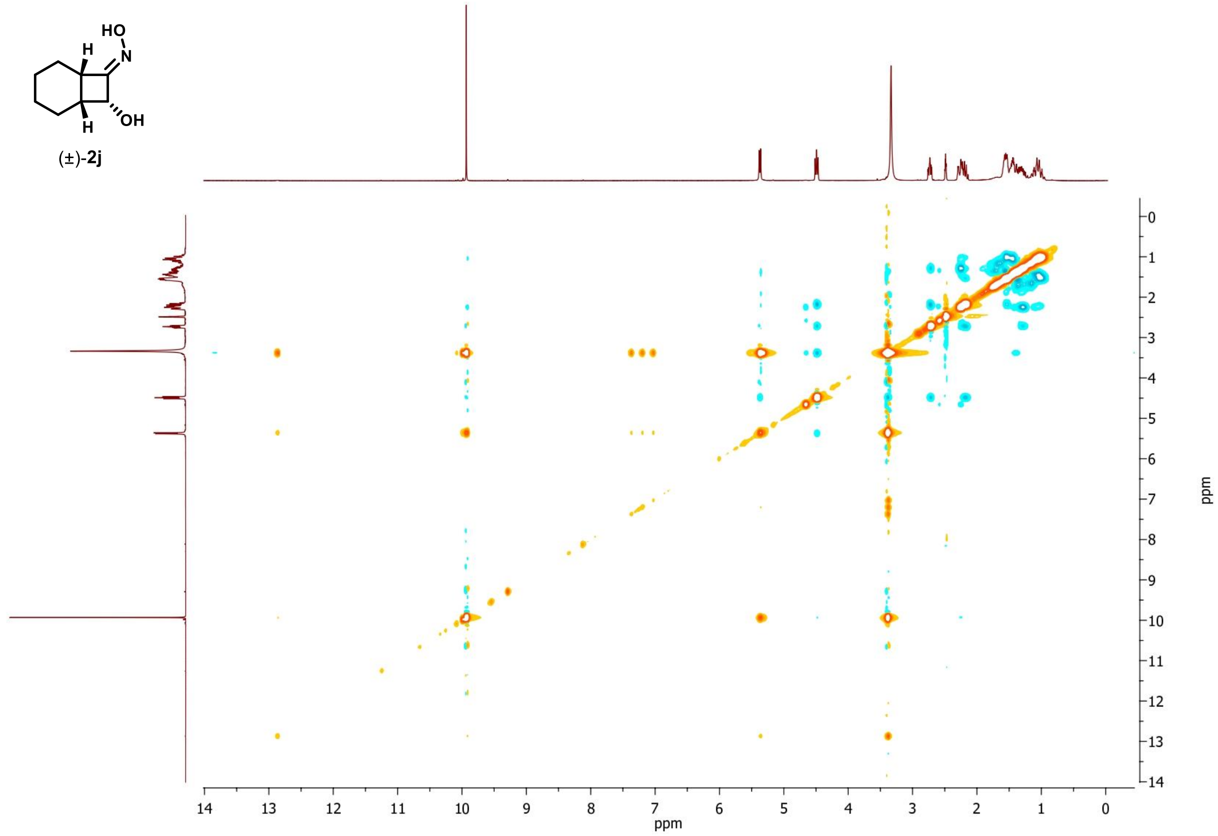

^{1}H-NMR (300 MHz, DMSO-d_6)

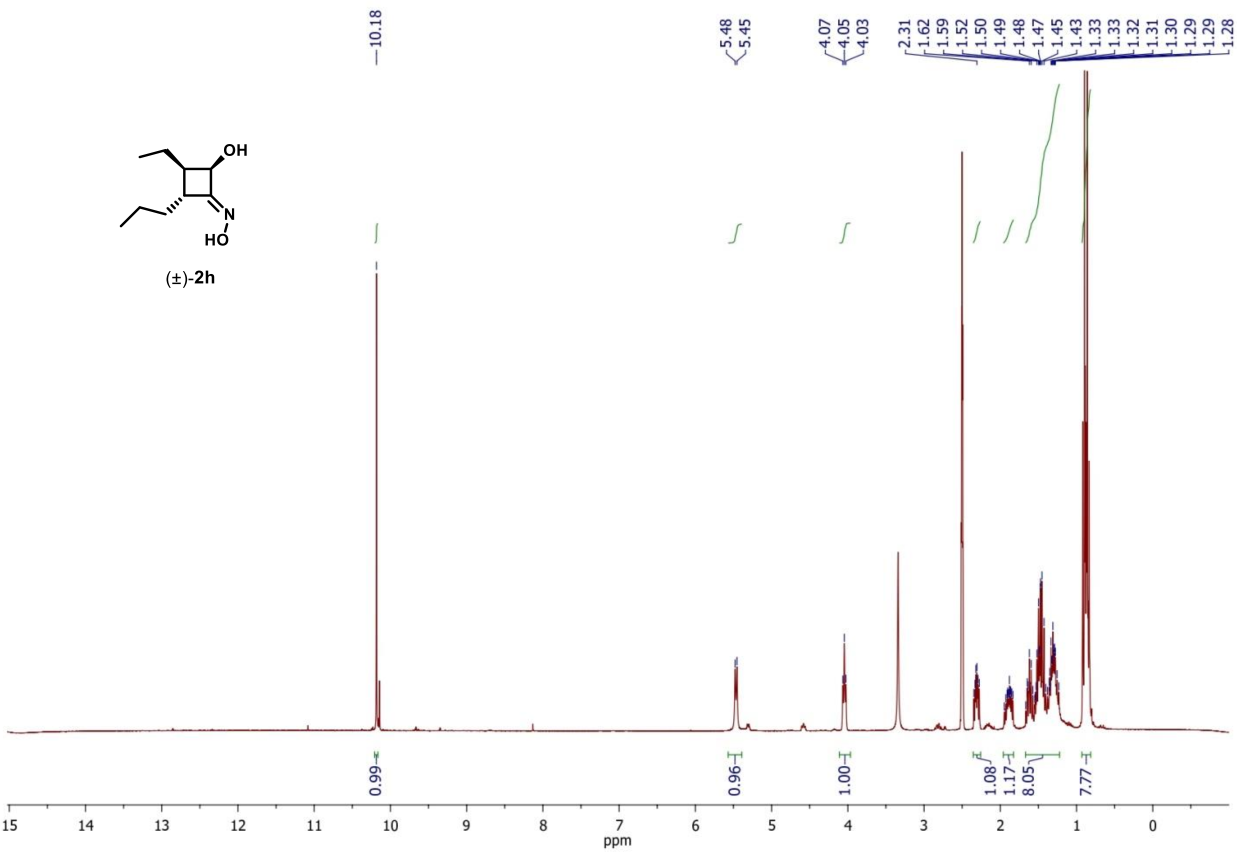

^{13}C{^{1}H}-NMR (75 MHz, DMSO-d_6)

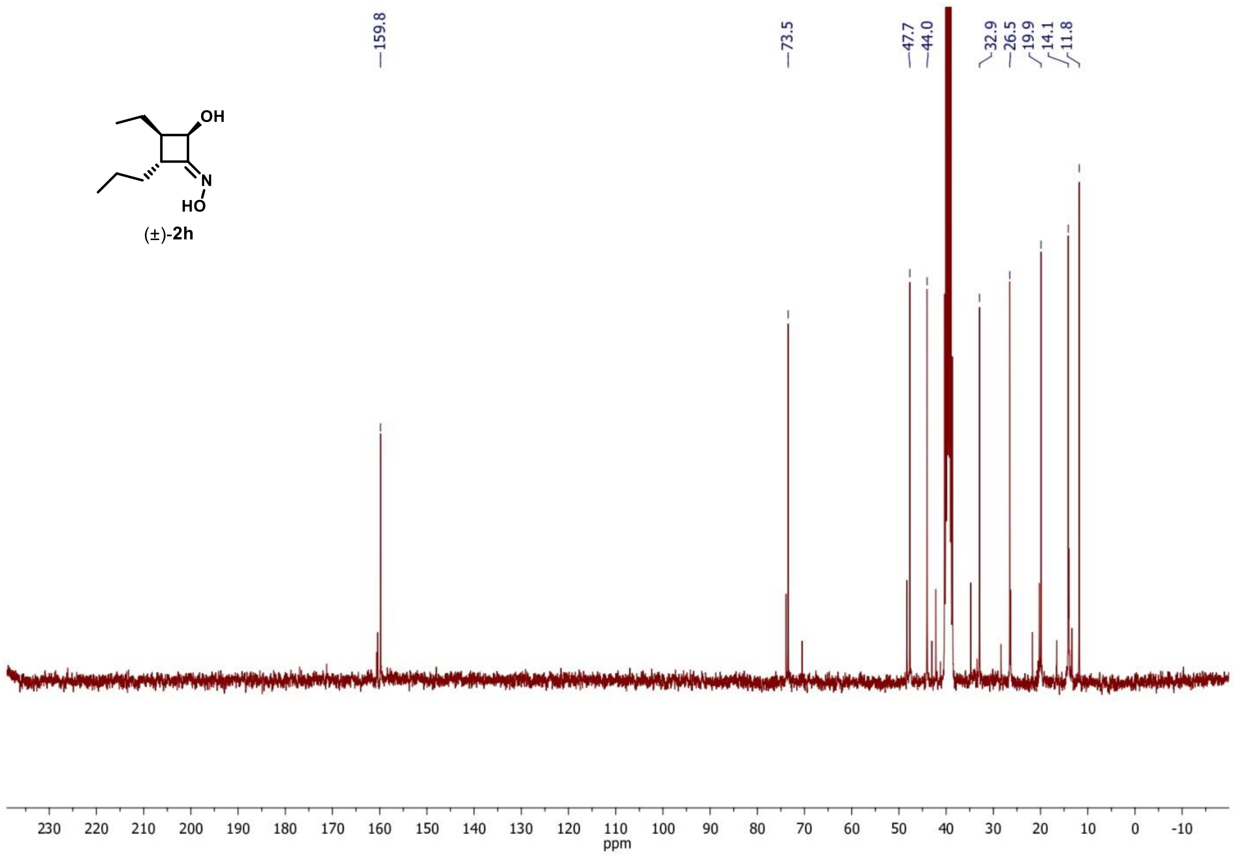

^{1}H-NMR (300 MHz, DMSO-d_6)

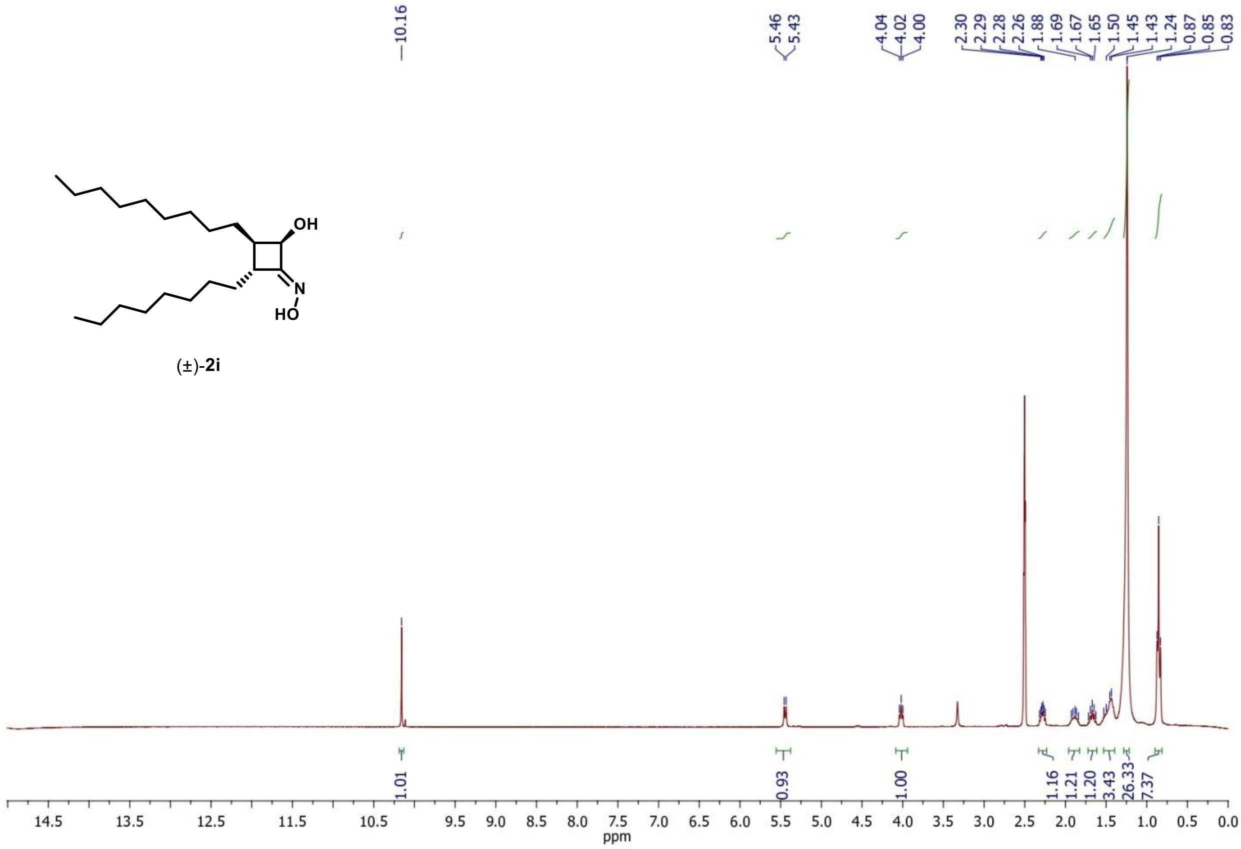

^{13}C{^{1}H}-NMR (75 MHz, DMSO-d_6)

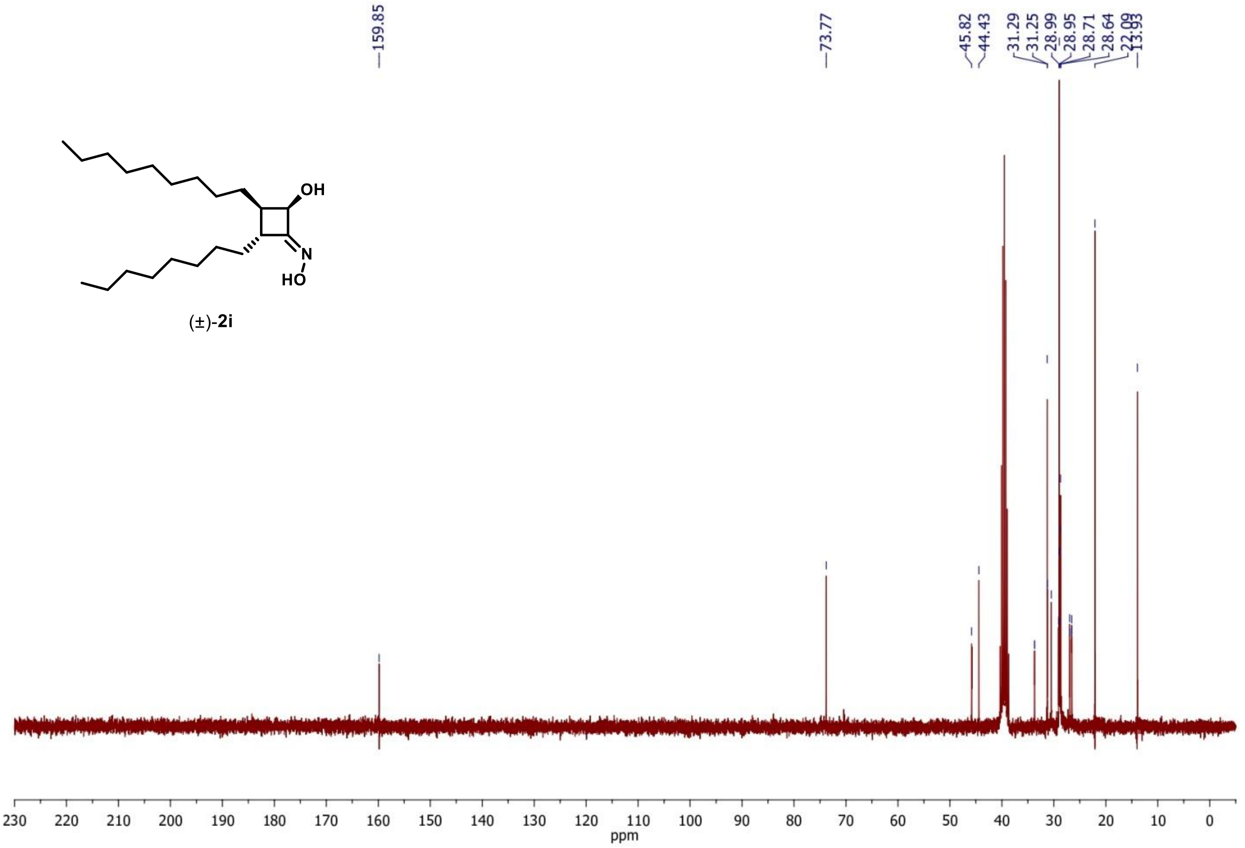

^{1}H-NMR (300 MHz, DMSO-d_6)

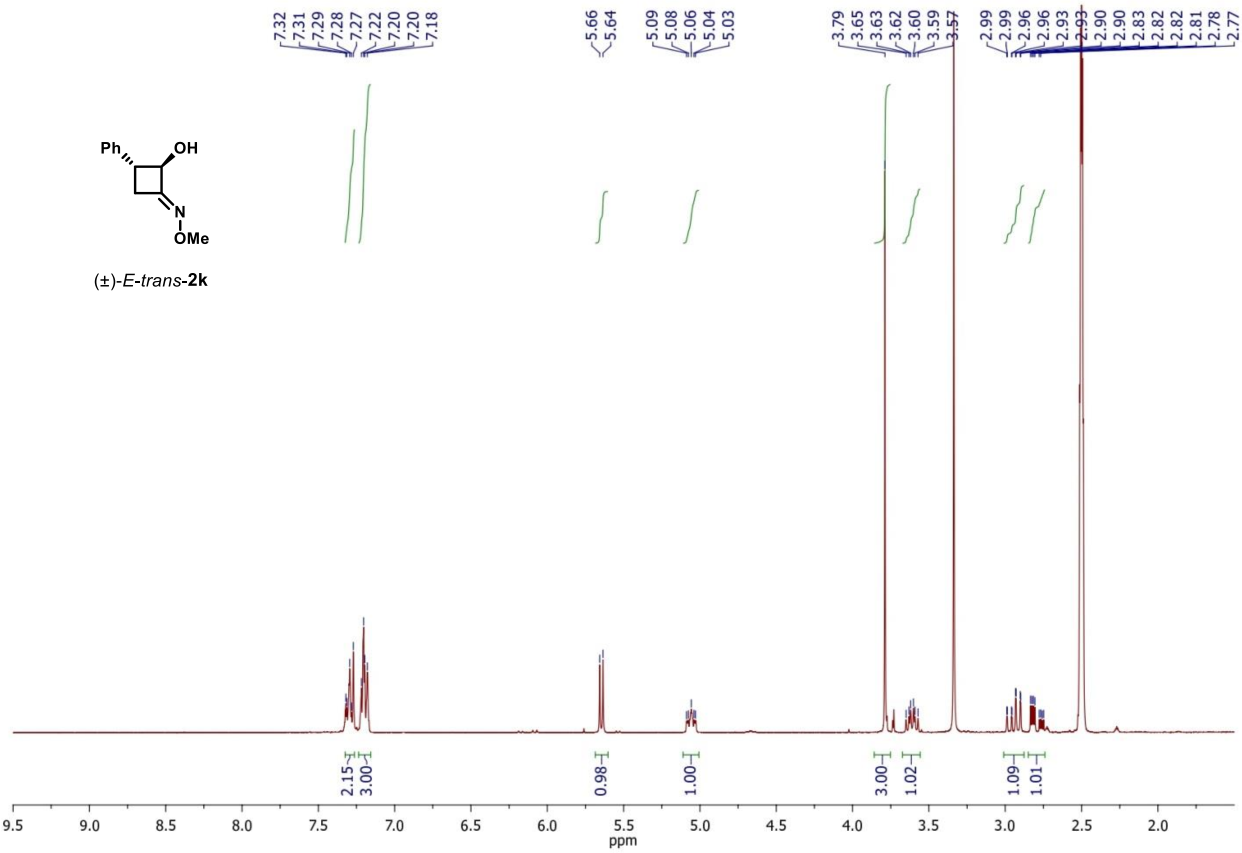

^{13}C{^{1}H}-NMR (75 MHz, DMSO-d_6)

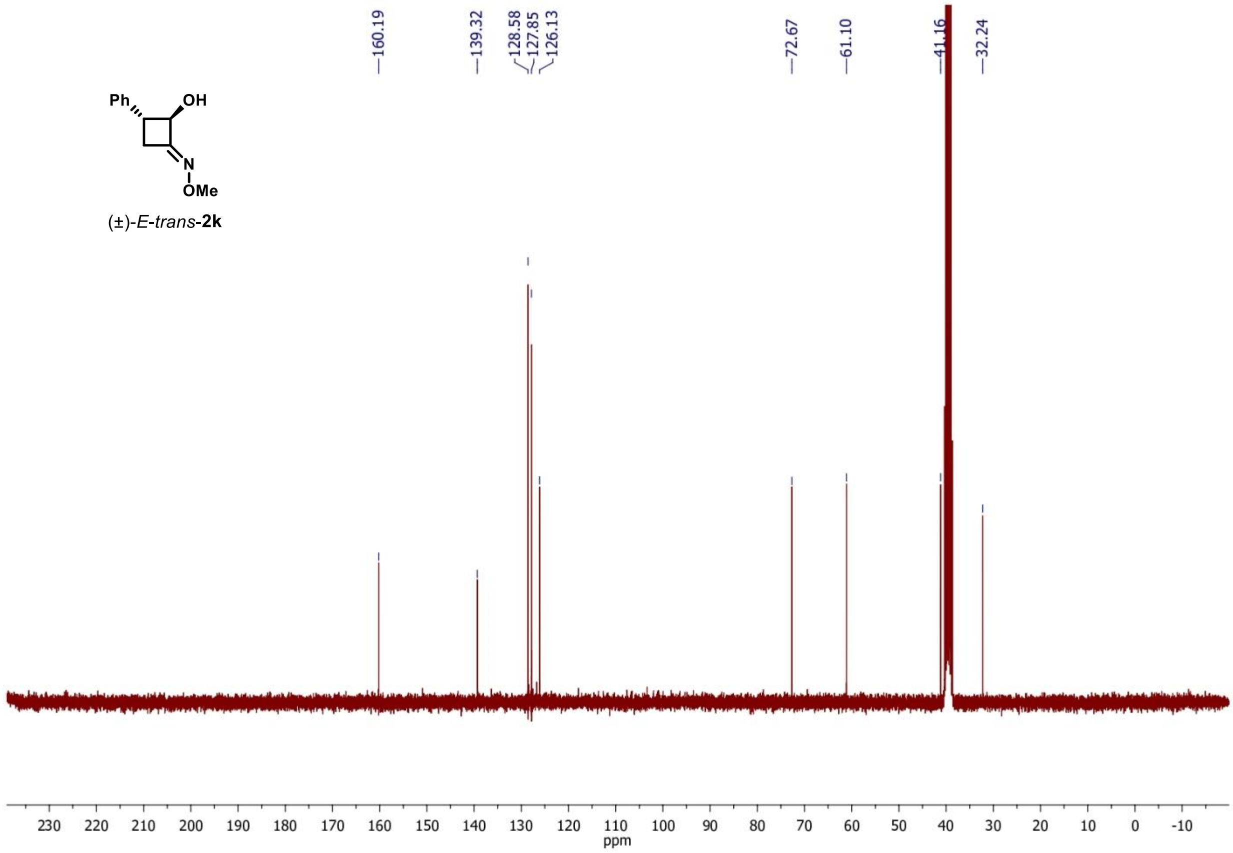

COSY-45 (300 MHz, DMSO-d_6)

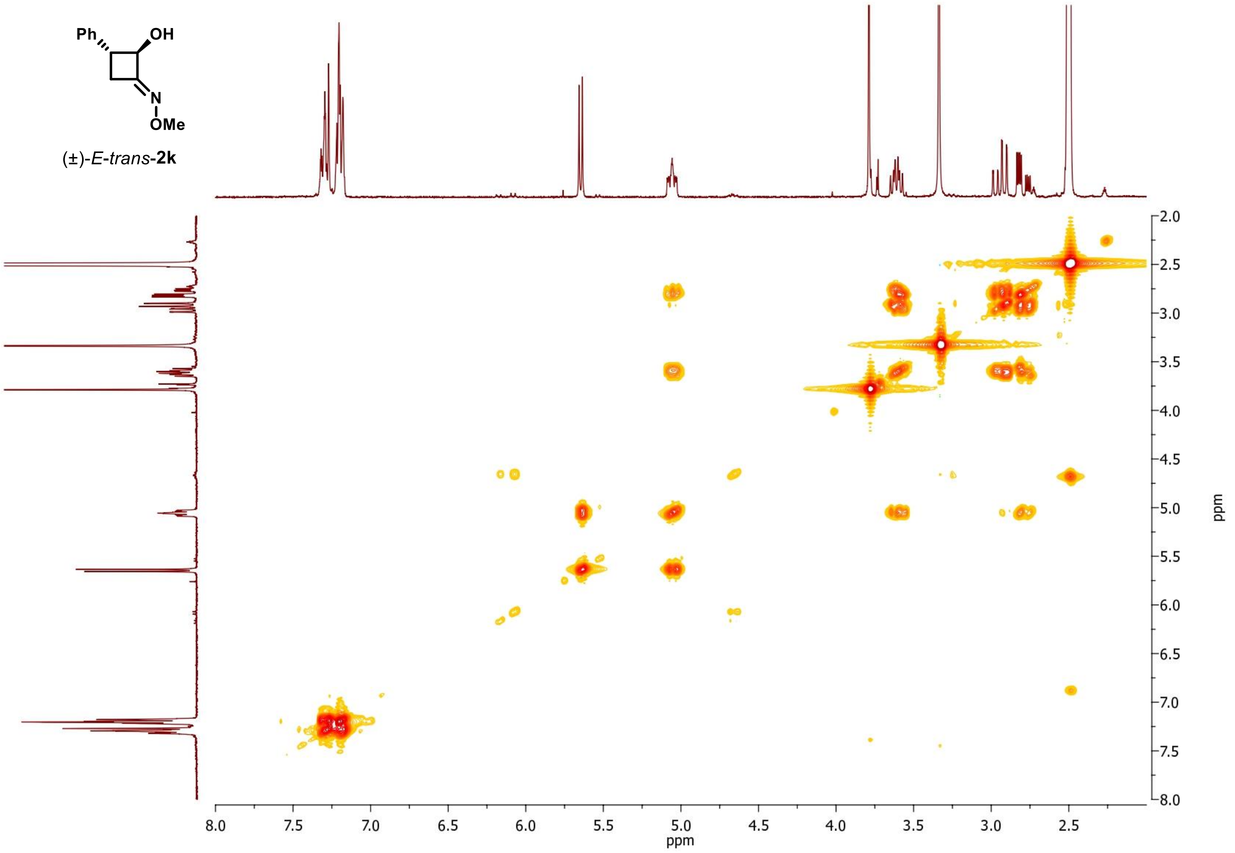

NOESY (300 MHz, DMSO-d_6)

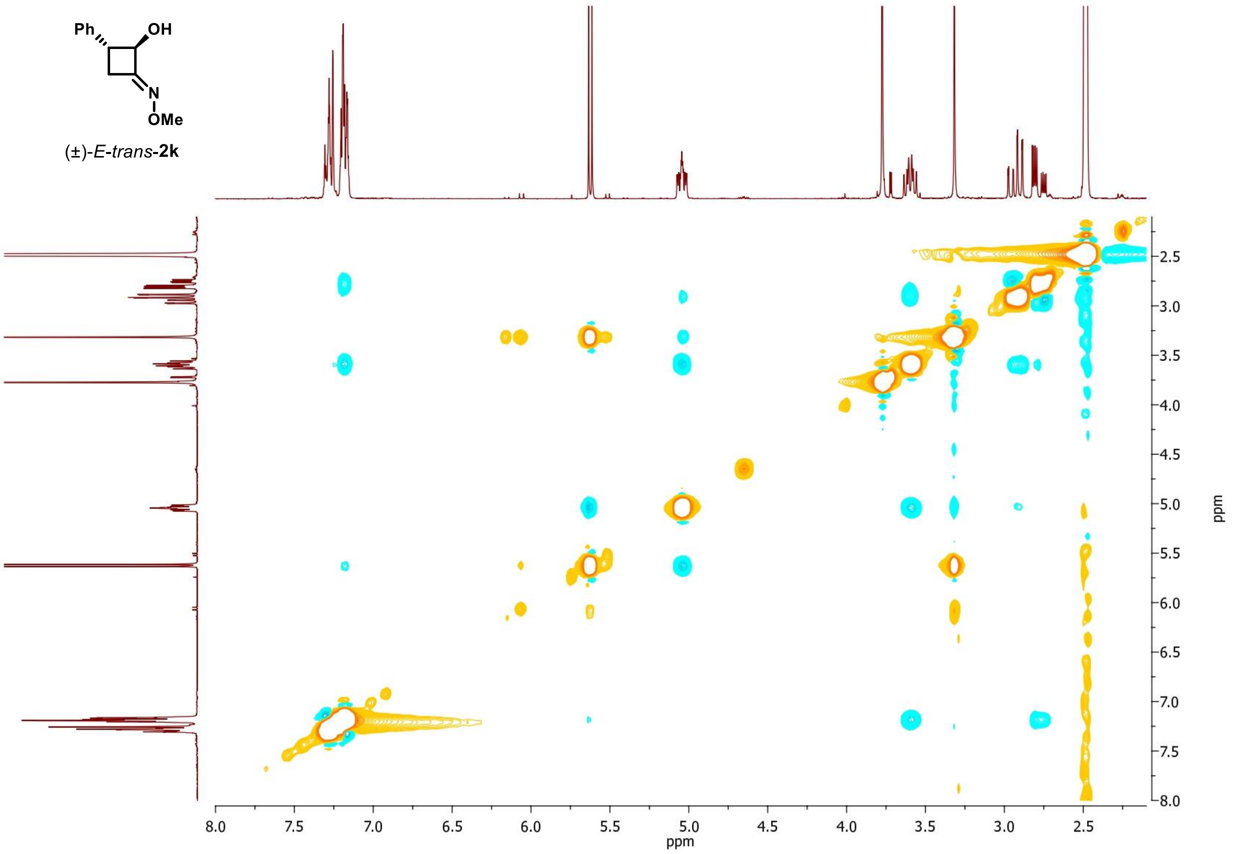

HSQC (300 MHz, DMSO-d_6)

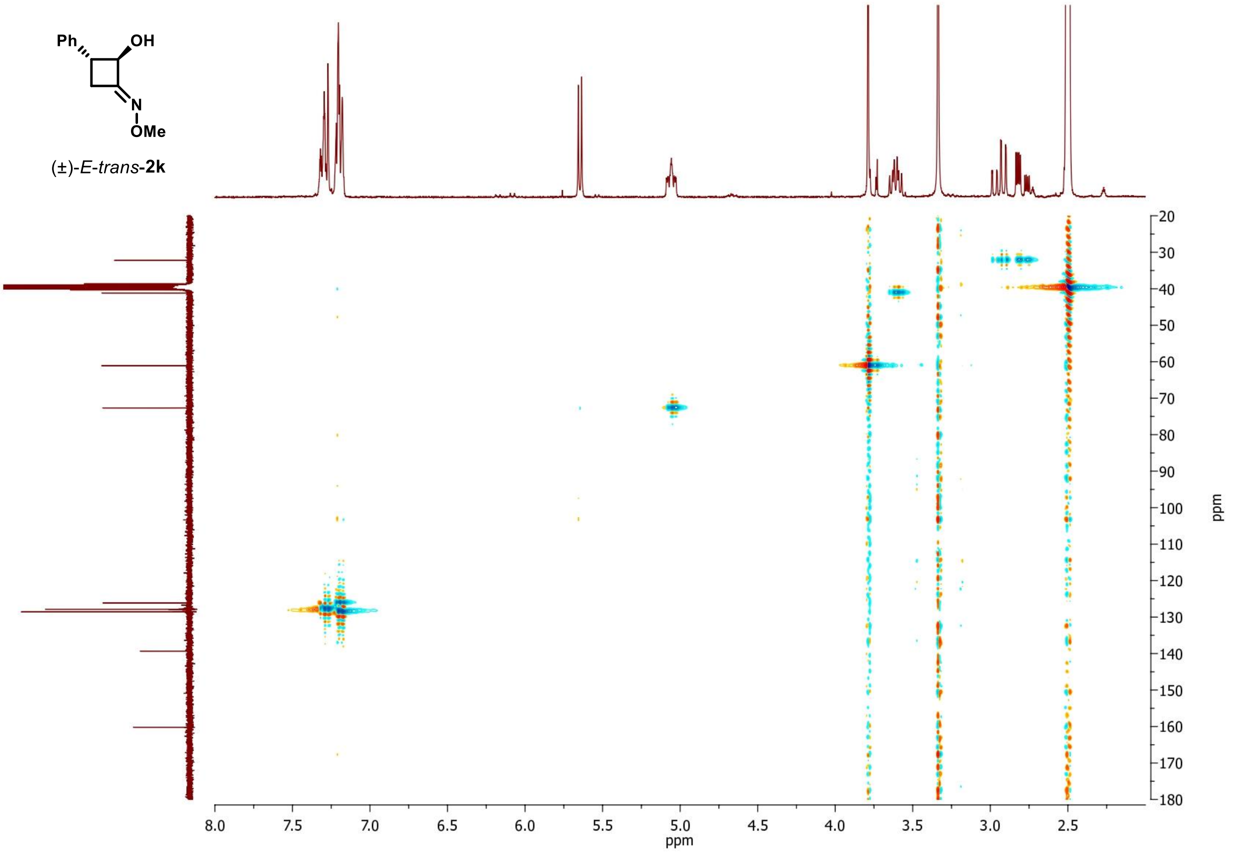

HMBC (300 MHz, DMSO-d_6)

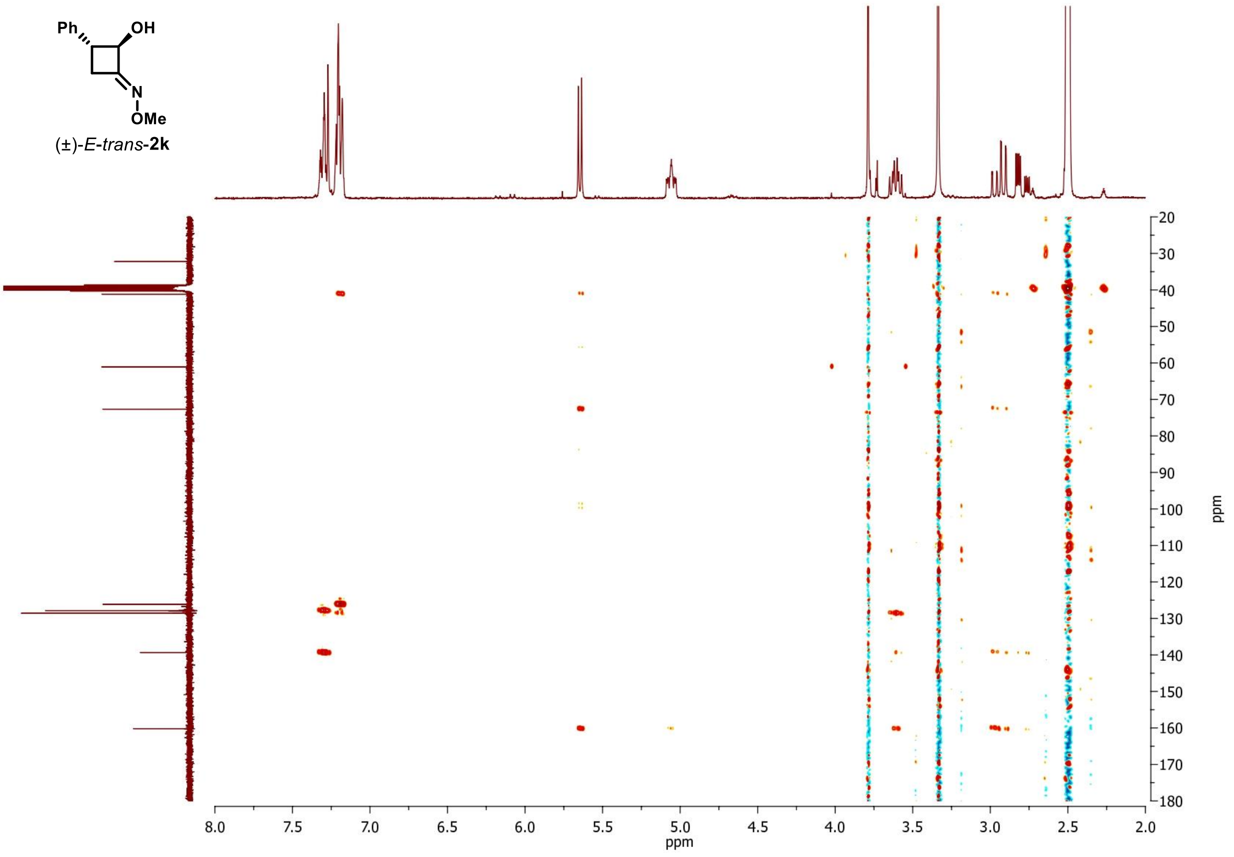

^{1}H-NMR (300 MHz, DMSO-d_6)

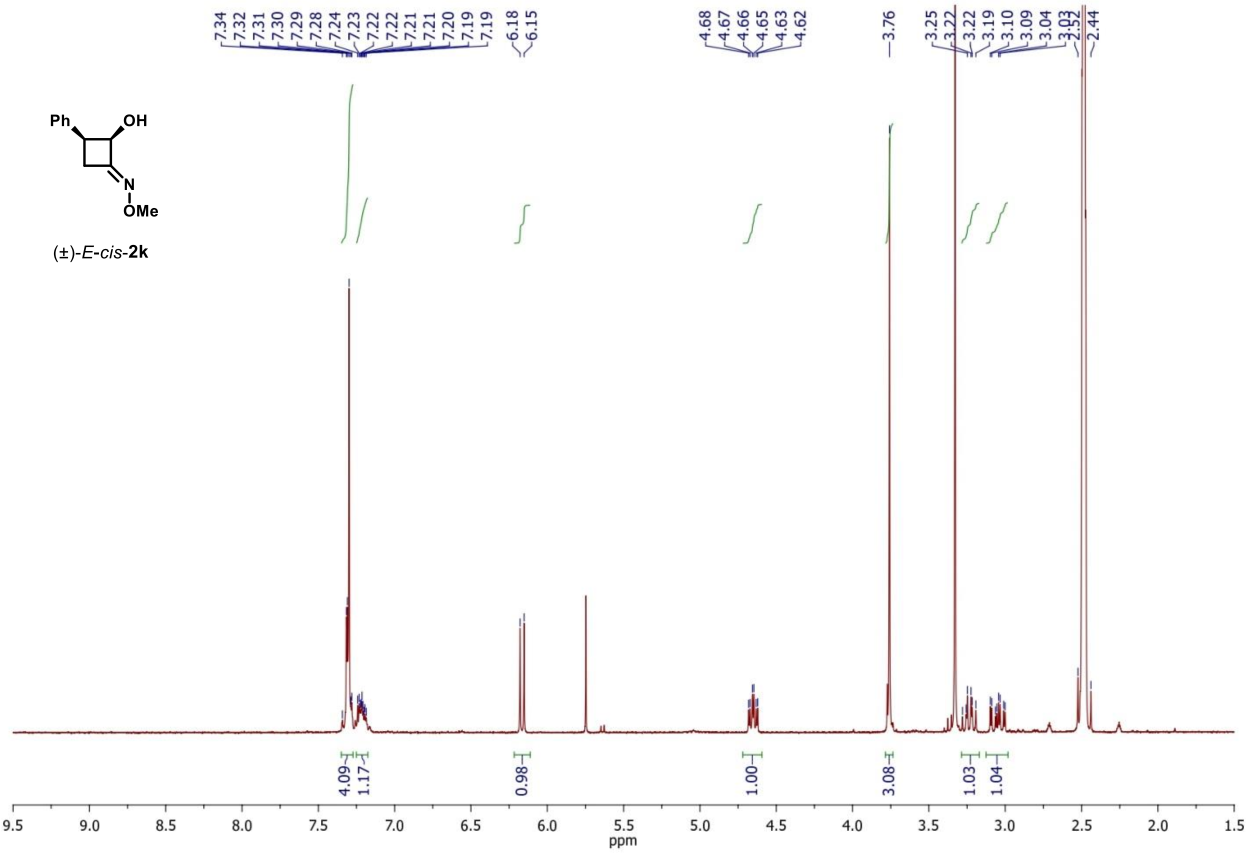

^{13}C{^{1}H}-NMR (75 MHz, DMSO-d_6)

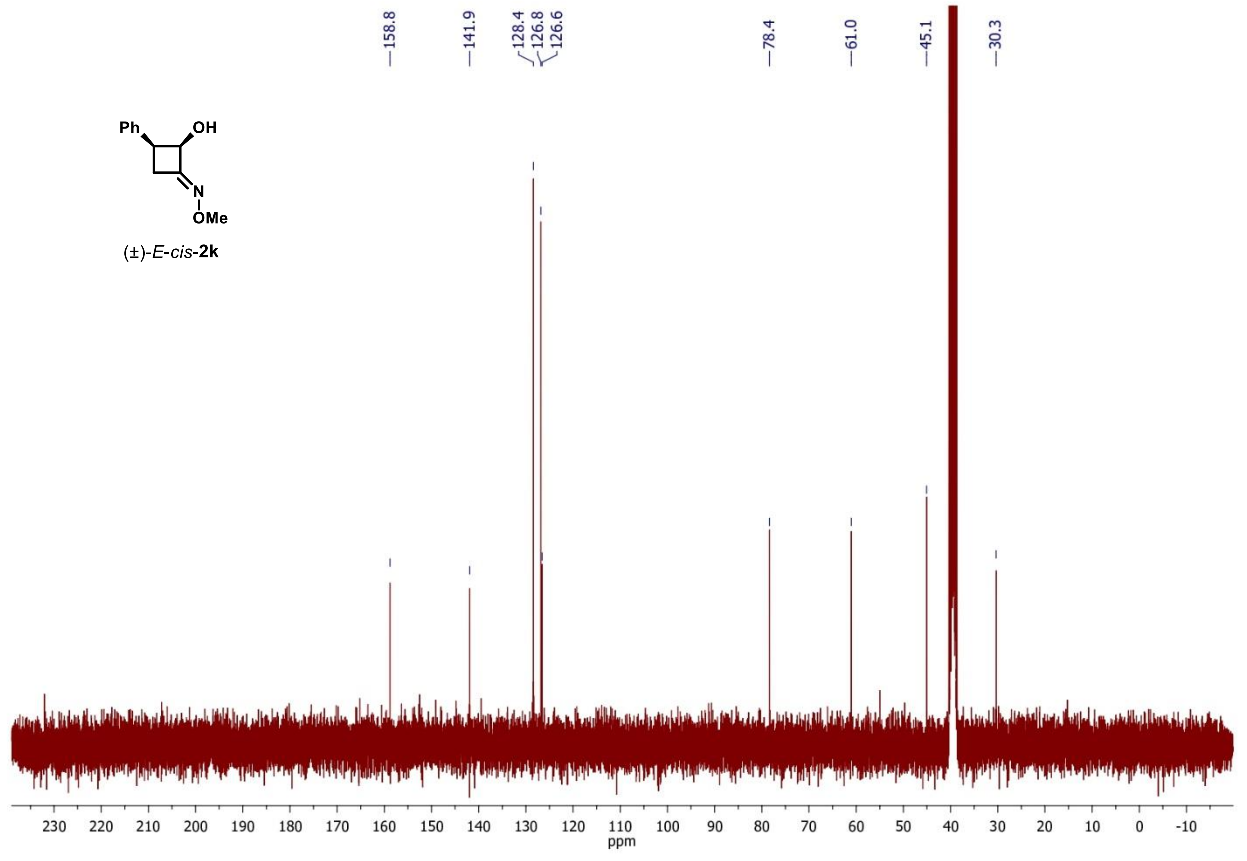

COSY-45 (300 MHz, DMSO-d_6)

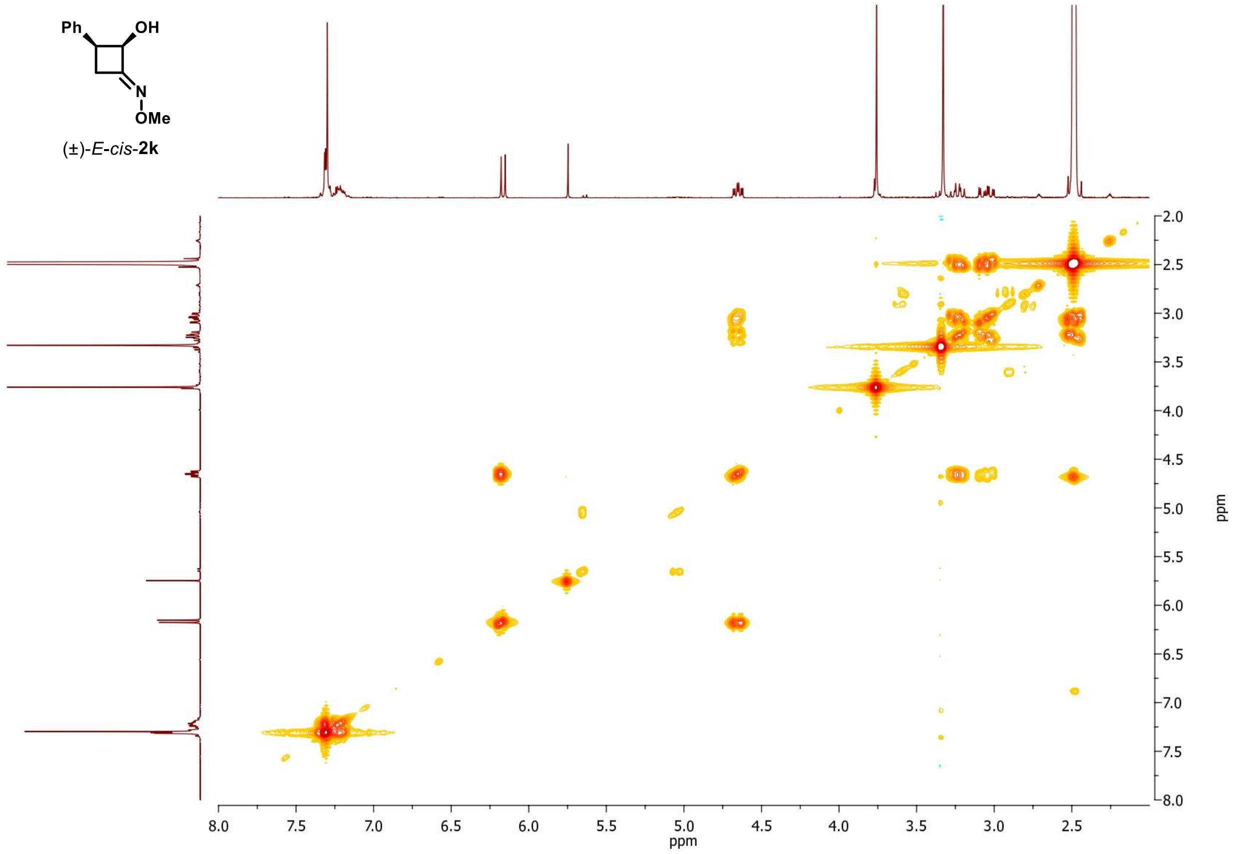

NOESY (300 MHz, DMSO-d_6)

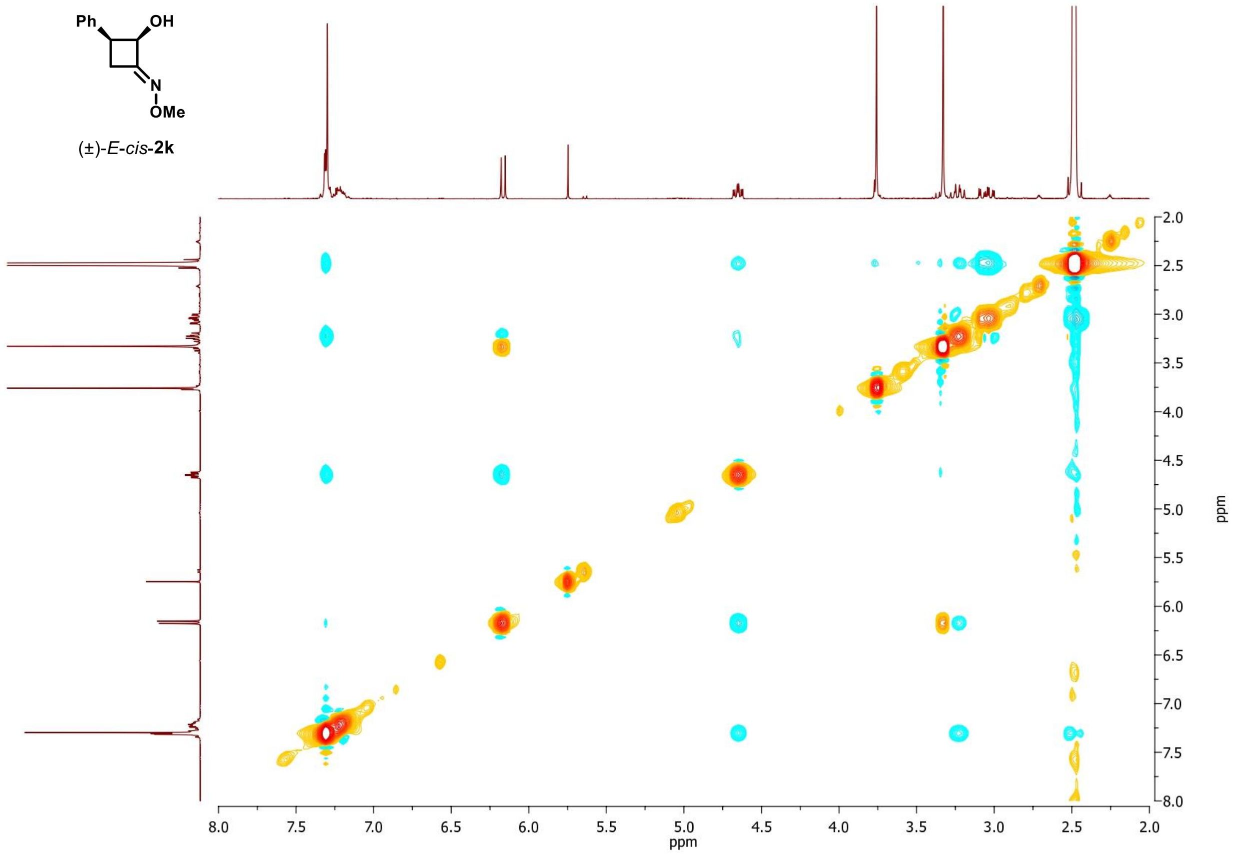

HSQC (300 MHz, DMSO-d_6)

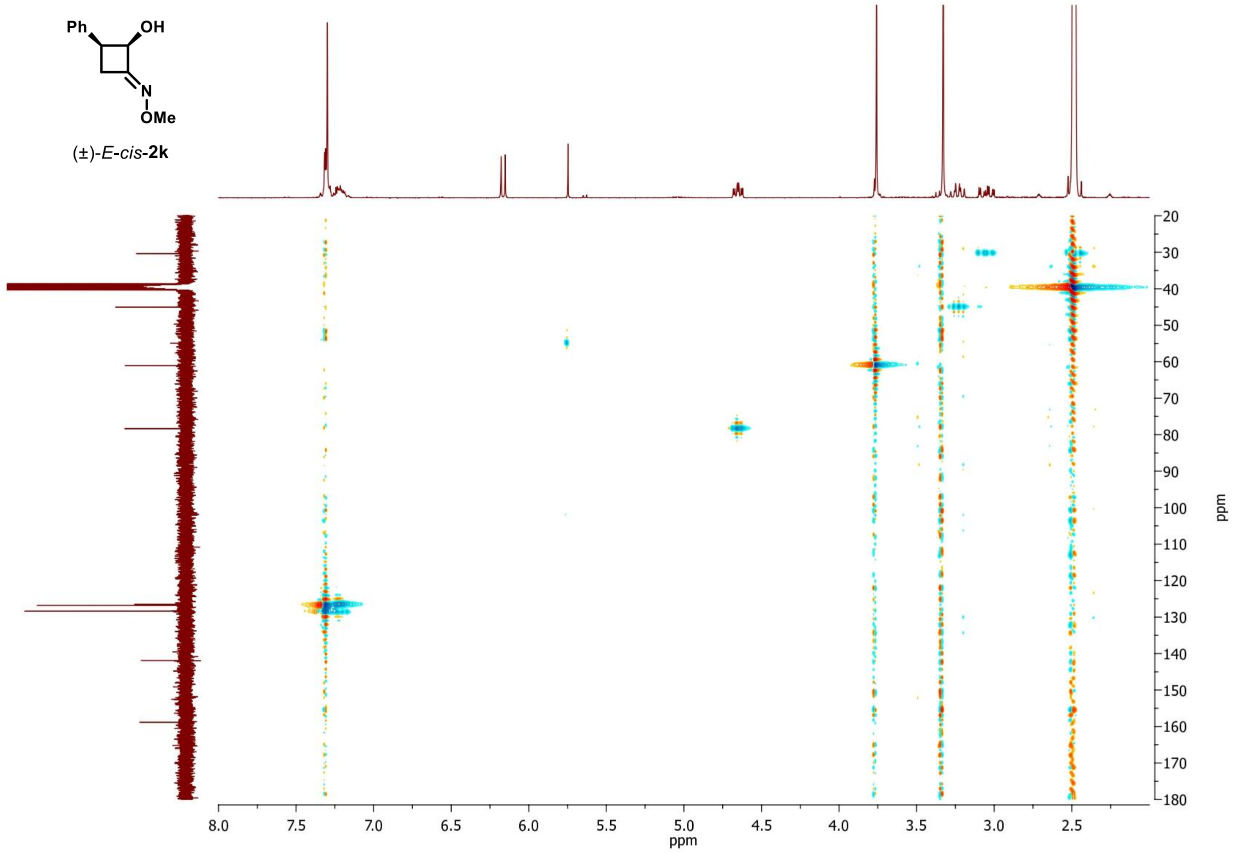

(±)-*E*-*cis*-**2k**

HMBC (300 MHz, DMSO-d_6)

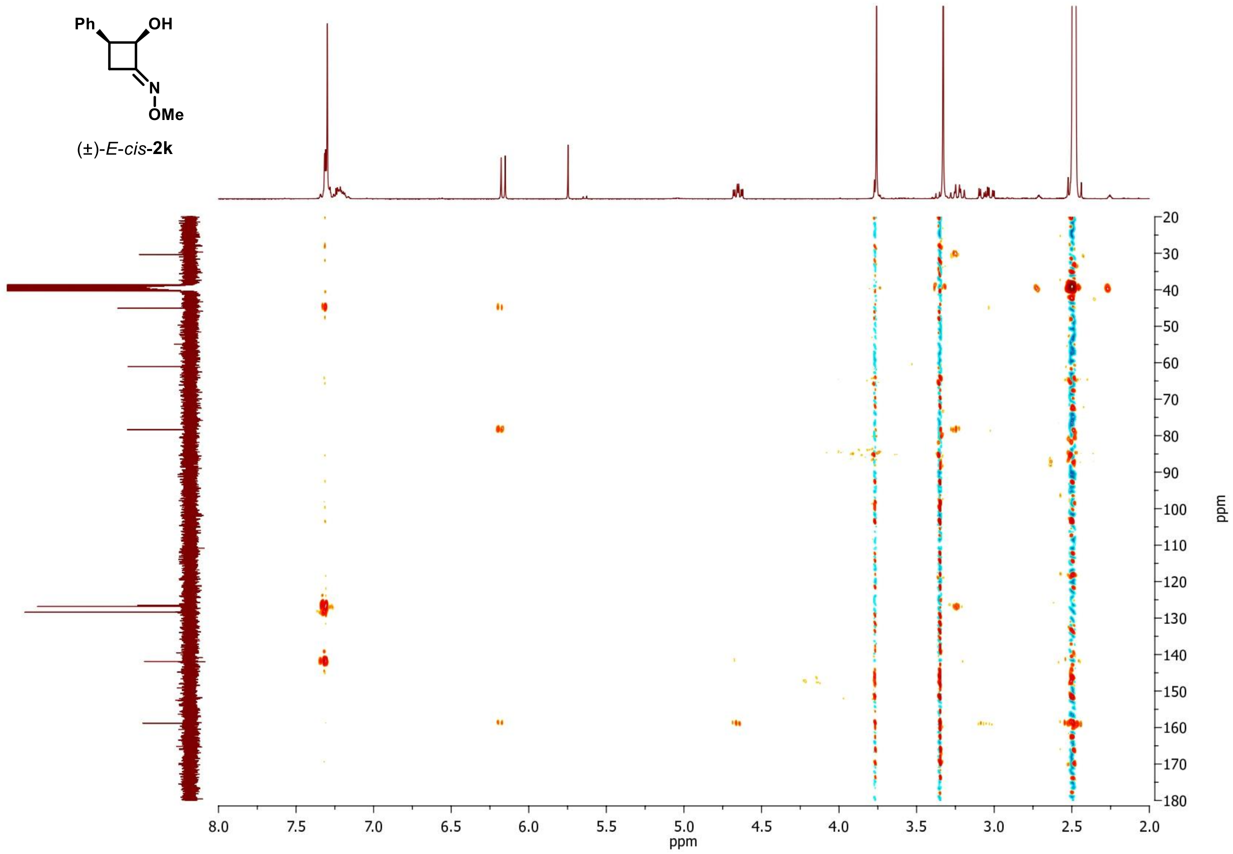

(±)-*E*-*cis*-**2k**

^{1}H-NMR (300 MHz, DMSO-d_6)

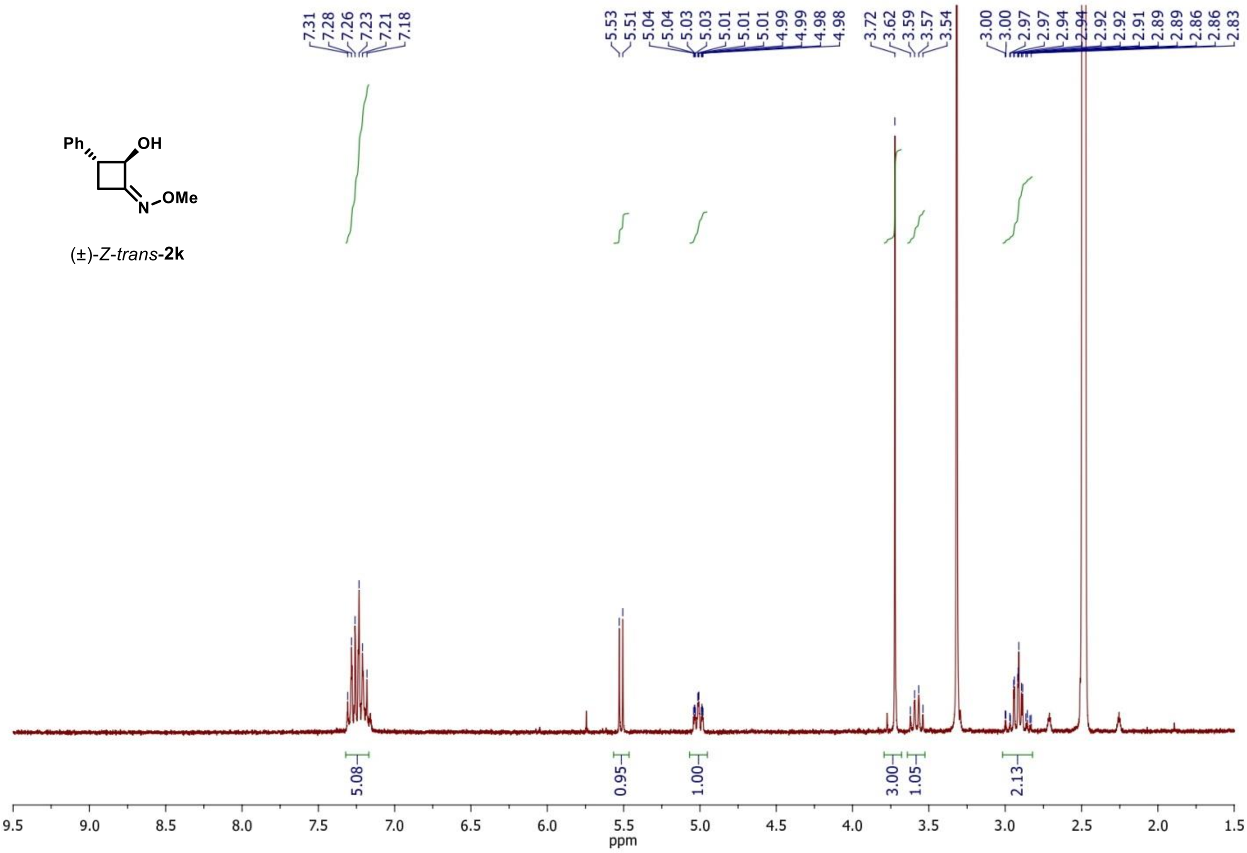

^{13}C{^{1}H}-NMR (75 MHz, DMSO-d_6)

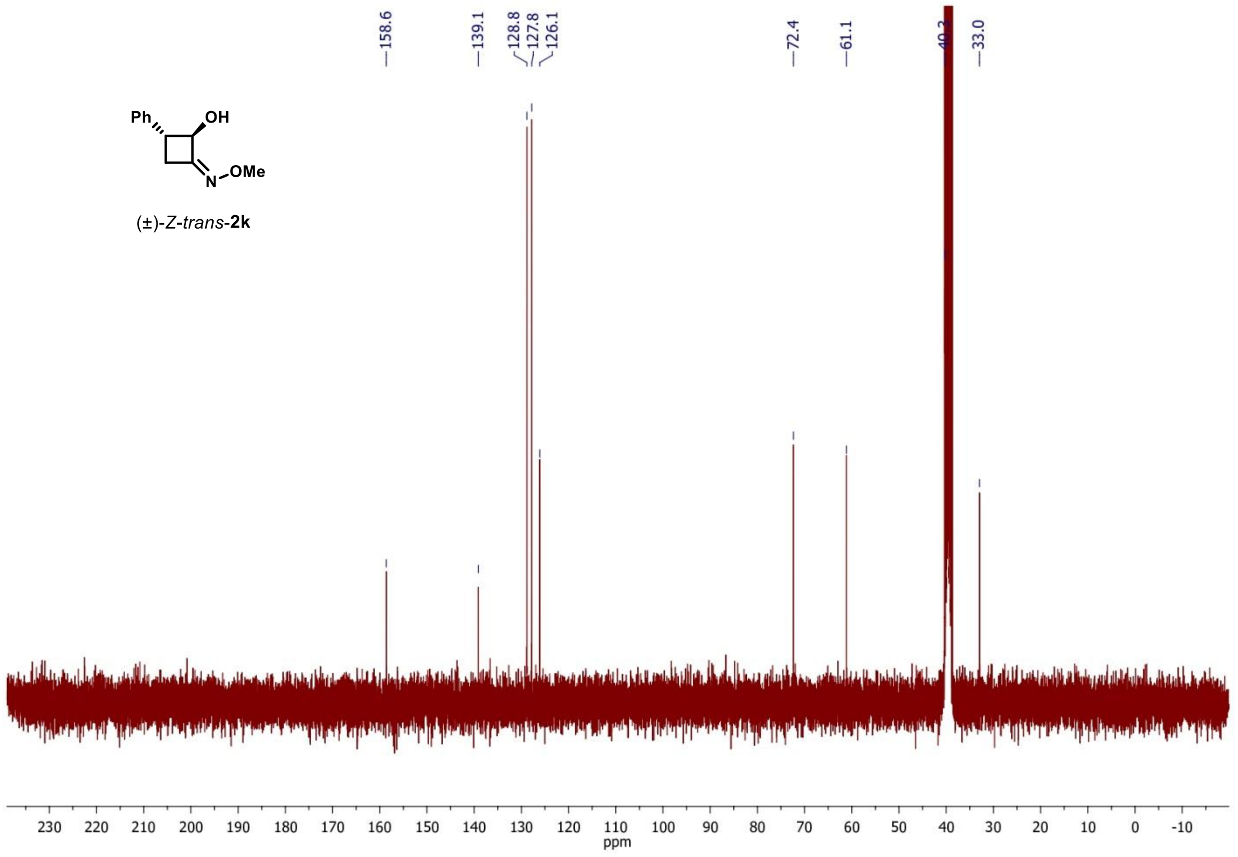

^{1}H-NMR (300 MHz, DMSO-d_6)

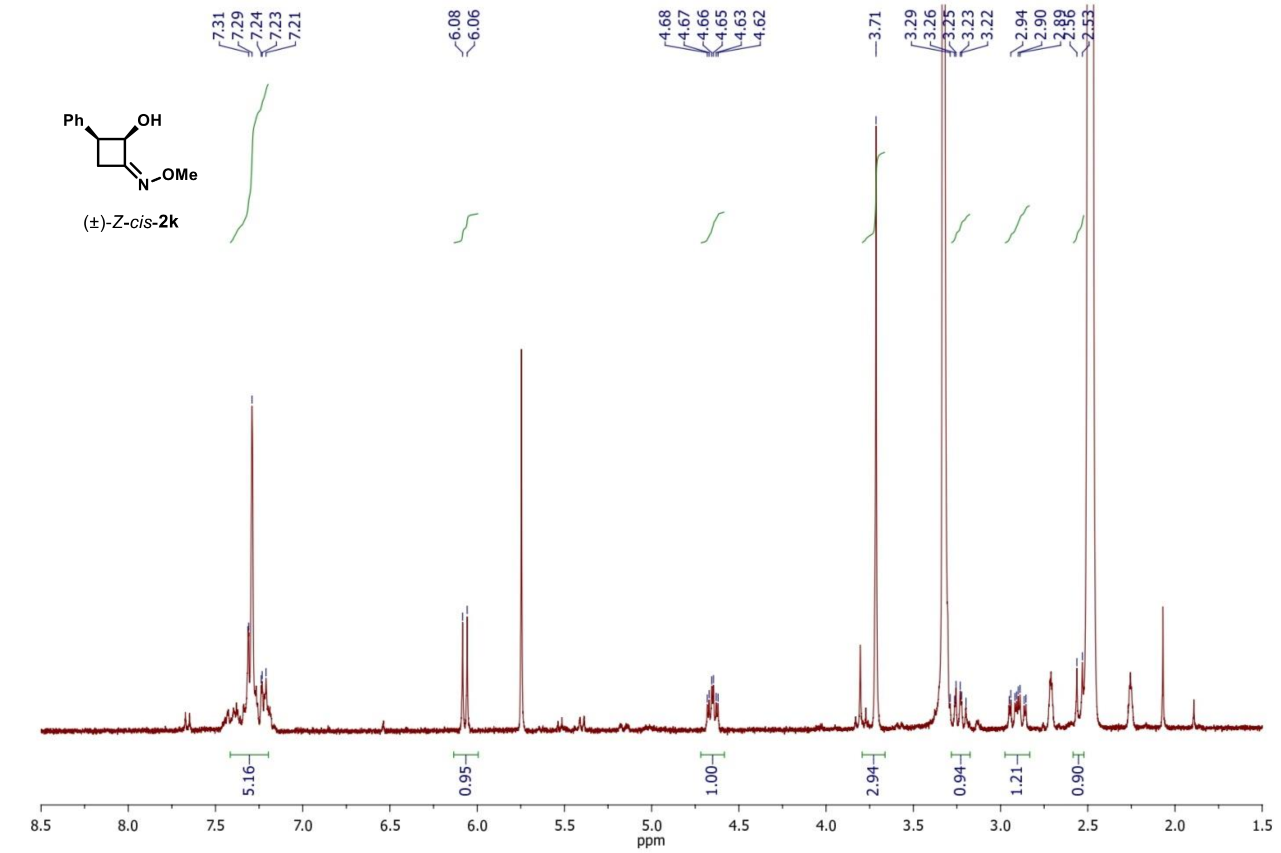

^{1}H-NMR (300 MHz, DMSO-d_6)

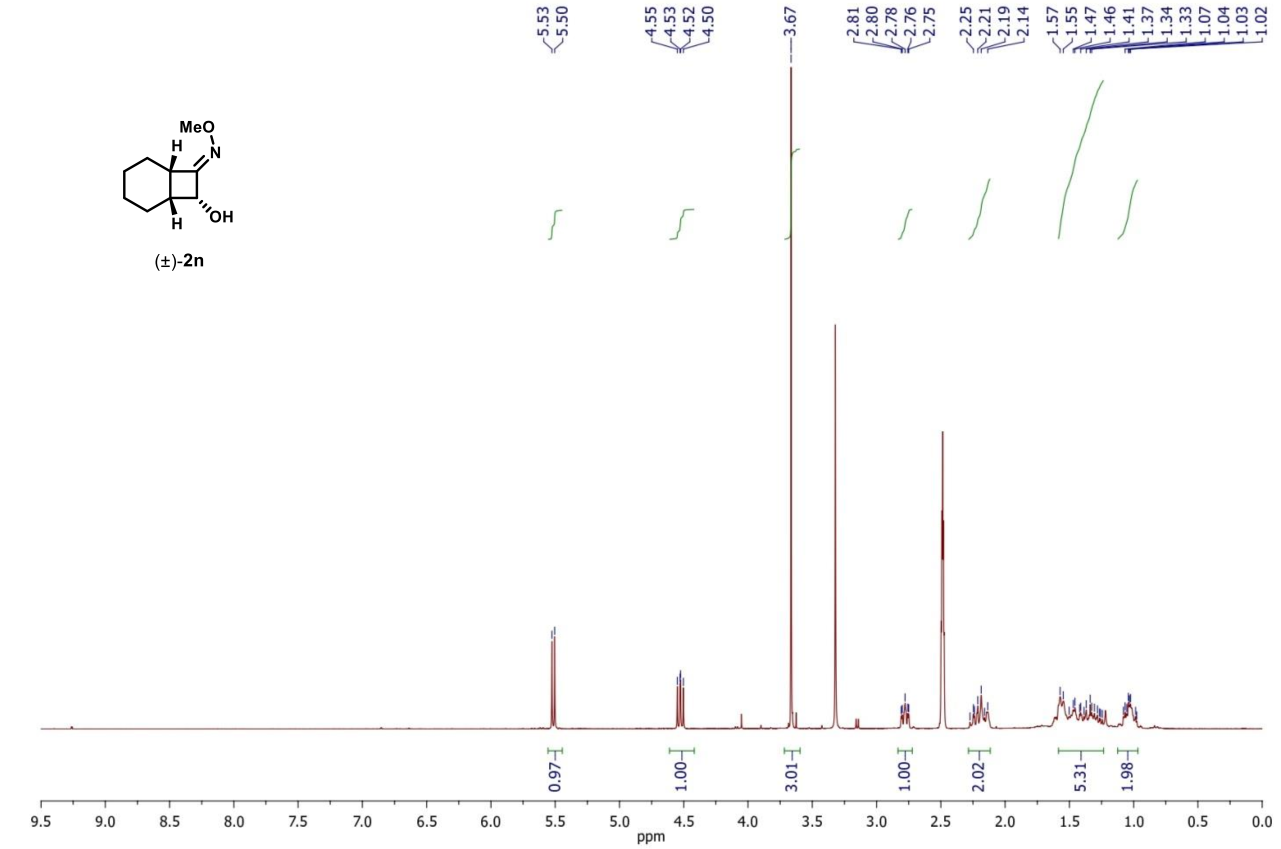

$^{13}C\{^{1}H\}$-NMR (75 MHz, DMSO-d_6)

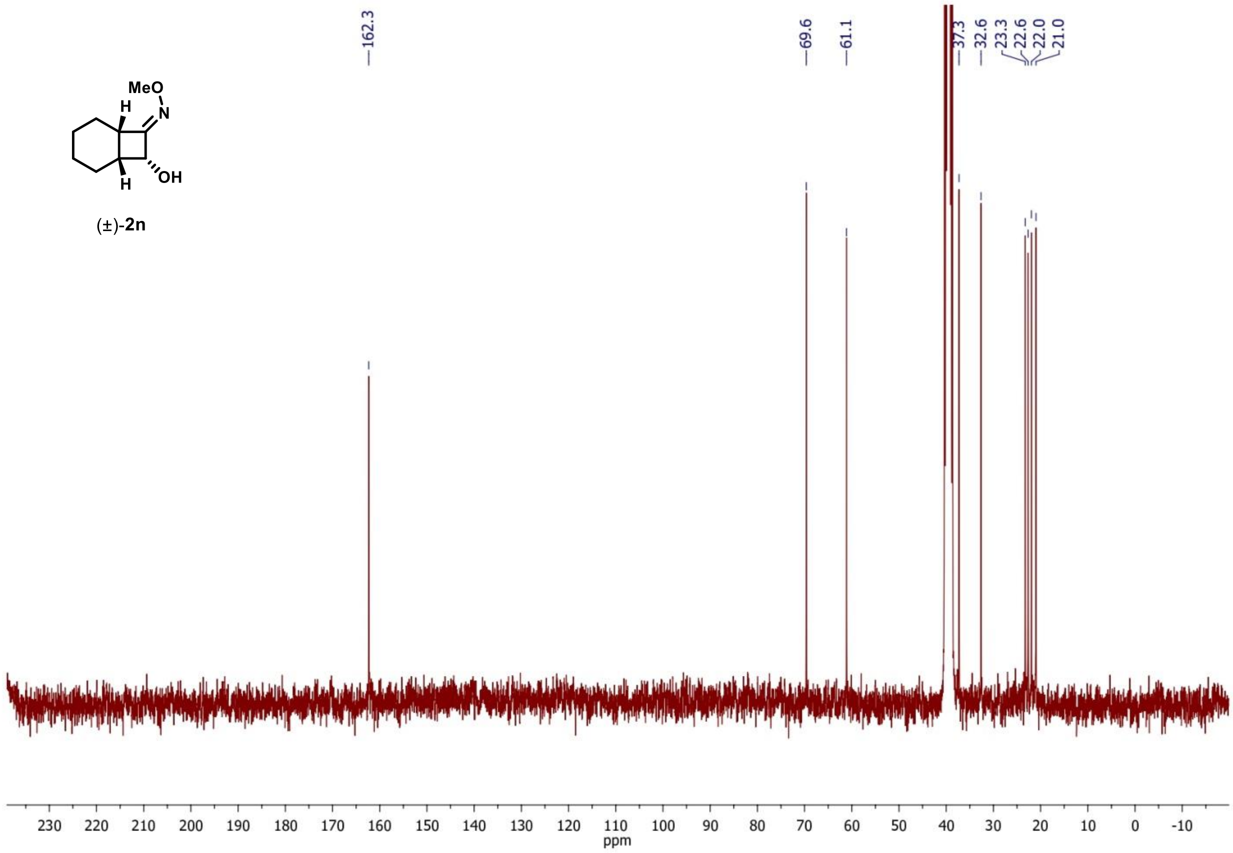

NOESY (300 MHz, DMSO-d_6)

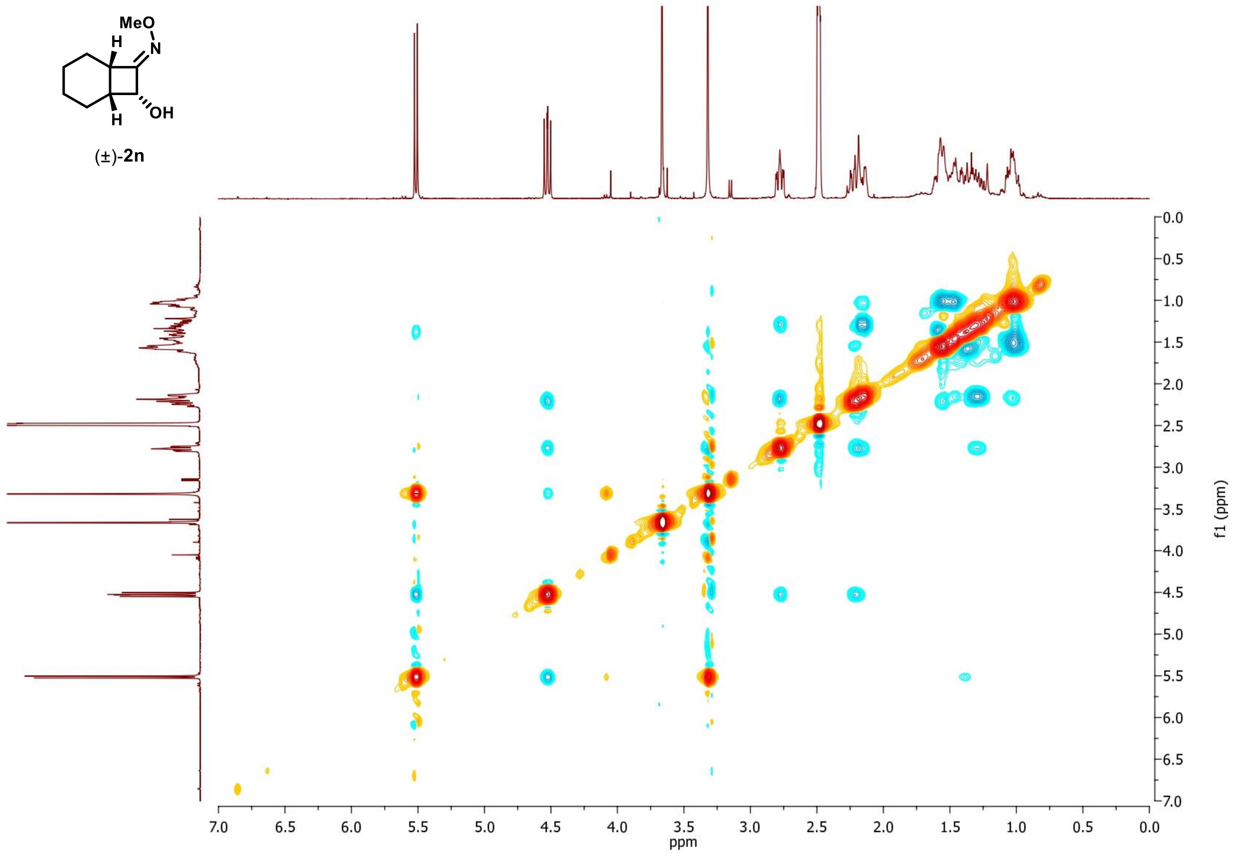

HSQC (300 MHz, DMSO-d_6)

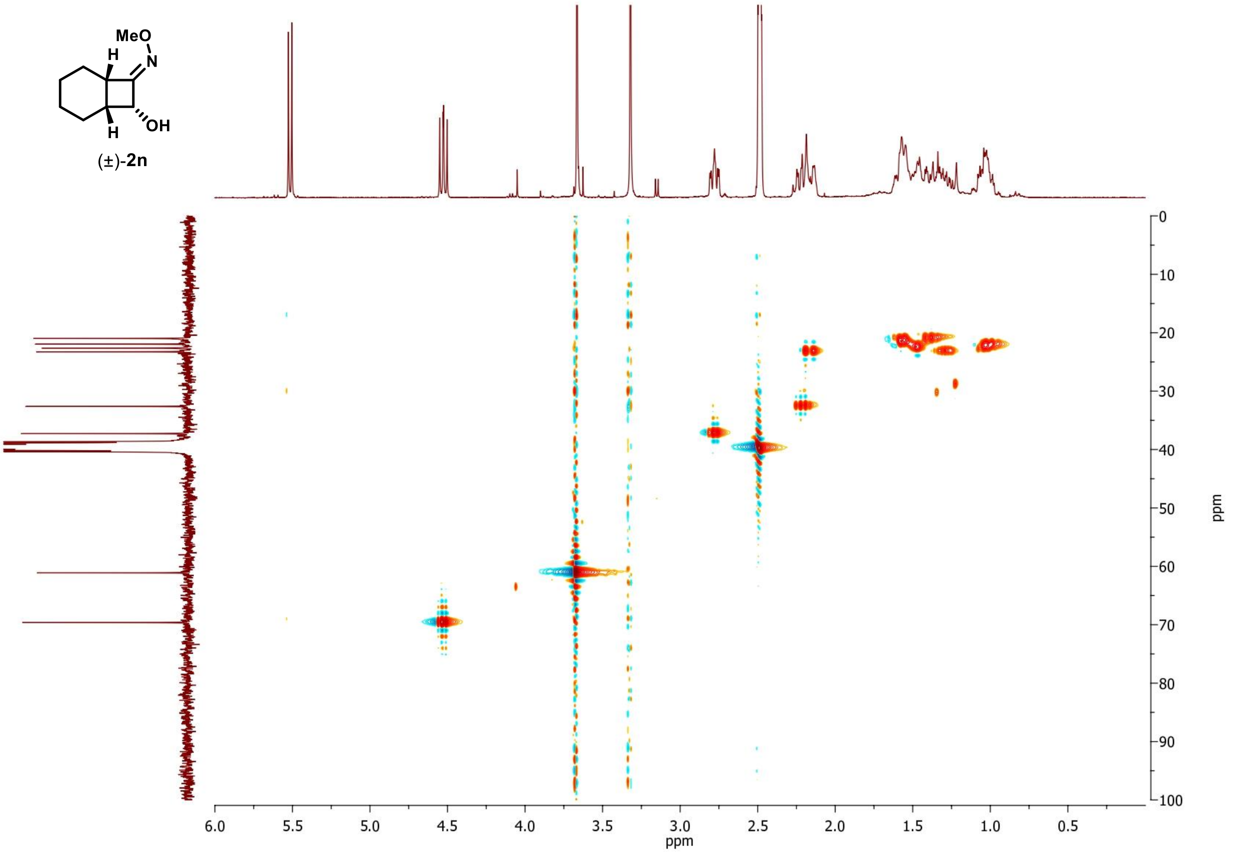

HMBC (300 MHz, DMSO-d_6)

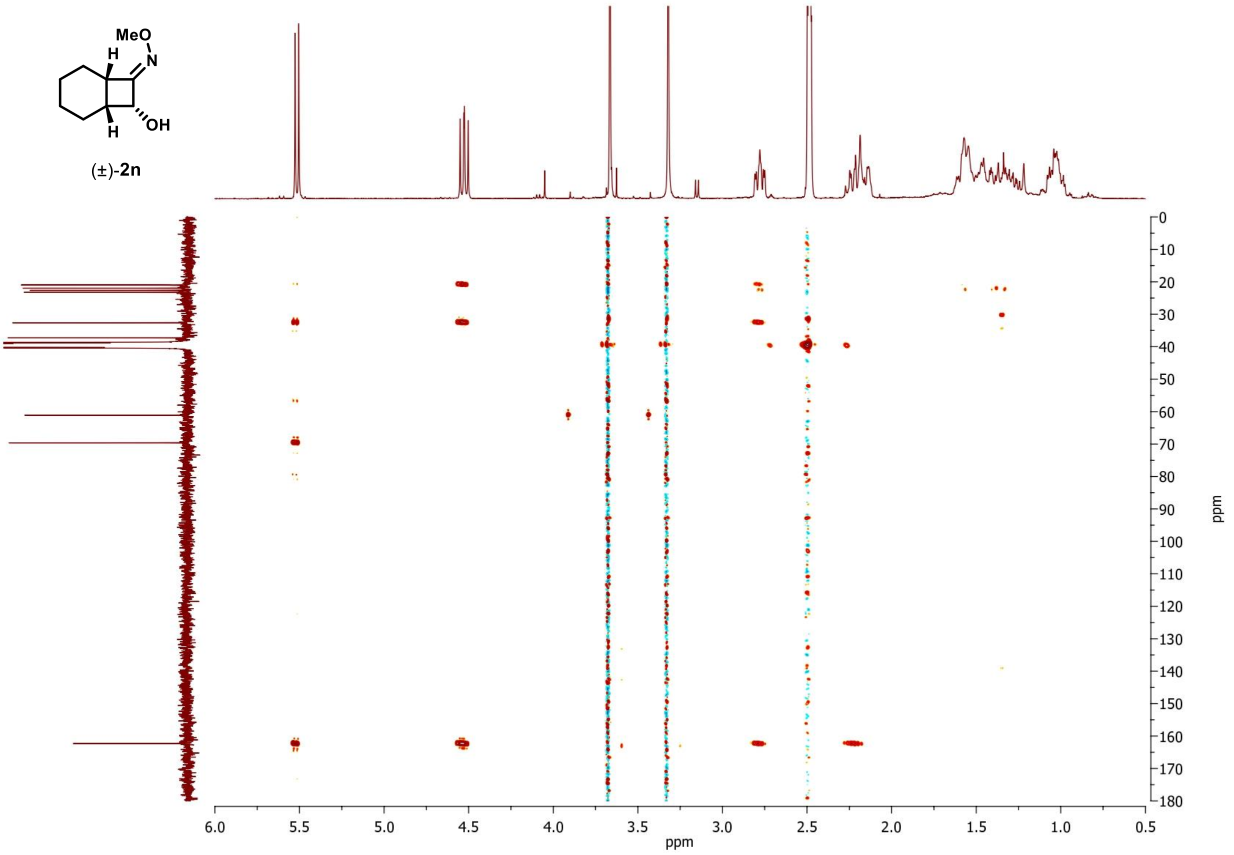

^{1}H-NMR (300 MHz, DMSO-d_6)

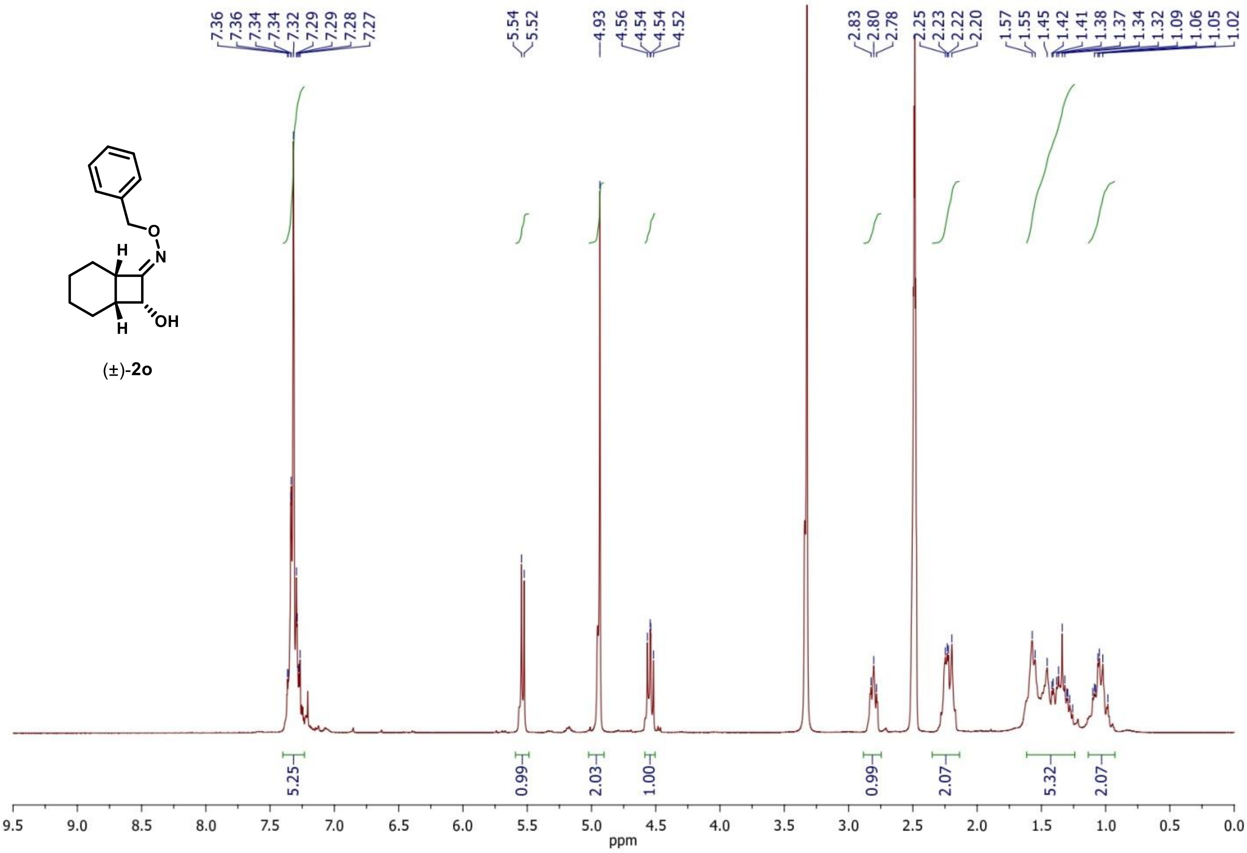

^{13}C{^{1}H}-NMR (75 MHz, DMSO-d_6)

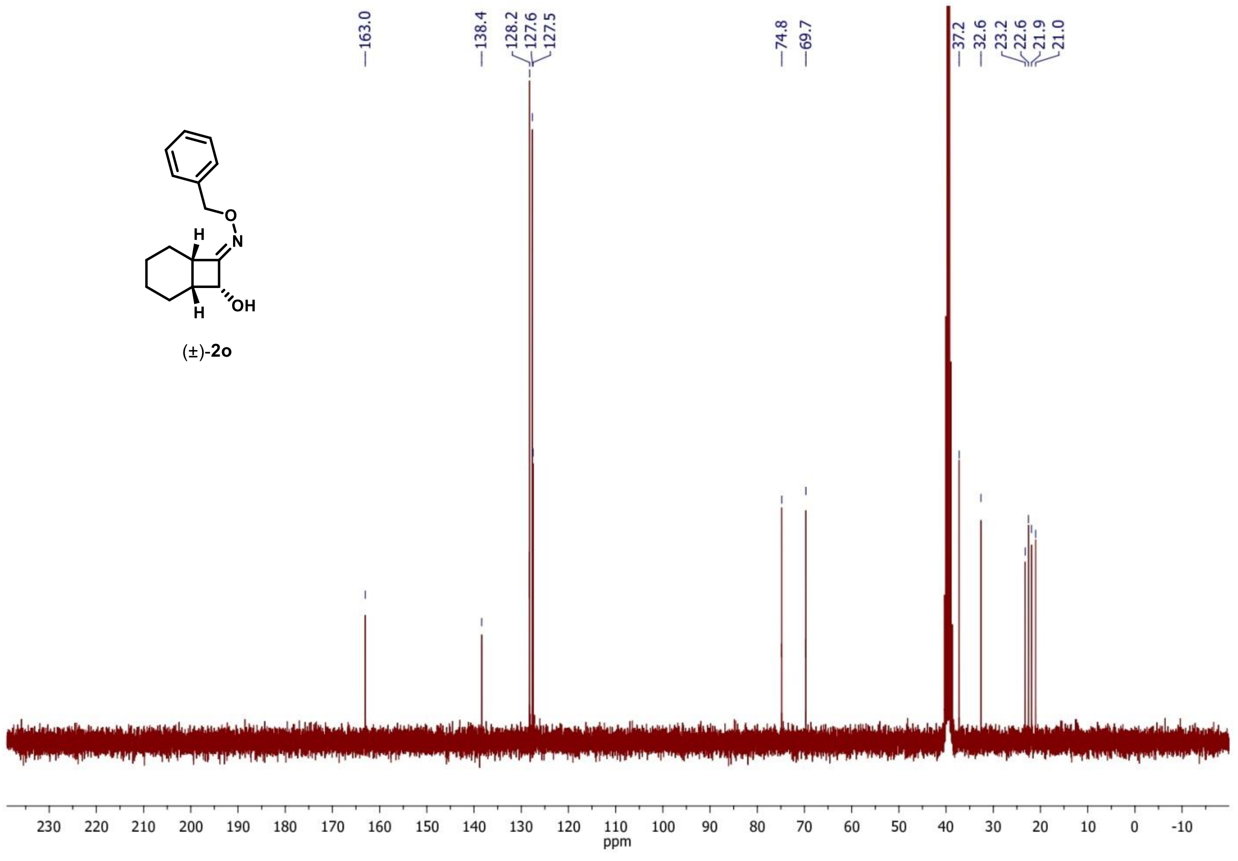

NOESY (300 MHz, DMSO-d_6)

(±)-**2o**

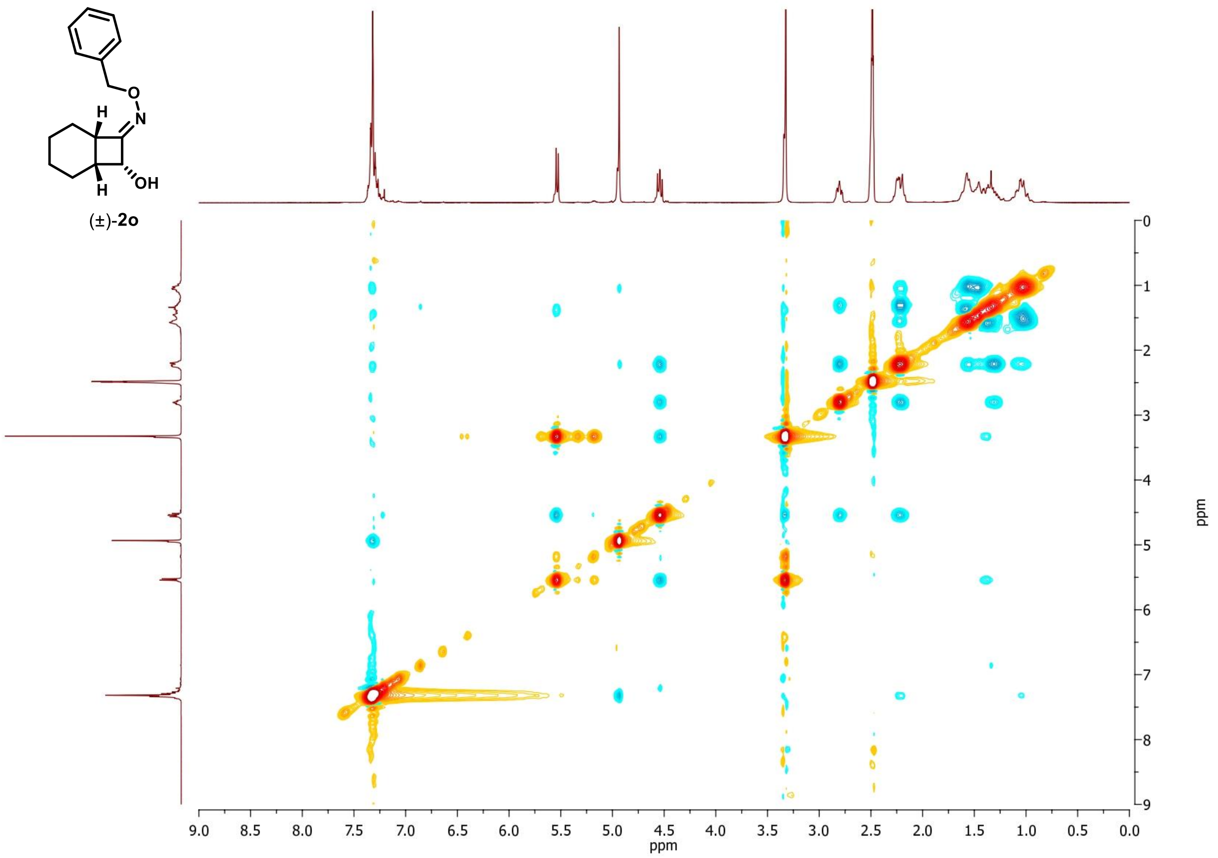

HSQC (300 MHz, DMSO-d_6)

(±)-**2o**

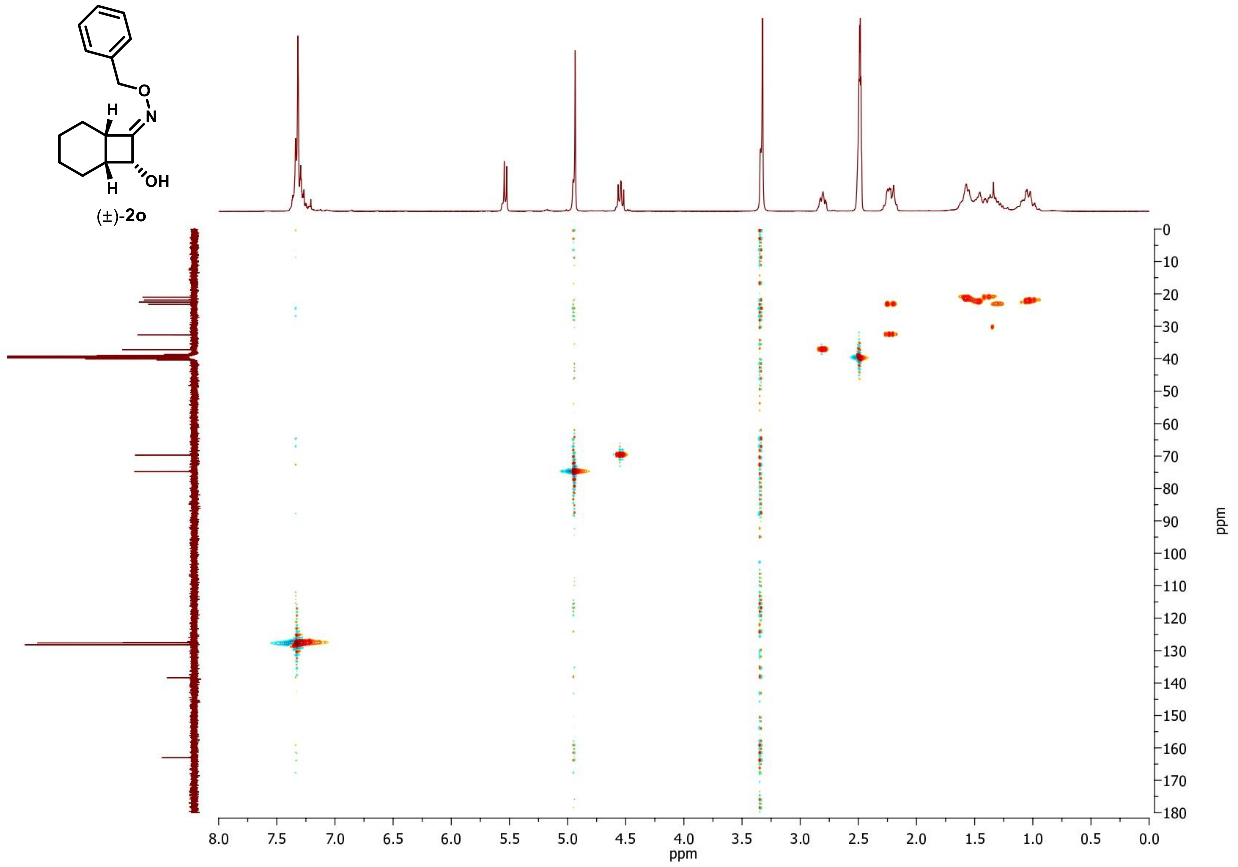

^{1}H-NMR (300 MHz, DMSO-d_6)

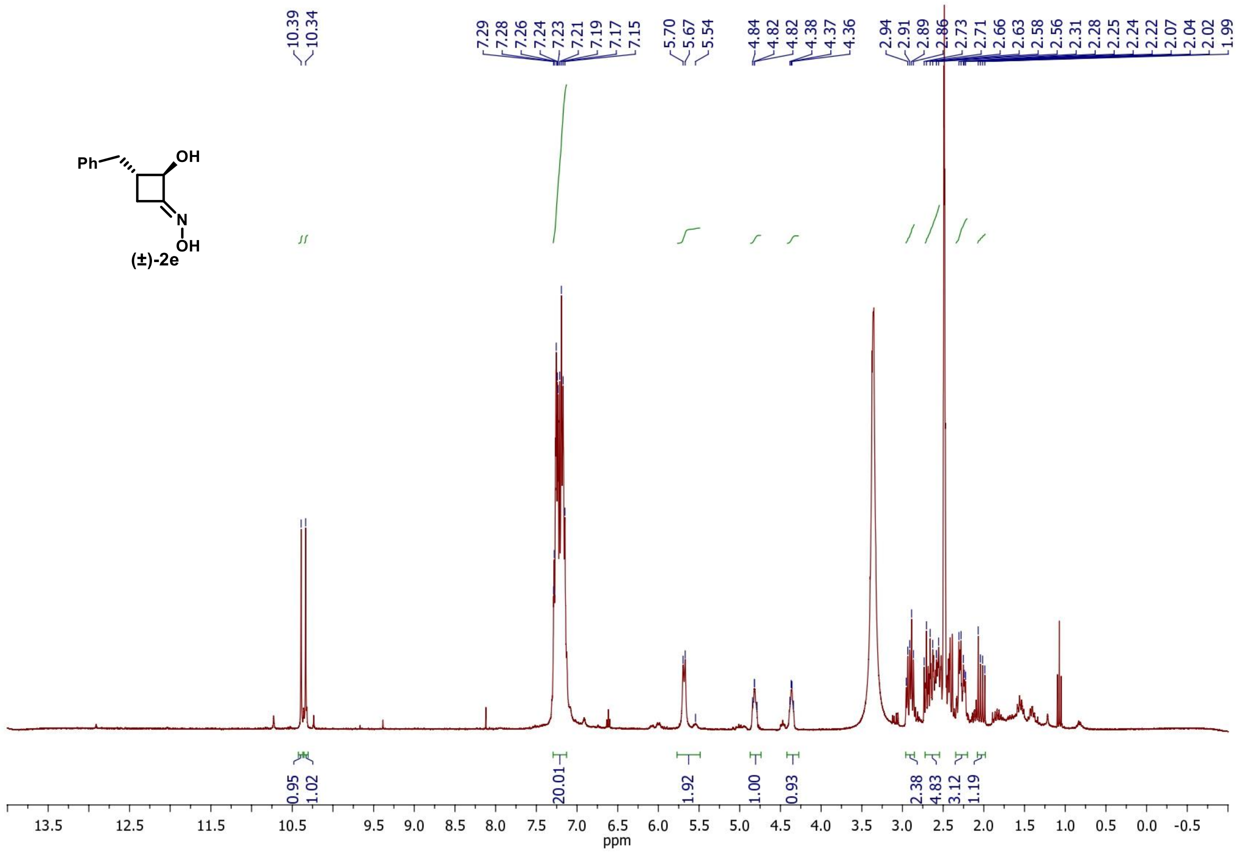

^{13}C{^{1}H}-NMR (75 MHz, DMSO-d_6)

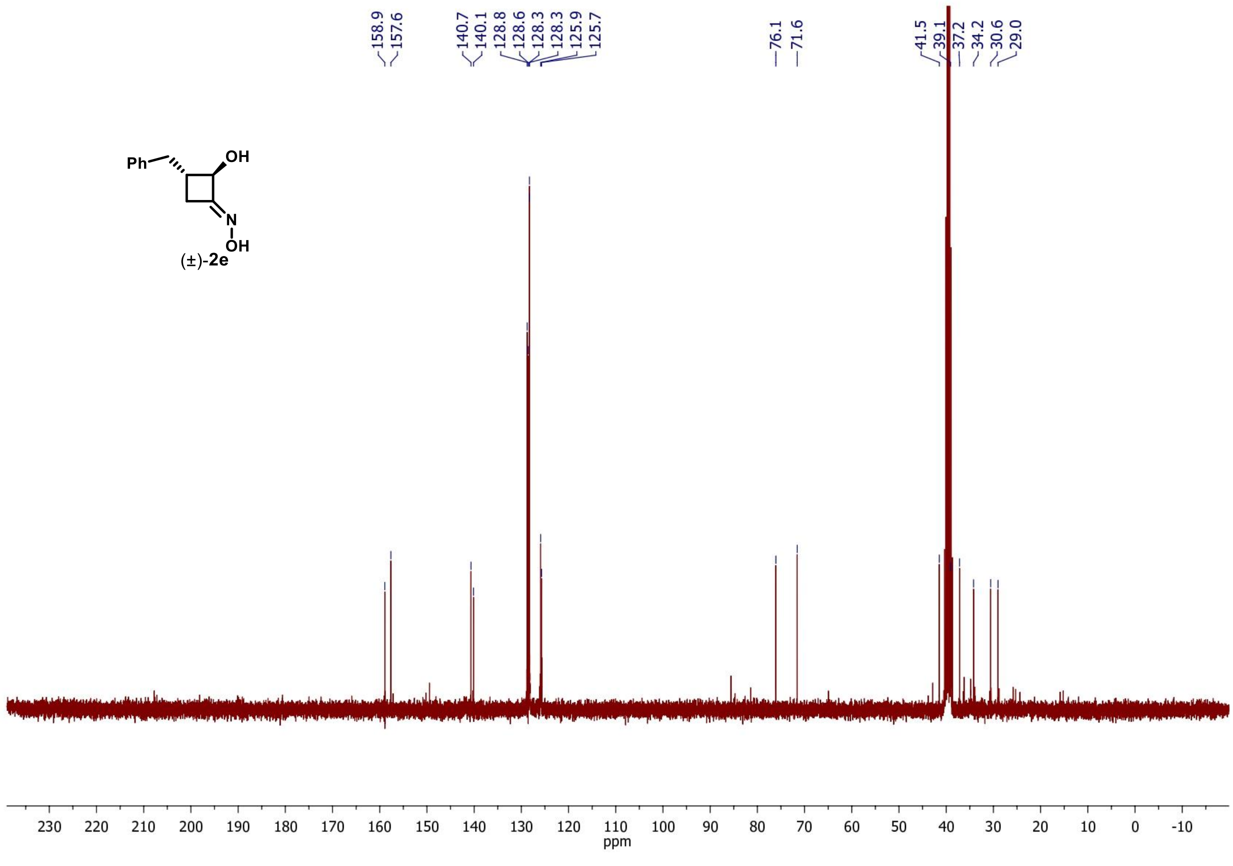

^{1}H-NMR (300 MHz, DMSO-d_6)

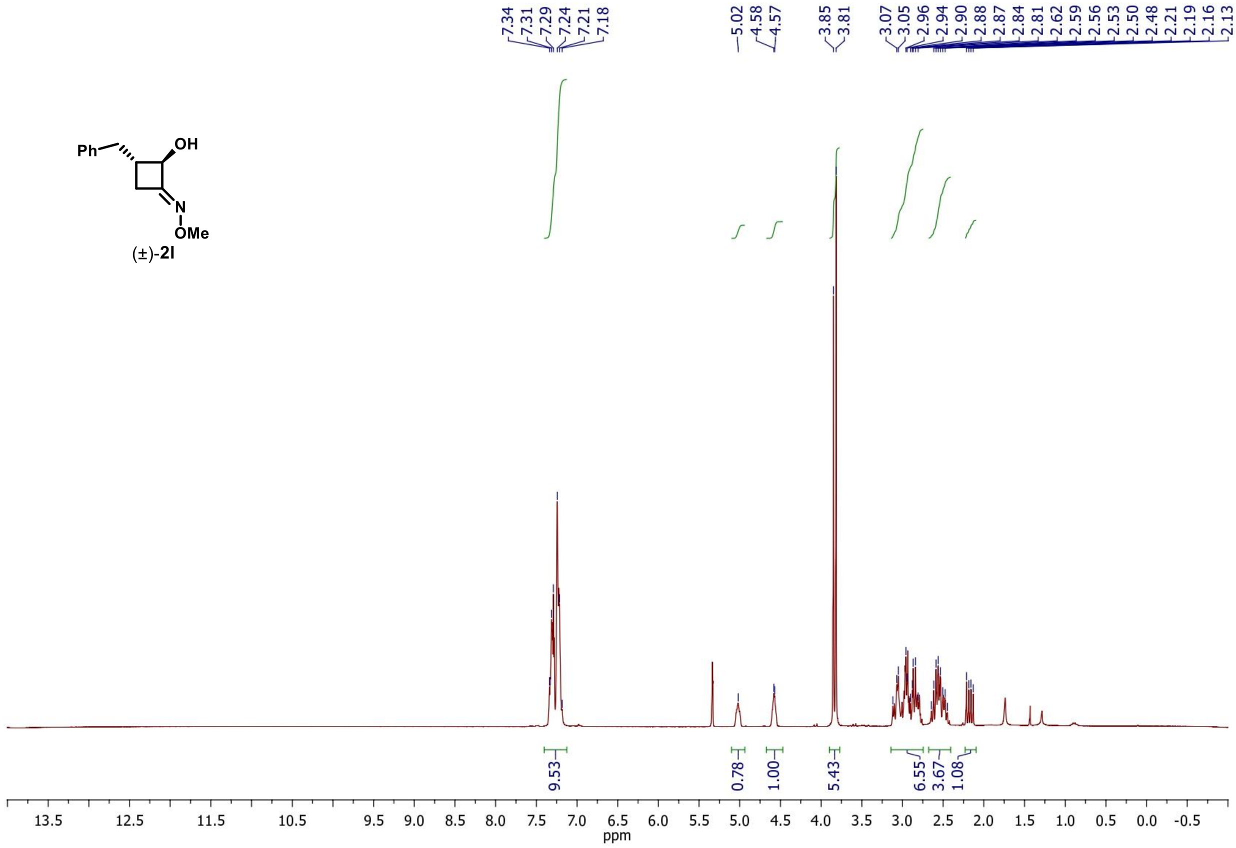

^{13}C{^{1}H}-NMR (75 MHz, DMSO-d_6)

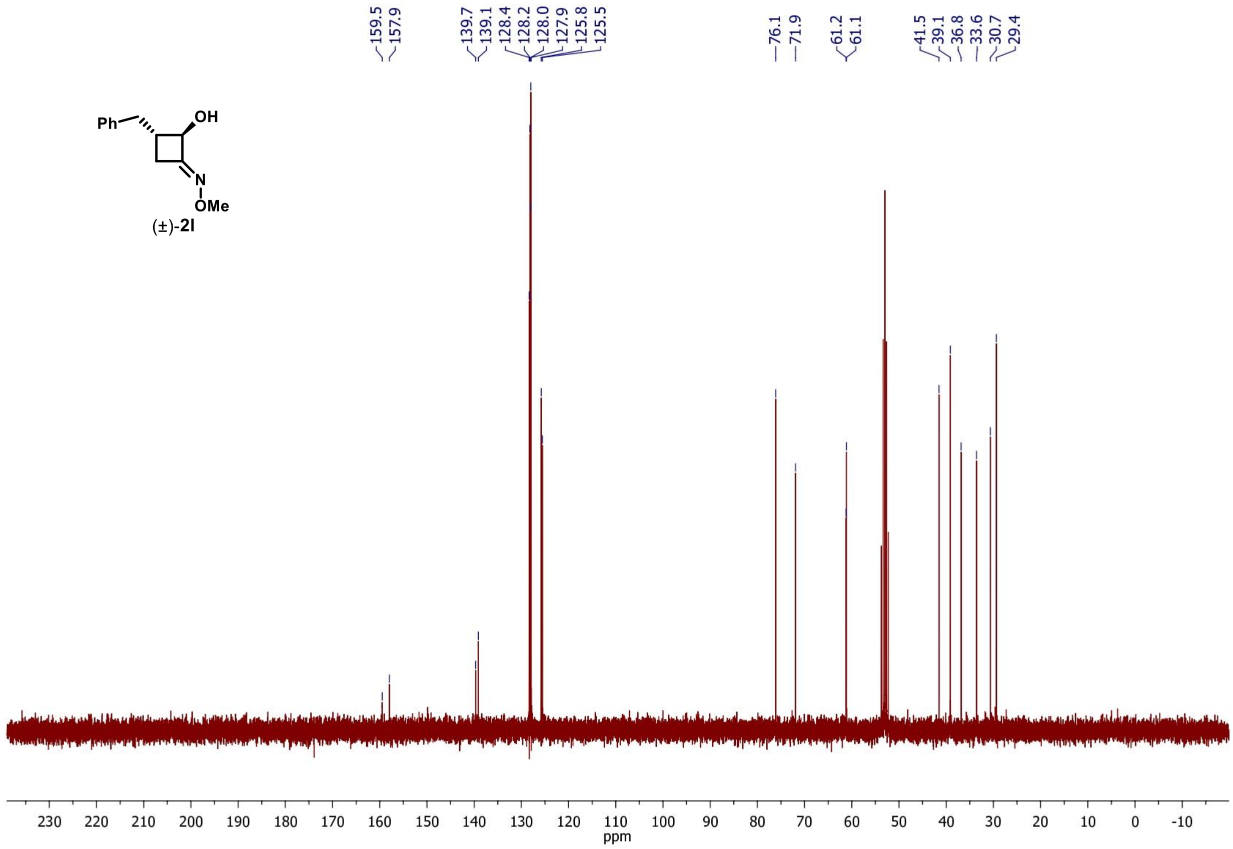

^{1}H-NMR (300 MHz, DMSO-d_6)

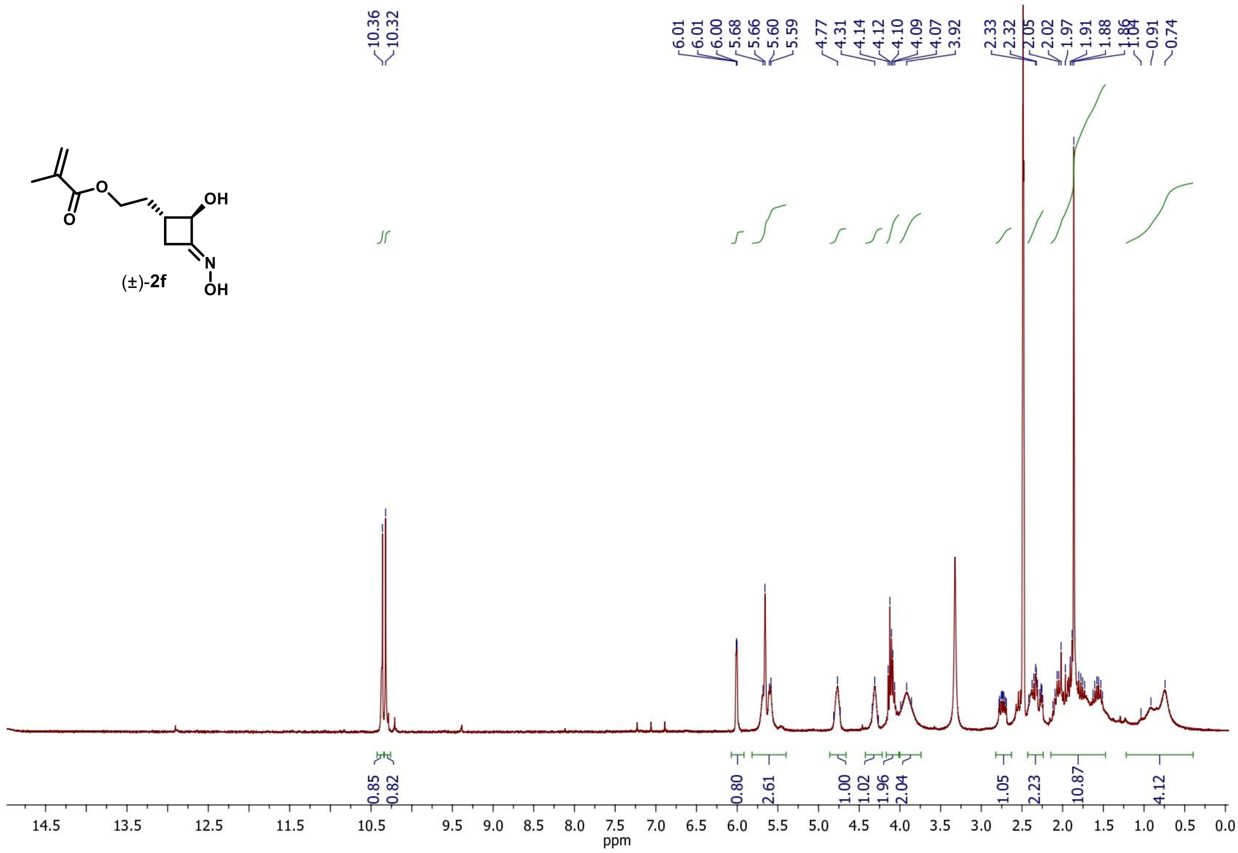

^{13}C{^{1}H}-NMR (75 MHz, DMSO-d_6)

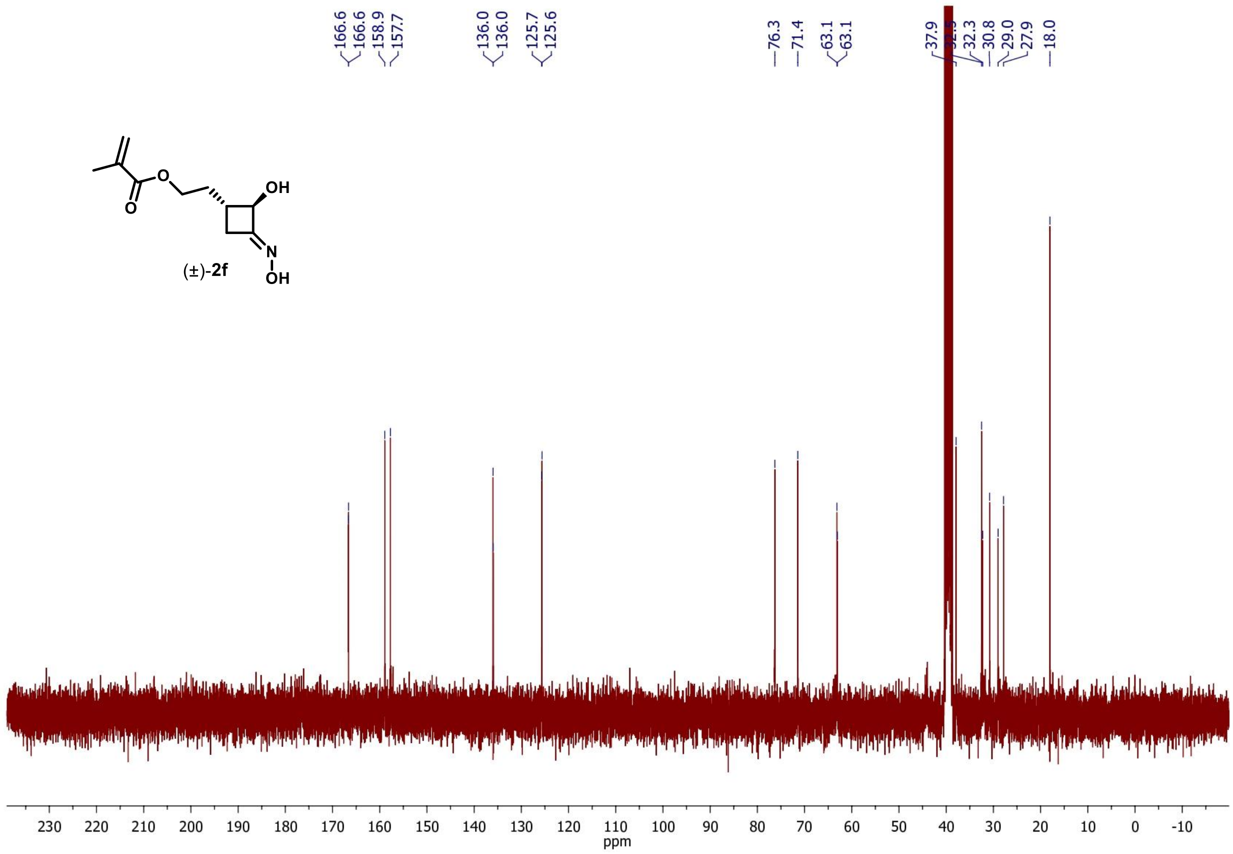

^{1}H-NMR (300 MHz, DMSO-d_6)

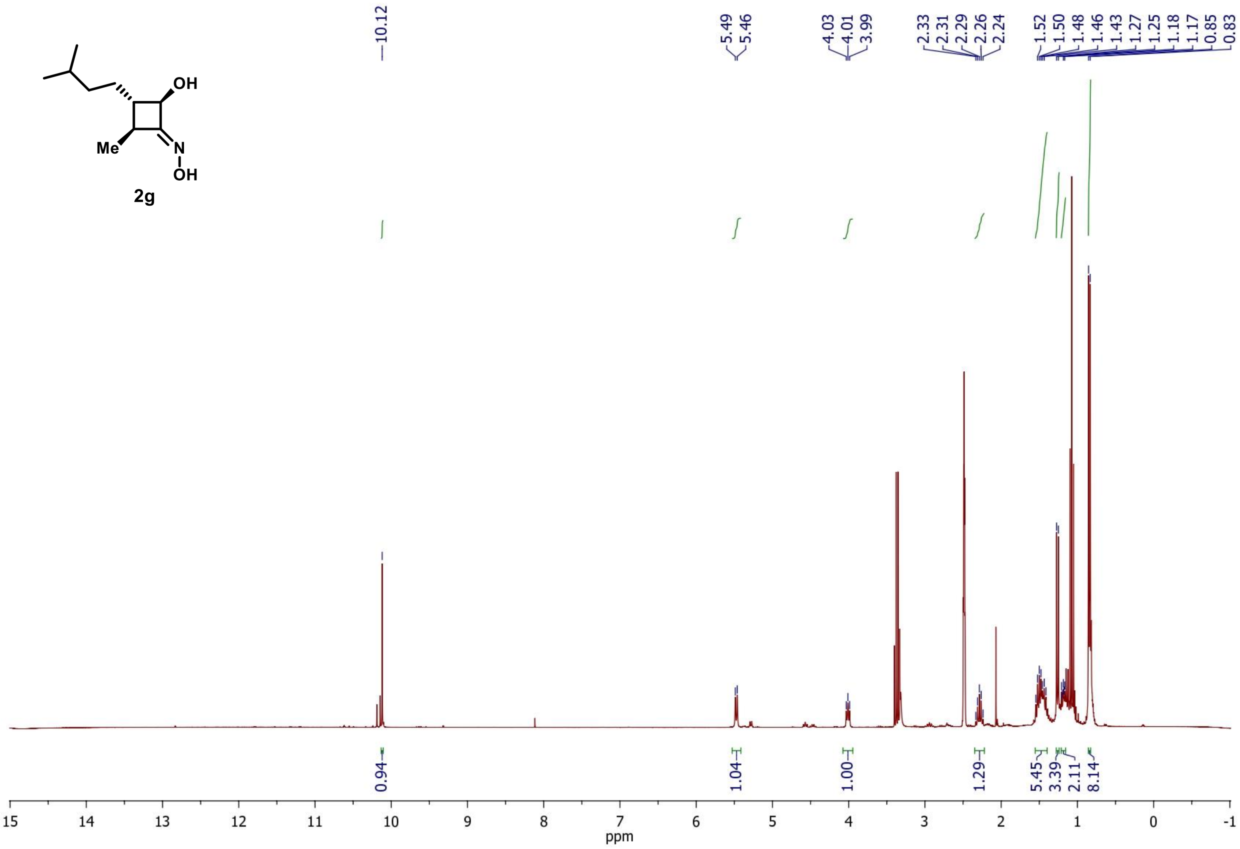

$^{13}C\{^{1}H\}$-NMR (75 MHz, DMSO-d_6)

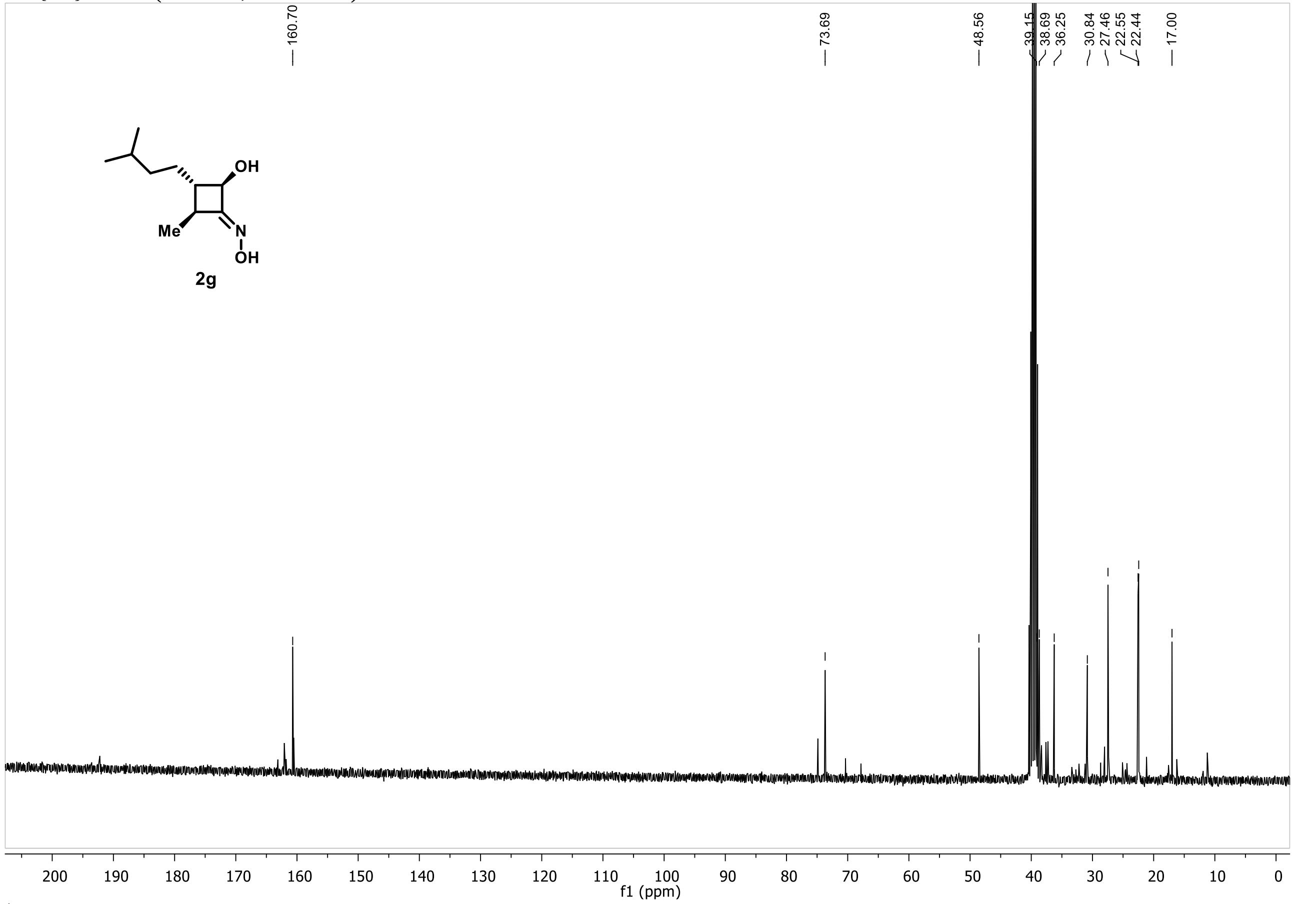

^{1}H-NMR (300 MHz, CD_2Cl_2)

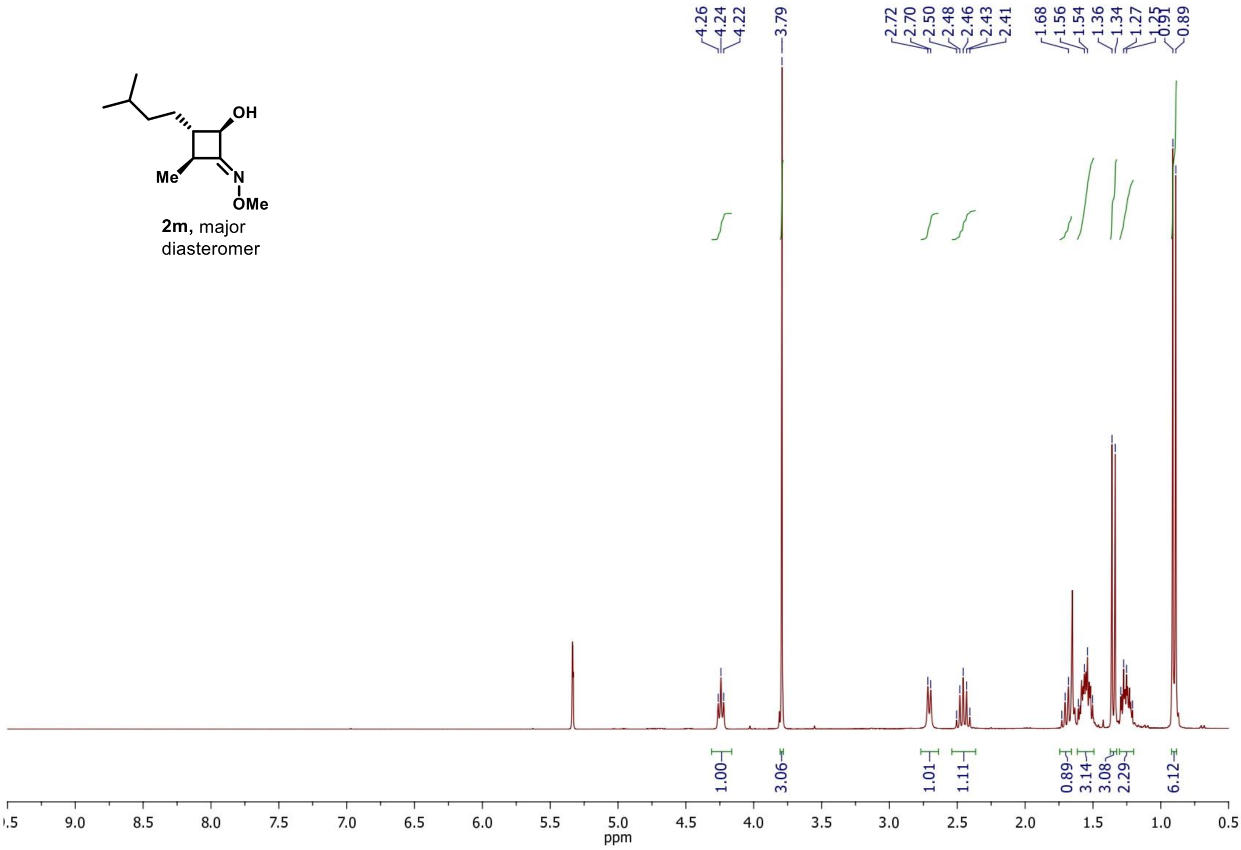

$^{13}C\{^{1}H\}$-NMR (75 MHz, CD_2Cl_2)

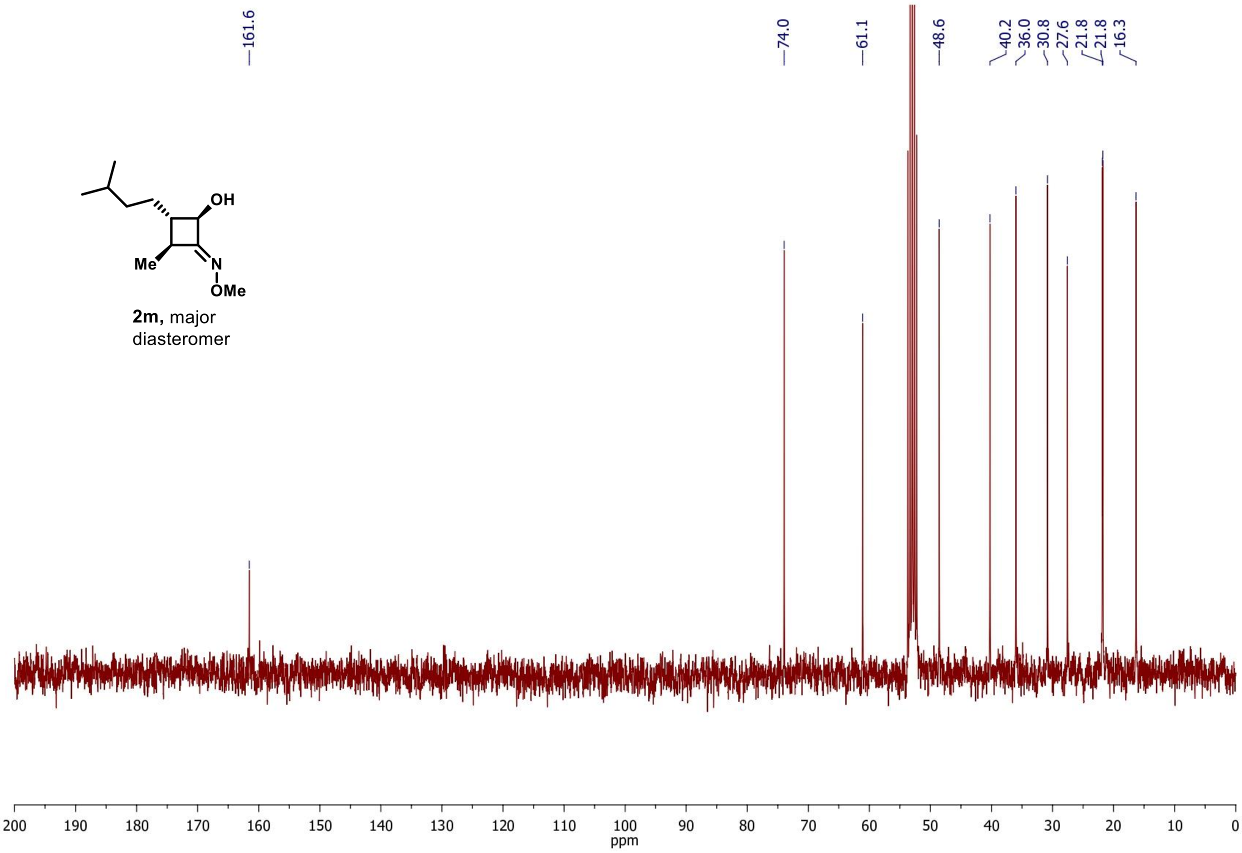

NOESY (300 MHz, CD_2Cl_2)

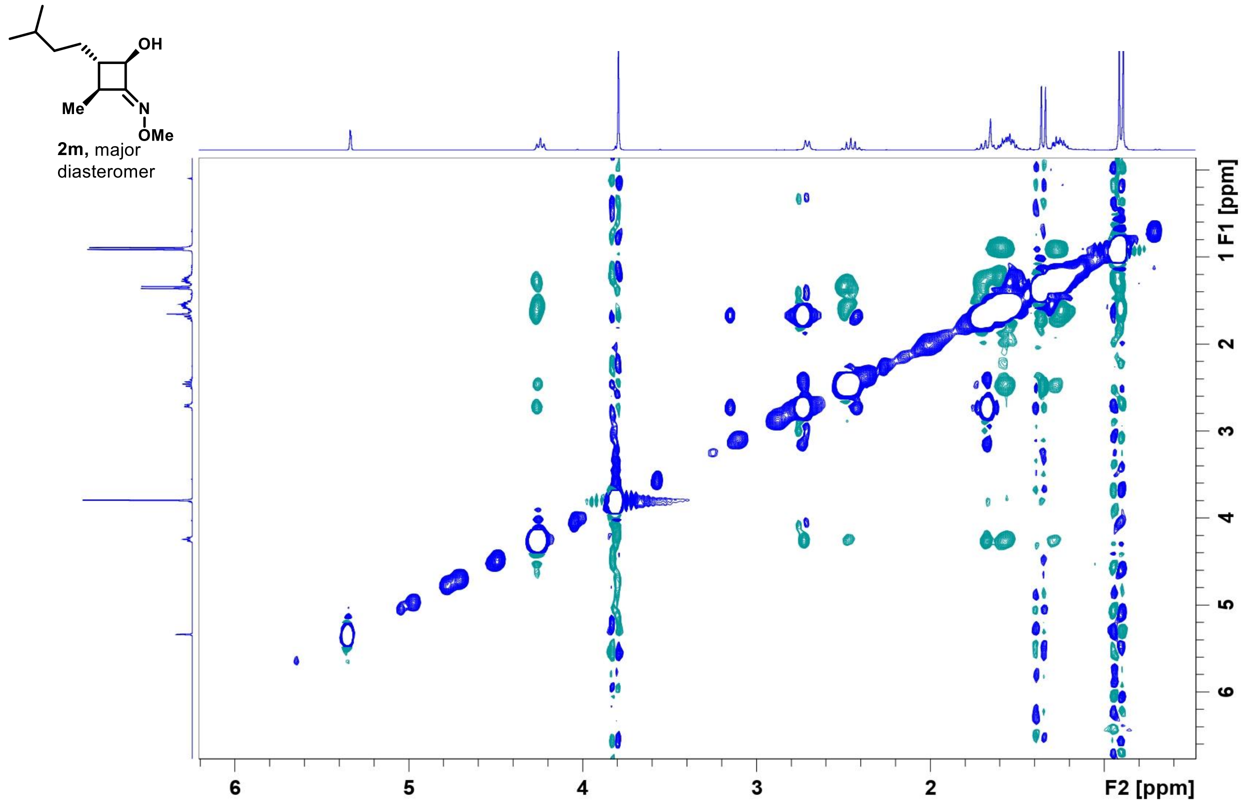

HSQC (300 MHz, CD_2Cl_2)

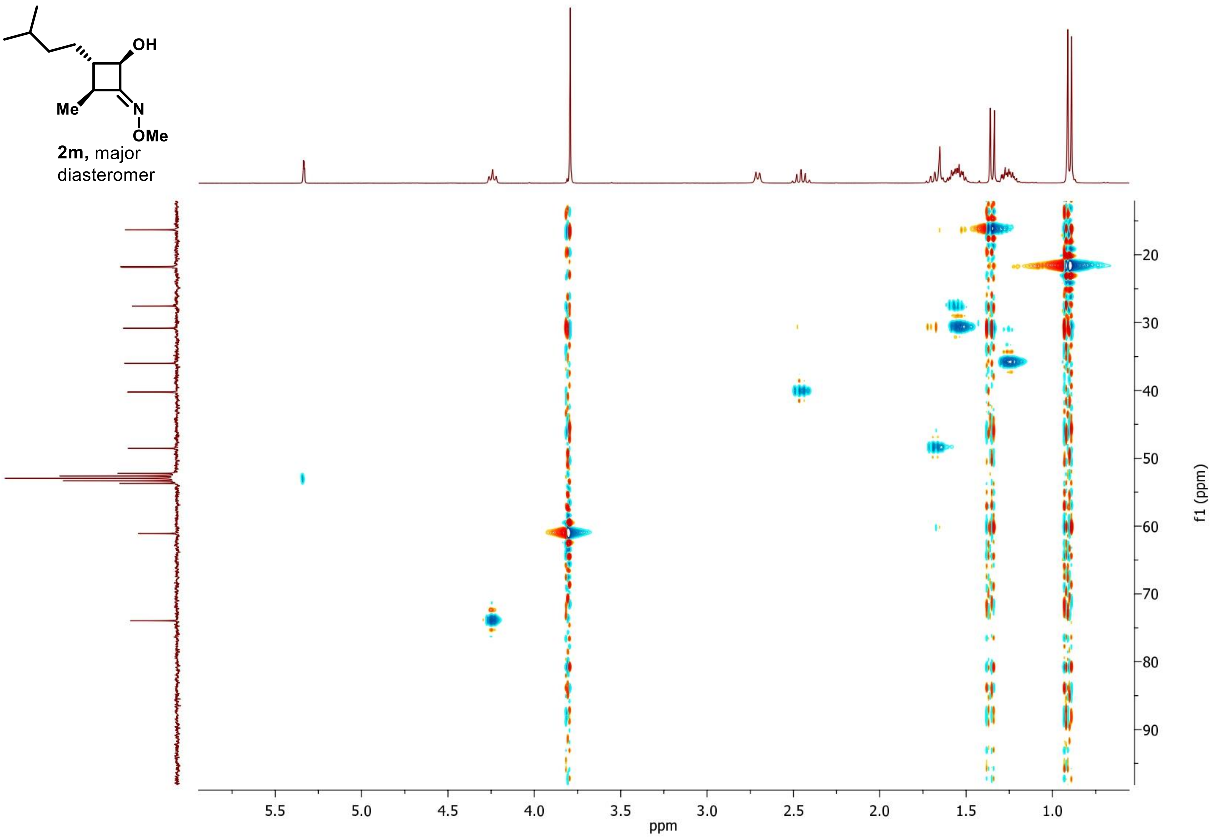

^{1}H-NMR (300 MHz, CD_2Cl_2)

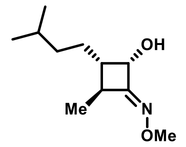

2m, minor
diastereomer

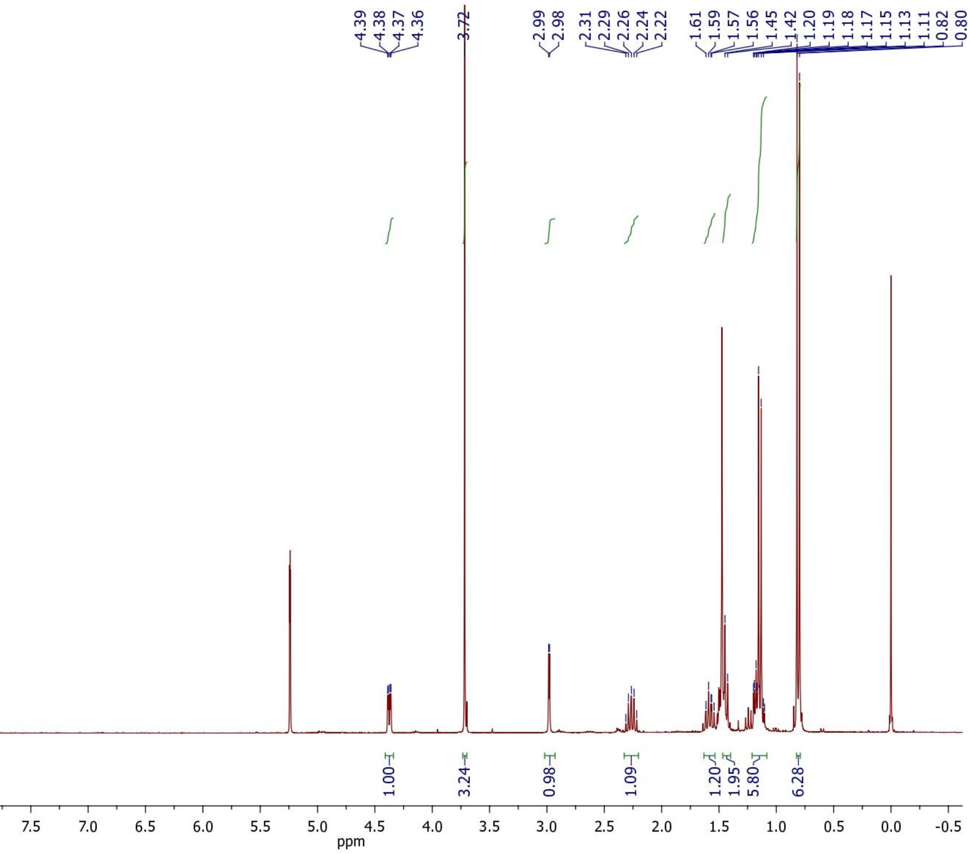

$^{13}C\{^1H\}$-NMR (75 MHz, CD_2Cl_2)

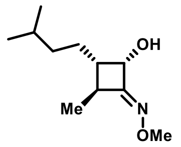

2m, minor
diastereomer

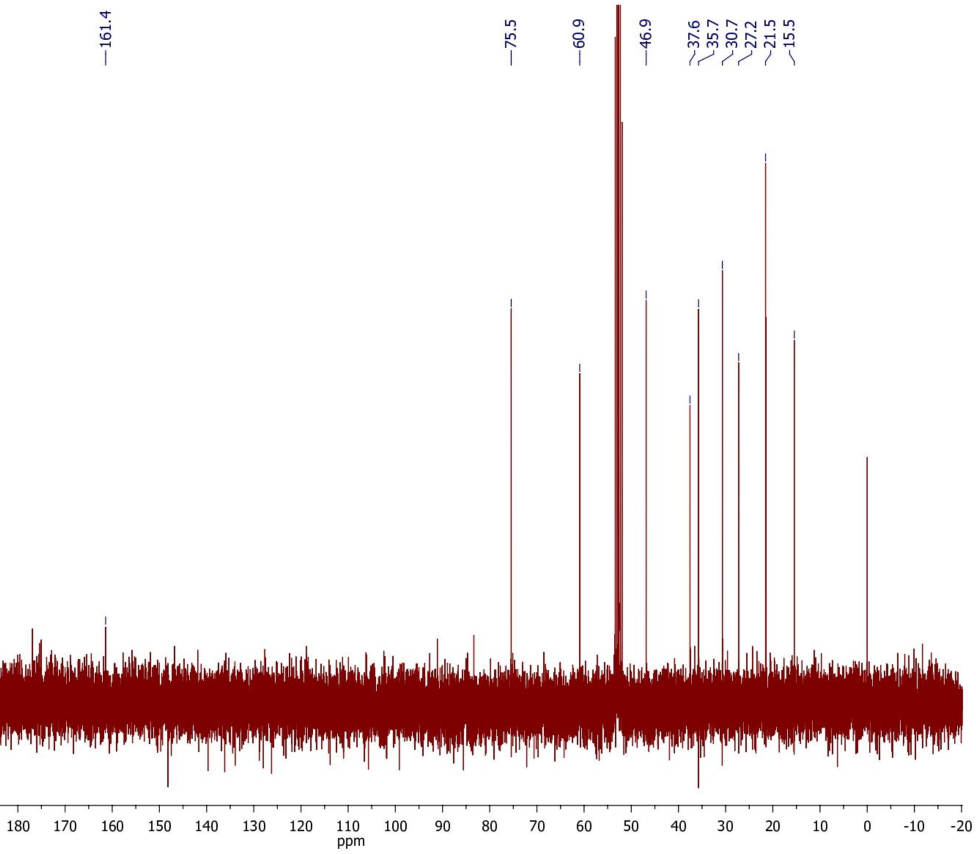

NOESY (300 MHz, CD_2Cl_2)

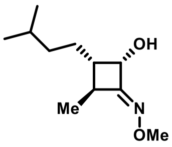

2m, minor diastereomer

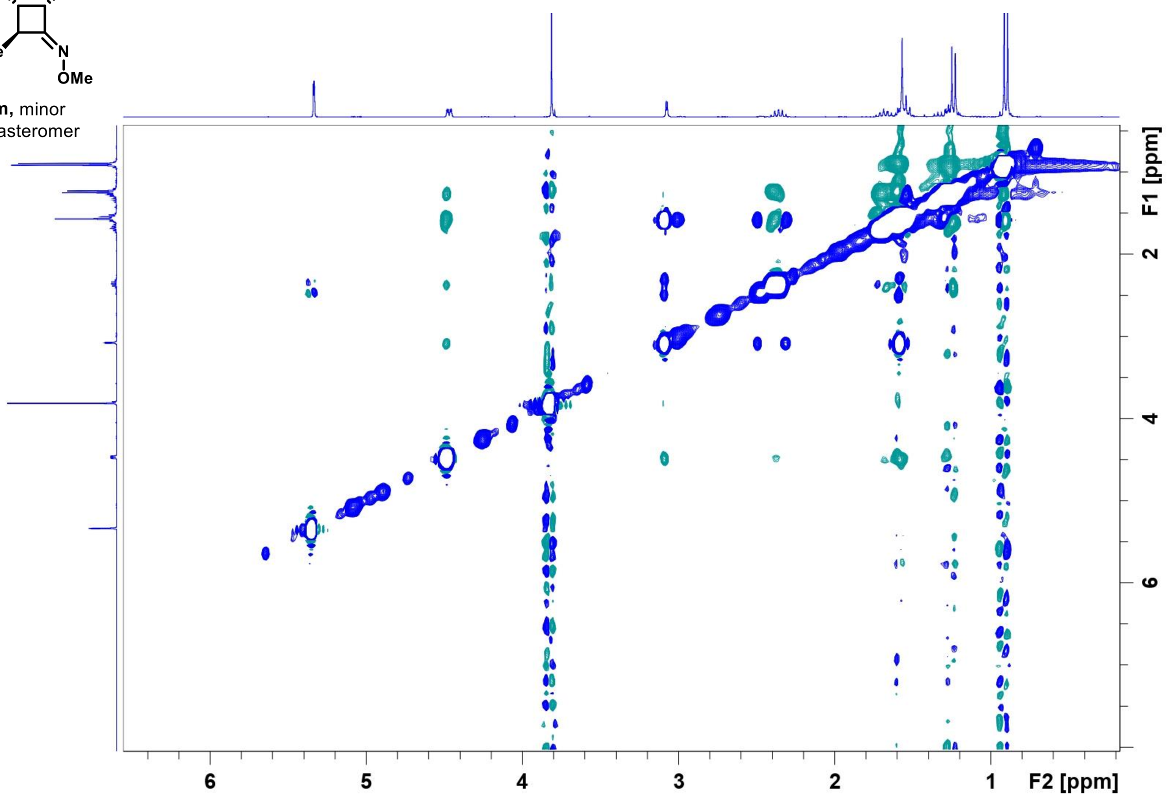

HSQC (300 MHz, CD_2Cl_2)

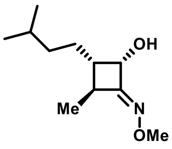

2m, minor diastereomer

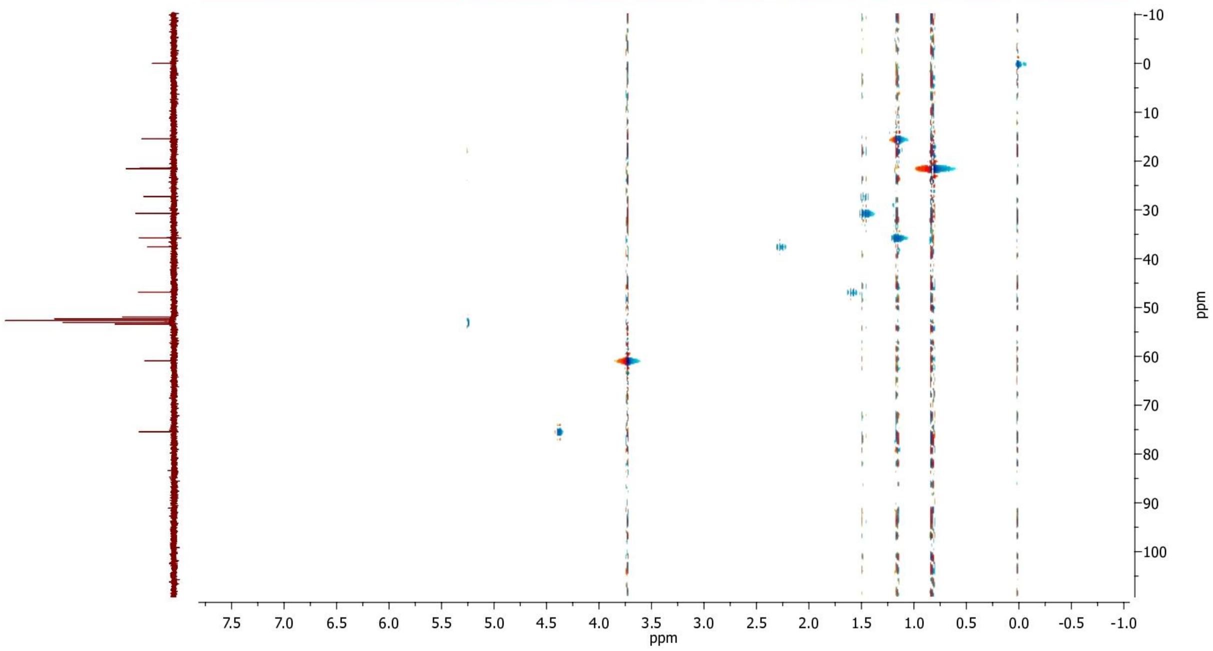

3.1 NOESY contacts relevant to the determination of the relative configuration of CBOs 2j, 2n and 2o.

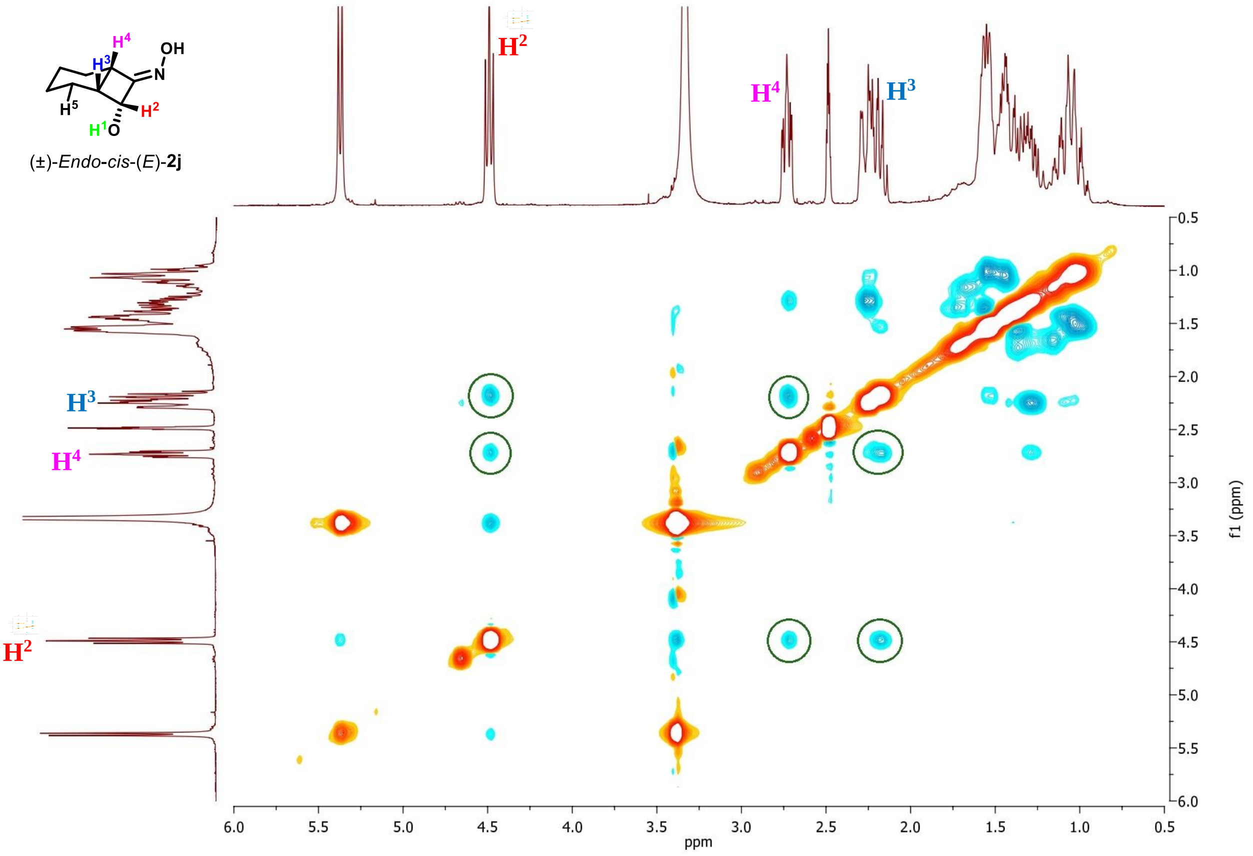

Figure S2. NOESY contacts between cyclobutane ring protons of CBO (±)-*Endo-cis*-(*E*)-**2j.**

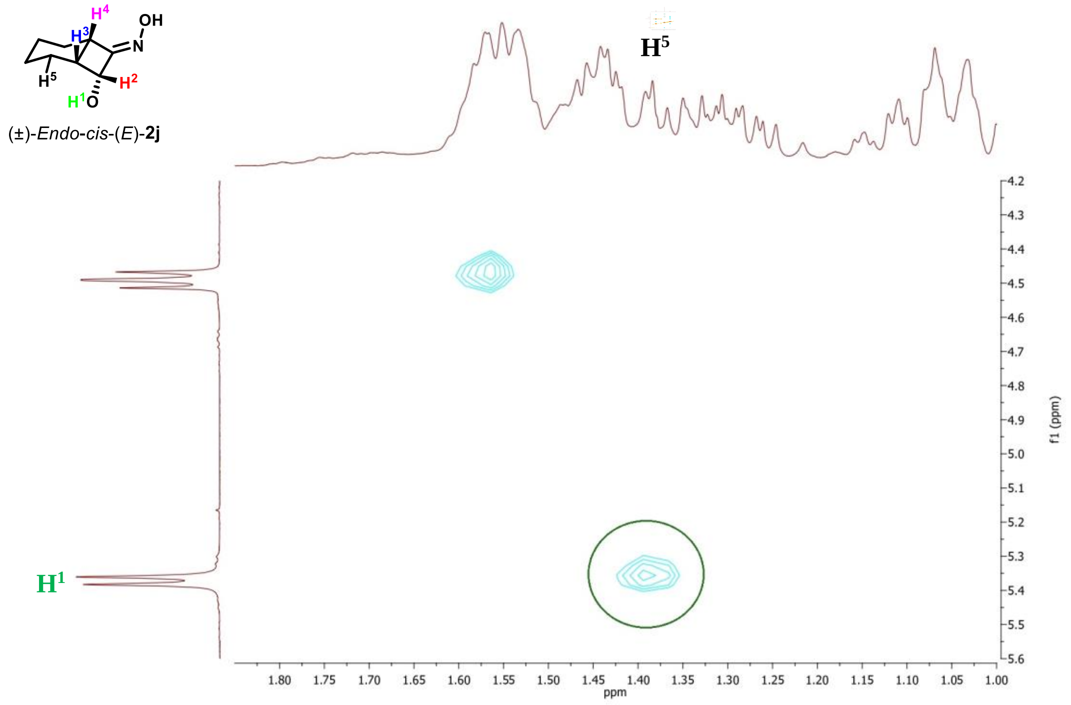

Figure S3. NOESY contacts between alcoholic and alkylic protons of CBO (±)-*Endo-cis*-(*E*)-**2j.**

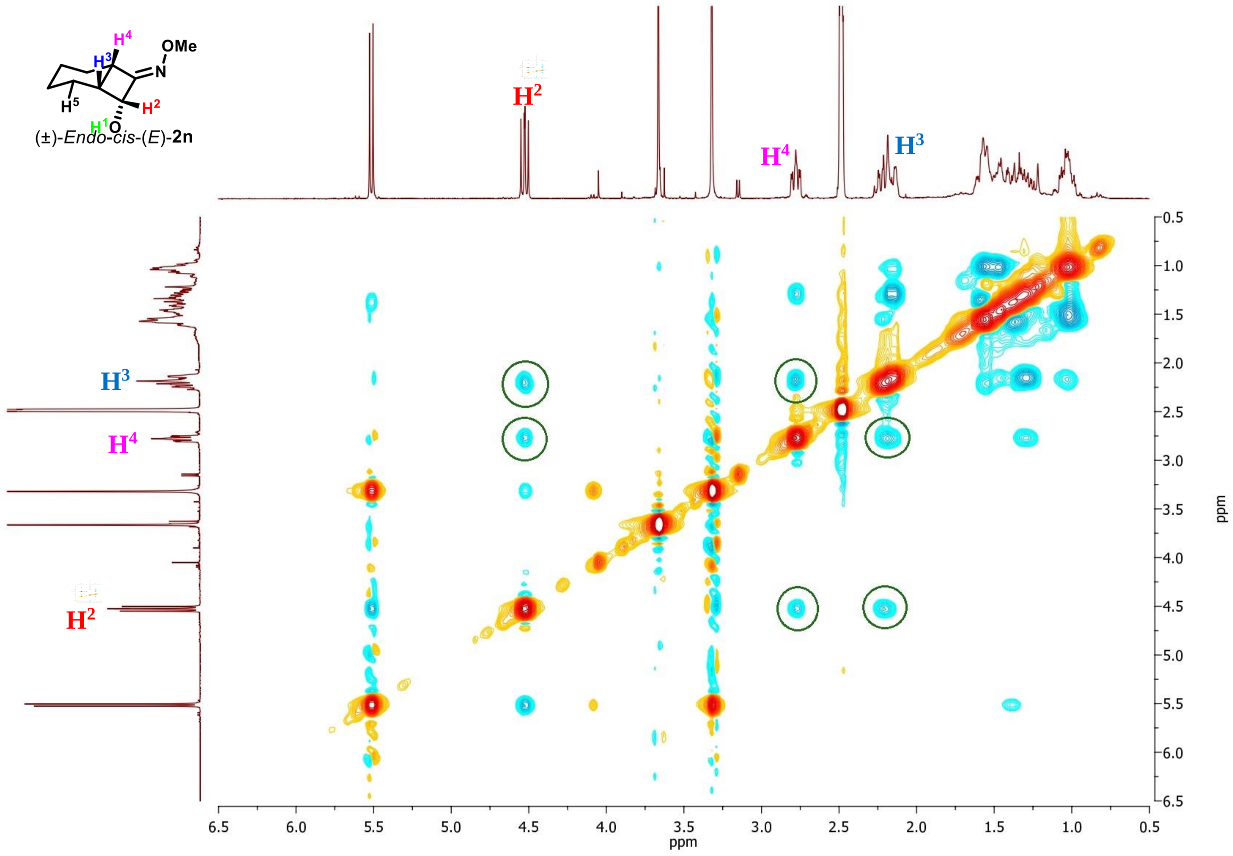

Figure S4. NOESY contacts between cyclobutane ring protons of CBO (±)-*Endo*-*cis*-(*E*)-**2n.**

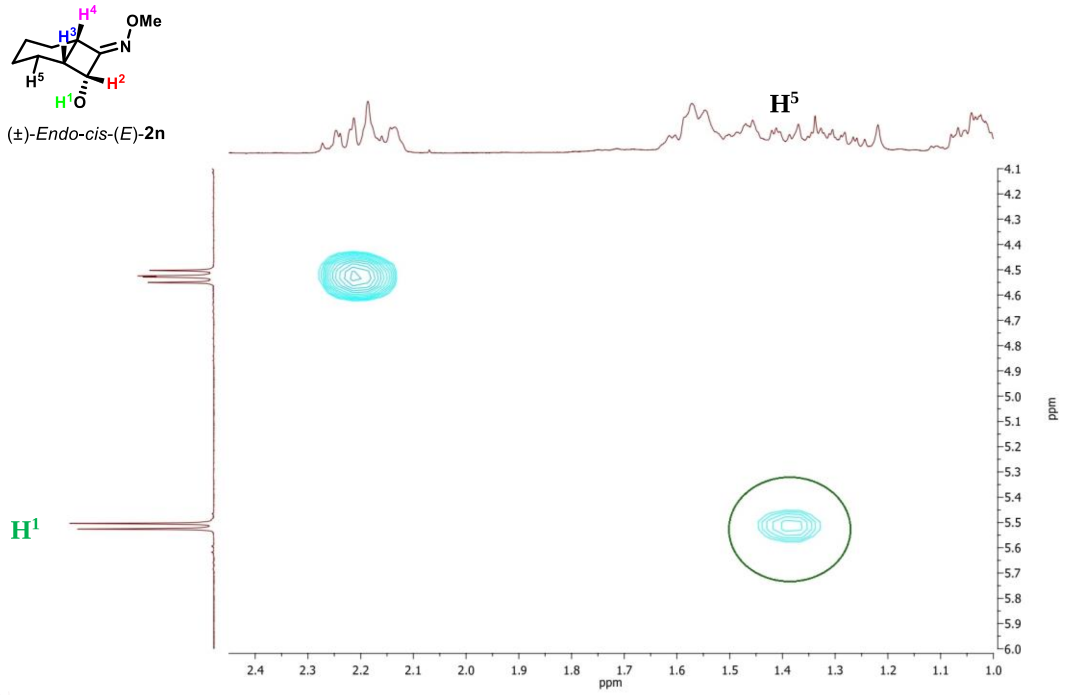

Figure S5. NOESY contacts between alcoholic and alkylic protons of CBO (±)-*Endo*-*cis*-(*E*)-**2n.**

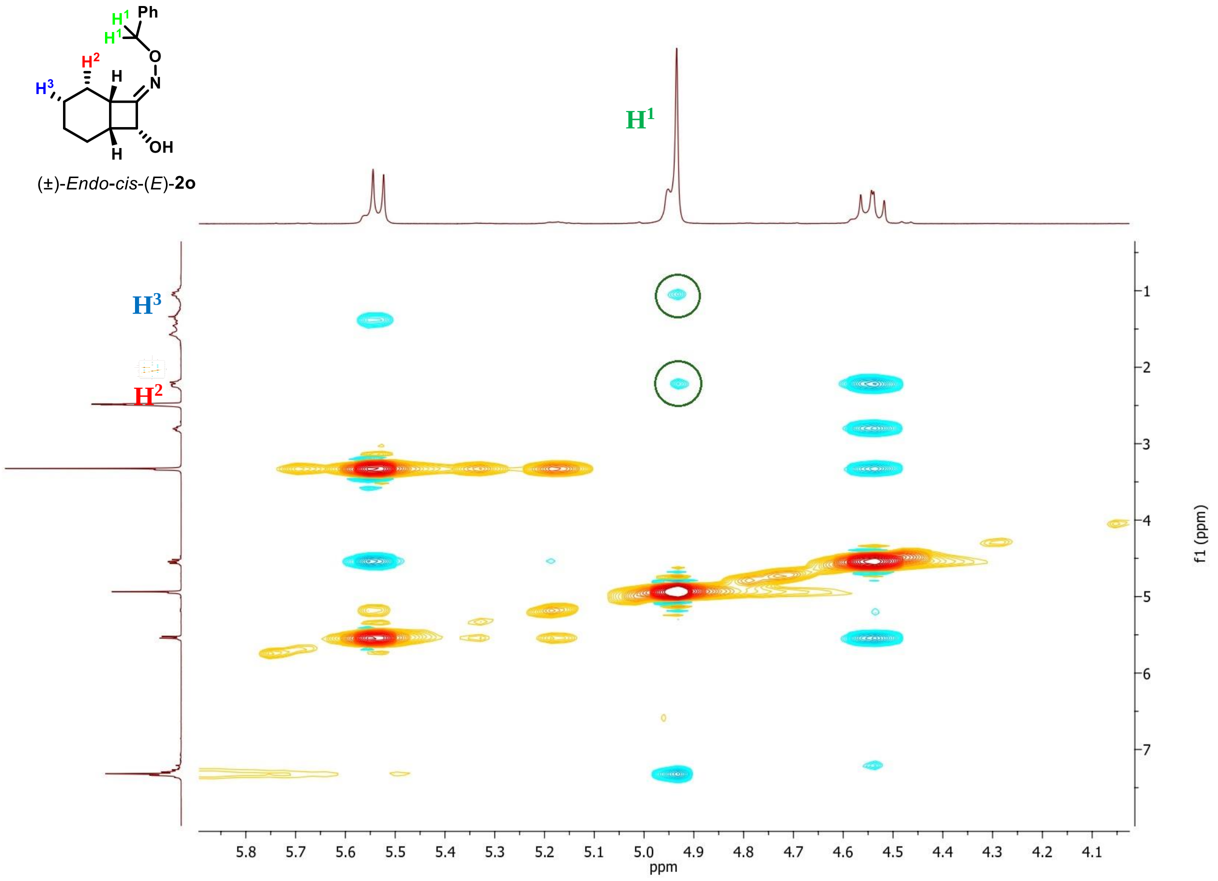

Figure S6. NOESY contacts between benzylic and alkylic protons of CBO (±)-*Endo*-*cis*-(*E*)-**2o.**

4. CBO C=N double bond *E/Z* isomerization

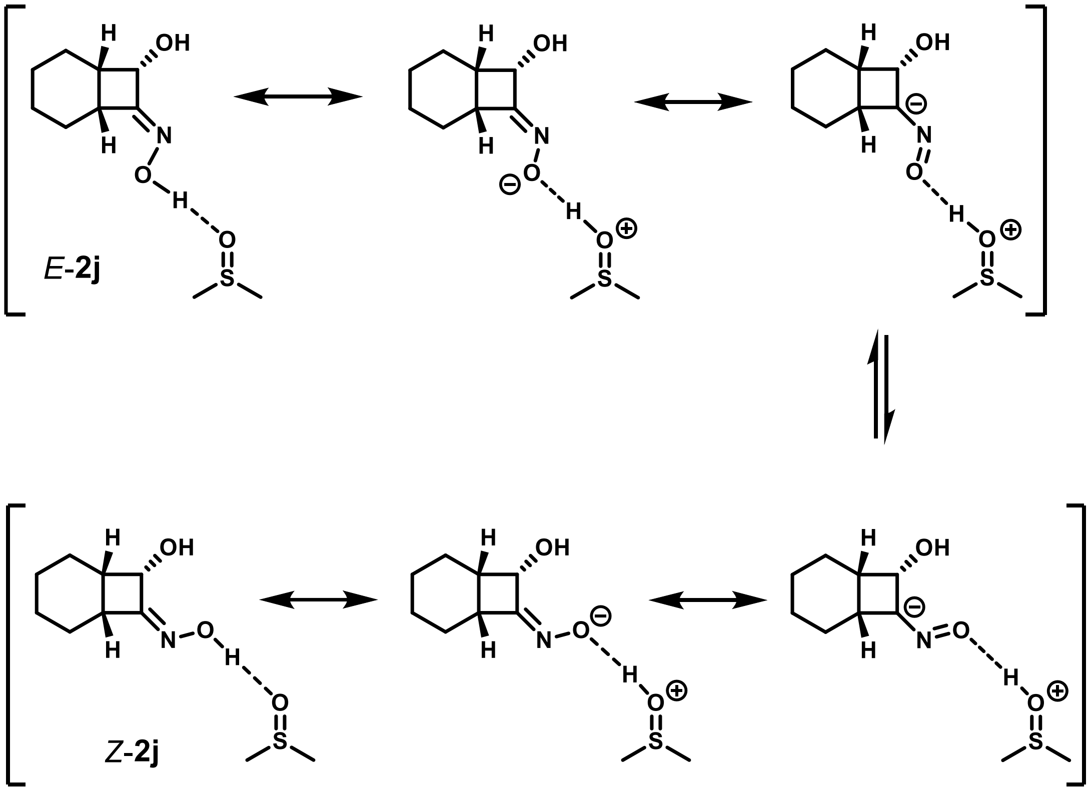

Scheme S1. Suggested mechanism of *E/Z* isomerization of CBO **2e**.

5. Molecular dynamics

5.1 Methods

The simulation box consists of a single oxime molecule surrounded by 163 solvent molecules. Different oxime isomers were here considered: *cis-endo-E*-**2j**, *cis-endo-Z*-**2j**, *cis-exo-E*-**2j**, *cis-exo-E*-**2j** as well as a *cis-endo-E*-**2i** CBOs. Simulations are carried out using leap-frog algorithm with a time-step of 0.5 fs. UFF force field is adopted for the purpose[14]. Long range electrostatic interactions are considered using PME method while a cut-off scheme with cut-off distance 1 nm is applied for Van der Waals interactions. Initial configuration minimization is carried out using conjugate gradient method; further MD simulations lasting 90 ns were performed for each system in order to guarantee equilibrium. Canonical ensembles (NVT) are obtained through V-rescale thermostat with coupling constant τ_T=0.1 ps and reference temperature 298.15 K. Periodic boundary conditions were applied in the three spatial directions. Data were collected during 30000000 step lasting simulations (20000000 in the case of DCM).

5.2 Discussion

In order to better highlight the crucial role of the solvent polarity in oxime double bond isomerization, the radial distribution functions (RDFs) of DCM chlorine with respect to the oxime oxygen for both *cis-endo-E*-**2j** and *cis-endo-Z*-**2j** isomers is reported in Figure S7. In the case of DCM solvent, we did not observe the appreciable differences between the two curves as in the case of DMSO solvent (Figure 8). The lower intensity of the radial distribution functions in Figure S8 suggests a weaker interaction of the alcoholic OH group with the DMSO.

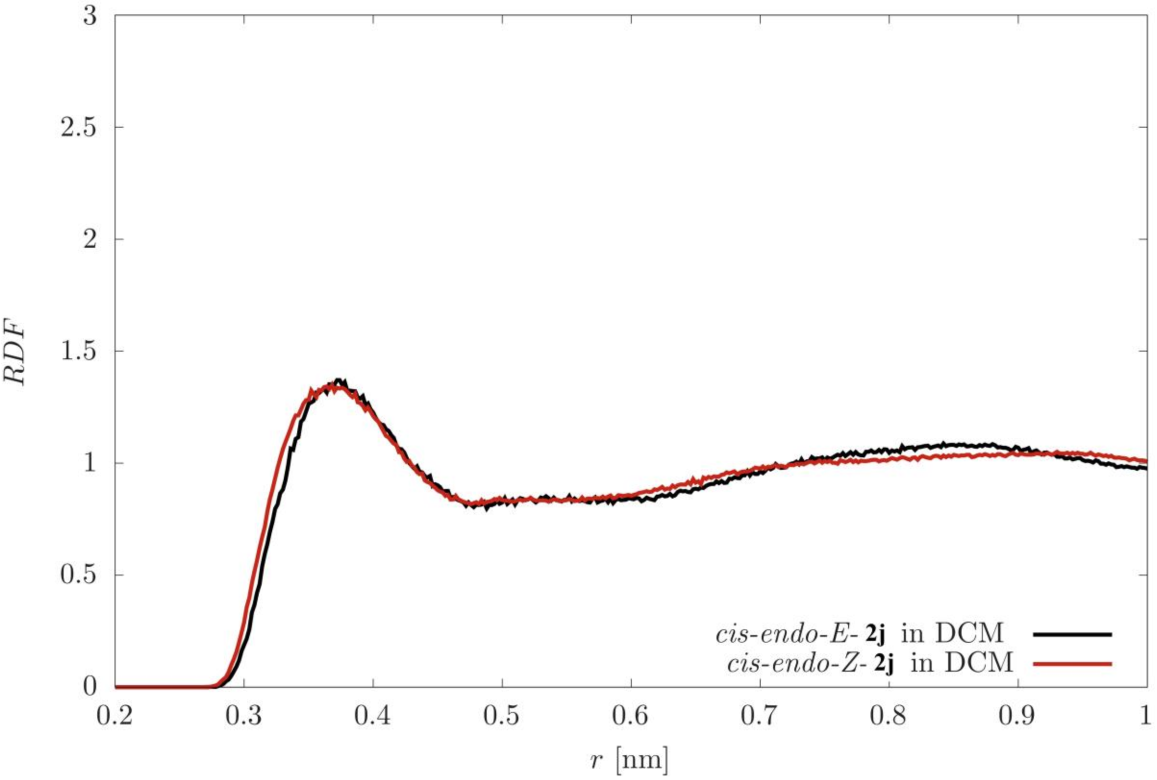

Figure S7. Radial distribution functions related to the interaction between CBO oxime oxygen and chlorine in DCM solvent.

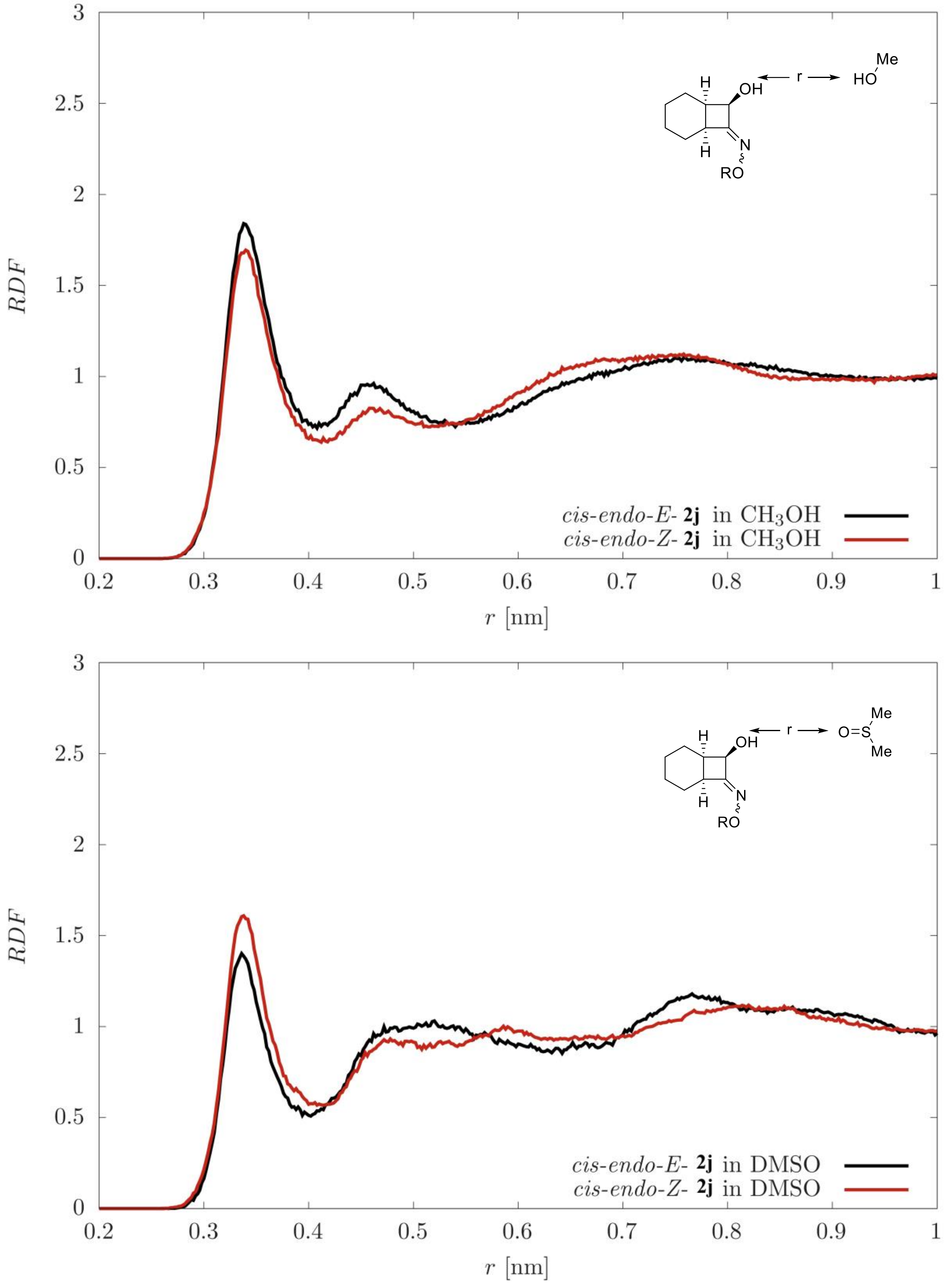

Figure S8. Radial distribution functions related to the interaction between CBO alcoholic oxygen and oxygen in DMSO and methanol.

In Figure S10, distribution function of the dihedral angle θ (defined by atoms 24-21-8-5, Figure S9) related to alcoholic OH group rotating around the oxygen-carbon bond of *cis*-*endo*-*E*-**2j** is reported. This distribution suggests that the most probable dihedral angles correspond to the conformations (θ = –89 and θ = 156), which have the alcoholic OH group near to the alkylic H (20, Figure S9). This observation is compatible with the

observed contact in the NOESY experiment in Figure S3 and the distance distribution function reported in Figure S11.

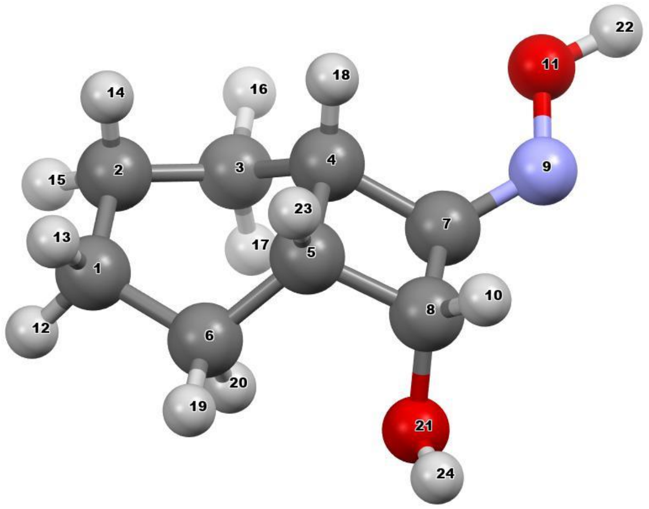

Figure S9. Snapshot of *cis*-*endo*-*E*-**2j** isomer with dihedral angle (defined by atoms 24-21-8-5) corresponding to θ = –95° in Figure S5.

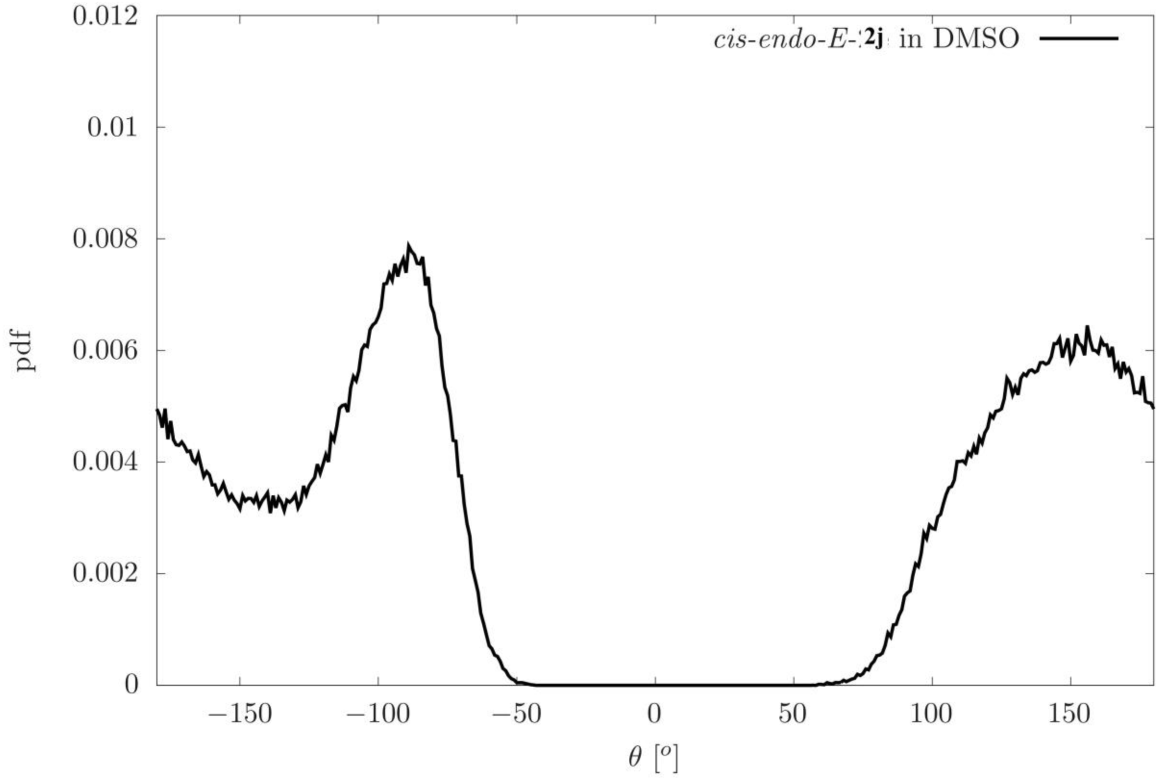

Figure S10. Distribution function of the dihedral angle θ (defined by atoms 24-21-8-5 in Figure S4) related to alcoholic OH group rotating around the oxygen-carbon bond of *cis*-*endo*-*E*-**2j**. θ = 0 corresponds to *syn* conformation. Positive angles define the counterclockwise rotation of the OH group.

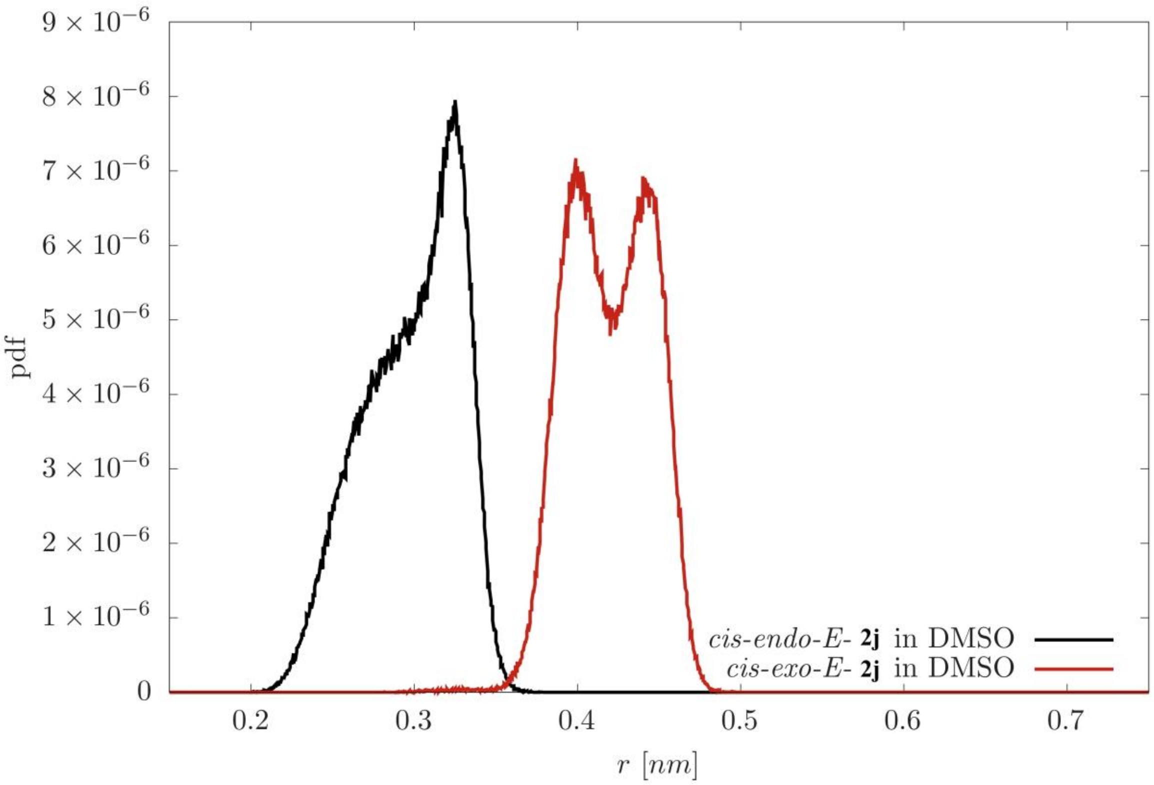

Figure S11. Distance distribution function between alcoholic H (atom 24 in Figure S4) and alkylic hydrogen (atom 20 in Figure S4) of *cis*-*endo*-*E*-**2j** and *cis*-*exo*-*E*-**2j** isomers in DMSO.

6. CID spectra

Collision induced dissociation (CID) on the deprotonated forms [M-H]$^{-}$ with collision energy (CE) 30 V.

(*E*)-2-(Hydroxyimino)-4-phenylbutanal **1a** ($C_{10}H_{11}NO_2$)

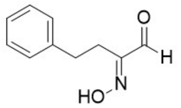

(*E*)-2-(hydroxyimino)-4-phenylbutanal
Chemical Formula: $C_{10}H_{11}NO_2$
Exact Mass: 177,0790

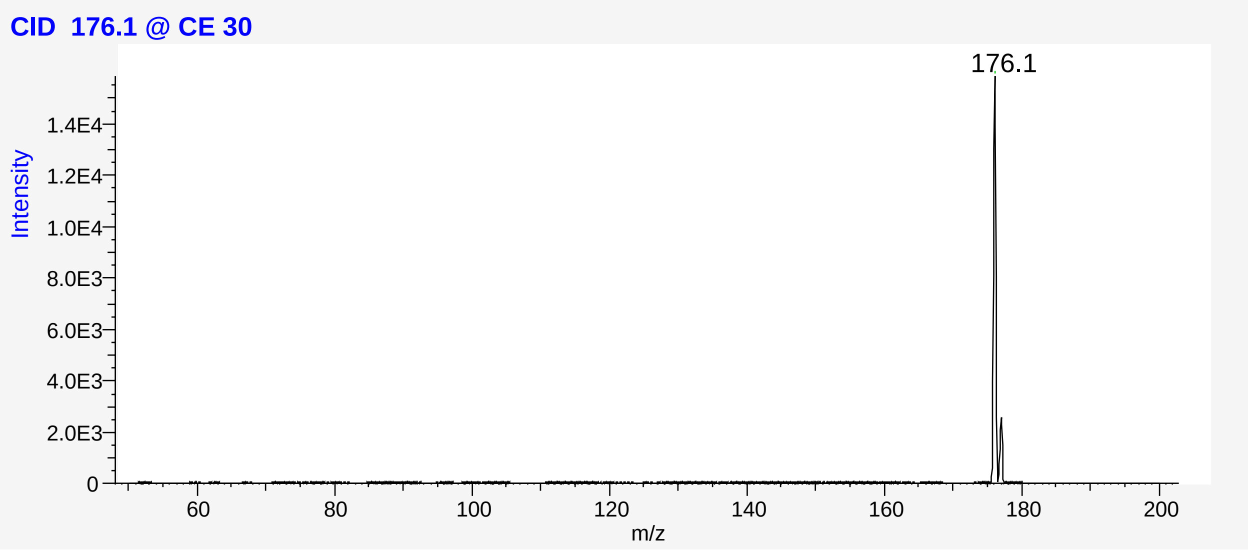

(*E*)-2-(Hydroxy)-3-phenylcyclobutan-1-one oxime **2a** ($C_{10}H_{11}NO_2$)

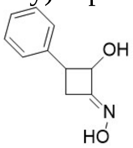

(*E*)-2-hydroxy-3-phenylcyclobutan-1-one oxime
Chemical Formula: $C_{10}H_{11}NO_2$
Exact Mass: 177,0790

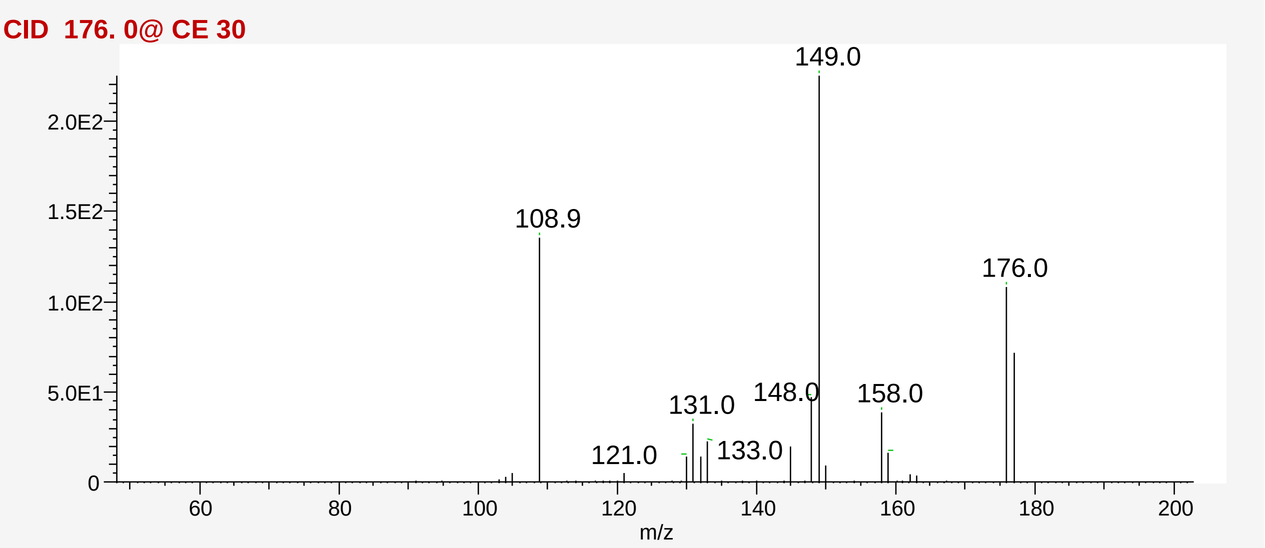

Collision induced dissociation (CID) were carried on the protonated forms $[M+H]^+$ with collision energy 12 V.

(*E*)-2-((Benzyloxy)imino-2-cyclohexylethanal **1j** ($C_{15}H_{19}NO_2$)

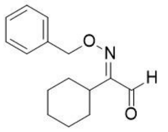

(*E*)-2-((benzyloxy)imino)-2-cyclohexylacetaldehyde
Chemical Formula: $C_{15}H_{19}NO_2$
Exact Mass: 245,1416

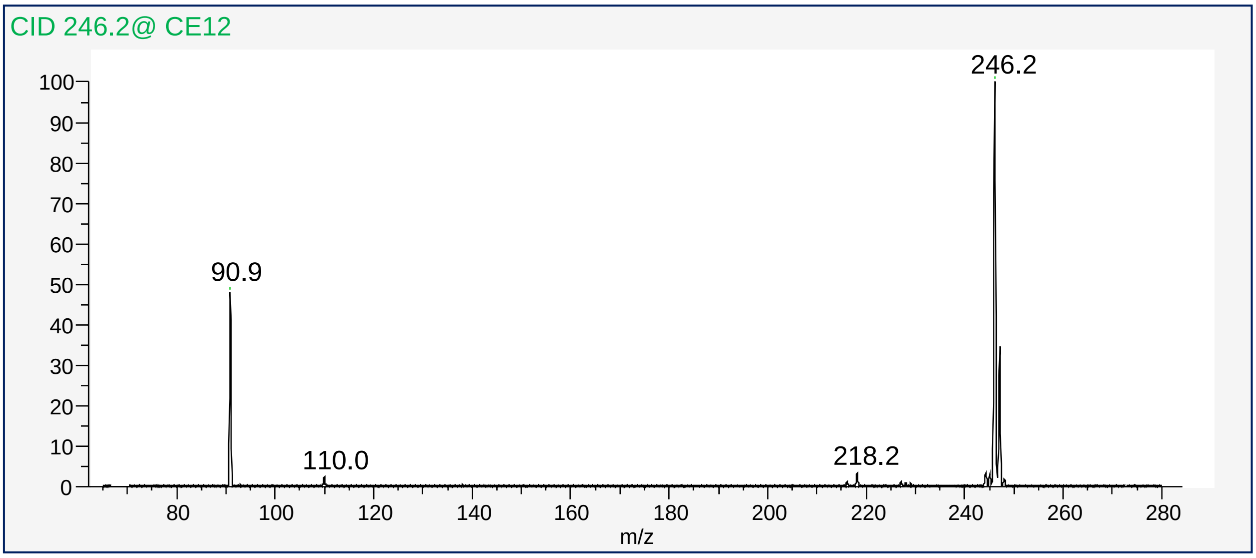

(*E*)-8-Hydroxybicyclo[4.2.0]octan-7-one *O*-benzyloxime **2j** ($C_{15}H_{19}NO_2$)

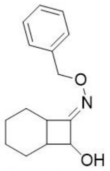

(*E*)-8-hydroxybicyclo[4.2.0]octan-7-one *O*-benzyl oxime
Chemical Formula: $C_{15}H_{19}NO_2$
Exact Mass: 245,1416

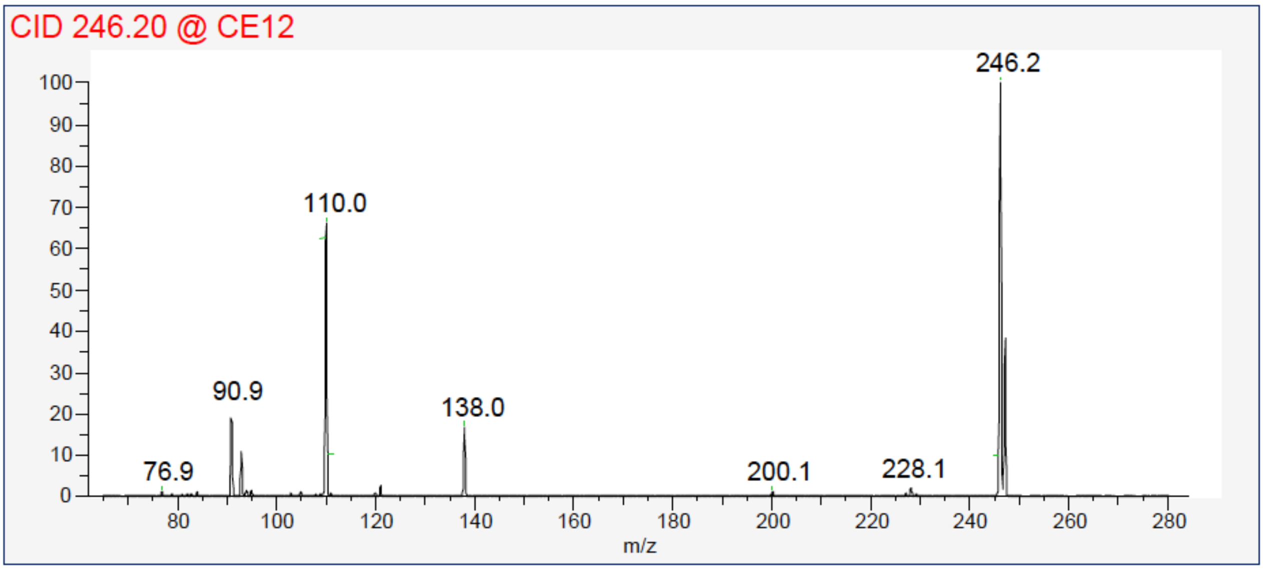

7. DFT Calculations

The two molecular systems for which we have characterized the reaction paths are

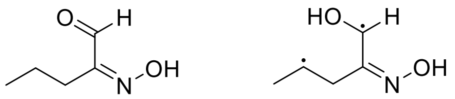

1p

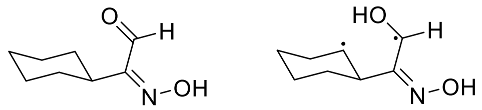

1j

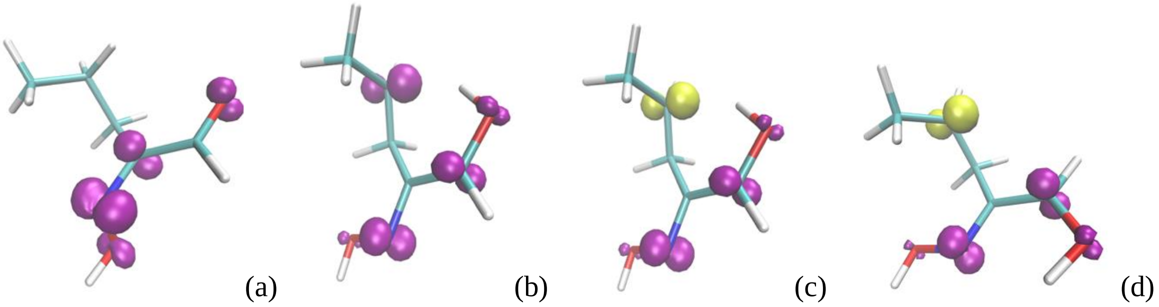

Figure S12. Relevant minimum energy structures along the reaction of compound HIA **1p**. The purple/yellow iso-surface represents the spin density. (a) Carbonilic triplet $\mathbf{T_1(CO)}$; (b) hydroxyl diradical triplet $\mathbf{T_1(OH)}$; (c) hydroxyl diradical singlet $\mathbf{S_0(OH)}$; (d) hydroxyl radical singlet $\mathbf{S_0(OH_\alpha)}$.

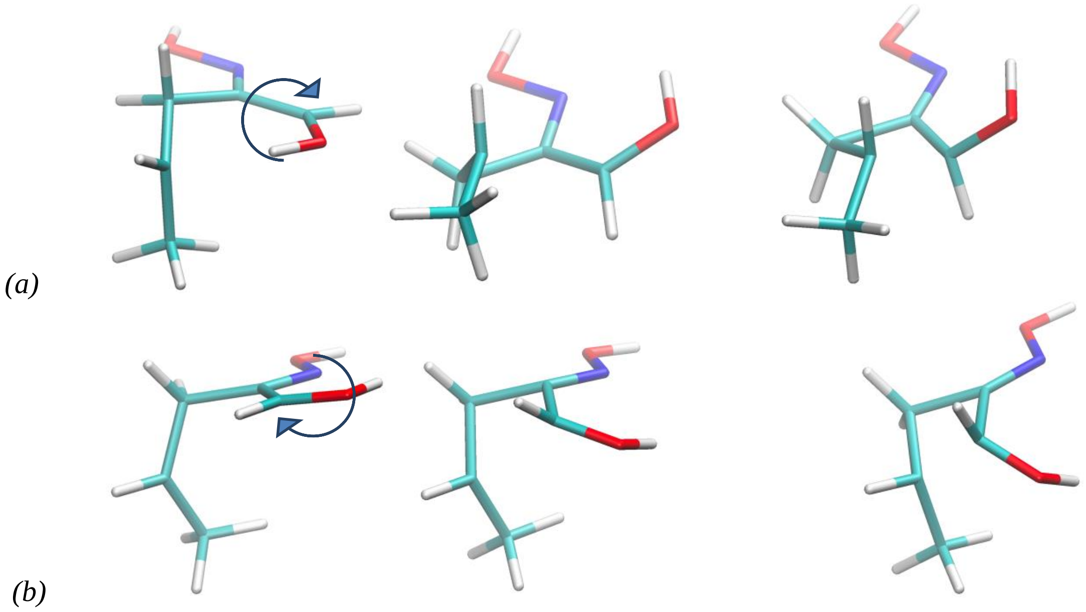

Figure S13. (a) Snapshots of the closing path for compound HIA **1p**. from $\mathbf{S_0(OH)}$ to the *trans* product. (b) Same for the path from $\mathbf{S_0(OH_\alpha)}$ to the *cis* product.

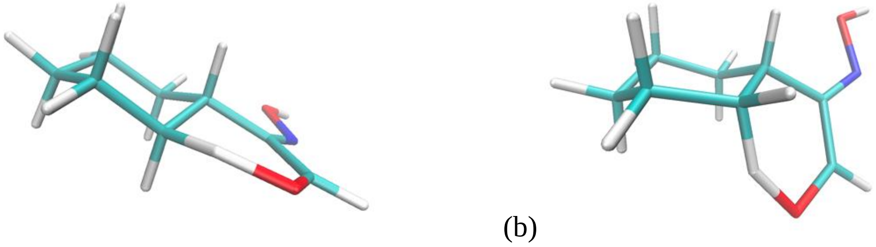

(a) (b)

Figure S14. Transition state structures for **1j** illustrating the two ways in which the H-abstraction can take place: (a) equatorial; (b) axial.

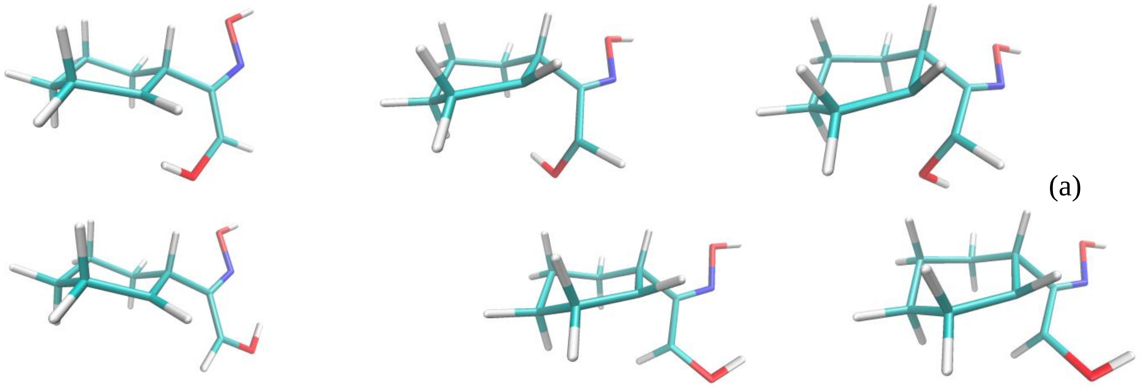

(a)

(b)

Figure S15. Closure paths for **1j**: (a) from $\mathbf{S_0(OH)}$ leading to the endo-cis product; (b) from $\mathbf{S_0(OH_\alpha)}$ leading to the exo-cis product.

Energetic profiles calculated with M062X/cc-pVTZ

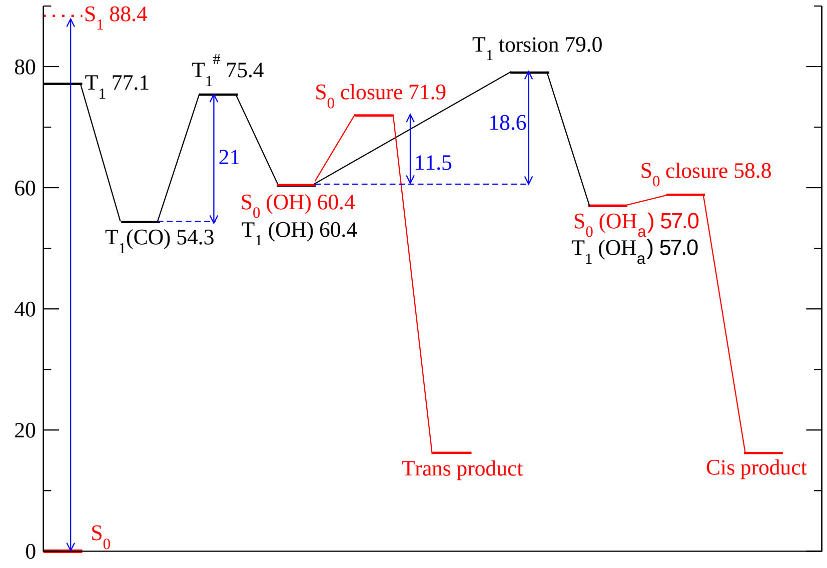

Figure S16. Energetic profile of the reaction of compound HIA **1p**. computed using M06-2X/cc-pVTZ. The triplet stationary points are drawn in black and the singlet ones in red. The numbers represent the energy differences with the ground state S_0 in its minimum geometry and are expressed in kcal/mol.

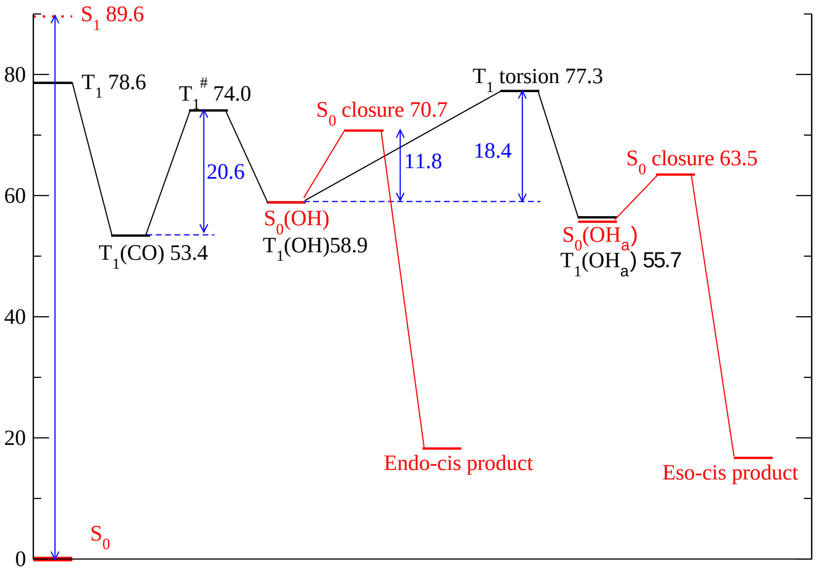

Figure S17. Energetic profile of the reaction of compound **1j** computed using M06-2X/cc-pVTZ. The triplet stationary points are drawn in black and the singlet ones in red. The numbers represent the energy differences with the ground state S_0 in its minimum geometry and are expressed in kcal/mol.

8. IRMPD

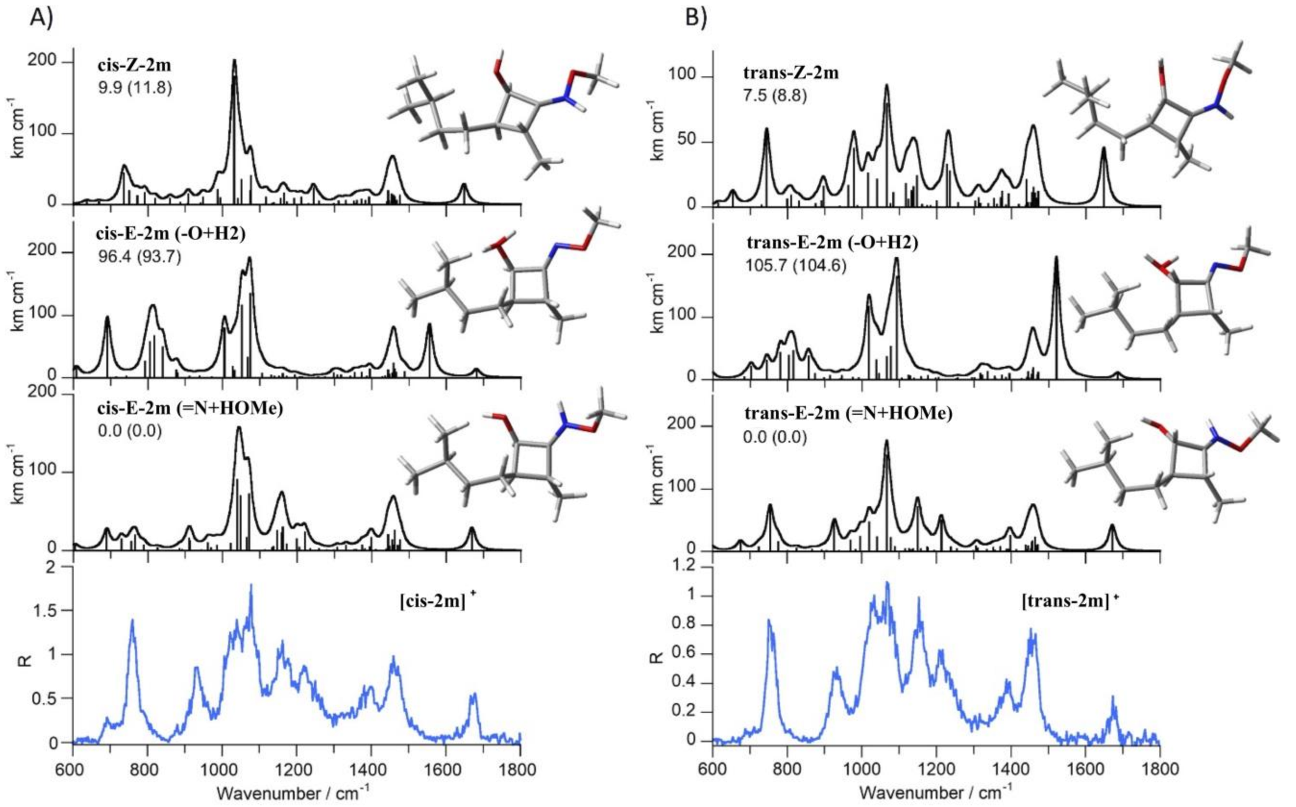

Figure S18. IRMPD spectra of A) [*cis*-**2m** +H]$^+$ and B) [*trans*-**2m** +H]$^+$ (bottom panels), compared with calculated IR spectra for selected structural isomers and protomers. Optimized geometries are reported together with relative free energies (enthalpies) at 298 K in kJ mol^{-1}.

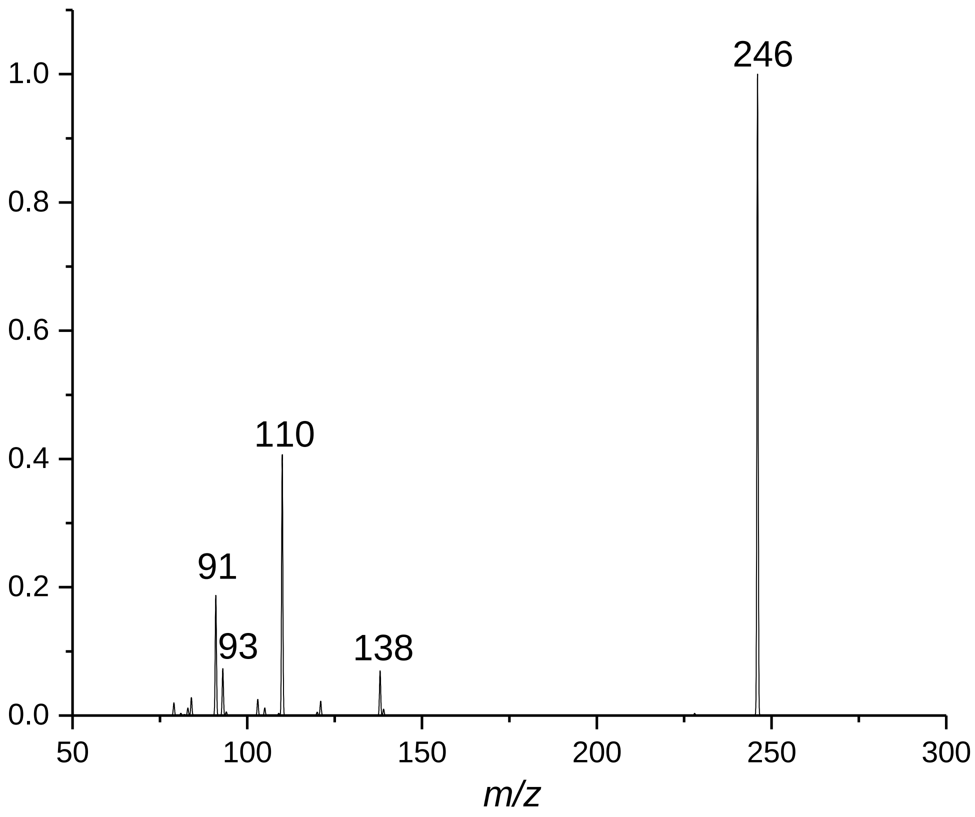

Figure S19. Photofragmentation mass spectrum recorded under irradiation of [**2o**+H]$^+$ at 869 cm^{-1}. Main fragments are at *m/z* 138 (**1**+H- $PhCH_2OH$), 110 (138 -CO), 93 (110 -NH_3) and 91 (93 -H_2).

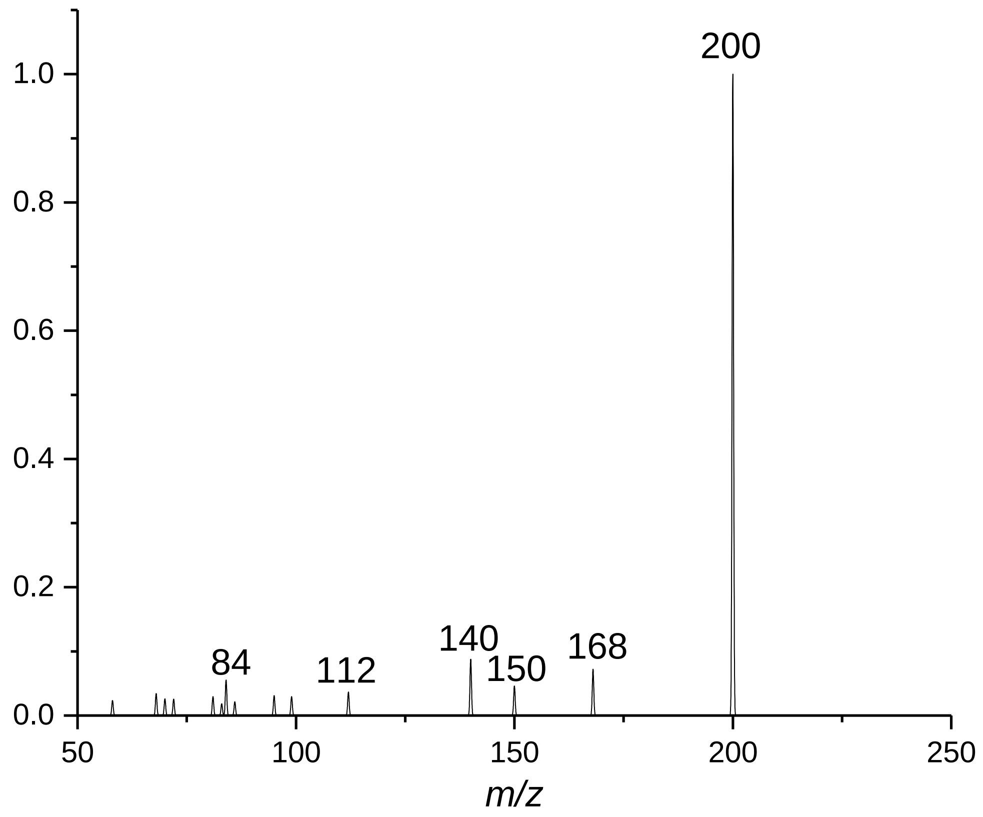

Figure S20. Photofragmentation mass spectrum recorded under irradiation of [*cis*-**2m** +H]$^+$ at 1460 cm^{-1}. Main fragments are at *m/z* 168 (**2**+H - CH_3OH), 150 (168 -H_2O), 140 (168 -CO), 112 (140 -C_2H_4) and 84 (112 - C_2H_4).

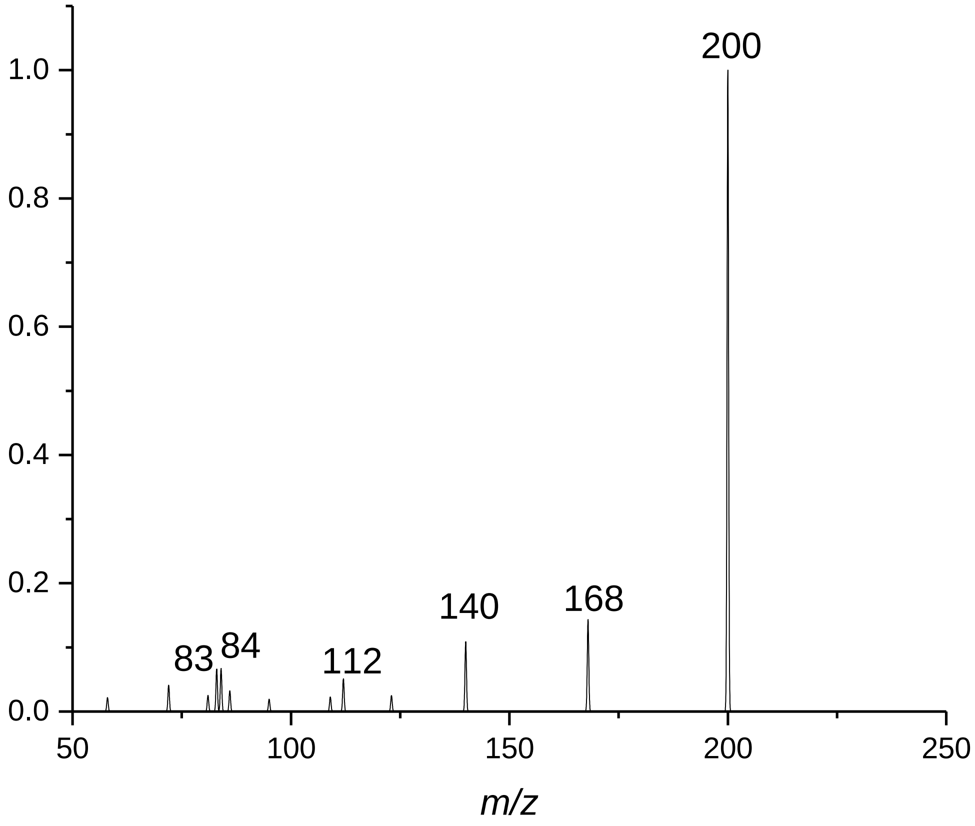

Figure S21. Photofragmentation mass spectrum recorded under irradiation of [*trans*-**2m**+H]$^+$ at 1454 cm^{-1} of [**3**+H]$^+$. Main fragments are at *m/z* 168 (**3**+H - CH_3OH), 140 (168 -CO), 112 (140 -C_2H_4), 84 (112 - C_2H_4) and 83 (112 -CH_3NH_2).

9. HPLC trace of compound (±)-2m (a) and (4R)-2m (b)

a)

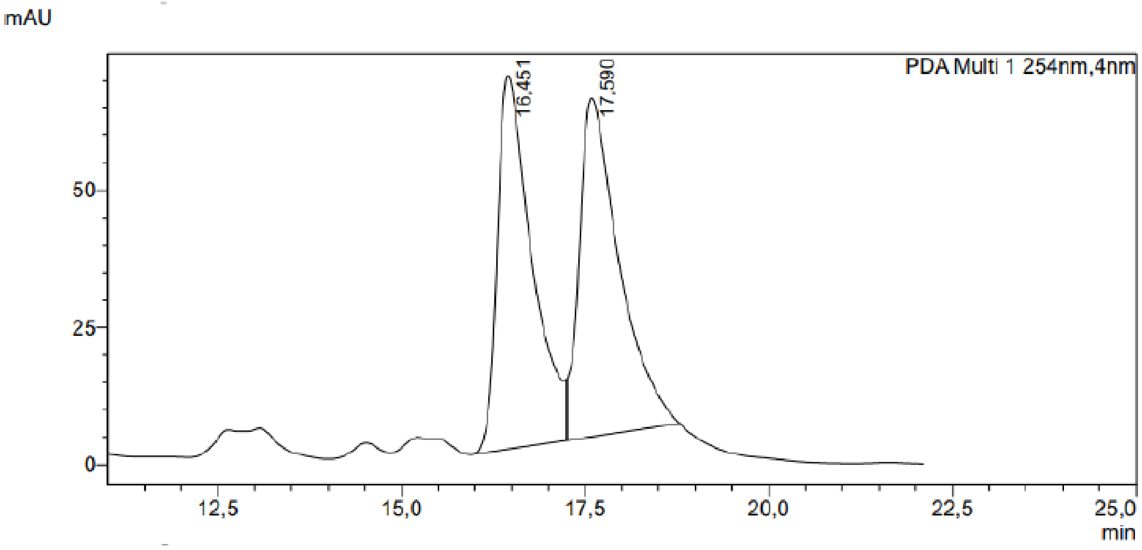

b)

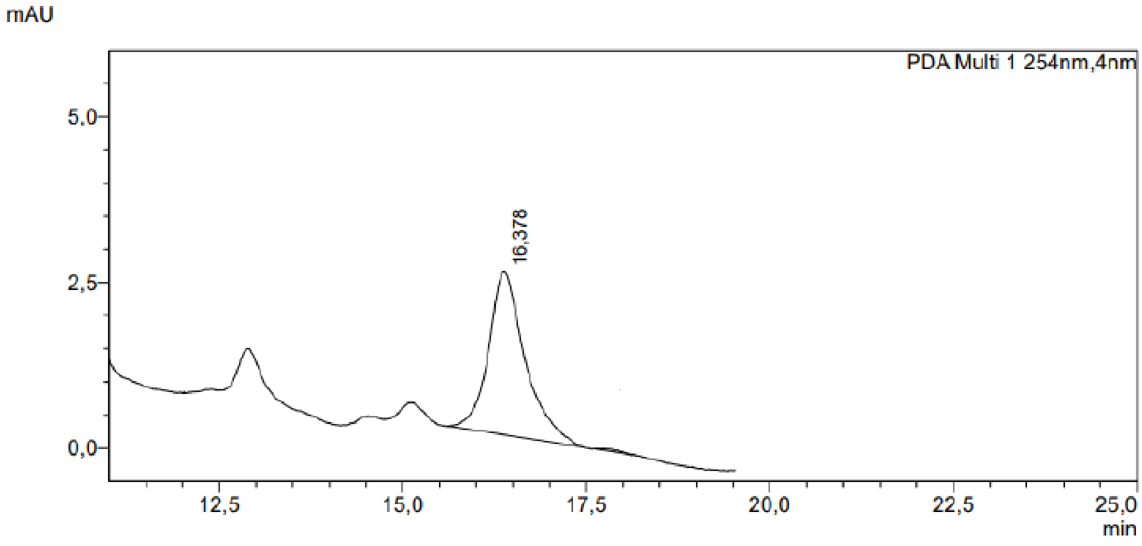

PDA Ch1 254nm

Peak#	Ret. Time	Area	Height	Conc.	Unit	Mark	Name
1	16,378	85250	2457	99,365		M	
2	17,667	544	15	0,635		M	
Total		85795	2472				

10. References

[1] R. H. Bradbury, J. S. Major, A. A. Oldham, J. E. Rivett, D. A. Roberts, A. M. Slater, D. Timms, D. Waterson, *J. Med. Chem.* **1990**, *33*, 2335 - 2342**.**

[2] E. Larionov, L. Lin, L. Guénée, C. Mazet, *J. Am. Chem. Soc.* **2014**, *136*, 16882–16894.

[3] B. Ardiansah, K. Kakiuchi, T. Morimoto, H. Tanimoto, T.Tomohiro., *Chem. Commun.*, **2021**, *57*, 8738 - 8741.

[4]] G. A. Molander, J. A. C Romero, . *Tetrahedron* **2005**, *61*, 2631–2643.

[5] J. W. Amoroso, L. S. Borketey, G. Prasad, N. A. Schnarr, *Org. Lett.* **2010**, *12*, 2330–2333.

[6] H. Hopf, A. Krüger, *Chem. Eur. J.* **2001**, *7*, 4378–4385.

[7] G. Damilano, K. Binnemans, W. Dehaen, *Org. Biomol. Chem.*, **2019**, *17*, 9778-9791.

[8] T. Ikai, M. Okubo, Y. Wada, *J. Am. Chem. Soc.* **2020**, *142*, 3254–3261.

[9] P. J. Gilligan, B. K. Folmer, , R. A. Hartz, S. Koch, K. K. Nanda, S. Andreuski, L. Fitzgerald, K. Miller, W. J. Marshall, *Bioorg. Med. Chem.*, **2003**, *11*, 4093–4102.

[10] Gilead Sciences, Inc.; Y. E. Hu, J. Kaur, R. Mcfadden, J. P. Murry, B. E. Schultz, H. H. Truong, Yu, H. WO2020/176505, 2020, A1.

[11]] P. Gentili, M. Nardi, I. Antignano, P. Cambise, M. D’Abramo, F. D’Acunzo, A. Pinna, E. Ussia, *Chem. Eur. J.*, **2018**, *24*, 7683–7694.

[12] P. Gentili, S. Pedetti, *Chem. Commun.*, **2012**, *48*, 5358–5360.

[13] F. D’Acunzo, L. Carbonaro, A. Dalla Cort, A. Di Sabato, D. Filippini, F. Leonelli, L.; Mancini, P. Gentili, *Eur. J. Org. Chem.*, **2021**, 289 –294.

[14] A. K. Rappe, C. J. Casewit, K. S. Colwell, W. A. III Goddard, W. M. Skiff *J. Am. Chem. Soc.* **1992,** *114*, 10024–10035.
